# Tetramethylphosphinane as a new secondary phosphine synthon

**DOI:** 10.1038/s42004-023-00876-8

**Published:** 2023-04-29

**Authors:** James D. Nobbs, Sigit Sugiarto, Xin Yi See, Choon Boon Cheong, Srinivasulu Aitipamula, Ludger P. Stubbs, Martin van Meurs

**Affiliations:** Institute of Sustainability for Chemicals, Energy and Environment (ISCE2), Agency for Science, Technology and Research (A*STAR), 1 Pesek Road, Jurong Island, Singapore, 627833 Republic of Singapore

**Keywords:** Ligands, Catalyst synthesis, Reactive precursors, Homogeneous catalysis, Synthetic chemistry methodology

## Abstract

Secondary phosphines are important building blocks in organic chemistry as their reactive P—H bond enables construction of more elaborate molecules. In particular, they can be used to construct tertiary phosphines that have widespread applications as organocatalysts, and as ligands in metal-complex catalysis. We report here a practical synthesis of the bulky secondary phosphine synthon 2,2,6,6-tetramethylphosphinane (TMPhos). Its nitrogen analogue tetramethylpiperidine, known for over a century, is used as a base in organic chemistry. We obtained TMPhos on a multigram scale from an inexpensive air-stable precursor, ammonium hypophosphite. TMPhos is also a close structural relative of di-*tert*-butylphosphine, a key component of many important catalysts. Herein we also describe the synthesis of key derivatives of TMPhos, with potential applications ranging from CO_2_ conversion to cross-coupling and beyond. The availability of a new core phosphine building block opens up a diverse array of opportunities in catalysis.

## Abstract (web summary)

Di-*tert*-butylphosphine is an important substituent in many phosphine ligands, however, its heterocyclic analogues have not yet been isolated. Here, the authors report the synthesis and isolation of 2,2,6,6-tetramethylphosphinane on a gram scale starting from ammonium hypophosphite, and its facile use as a building block in ligand construction.

## Introduction

**Fig. 1:**
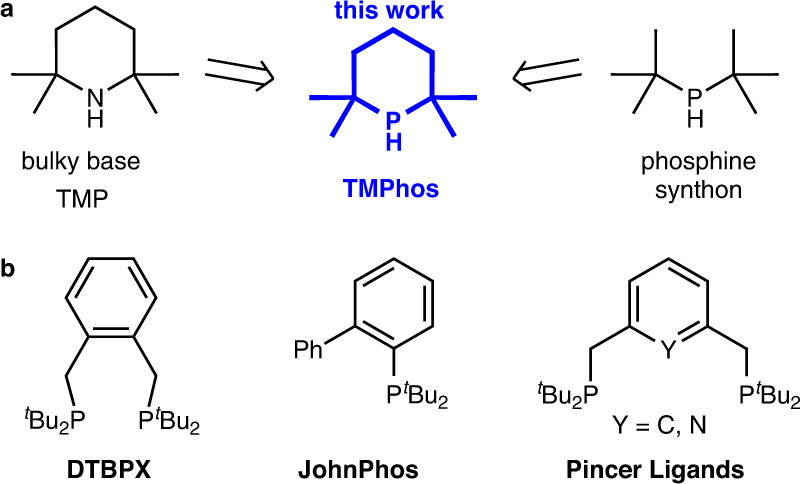
Target phosphine synthon TMPhos. **a** TMPhos (2,2,6,6-tetramethylphosphinane) as the P-analogue of TMP and structurally related to ^*t*^Bu_2_PH. **b** Well-known phosphine ligands containing -P^*t*^Bu_2_.

Over a century ago, the sterically hindered base 2,2,6,6-tetramethylpiperidine (TMP) was first isolated by Franchimont and Friedmann from aqueous ammonia and phorone^1^. TMP and its derivatives would turn out to have widespread use in organic chemistry including the oxidant and radical trap TEMPO^2,3^, the superbase LiTMP^4^, and hindered amine light stabilisers (HALS)^5^. Recently, it has been used as the base component of certain frustrated Lewis Pairs^6^. Yet surprisingly, the synthesis of the phosphorus congener, 2,2,6,6-tetramethylphosphinane (TMPhos, Fig. 1), has not been described until now.

Structurally related to TMPhos is di-*tert*-butylphosphine (^*t*^Bu_2_PH), a bulky secondary phosphine synthon and an important component in many well-known ligands for homogeneous catalysis (Fig. 1). For example, the Alpha process for the commercial production of methyl methacrylate employs di-*tert*-butylphosphino-*o*-xylene palladium catalyst (Pd-DTBPX) in one of the key steps: methoxycarbonylation of ethylene to methylpropanoate^7^. JohnPhos from the Buchwald ligand family has multiple applications in Pd–catalysed cross-coupling reactions, such as Suzuki–Miyaura reactions between boronic acids and aryl halides^8^, amination of aryl halides and triflates^9,10^, as well as arylation of thiophenes^11^. Pincer ligands^12^ also have widespread applications in homogeneous catalysis and have undergone a renaissance in recent decades due to their ability to participate in metal-ligand cooperation^13^. In many examples the ^*t*^Bu_2_P motif is an important component of such pincer ligands^14,15^.

**Fig. 2:**
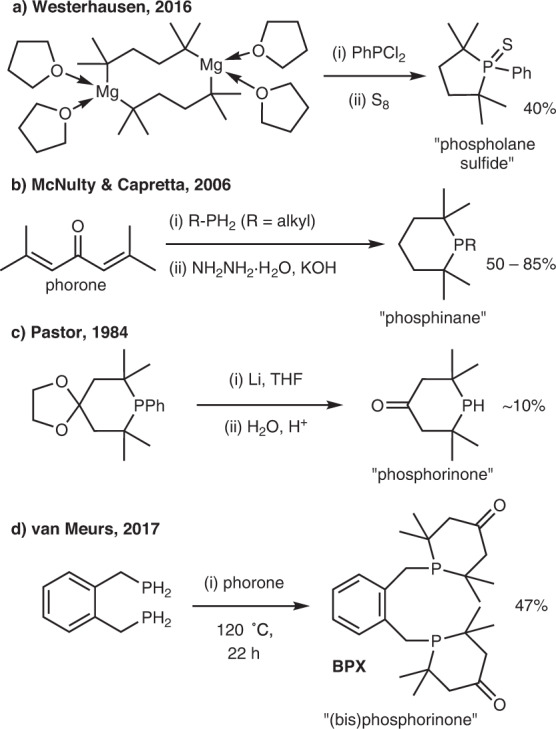
Known bulky phospholanes and phosphinanes. **a** Phospholane sulfide, **b** phosphinanes, **c** phosphorinones, **d** bis(phosphorinone)xylene, BPX.

^*t*^Bu_2_PH is a widely used building block in the design of many ligands. Yet its 5- and 6-membered heterocyclic secondary phosphine analogues, 2,2,5,5-tetramethylphospholane and TMPhos have not yet been isolated. Sulfide derivatives of the former were detected from the reaction between the corresponding di-Grignard and PhPCl_2_ (Fig. 2a), but the secondary phosphine has thus far remained elusive^16^. McNulty and Capretta obtained a series of substituted *tertiary* phosphinanes from the reaction between R-PH_2_ and phorone, followed by reduction of the ketone group, Fig. 2b^17^. They found that this family of ligands was tunable, cheap and efficient in cross-coupling reactions, like popular ligands such as ^*t*^Bu_3_P. The six-membered heterocycle 2,2,6,6-tetramethylphosphinan-4-one has been prepared by a lithium cleavage of the corresponding phenyl derivative, Fig. 2c. This bulky secondary phosphine is probably the closest known structure to TMPhos, however, the yield of the reaction was low (~10%) most likely due to competing lithium cleavage of the P-*tert-*alkyl bonds^18^. Van Meurs and co-workers have also recently demonstrated that the bulky 1,2-bis(2,2,6,6-tetramethylphosphorinan-4-one)xylene (BPX, Fig. 2d), is a more effective ligand in the isomerising carbonylation of alkenes, compared to its acyclic analogue^19^.

Heterocyclic phosphines offer unique properties that can be tuned by ring size, substituents and functional groups^20–23^. In order to access more ligands incorporating bulky phosphinanes, we targeted the synthesis of the secondary phosphine synthon, TMPhos. Herein, we report the synthesis and isolation of TMPhos on a multigram scale starting from ammonium hypophosphite (NH_4_H_2_PO_2_), an abundant low-cost reducing agent used in metallurgy^24^. We demonstrate the facile use of TMPhos as a building block in the construction of various ligands and compare them to structurally similar commercial counterparts.

## Results and discussion

### Initial synthesis of TMPhos

2,2,6,6-Tetramethylpiperidine can be made via conjugate addition of ammonia and phorone to give 2,2,6,6-tetramethyl-4-piperidinone, followed by a Wolff–Kishner reduction of the ketone^4^. Our approach was based on a similar strategy: a ring forming reaction between phorone and a suitable phosphorus precursor; followed by the subsequent reduction of the ketone group to furnish TMPhos. Phorone can be readily obtained by the aldol condensation of acetone^25^. However, Welcher and Day had attempted the Michael addition of phosphine (PH_3_) to phorone 60 years ago and reported that no reaction occurred^26^. Furthermore, phosphine gas is highly toxic, and pyrophoric, making further investigations into this reaction hazardous, in spite of recent progress being made in the development of procedures using in situ generated PH_3_^27^.

**Fig. 3:**
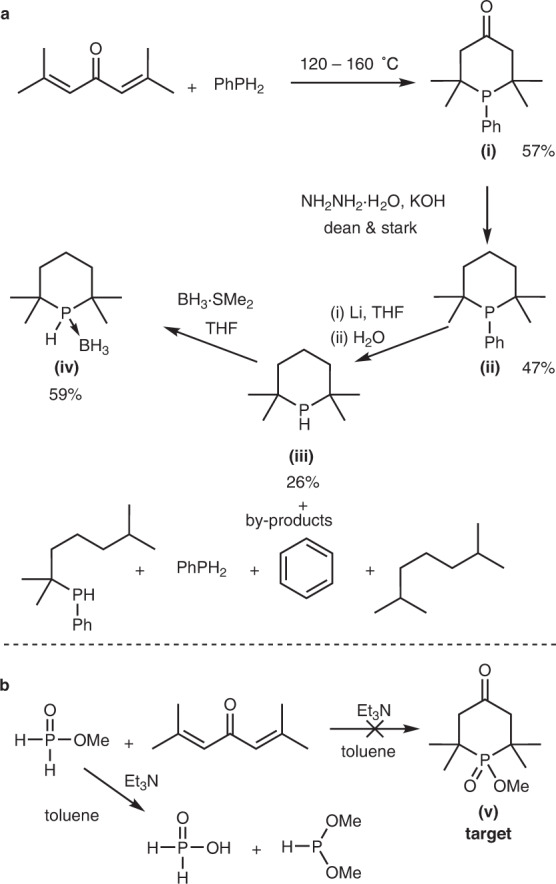
Initial attempts to isolate TMPhos. **a** Synthesis of TMPhos via Li cleavage of 1-phenyl-2,2,6,6-tetramethylphosphinane. **b** Attempted synthesis of **(v)**.

Aiming to avoid handling PH_3_ gas we first attempted to obtain TMPhos via a Li–cleavage of 1-phenyl-2,2,6,6-tetramethylphosphinane in an adaptation of Pastor’s procedure^18^, see Fig. 3a. We first obtained phenyl phosphorinone **(i)** from the condensation of phorone and PhPH_2_^26^, followed by Wolff–Kishner reduction to produce phenyl phosphinane **(ii)**^17^. Reductive cleavage of aryl-phosphorus bonds using alkali metals can be used to generate lithium phosphide species which upon hydrolysis will generate secondary phosphines^28^. The reaction between **(ii)** and Li at 5 °C furnished the desired secondary phosphine **(iii)** (^31^P *δ* = −9.1 ppm) but only as a minor product (~26% yield). The major product, 2,6-dimethylheptan-2-yl(phenyl)phosphine, has a slightly upfield ^31^P chemical shift of −11.0 ppm. This product arises from a competing ring opening reaction, as Li preferentially cleaves phosphorus bonded to the *tertiary* carbon atom^28^. The ring-opened compound undergoes a second cleavage to generate PhPH_2_ as well as 2,6-dimethylheptane. Despite being only a minor product, the desired secondary phosphine, TMPhos, could be isolated as the borane adduct by first performing vacuum distillation to separate it from the high boiling phosphines, followed by addition of borane dimethylsulfide complex to generate TMPhos·BH_3_, **(iv)**.

### Improved synthesis of TMPhos

Since the atom economy and reaction yield from the Li cleavage of **(ii)** were both poor, this route was not attractive for obtaining sufficient amounts of TMPhos to explore its downstream chemistry. Therefore, we continued to investigate alternative routes for the synthesis of TMPhos. There are a number of primary phosphine surrogates that mimic the reactivity of PH_3_, such as methyl hypophosphite (H_2_PO_2_Me) and bis(trimethylsilyl)phosphonite ((Me_3_SiO)_2_PH) that can both be derived from commercially available and relatively benign hypophosphorous acid. By this route, we obtained H_2_PO_2_Me from the alkylation of hypophosphorous acid with trimethyl orthoformate^29^. However, in the presence of NEt_3_ we observed no reaction between phorone and H_2_PO_2_Me, but instead a base-catalysed disproportionation to hypophosphorous acid and dimethoxyphosphine occurred, see Fig. 3b.

**Fig. 4:**
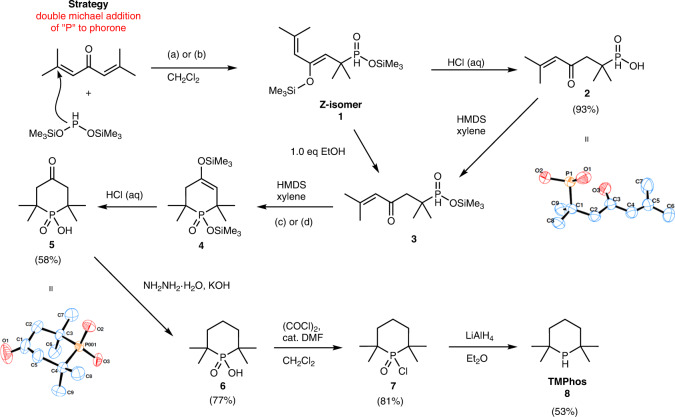
Synthesis of TMPhos (**8**). **a** Hünig’s Base/TMSCl/NH_4_H_2_PO_2_, **b** HMDS/NH_4_H_2_PO_2_, **c** 125 °C, 72 h, **d** *microwave heating* at 220 °C, 90 min.

We therefore turned our attention to the more reactive (Me_3_SiO)_2_PH, which had been shown to undergo Michael additions to conjugated alkenes^30–32^, and nucleophilic substitutions with alkyl halides^33^ to furnish mono- or di-substituted phosphinic acids and even heterocycles. As (Me_3_SiO)_2_PH is pyrophoric it is typically generated in situ. We generated (Me_3_SiO)_2_PH either by the reaction between NH_4_H_2_PO_2_ and hexamethyldisilazane (HMDS)^31^ or with NH_4_H_2_PO_2_ and trimethylsilyl chloride (TMSCl) in the presence of Hünig’s base (Fig. 4)^30^. The formation of (Me_3_SiO)_2_PH is evidenced by a doublet in the ^31^P NMR spectrum (*δ* = 141.7 ppm, ^1^*J*_HP_ = 175.4 Hz). In both cases the (Me_3_SiO)_2_PH generated in situ reacted readily at RT with phorone producing intermediate **1**. In this intermediate, one of the TMS groups has migrated to form a silyl enol ether. This was reflected by two inequivalent TMS groups in ^29^Si{^1^H} NMR, of which only one is coupled to ^31^P (d, 11.0 Hz) while the other remains a singlet, and was further confirmed by ^1^H-^29^Si 2D HMBC. The *Z*-isomer was determined to be the major product from 2D NOESY experiment, in which a distinctive NOE was observed between one terminal methyl group and the (distal) alkene proton of the enol ether (Supplementary Fig. 39 and Supplementary Data 1). In the ^1^H NMR spectrum the PH appears as a doublet at 6.96 ppm, with a large coupling constant (^1^*J*_HP_ = 544.2 Hz), consistent with that of a hypophosphite^34^.

The enolisation of the ketone group prevents a second Michael addition and ring closure from occurring due to the disruption of the conjugation. It could, however, be easily deprotected using 2 M aq. HCl to furnish enone-phosphinic acid **2** (^31^P δ = 46.1 ppm) in 93% yield on a multigram scale (>40 g). SC-XRD of compound **2** confirmed the expected structure (see Fig. 4 and Supplementary Fig. 3). We achieved the ring closure of **2** by addition of HMDS while monitoring the reaction by ^31^P NMR spectroscopy. Upon addition of HMDS to **2** the silyl ester **3** forms immediately, and upon heating this gradually forms the cyclised silyl intermediate **4**.

The ring closure (6-*endo*-trig) is sluggish likely due to the steric constraints. Initially attempts at ring closure in 1,2-dichloroethane (DCE) at 70 °C required two weeks to approach full conversion. Later we changed the solvent to xylenes, which allowed for a reaction temperature of 125 °C and enabled good conversion (>80%) in 3 days. Alternatively, full conversion can be obtained in just 90 min using a microwave reactor at 220 °C, with a comparable isolated yield of 40%. The reaction was typically carried out at a concentration of ~0.2 M of phosphinic acid in xylenes) in order to minimise potential intermolecular side reactions; at higher concentration (0.6 M) a polymeric precipitate was observed. We also found that intermediate **3** can be formed via a partial hydrolysis of intermediate **1**, since the silyl enol ether will preferentially hydrolyse in the presence of one equivalent of protic solvent such as ethanol. This enables a one-pot synthesis of 2,2,6,6-tetramethylphosphorinic acid, **5**, however, we found that less side-products were obtained if the phosphinic acid **2** was first isolated and purified. After acid hydrolysis of intermediate **4**, bifunctional bulky heterocyclic **5** was isolated in 58% yield from **2** at >10 g scale. Single crystals of **5** were grown by evaporation of a solution of the compound in acetone and a representation of the molecular structure is shown in Fig. 4 and Supplementary Fig. 4.

The conversion of **5** to TMPhos involves the reduction of both the ketone and phosphinic acid functional groups. We first reduced the ketone group using a standard Wolff–Kishner procedure to give 2,2,6,6-tetramethylphosphaninic acid, **6**, in good yield (77%). Direct reduction of phosphinic acids to phosphine has been reported using Ph_2_SiH_2_^35^ or PhSiH_3_^30^. However, these approaches gave low or no yield of the desired secondary phosphine when applied to compound **6**, presumably due to either the increased steric bulk or the more basic P character (as compared to aromatic P compounds). Conversion to TMPhos was instead achieved via reduction of the corresponding phosphinic chloride **7**, obtained by chlorination using (COCl)_2_ in the presence of catalytic DMF. After LiAlH_4_ reduction, TMPhos, **8**, was obtained by distillation in moderate yield (53%). We have performed all synthetic steps to TMPhos on at least 5 g scale (reactant) with some on significantly larger scales. For safety reasons, at lab scale some steps were difficult to scale beyond certain thresholds (e.g. Wolff–Kishner reduction and LiAlH_4_ reduction) and we are working on the process development of these steps.

### Properties and reactivity of TMPhos

**Fig. 5:**
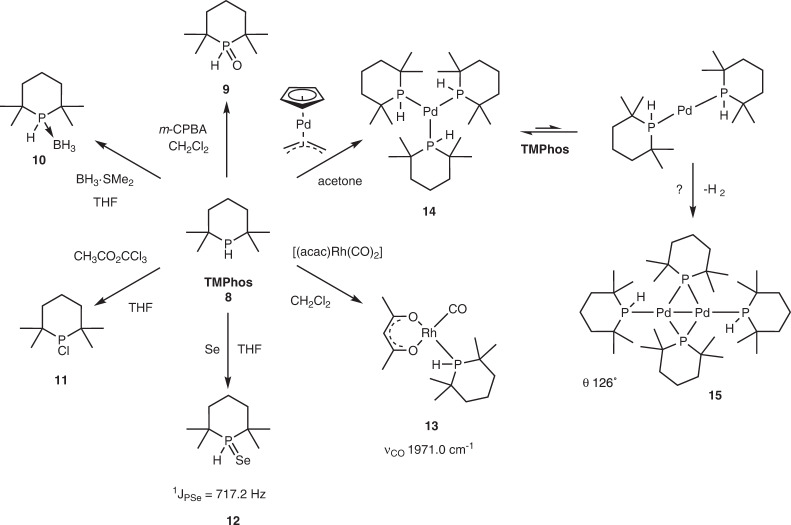
Conversion of TMPhos to derivatives and its coordination compounds. Conversion of TMPhos to the corresponding oxide (**9**), borane adduct (**10**), chlorophosphine (**11**), selenide (**12**) and rhodium (**13**), and palladium complexes (**14**, **15**).

TMPhos is a colourless liquid with a ^31^P chemical shift of −9.1 ppm (^1^*J*_PH_ = 200.0 Hz), significantly upfield of ^*t*^Bu_2_PH (*δ* = 20.6 ppm, ^1^*J*_PH_ = 193.0 Hz). Six-membered phosphinanes typically adopt ring shapes typical of cyclohexanes, with chair confirmations normally observed in solid state structures (vide infra)^22^. Compared to ^*t*^Bu_2_PH, TMPhos is notably more resistant to oxidation. When a solution of TMPhos in CDCl_3_ was exposed to air, remarkably no oxidation was detected in the first 24 h. The slower oxidation of TMPhos was gratifying since air oxidation is a categorical weakness of alkyl phosphines and may arise from a less basic P in TMPhos compared with ^*t*^Bu_2_PH (vide infra). Even after exposure to air for 5 days only 20% of TMPhos had oxidised to the phosphine oxide **9** as the sole product (which could also be generated cleanly using *m*-CPBA, Fig. 5). In contrast, 80% of ^*t*^Bu_2_PH had decomposed to a mixture of products resulting from oxygen insertion into a P-^*t*^Bu bond^36^ forming *t*-butylphosphinate which hydrolyses to *t*-butylphosphinic acid (Supplementary Figs. 1 and 2). The heterocyclic conformation of TMPhos presumably prevents oxygen insertion from occurring which is a clear demonstration of its unique properties.

To demonstrate the versatility of TMPhos as a building block in organic synthesis, several TMPhos derivatives **10** and **11** were also synthesised (Fig. 5). For benchtop use, air-stable tetramethylphosphinane borane complex TMPhos·BH_3_ (compound **10**) was obtained by stirring equimolar amounts of TMPhos with BH_3_·SMe_2_. Dialkylchlorophosphines are also synthetically versatile synthons, and chlorination of TMPhos with one equivalent of methyl trichloroacetate affords the chlorophosphine **11** in high yield (72%).

We synthesised the phosphorus selenide **12** since ^1^*J*_PSe_ coupling constants can give an indication of the basicity of phosphines^37^. The ^1^*J*_PSe_ of compound **12** (717.2 Hz) is larger than reported for the corresponding ^*t*^Bu_2_P(H) = Se (704 Hz) which suggests TMPhos is less basic^38^. This was supported by a ν(C‍ ≡ ‍O) of 1971.0 cm^−1^ for [(TMPhos)Rh(CO)(acac)] **13** compared to 1963.4 cm^−1^ for [(^*t*^Bu_2_PH)Rh(CO)(acac)] (see Supplementary Methods). This is consistent with observations for similar compounds where it was postulated that a smaller C-P-C angle resulting from the ring conformation contributes to a less basic P^19^.

**Fig. 6:**
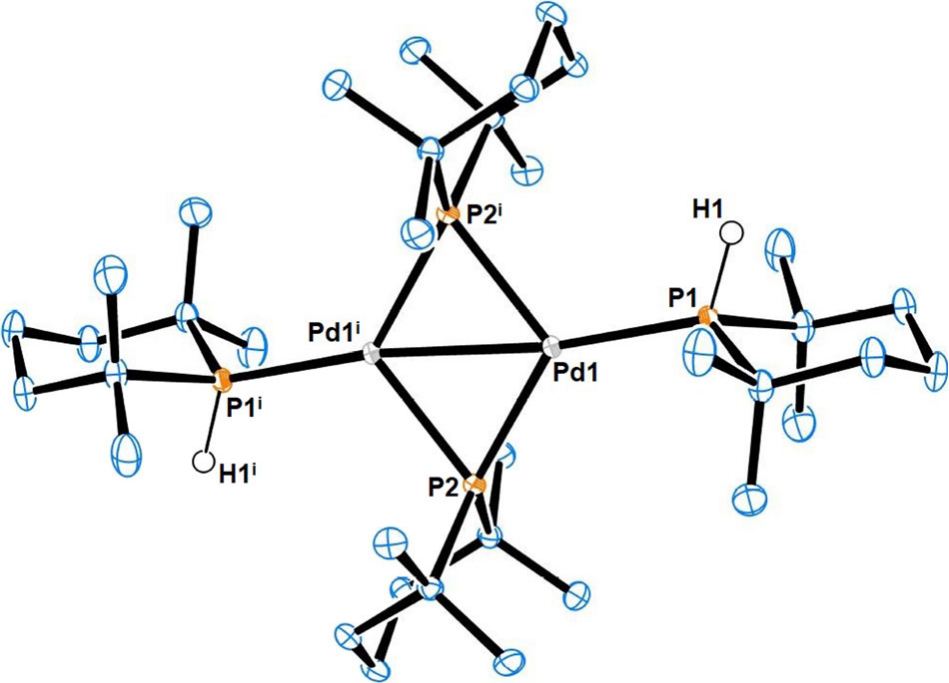
Molecular structure of compound **15**. Selected bond lengths (Å) and angles (°): Pd1-Pd1^i^ 2.6053(3), Pd1-P1 2.2686(3) Pd1-P2 2.3124(3), Pd1-P2^i^ 2.3426(3), P1-Pd1-P2 131.267(9), P1-Pd1-P2^i^ 116.715(9), P2-Pd1-P2^i^ 112.017(7), Pd1-P2-Pd1^i^ 67.982(7), P1-Pd1-Pd1^i^ 172.166(8), P2-Pd1-Pd1^i^ 56.562(7), P2^i^-Pd1-Pd1^i^ 55.455(7), C1-P1-C5 105.34(5), and C10-P2-C14 104.64(4).

In comparison with tertiary phosphines, palladium complexes bearing secondary phosphines are relatively uncommon owing to their reactive P–H bond. Homoleptic complexes of secondary phosphines are rare, with [(^*t*^Bu_2_PH)_3_Pd^(0)^] and [(Ph_2_PH)_4_Pd^(0)^] being the only reported examples for palladium^39,40^. We were therefore interested to obtain a homoleptic complex of Pd bearing TMPhos ligands. [(TMPhos)_3_Pd^(0)^] **14** was synthesised following the same procedure reported for [(^*t*^Bu_2_PH)_3_Pd^(0)^]^39^. The addition of excess TMPhos and allyl(cyclopentadienyl)palladium(II) was accompanied by the reductive elimination of 5-allyl-1,3-cyclopentadiene to give **14** as a yellow solid in 67% yield. In solution, **14** gave a broad doublet at 42.1 ppm (^1^*J*_PH_ = 246 Hz) in its ^31^P NMR spectrum, upfield of the corresponding ^*t*^Bu_2_PH complex (δ = 54.5 ppm, ^1^*J*_PH_ = 256 Hz). In solution Leoni found evidence of a rapid equilibrium between [(^*t*^Bu_2_PH)_3_Pd] and [(^*t*^Bu_2_PH)_2_Pd]^39^. The latter thermally transforms to dimeric [Pd(*μ*-P^*t*^Bu_2_)(HP^*t*^Bu_2_)]_2_ with the concomitant loss of H_2_. We found that complex **14** underwent a similar transformation. A solution of **14** kept at RT formed red single crystals of bimetallic complex **15**, its identity being confirmed by SC-XRD (Fig. 6 and Supplementary Fig. 5). The structure contains two bridging TMPhos ligands and two terminally bound TMPhos ligands with H atoms in the axial position. The Pd1-Pd1^i^ bond length of 2.61(1) Å is statistically comparable with corresponding [Pd(μ-P^*t*^Bu_2_)(HP^*t*^Bu_2_)]_2_ complex where it was 2.60(1) Å^41^. Likewise the Pd-P1 and Pd-P2 bond lengths of 2.27(1) and 2.31(1) Å, respectively, were also comparable to the ^*t*^Bu_2_PH complex, where they were 2.29(1) and 2.34(1) Å. The most noticeable difference is the anticipated C-P-C bond angle contraction in the phosphinane rings. In the case of terminal phosphinane the C1-P1-C5 angle was 105.3(1)° whereas the bridging phosphinane had an even smaller C10-Pd-C14 angle of 104.6(1)°. These angles are noticeably smaller than the corresponding angles in the ^*t*^Bu_2_PH complex which were 112.0(3)° and 111.3(3)°, respectively. These differences are reflected in a smaller cone angle calculated for the terminal TMPhos-Pd (126°) compared with ^*t*^Bu_2_PH-Pd (134°), see Supplementary Methods Section 5.

**Fig. 7:**
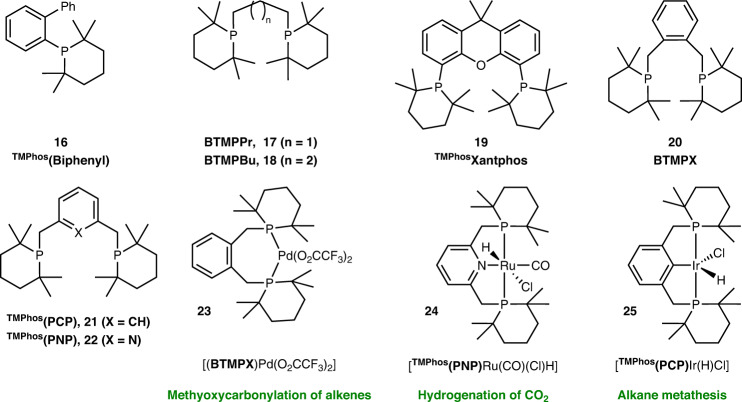
Ligands and complexes incorporating TMPhos synthons. Ligands incorporating TMPhos (**16**–**22**) and associated metal complexes (**23**–**25**) with potential catalytic applications (in green).

Using TMPhos we were also able to readily construct a variety of pro-ligands based on well-known phosphines as shown in Fig. 7. Simple alkyl phosphinanes, such as those shown in Fig. 2b, have been demonstrated to be effective ligands in palladium catalysed cross-coupling reactions^17^, and JohnPhos is commercialised for the same purpose^42^. We therefore synthesised the monophosphine ^TMPhos^(Biphenyl), **16** which is a combination of a phosphinane ring and a classic Buchwald biphenyl substituent. To showcase TMPhos-based bidentate ligands, we first prepared the simple bis-phosphines BTMPPr **17** and BTMPBu **18** with propyl and butyl backbones respectively. The ^*t*^Bu_2_P versions of these ligands have been applied in carbonylation of alkenes^43^ and polymerisation of phenylacetylene^44^. Next we constructed a bis-phosphine bearing the well-known xanthene backbone ^TMPhos^(Xantphos) **19** as Xantphos has many applications in coordination chemistry and catalysis^45^. For example, ^*t*^Bu-Xantphos has been shown to stabilise Ni^I^ alkyl complexes that rapidly insert CO_2_ to form the corresponding Ni-carboxylate species^46^. We also made bis(tetramethylphosphinane)xylene (BTMPX) **20** and its SC-XRD data is shown in Supplementary Fig. 6. This bis(phosphine) is analogous to DTBPX, a ligand used industrially and recently reviewed^47^. Finally we synthesised the pincer ligands ^TMPhos^(PCP) and ^TMPhos^(PNP), (**21** and **22**), as pincer ligands are now well established in homogenous catalysis with an abundance of applications^48,49^. The above examples were chosen as their ^*t*^Bu_2_P analogues are well known, in many cases affording highly active catalysts in a variety of different reactions. In all cases the TMPhos derivatives possess significantly upfield ^31^P chemical shifts: between 16 to 24 ppm more negative than their ^*t*^Bu_2_P counterparts. This is likely a consequence of the γ-substituent effects imposed by the ring, rather than a simple reflection of the electron-donating power of the phosphine^50^.

**Fig. 8:**
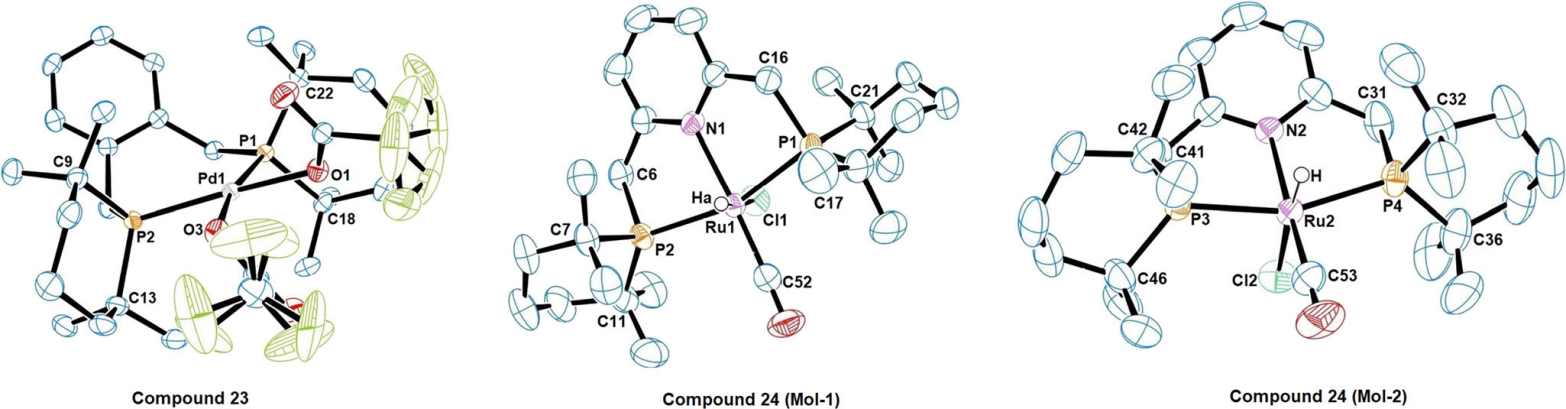
Molecular structures of TMPhos-containing metal complexes. ORTEP representations for the crystal structures of [(BTMPX)Pd(O_2_CCF_3_)_2_] **23** and [^TMPhos^(PNP)Ru(CO)(Cl)H] **24**, drawn at 50% probability. The fluorine atoms of the CF_3_ groups exhibit rotational disorder in the crystal structure.

We went on to explore the coordination chemistry of selected pro-ligands, targeting variations of well-known pre-catalysts. The first example, [(BTMPX)Pd(O_2_CCF_3_)_2_] **23** was synthesised from [(BTMPX)PdCl_2_] (**S3**, Supplementary Methods and Supplementary Fig. 7) and investigated due to its similarity to the commercially relevant Mitsubishi–Lucite catalyst. Its molecular structure is shown in Fig. 8 and Supplementary Fig. 8, together with selected key bond lengths and angles in Table 1. The complex adopts a four-coordinate square planar geometry. The P-Pd-P angle of 101.9(1)° is slightly larger than for similar complexes like [(DTBPX)Pd(O_3_SMe)_2_]^51^ and [(BPX)Pd(O_2_CCF_3_)_2_]^19^, where the bite angles are 100.6(1)° and 100.1(1)°, respectively. As seen in complex **15**, smaller C-P-C angles were observed in the phosphinane ring. The C(^*t*^Bu)-P-C(^*t*^Bu) angle in the DTBPX analogues is about ~110°^52^, whereas for **23** the bond angles for C18-P1-C22 and C9-P2-C13 are 105.8(1)° and 106.8(1)° respectively. Taken together, it follows that complex **23** has a marginally smaller buried volume (%*V*_Bur_ = 50.0%) compared to that calculated for structurally related [(DTBPX)Pd(O_3_SMe)_2_] (%*V*_Bur_ = 53.1%), see Supplementary Figs. 11 and 14 for topographic steric maps^51,53^.

**Table 1:** Selected bond lengths (Å) and angles (°) of [(BTMPX)Pd(O_2_CCF_3_)_2_], **23**, and [^TMPhos^(PNP)Ru(CO)(Cl)H], **24**.

[(BTMPX)Pd(O_2_CCF_3_)_2_], 23				[^TMPhos^(PNP)Ru(CO)(Cl)H], 24					
Bond lengths (Å)		Bond angles (°)		Bond lengths (Å)			Bond angles (°)		
					Molecule-1	Molecule-2		Molecule-1	Molecule-2
Pd1-P1	2.2964(6)	P1-Pd-P2	101.90(2)	Ru1-P1	2.3285(8)	2.3412(8)	P1-Ru1-P2	159.92(3)	159.68(4)
Pd1-P2	2.3147(7)	P1-Pd1-O1	87.64(5)	Ru1-P2	2.3289(8)	2.3379(9)	N1-Ru1-C52	175.52(13)	173.22(14)
Pd1-O1	2.0698(17)	P1-Pd1-O3	168.17(5)	Ru1-Cl1	2.5463(8)	2.5471(9)	C17-P1-C21	105.23(19)	104.77(18)
Pd1-O3	2.0974(17)	P2-Pd1-O1	170.44(5)	Ru1-N1	2.154(3)	2.176(3)	C7-P2-C11	104.46(18)	105.1(2)
		C22-P1-C18	105.80(11)	Ru1-C52	1.843(4)	1.836(4)			
		C9-P2-C13	106.82(11)						

We also prepared the ruthenium hydride complex [^TMPhos^(PNP)Ru(CO)(Cl)H] **24**. The ^*t*^Bu_2_P variant of this complex is an effective catalyst for the reversible hydrogenation of CO_2_ to formates, giving a TOF in excess of 1 million^54^. In our complex, the hydride signal appears as a triplet at −15.05 ppm (^2^J_HP_ = 19.2 Hz) in the ^1^H NMR spectrum, very similar to the hydride signal in the corresponding ^*t*^Bu_2_P complex (−15.22 ppm, ^2^J_HP_ = 19.4 Hz). Single crystals were obtained from the vapour diffusion of pentane into a saturated solution of the complex in CH_2_Cl_2_. The crystal structure contained two crystallographically independent complexes per unit cell (Fig. 8 and Supplementary Figs. 9-10). In one molecule there is one phosphorus above and below the plane of the pyridine ring, by 0.77 Å and 0.57 Å respectively. By contrast in the second molecule both P atoms lie on the same side of the pyridine ring by 0.55 Å and 0.91 Å respectively. The P-Ru-P angles of 159.9(1)° and 159.7(1)° are slightly larger than that for the corresponding ^*t*^Bu_2_P complex at 158.4(1)°^55^, whereas the N-Ru-CO angles of 175.5(1)° and 173.2(1)° are quite compressed as compared to an angle of 178.5(1)° in the ^*t*^Bu_2_P version. Complex **24** has a slightly smaller %*V*_Bur_ of 51.9% compared to 53.3% for the analogous ^*t*^Bu_2_P complex^55^, see Supplementary Figs. 12, 13, and 15.

Finally, we prepared the TMPhos analogue of the classic Shaw Ir-^*t*Bu^PCP pincer complex, [^TMPhos^(PCP)Ir(H)Cl], complex **25**. The TMPhos variant, **25**, possesses a broad hydride resonance in its ^1^H NMR spectrum (−40.44 ppm) which is downfield compared to the ^*t*^Bu_2_P complex (−43.37 ppm in CD_2_Cl_2_). This suggests a decreased electron density at the metal centre when TMPhos is used. Nearly half a century ago, Shaw’s seminal work on PCP pincers recognised the special properties conferred by bulky tertiary phosphine ligands^56^. These include the ability to promote hydride formation and metalation reactions, as well as the stabilisation of coordinative unsaturation^56^, anticipating the huge contribution bulky pincer ligands have subsequently made in catalysis. We therefore expect TMPhos ligands to also have useful catalytic applications and work is ongoing to investigate the use of **16**–**25** in this regard.

## Conclusion

We have developed a multigram synthetic route to a bulky secondary heterocyclic phosphine synthon, TMPhos, starting from an inexpensive and air-stable phosphine precursor. Remarkably, this phosphorus heterocycle has been only described now, almost 120 years after its congener, the widely-used TMP. We have successfully demonstrated its use as a synthon by constructing a variety of *tertiary* phosphine ligands as well as several metal complexes incorporating the TMPhos substituent. We believe that TMPhos could find similar applications as the important acyclic di-*tert*-butylphosphine substituent in ligand design and catalysis as it offers a different steric environment, restricted rotation, and different electronic properties to previously known phosphine ligands.

## Methods

### Synthetic procedures

See Supplementary Information Section 1.

### Phosphine oxidation tests

See Supplementary Figs. 1-2

### NMR spectra

See Supplementary Figs. 17–123 in Supplementary Data 1.

### Single crystal X-ray diffraction data

See Supplementary Figs. 3–10 and Supplementary Tables 1–14.

### Buried volume calculations

See Supplementary Figs. 11–15.

### Calculation of Tolman cone angles

See Supplementary Fig. 16 and Supplementary Tables 15–16.

## Supplementary information

Description of Additional Supplementary Files

Supplementary Data 1

Supplementary Data 2

Supplementary Data 3

Supplementary Data 4

Supplementary Data 5

Supplementary Data 6

Supplementary Data 7

Supplementary Data 8

Supplementary Information

## Data Availability

The authors declare that the data supporting the findings of this study are available within the article and Supplementary Information. For experimental details and compound characterisation data, see Supplementary Information. For NMR spectra, see Supplementary Data 1. The X-ray crystallographic data for compounds can be found in Supplementary Data 2–8 or obtained free of charge from The Cambridge Crystallographic Data Centre with the accession codes CDCC #2182394 (**2**), #2182395 (**5**), #2182396 (**15**), #2182397 (**20**), #2182398 (**23**), #2182399 (**24**) and #2182393 (**S3**) via www.ccdc.cam.ac.uk/data_request/cif.
