# Supplementary material for: Tetramethylphosphinane as a new secondary phosphine synthon
Source: Commun Chem. 2023 Apr 29;6:85. doi: 10.1038/s42004-023-00876-8 (PMC10148838; doi:10.1038/s42004-023-00876-8)
Supplement: Supplementary file 1 — Description of Additional Supplementary Files [file 42004_2023_876_MOESM1_ESM.pdf]

Description of Additional Supplementary Files

File name: Supplementary Data 1

Description: 1 NMR Spectra

File name: Supplementary Data 2

Description: cif file for compound 2 (CDCC #2182394)

File name: Supplementary Data 3

Description: cif file for compound 5 (CCDC #2182395)

File name: Supplementary Data 4

Description: cif file for compound 15 (#2182396)

File name: Supplementary Data 5

Description: cif file for compound 20 (CCDC #2182397)

File name: Supplementary Data 6

Description: cif file for compound 23 (CCDC #2182398)

File name: Supplementary Data 7

Description: cif file for compound 24 (CCDC #2182399)

File name: Supplementary Data 8

Description: cif file for compound S3 (CCDC #2182393)
