# Supplementary material for: Tetramethylphosphinane as a new secondary phosphine synthon
Source: Commun Chem. 2023 Apr 29;6:85. doi: 10.1038/s42004-023-00876-8 (PMC10148838; doi:10.1038/s42004-023-00876-8)
Supplement: Supplementary file 2 — Supplementary Data 1 [file 42004_2023_876_MOESM2_ESM.pdf]

Supplementary Data 1 (NMR Spectra)

Tetramethylphosphinane as a new secondary phosphine synthon

James D. Nobbs, Sigit Sugiarto, Xin Yi See, Choon Boon Cheong, Srinivasulu Aitipamula, Ludger P. Stubbs and Martin van Meurs*

1. NMR Spectra

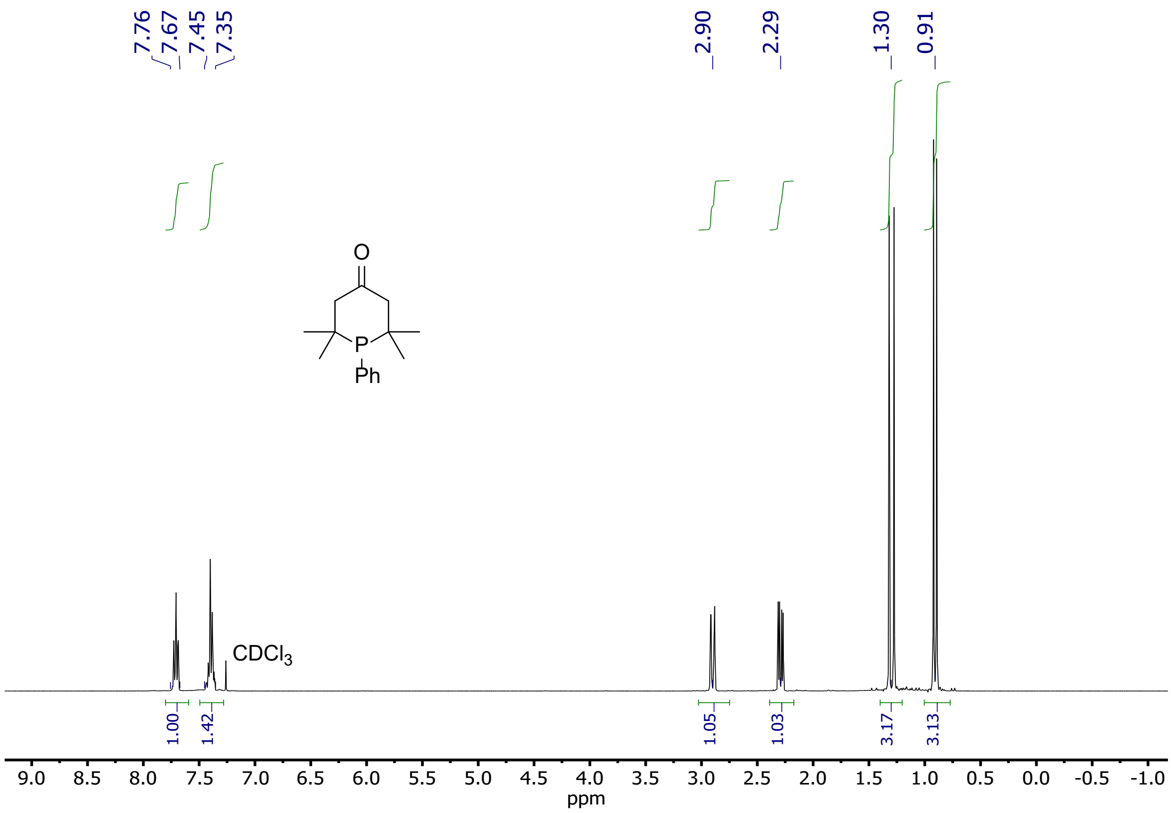

Supplementary Figure 17. ^1H NMR (400 MHz, $CDCl_3$) spectrum of 2,2,6,6-tetramethyl-1-phenylphosphinan-4-one, compound **(i)**.

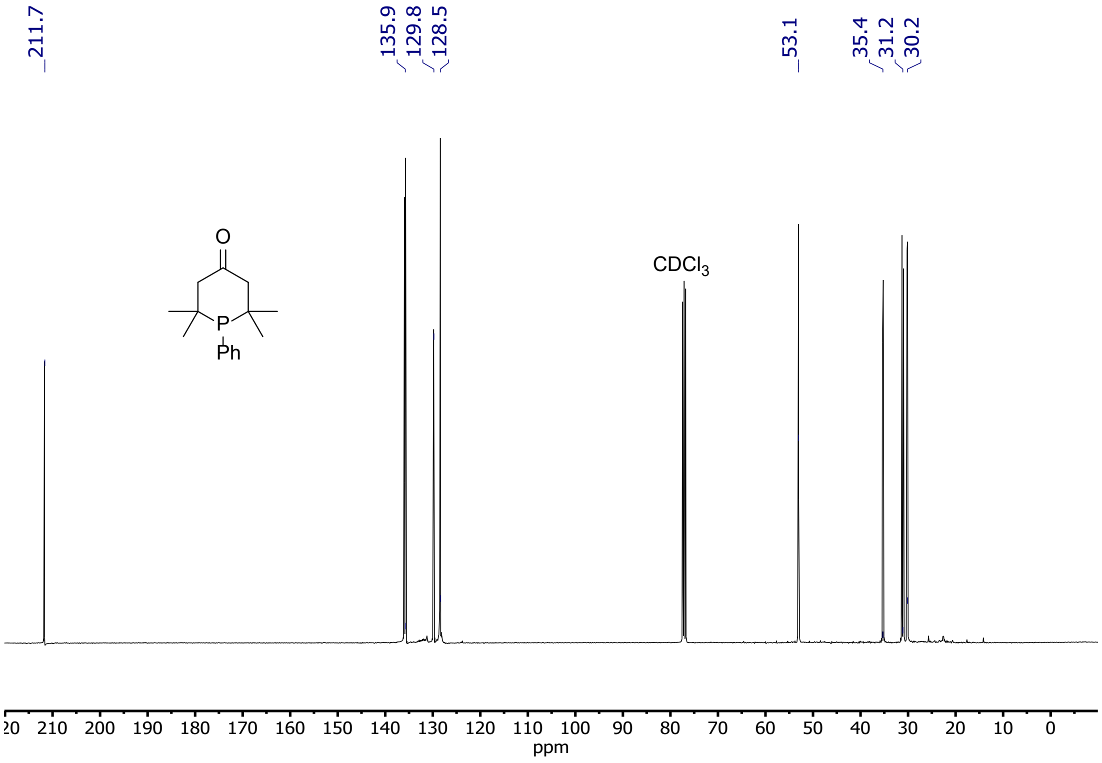

Supplementary Figure 18. $^{13}C\{^1H\}$ NMR (101 MHz, $CDCl_3$) spectrum of 2,2,6,6-tetramethyl-1-phenylphosphinan-4-one, compound **(i)**.

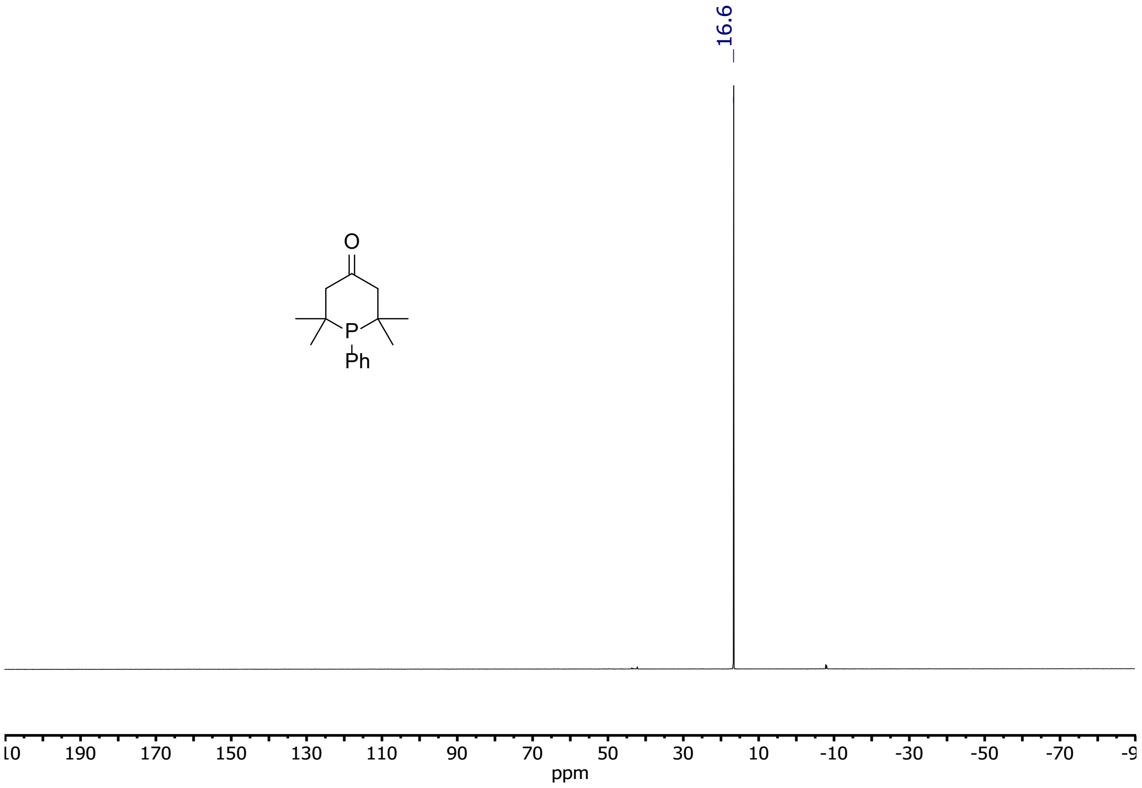

Supplementary Figure 19. ^{31}P NMR (162 MHz, $CDCl_3$) spectrum of 2,2,6,6-tetramethyl-1-phenylphosphinan-4-one, compound **(i)**.

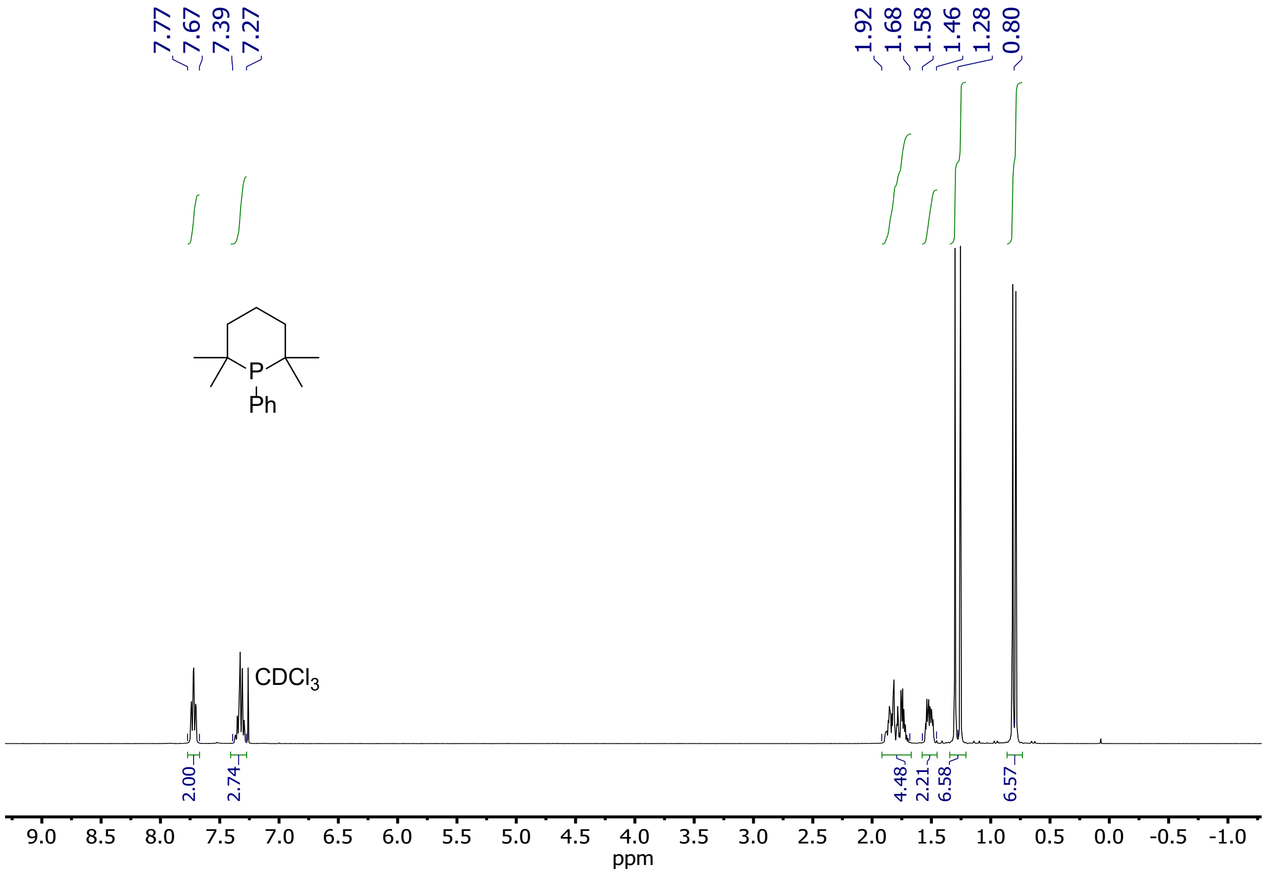

Supplementary Figure 20. ^{1}H NMR (400 MHz, $CDCl_3$) spectrum of 2,2,6,6-tetramethyl-1-phenylphosphinane, compound **(ii)**.

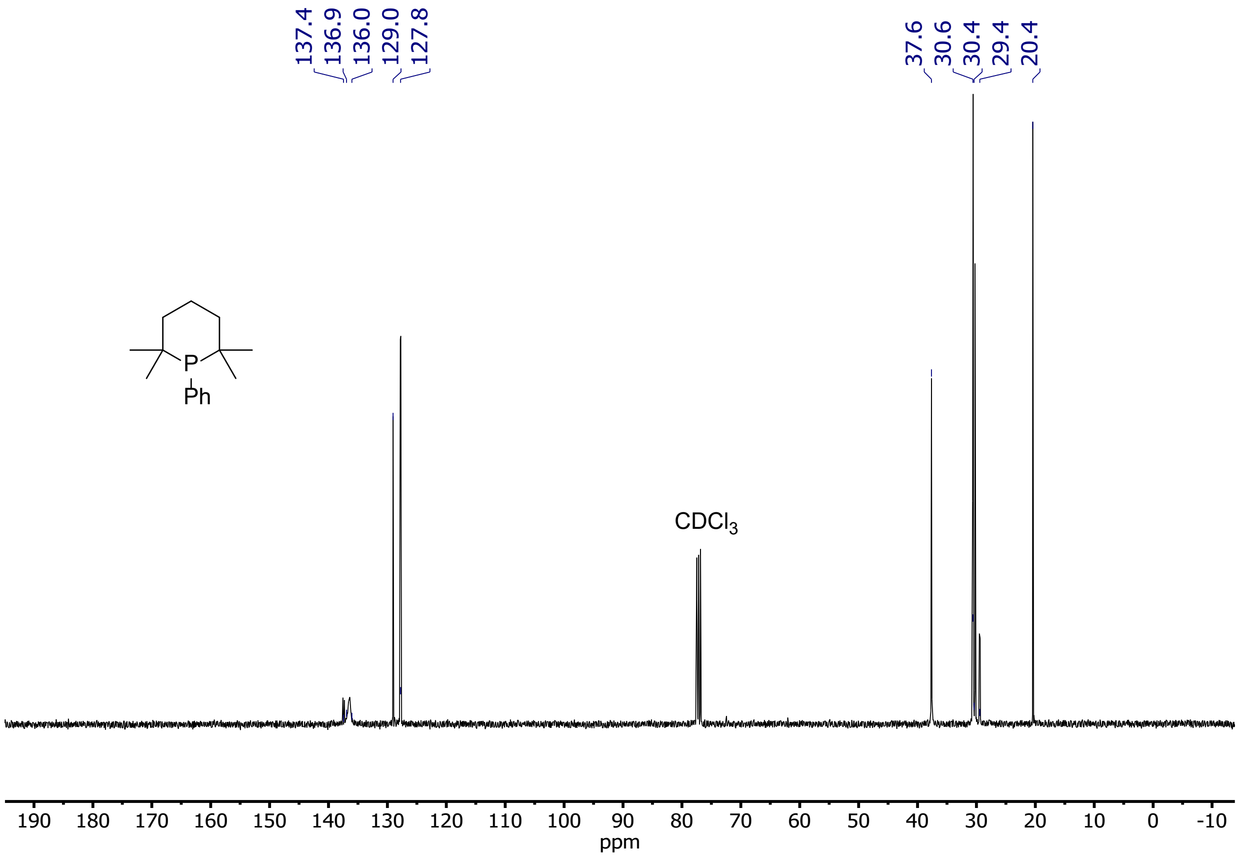

Supplementary Figure 21. $^{13}C\{^{1}H\}$ NMR (101 MHz, $CDCl_3$) spectrum of 2,2,6,6-tetramethyl-1-phenylphosphinane, compound **(ii)**.

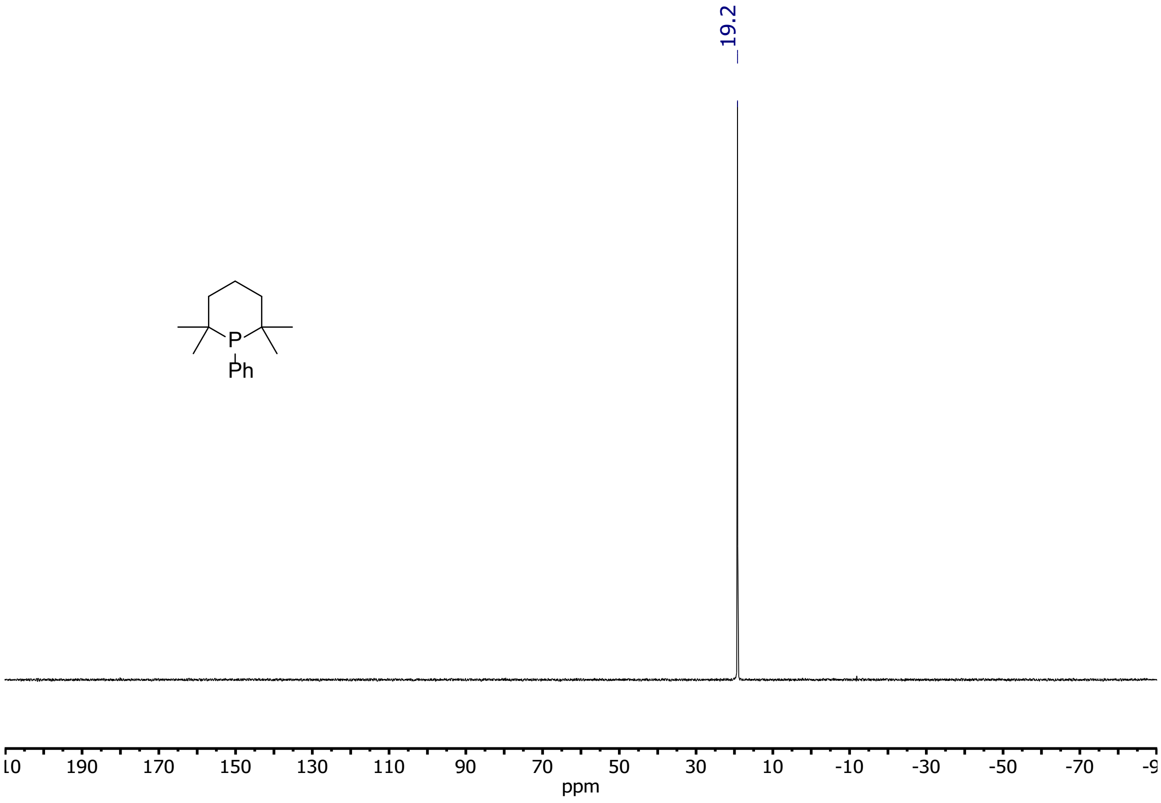

Supplementary Figure 22. ^{31}P NMR (162 MHz, $CDCl_3$) spectrum of 2,2,6,6-tetramethyl-1-phenylphosphinane, compound **(ii)**.

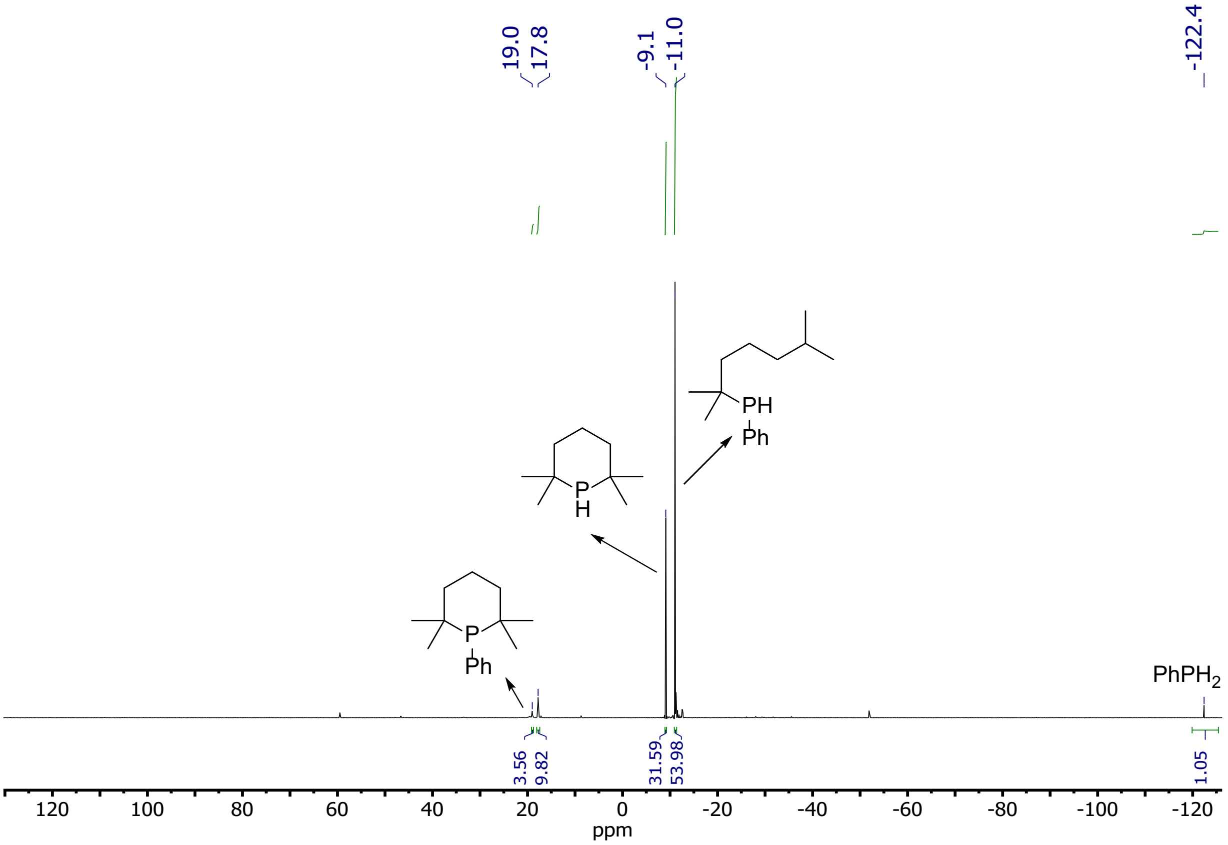

Supplementary Figure 23. ^{31}P NMR (162 MHz, $CDCl_3$) spectrum of the crude mixture obtained from the Li cleavage of compound **ii** to give compound **(iii)** (**TMPhos**) and by-products.

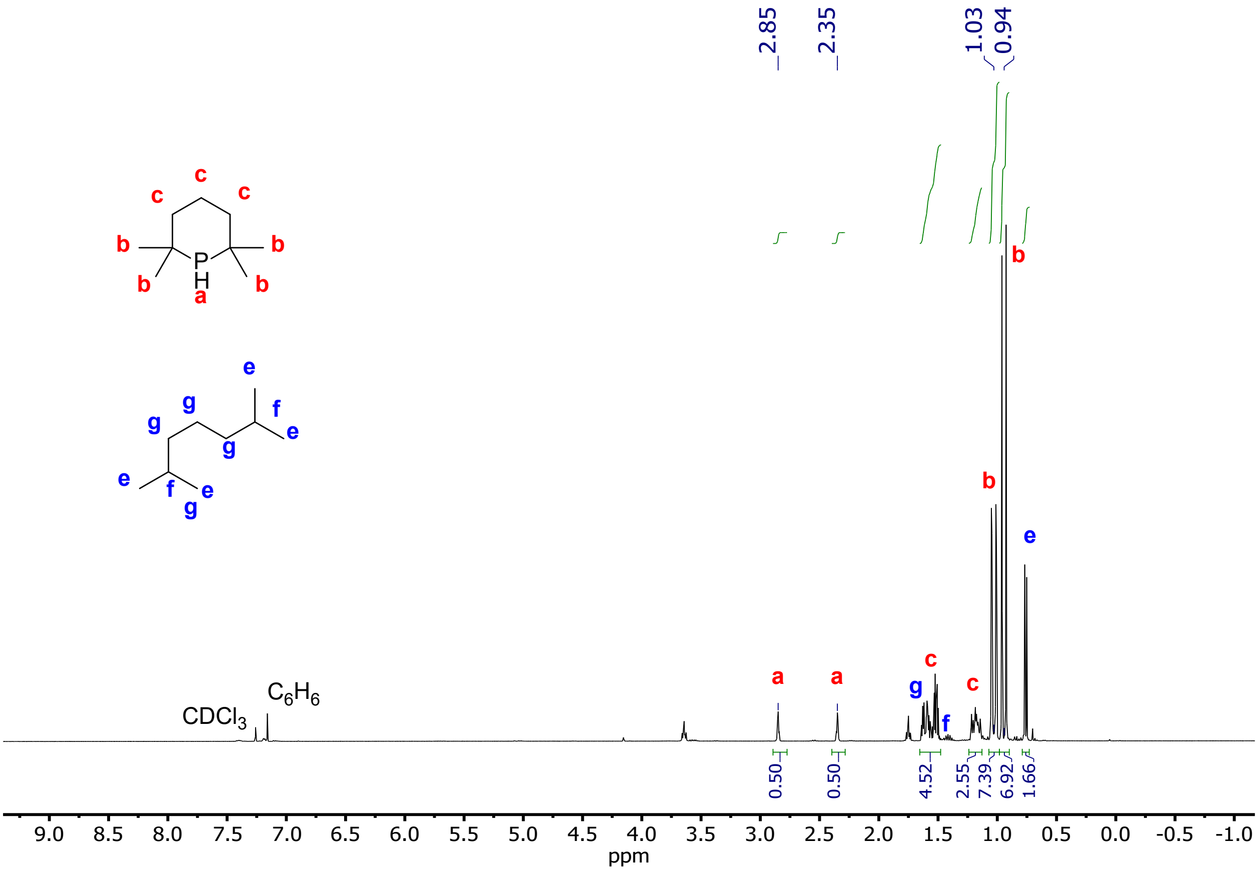

Supplementary Figure 24. ^{1}H NMR (400 MHz, $CDCl_3$) spectrum of 2,2,6,6-tetramethylphosphinane, **TMPhos**, compound **(iii)**, containing residual by-product 2,6-dimethylheptane.

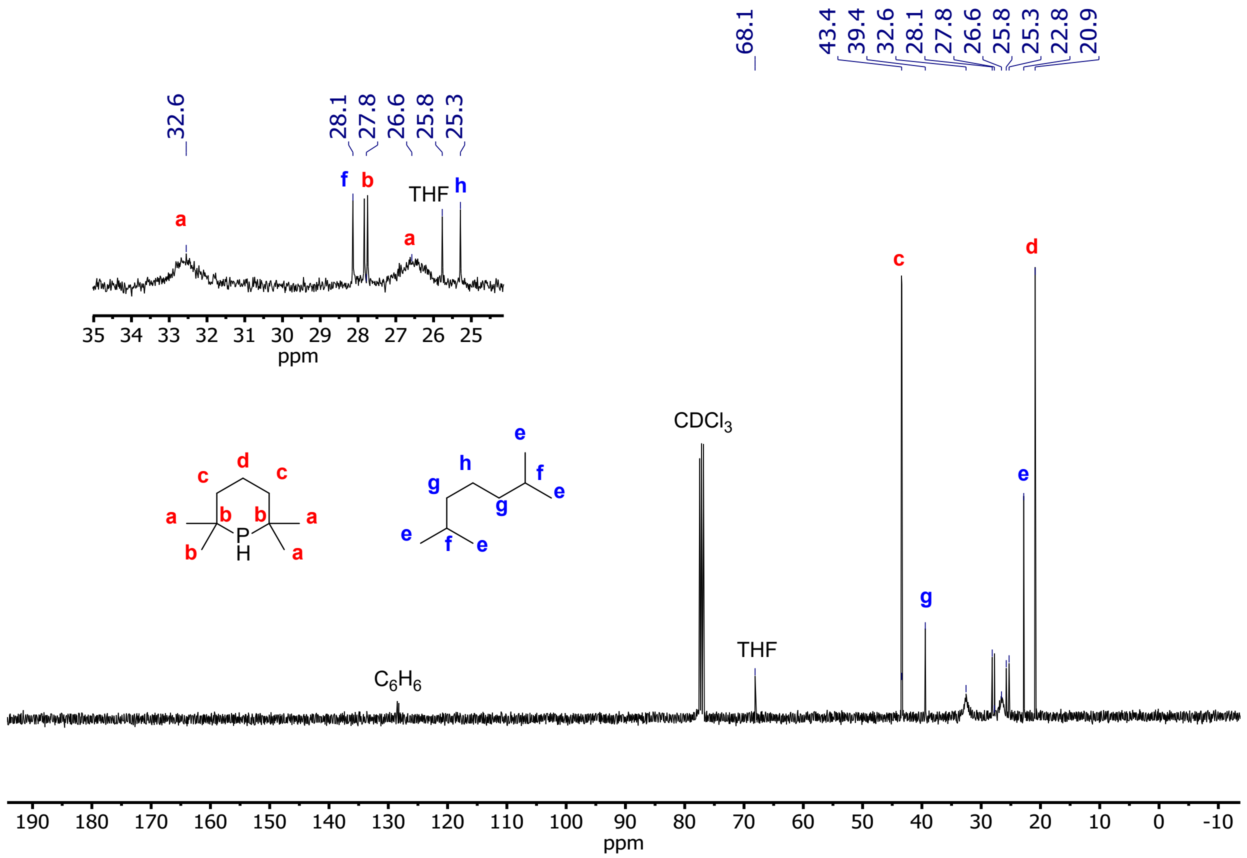

Supplementary Figure 25. $^{13}C\{^{1}H\}$ NMR (101 MHz, $CDCl_3$) spectrum of 2,2,6,6-tetramethylphosphinane, **TMPhos**, compound **(iii)**, containing residual by-product 2,6-dimethylheptane.

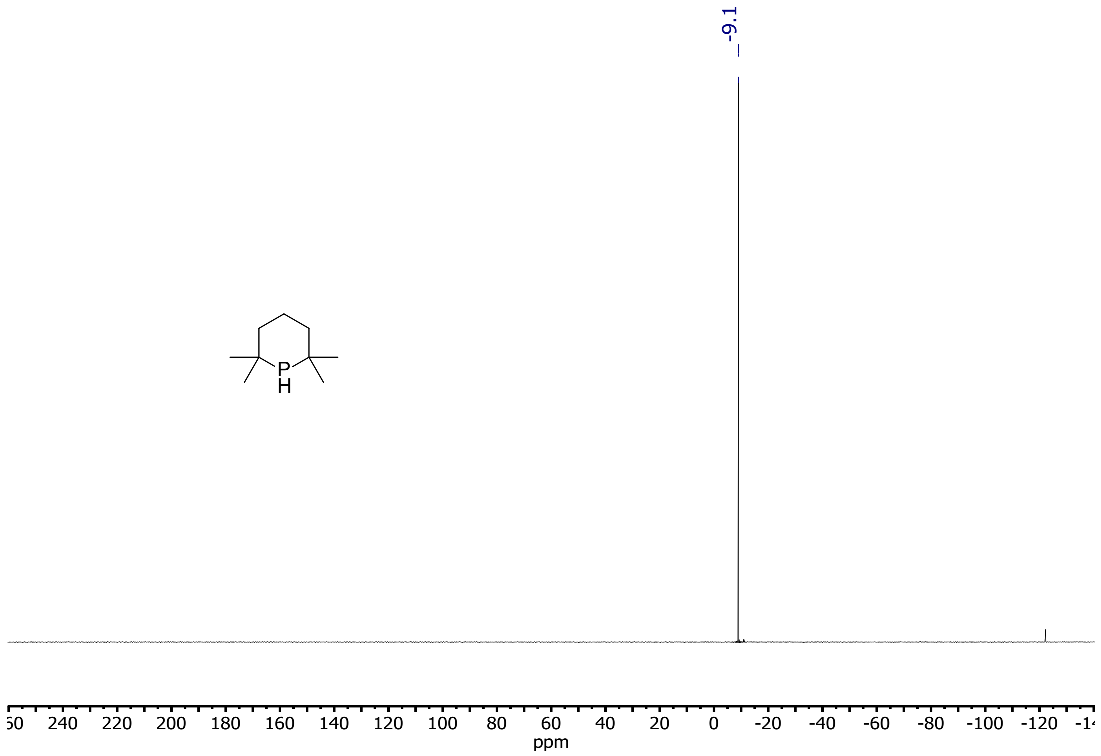

Supplementary Figure 26. ^{31}P NMR (162 MHz, $CDCl_3$) spectrum of 2,2,6,6-tetramethylphosphinane, **TMPhos**, compound **(iii)**, containing residual by-product 2,6-dimethylheptane.

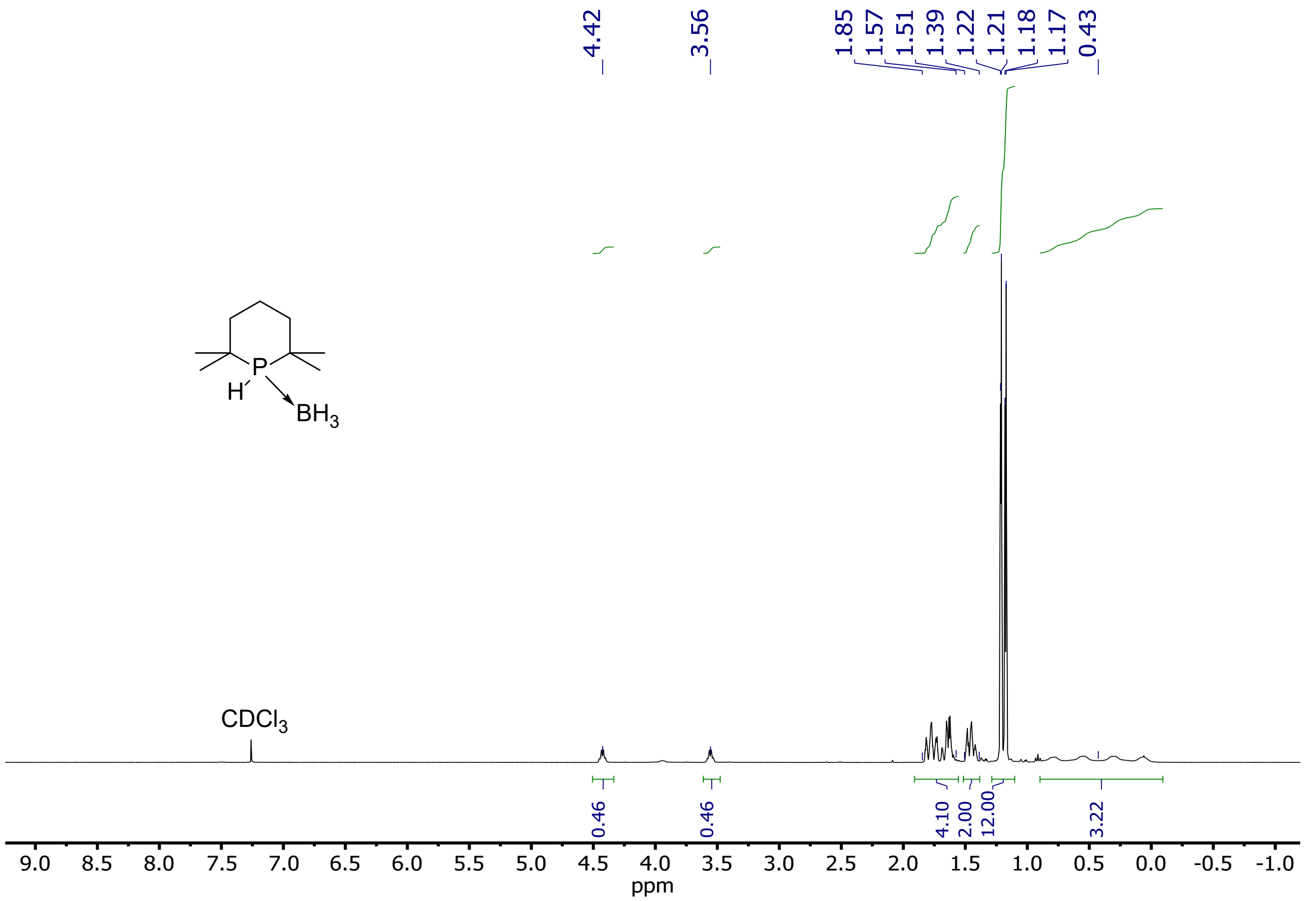

Supplementary Figure 27. ^{1}H NMR (400 MHz, $CDCl_3$) spectrum of 2,2,6,6-tetramethylphosphinane borane complex, compound **(iv)**, **TMPhos·BH₃**.

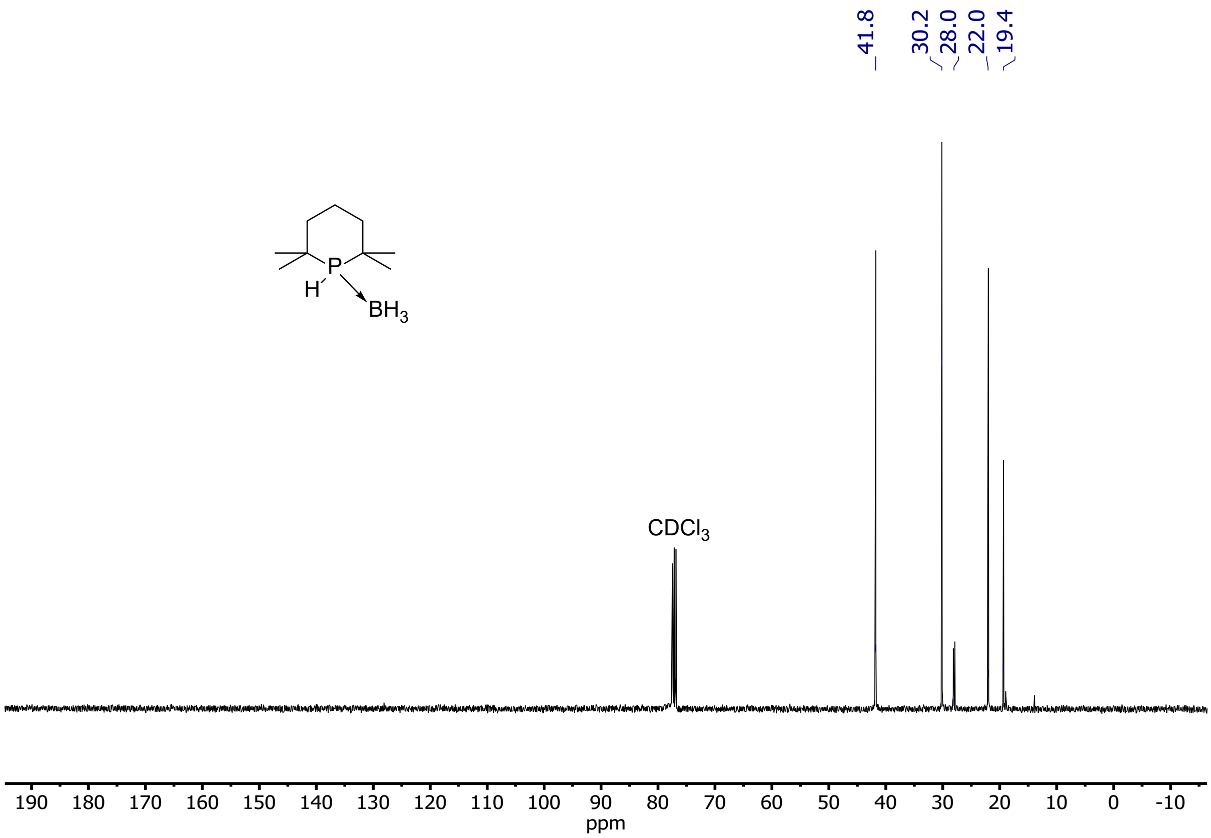

Supplementary Figure 28. $^{13}C\{^{1}H\}$ NMR (101 MHz, $CDCl_3$) spectrum of 2,2,6,6-tetramethylphosphinane borane complex, compound **(iv)**, **TMPhos·BH₃**.

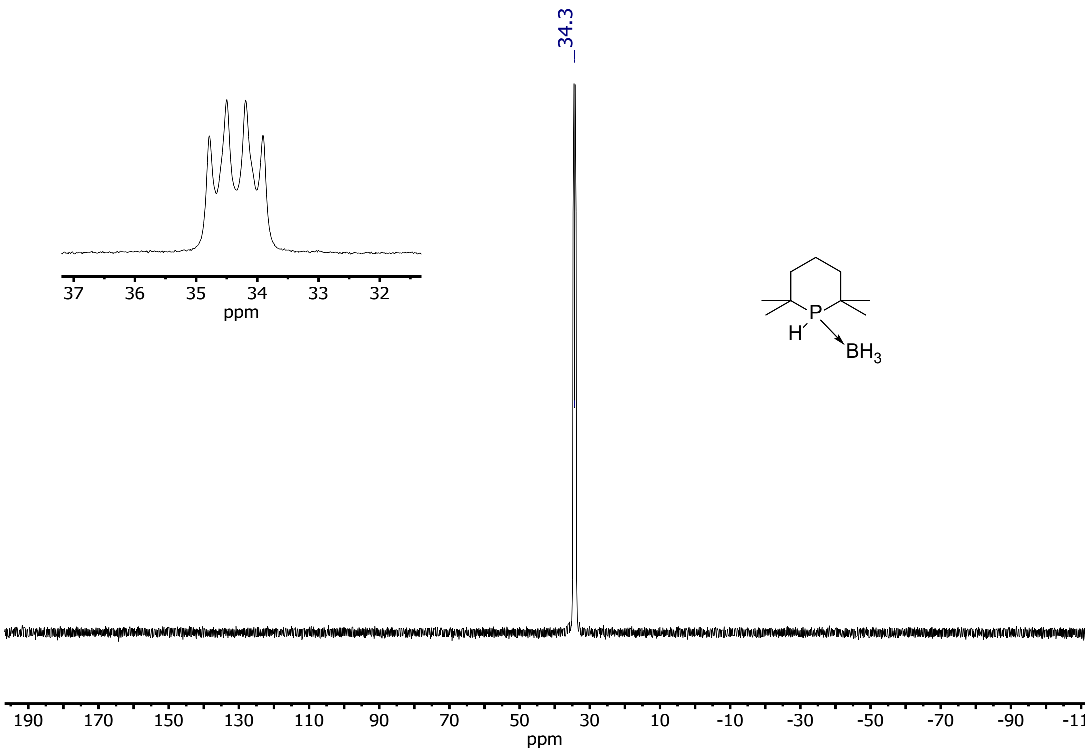

Supplementary Figure 29. ^{31}P NMR {^{1}H} (162 MHz, $CDCl_3$) spectrum of 2,2,6,6-tetramethylphosphinane borane complex, compound **(iv)**, **TMPhos·BH$_3$**.

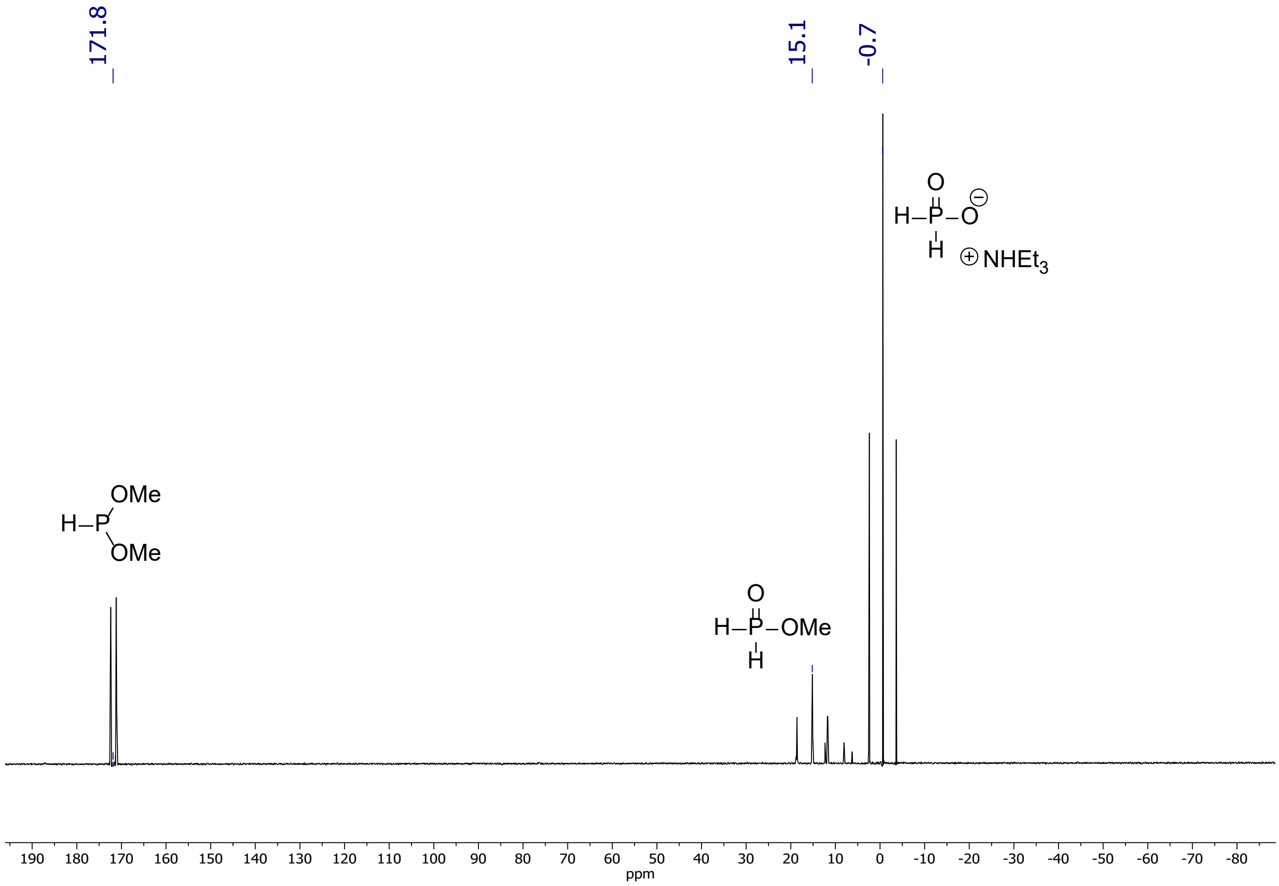

Supplementary Figure 30. ^{31}P NMR {^{1}H} (162 MHz, THF/d_8-tol) spectrum of the reaction between methyl hypophosphite, H_2PO_2Me and phorone in the presence of NEt_3.

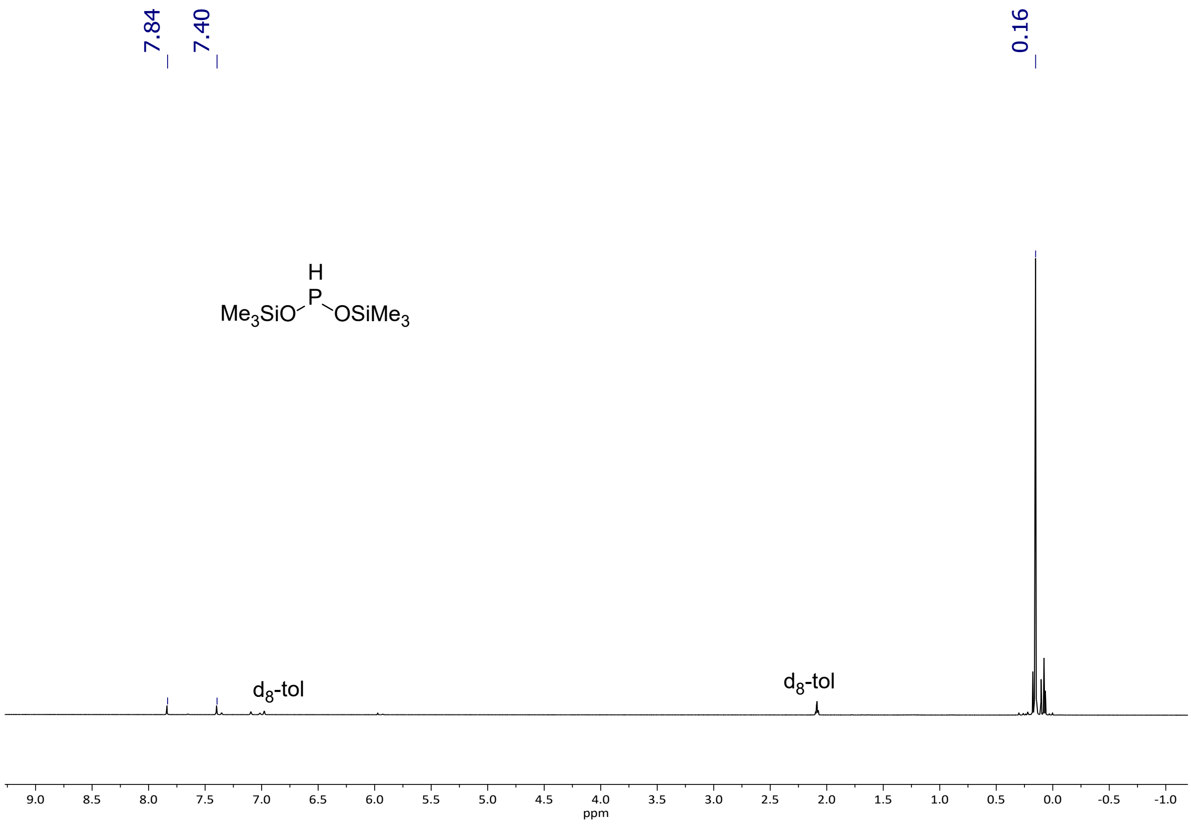

Supplementary Figure 31. ^{1}H NMR (400 MHz, d_8-toluene) spectrum of bis(trimethylsilyl)phosphonite.

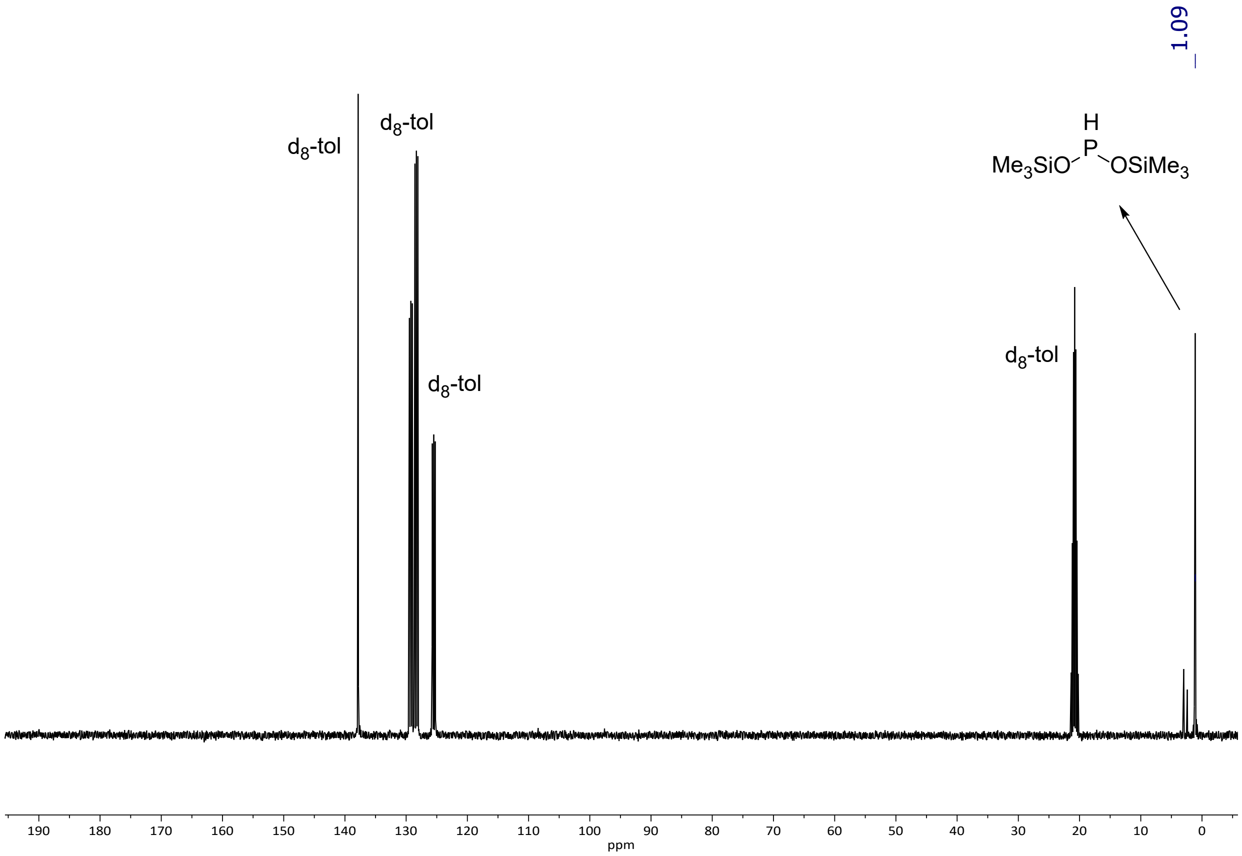

Supplementary Figure 32. $^{13}C\{^{1}H\}$ NMR (101 MHz, d_8-toluene) spectrum of bis(trimethylsilyl)phosphonite.

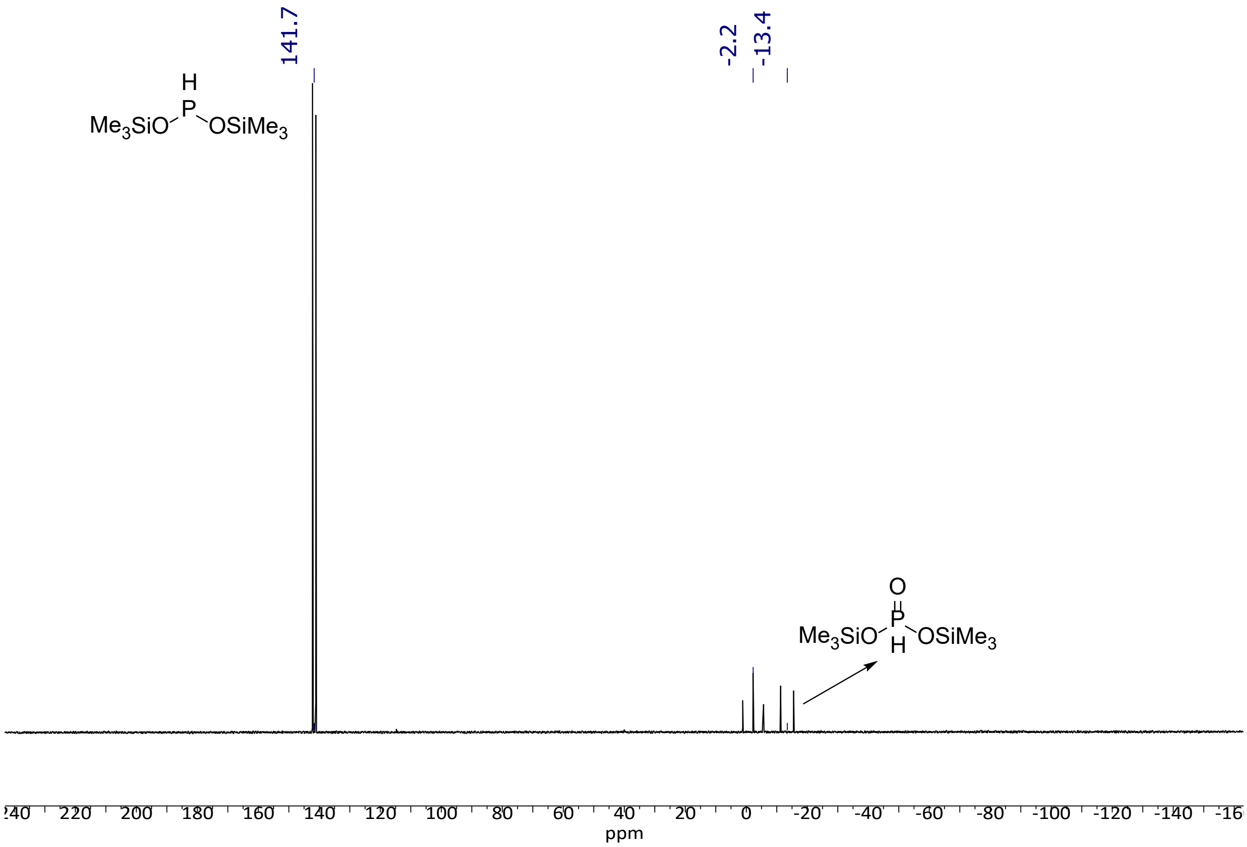

Supplementary Figure 33. ^{31}P NMR (162 MHz, d_8-toluene) spectrum of bis(trimethylsilyl)phosphonite.

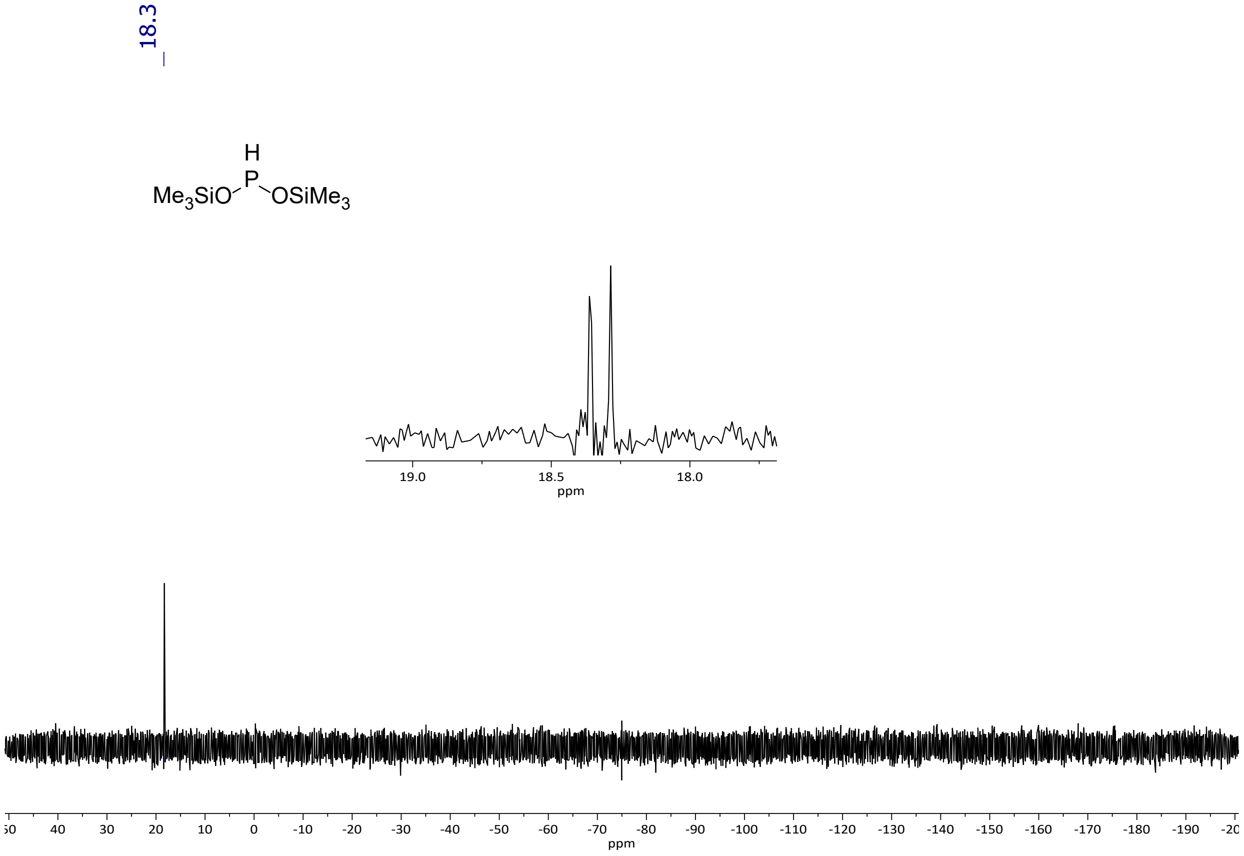

Supplementary Figure 34. $^{29}Si\{^1H\}$ NMR (60 MHz, d_8-toluene) spectrum of bis(trimethylsilyl)phosphonite.

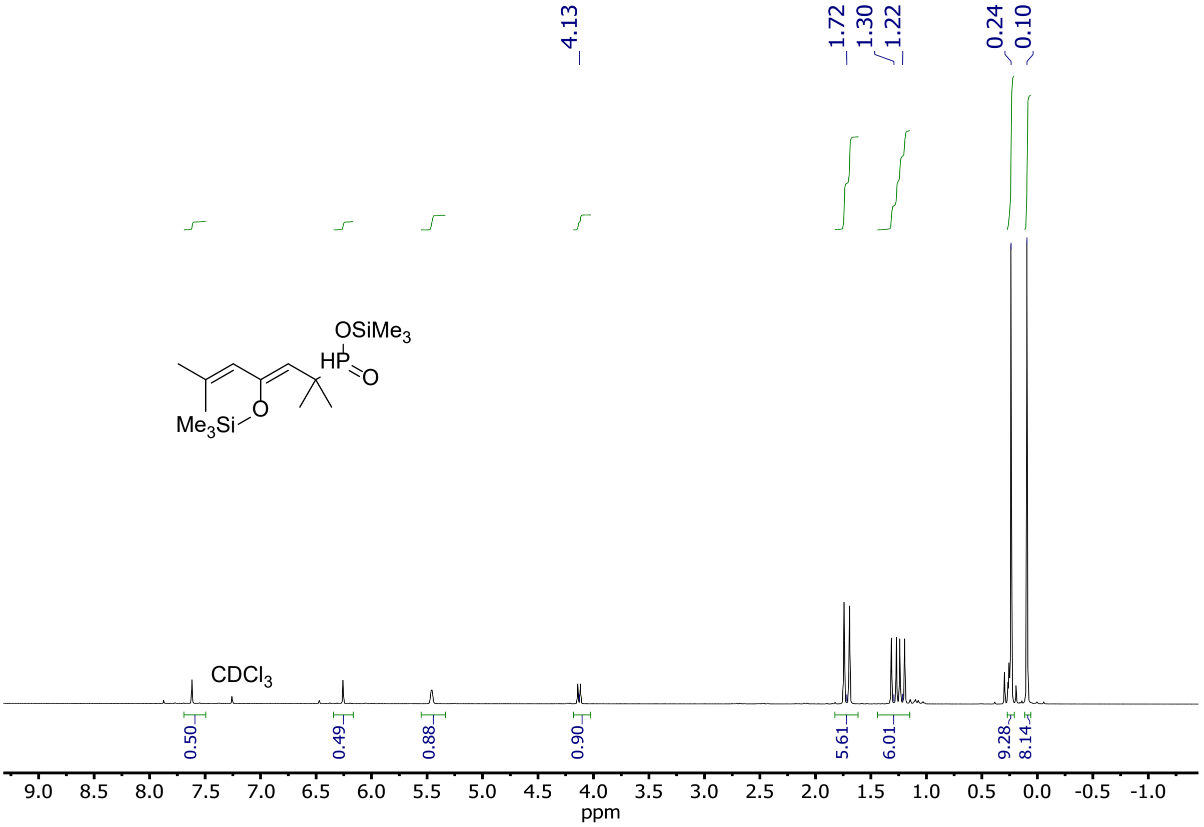

Supplementary Figure 35. ^{1}H NMR (400 MHz, $CDCl_3$) spectrum of trimethylsilyl (Z)-(2,6-dimethyl-4-((trimethylsilyl)oxy)hepta-3,5-dien-2-yl)phosphinate, compound **1**.

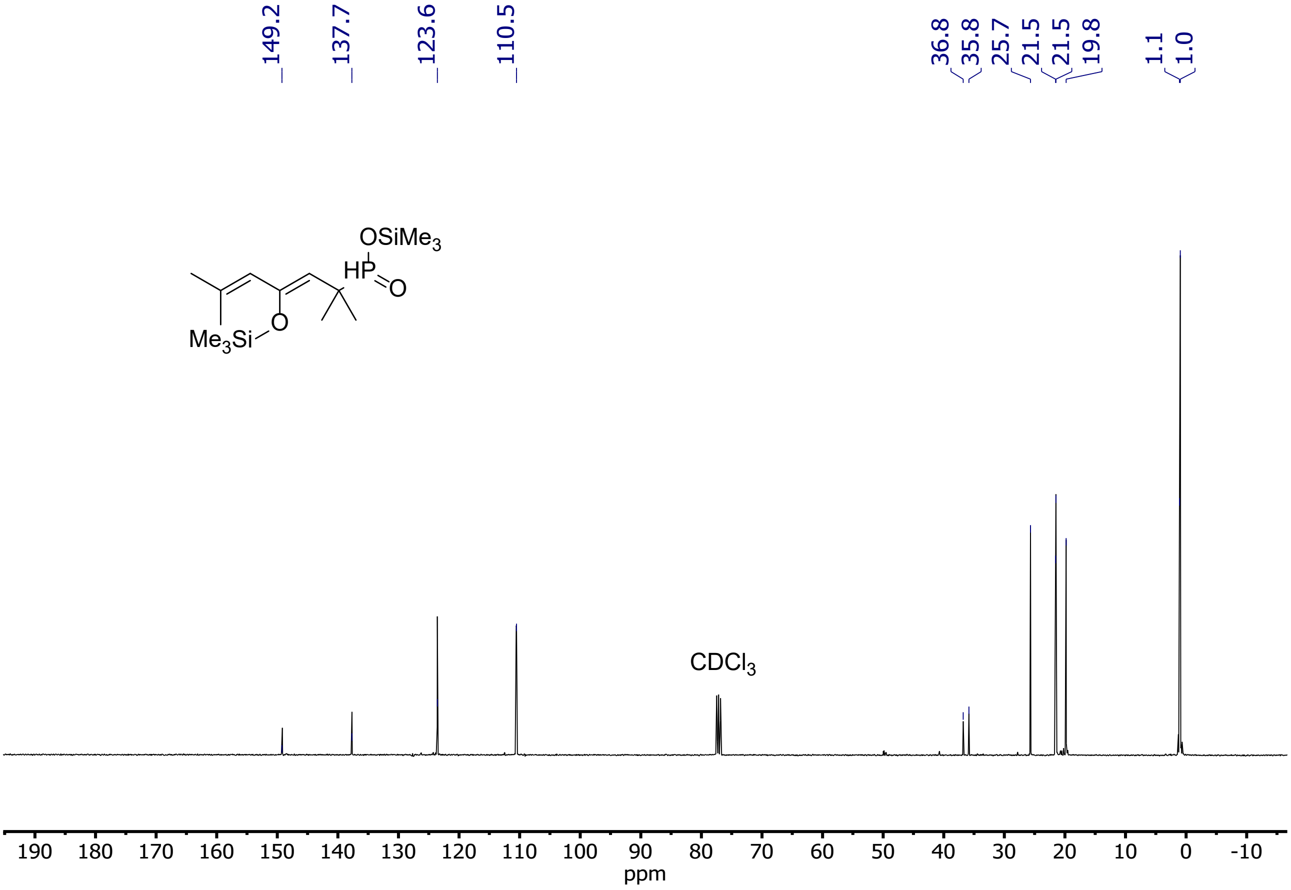

Supplementary Figure 36. $^{13}C\{^{1}H\}$ NMR (101 MHz, $CDCl_3$) spectrum of trimethylsilyl (Z)-(2,6-dimethyl-4-((trimethylsilyl)oxy)hepta-3,5-dien-2-yl)phosphinate, compound **1**.

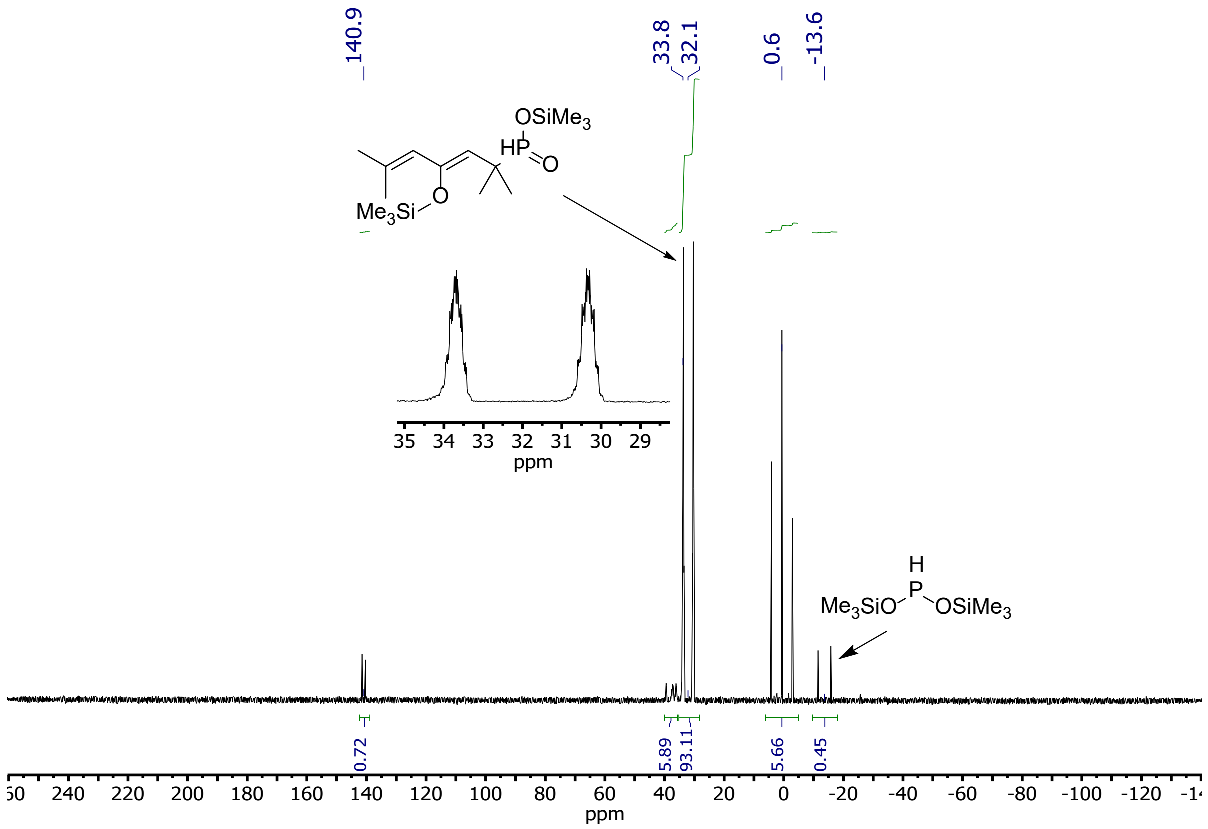

Supplementary Figure 37. ^{31}P NMR (162 MHz, $CDCl_3$) spectrum of trimethylsilyl (Z)-(2,6-dimethyl-4-((trimethylsilyl)oxy)hepta-3,5-dien-2-yl)phosphinate, compound **1**.

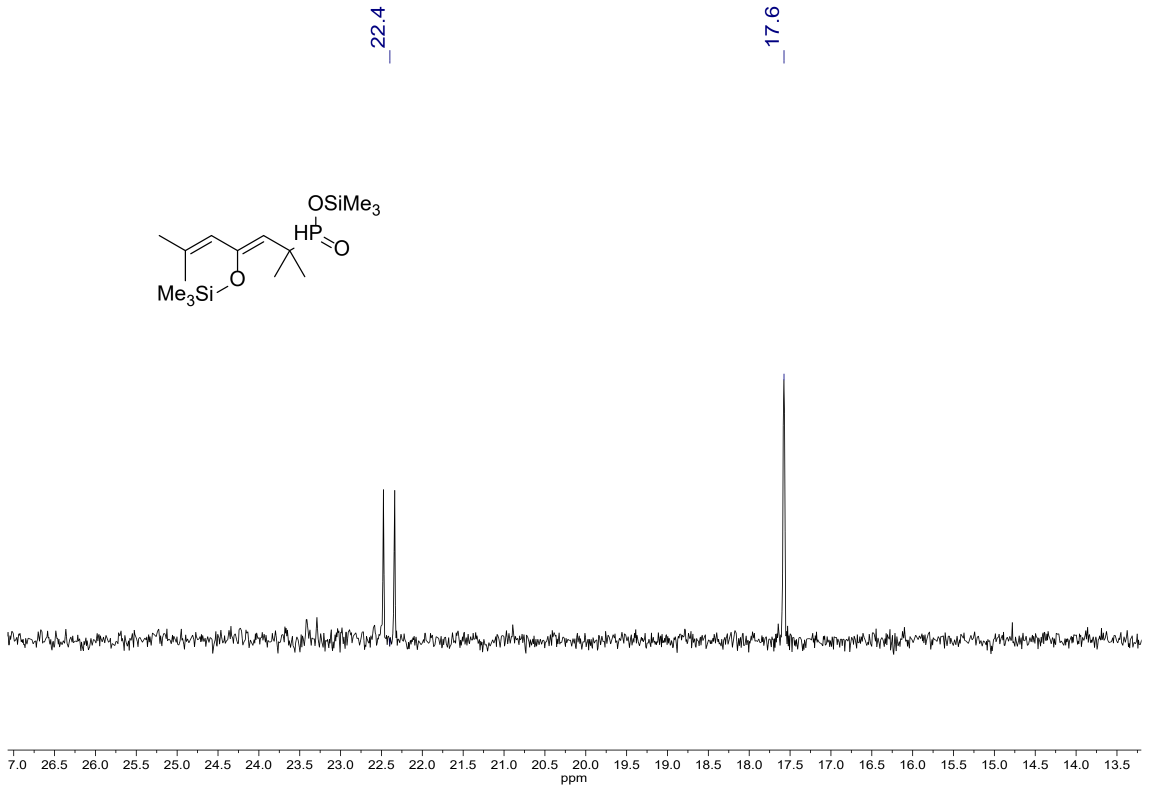

Supplementary Figure 38. ^{29}Si{^{1}H} NMR (60 MHz, $CDCl_3$) spectrum of trimethylsilyl (Z)-(2,6-dimethyl-4-((trimethylsilyl)oxy)hepta-3,5-dien-2-yl)phosphinate, compound **1**.

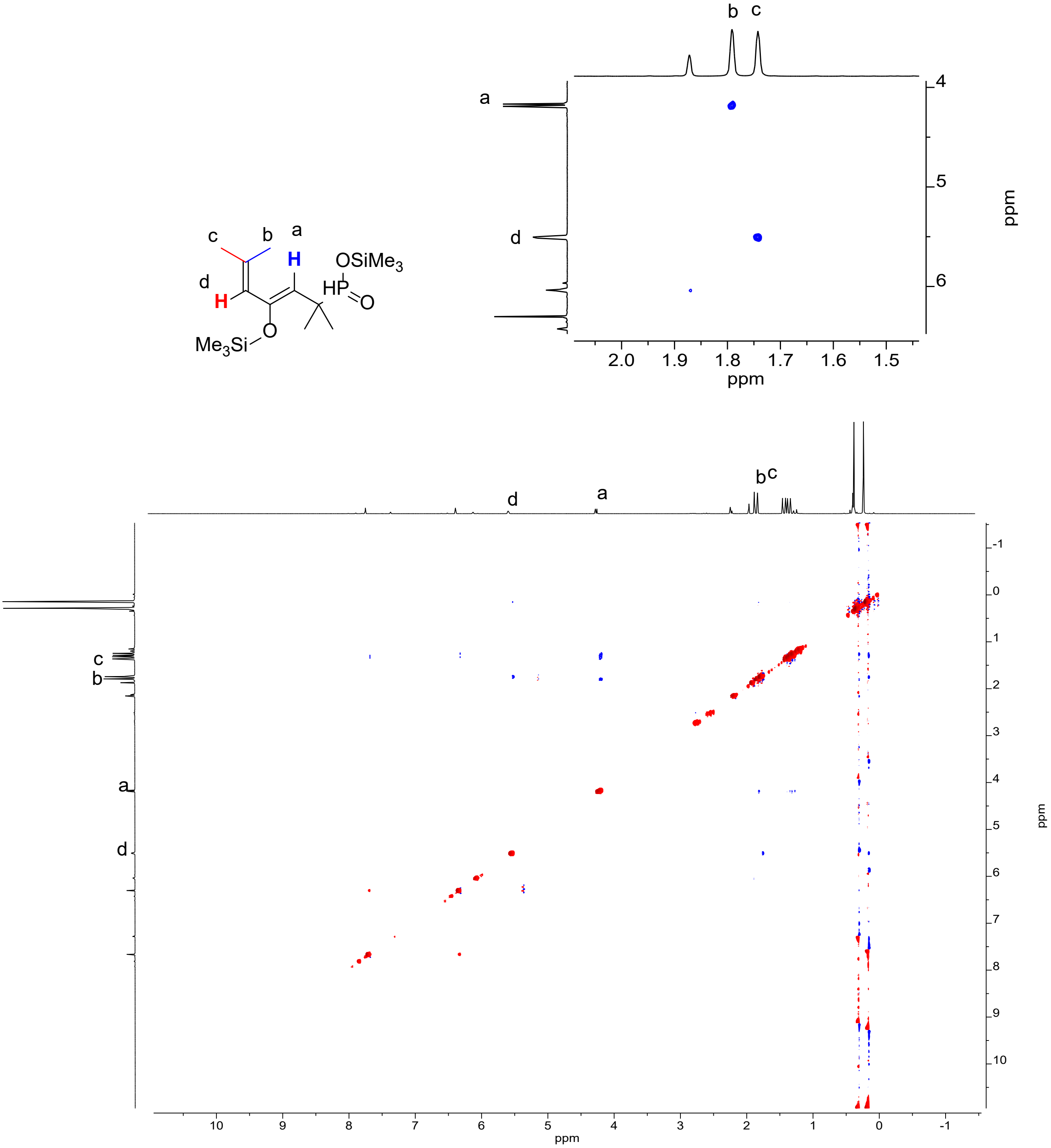

Supplementary Figure 39. 1H-1H NOESY NMR (400 MHz, CDCl3) spectrum of trimethylsilyl (Z)-(2,6-dimethyl-4-((trimethylsilyl)oxy)hepta-3,5-dien-2-yl)phosphinate, compound **1**.

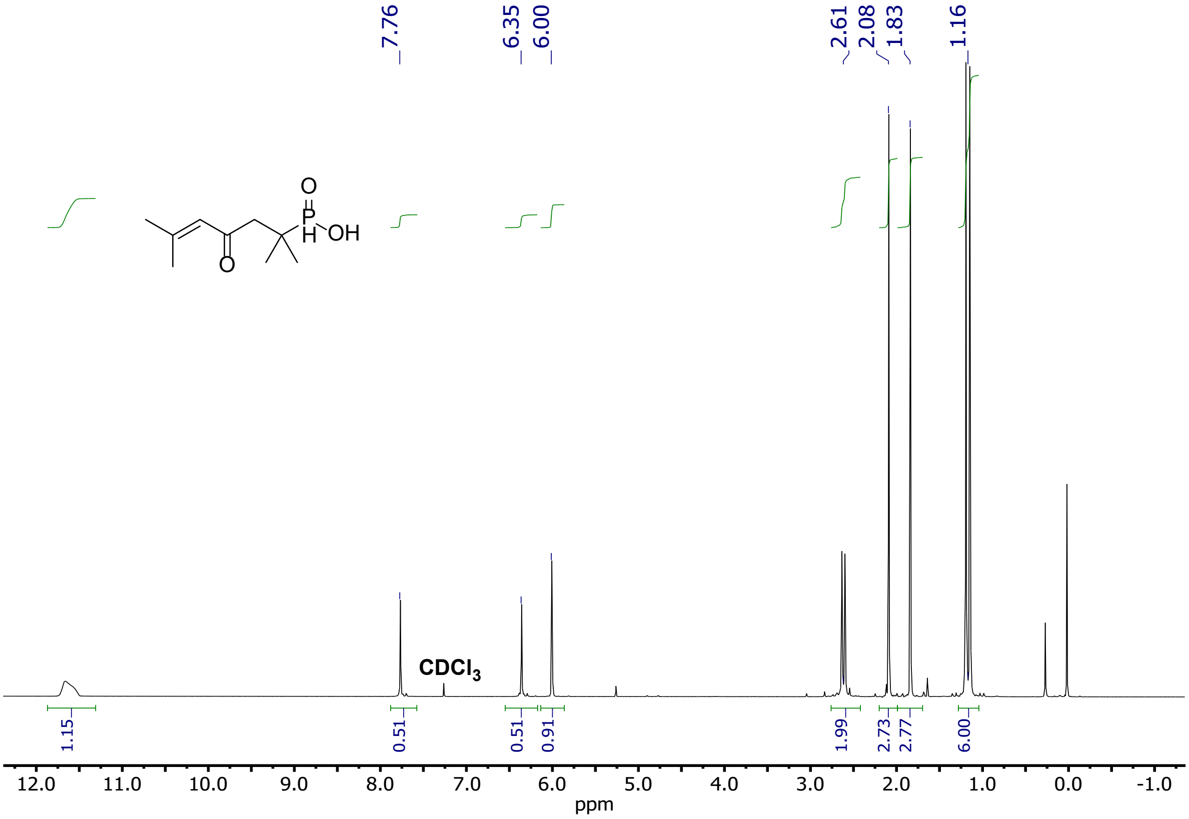

Supplementary Figure 40. ^{1}H NMR (400 MHz, $CDCl_3$) spectrum of (2,6-dimethyl-4-oxohept-5-en-2-yl)phosphinic acid, compound **2**.

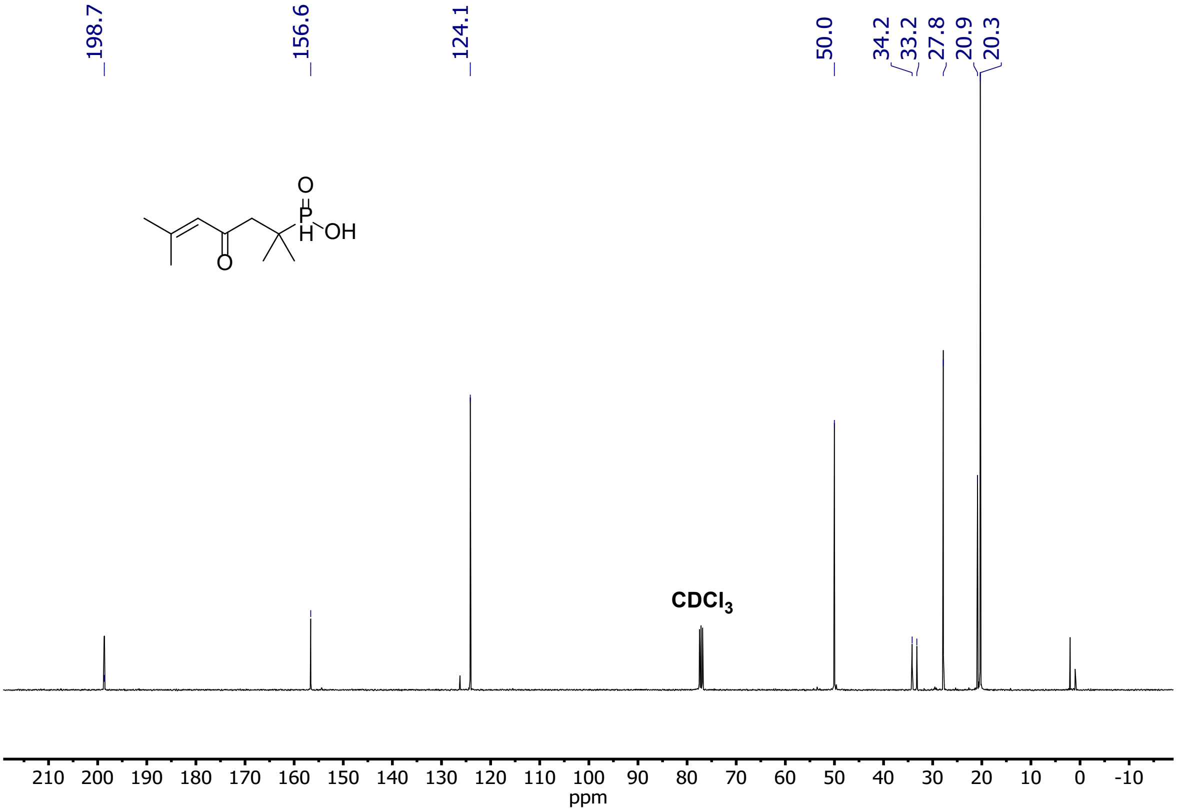

Supplementary Figure 41. $^{13}C\{^{1}H\}$ NMR (101 MHz, $CDCl_3$) spectrum of (2,6-dimethyl-4-oxohept-5-en-2-yl)phosphinic acid, compound **2**.

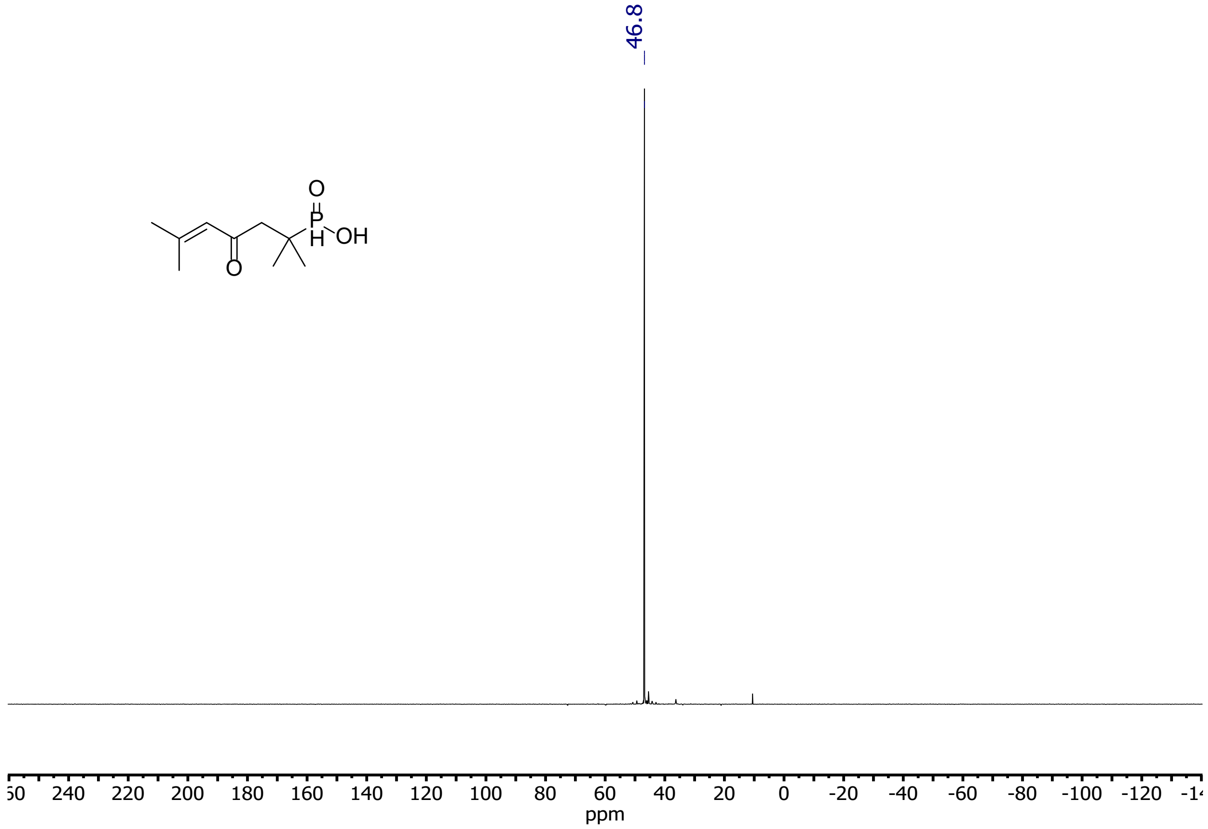

Supplementary Figure 42. $^{31}P\{^{1}H\}$ NMR (162 MHz, $CDCl_3$) spectrum of (2,6-dimethyl-4-oxohept-5-en-2-yl)phosphinic acid, compound **2**.

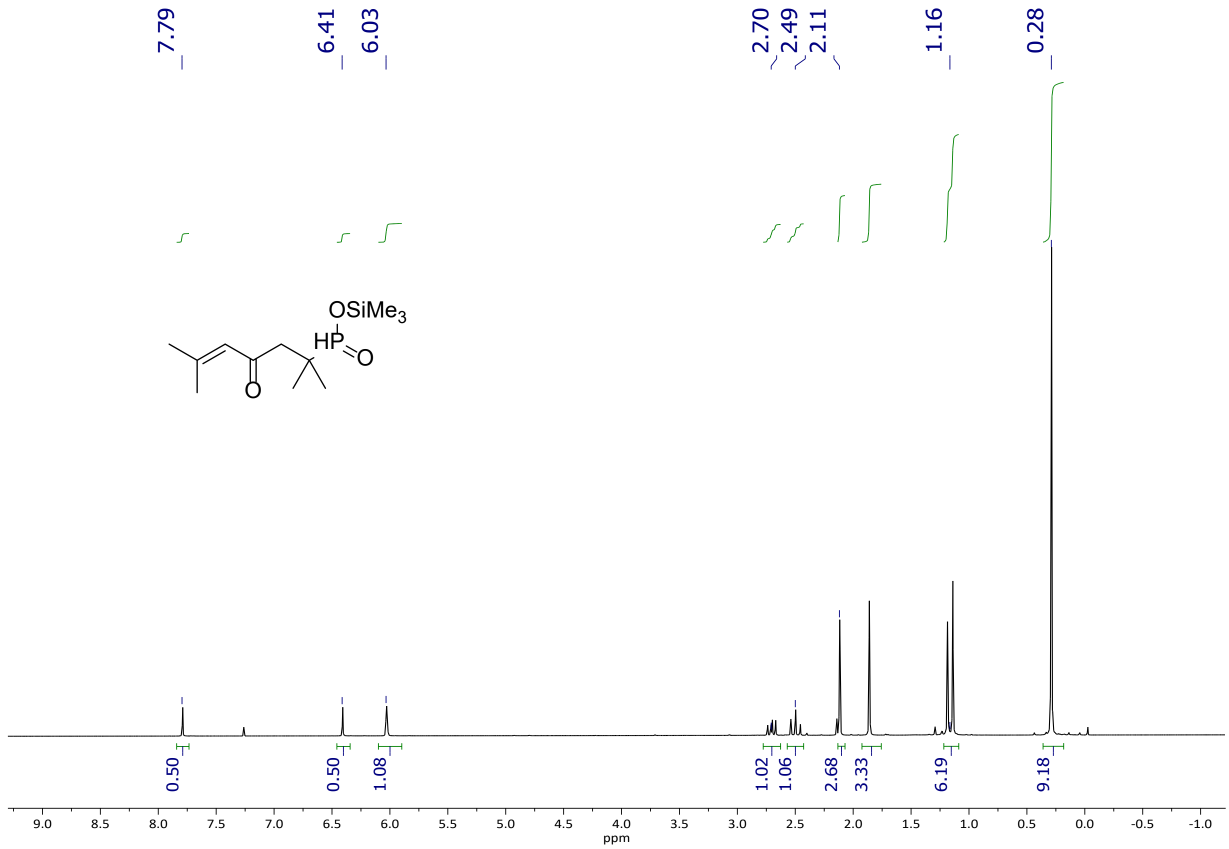

Supplementary Figure 43. ^{1}H NMR (400 MHz, $CDCl_3$) spectrum of trimethylsilyl (2,6-dimethyl-4-oxohept-5-en-2-yl)phosphinate, compound **3**.

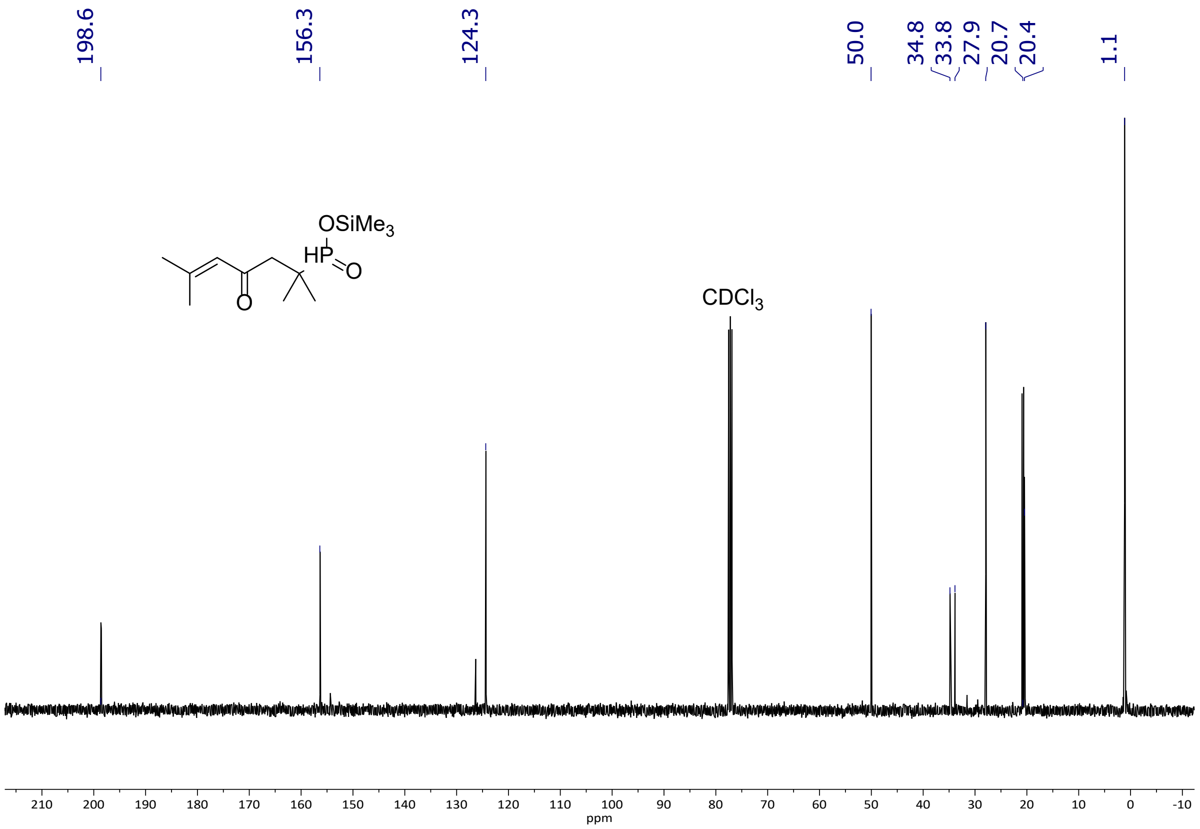

Supplementary Figure 44. $^{13}C\{^{1}H\}$ NMR (101 MHz, $CDCl_3$) spectrum of trimethylsilyl (2,6-dimethyl-4-oxohept-5-en-2-yl)phosphinate, compound **3**.

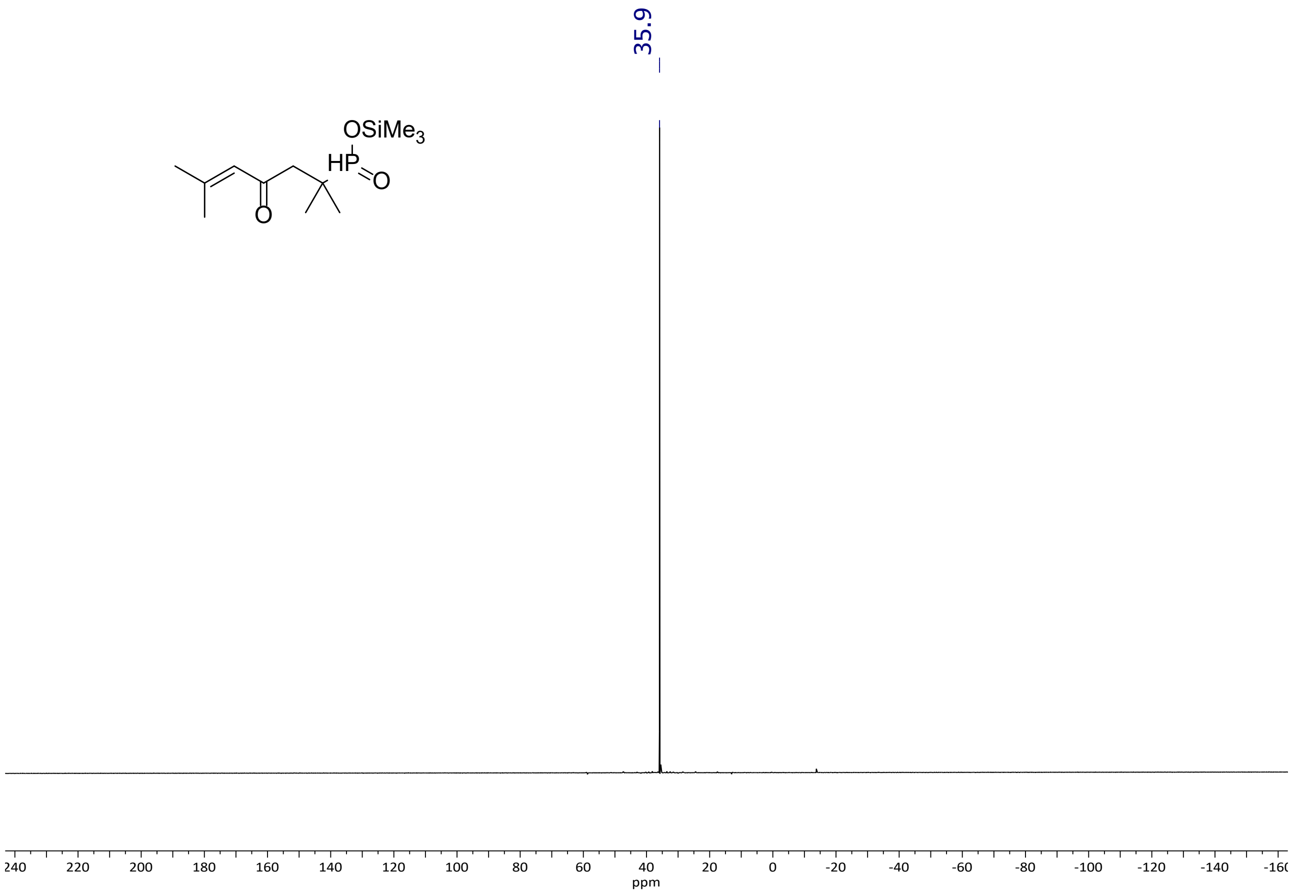

Supplementary Figure 45. $^{31}P\{^{1}H\}$ NMR (400 MHz, $CDCl_3$) spectrum of trimethylsilyl (2,6-dimethyl-4-oxohept-5-en-2-yl)phosphinate, compound **3**.

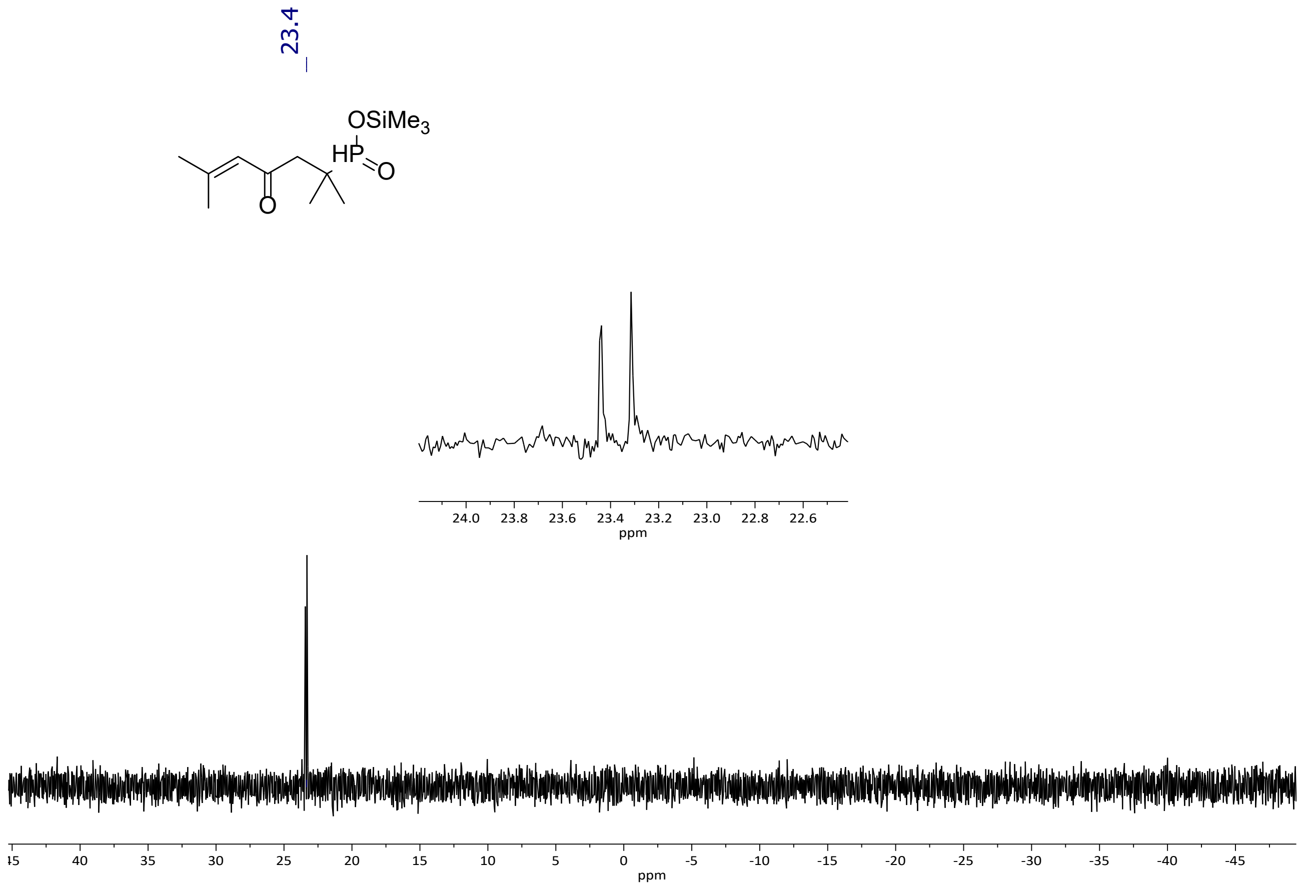

Supplementary Figure 46. $^{29}Si\{^{1}H\}$ NMR (60 MHz, $CDCl_3$) spectrum of trimethylsilyl (2,6-dimethyl-4-oxohept-5-en-2-yl)phosphinate, compound **3**.

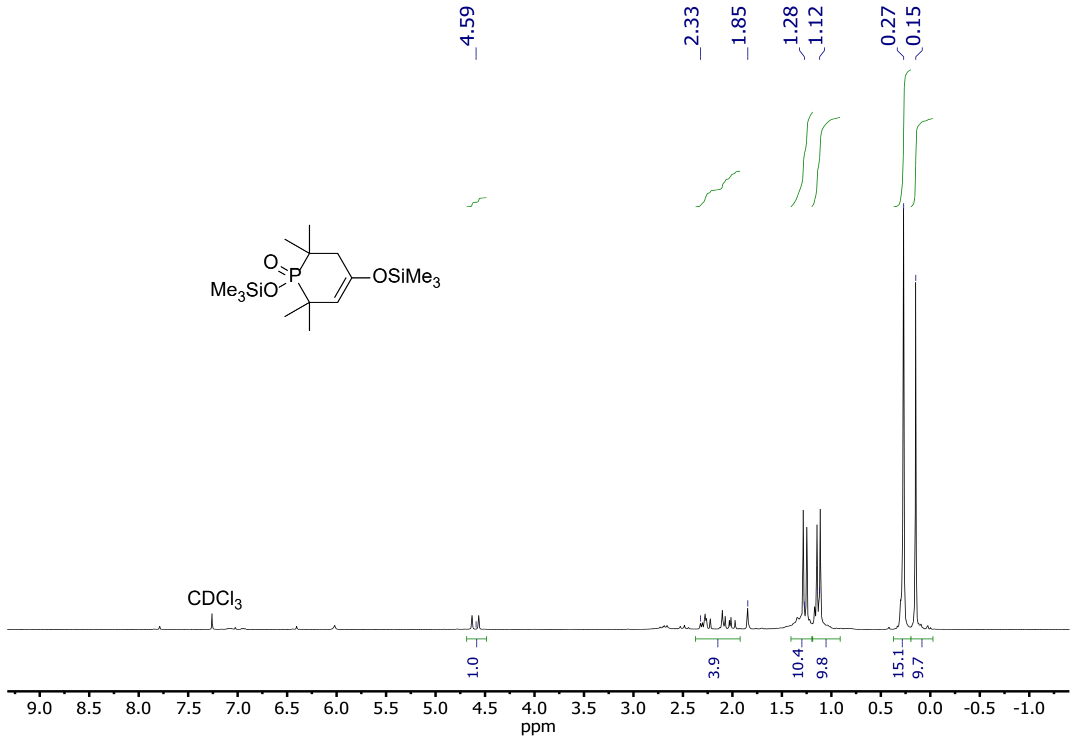

Supplementary Figure 47. ^{1}H NMR (400 MHz, $CDCl_3$) spectrum of 2,2,6,6-tetramethyl-1,4-bis((trimethylsilyl)oxy)-2,3,6-trihydrophosphinine 1-oxide, compound **4**.

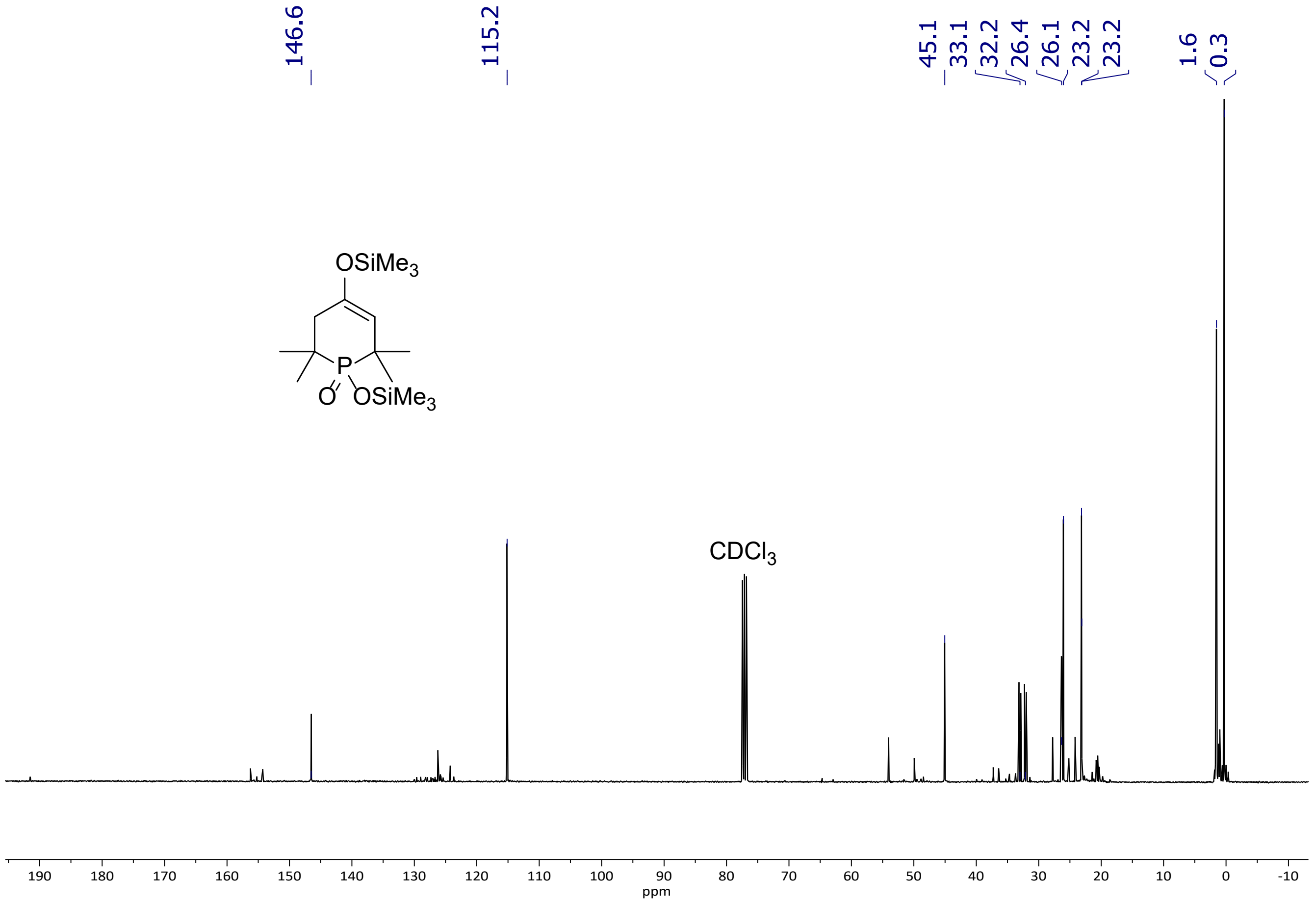

Supplementary Figure 48. $^{13}C\{^1H\}$ NMR (101 MHz, $CDCl_3$) spectrum of 2,2,6,6-tetramethyl-1,4-bis((trimethylsilyl)oxy)-2,3,6-trihydrophosphinine 1-oxide, compound **4**.

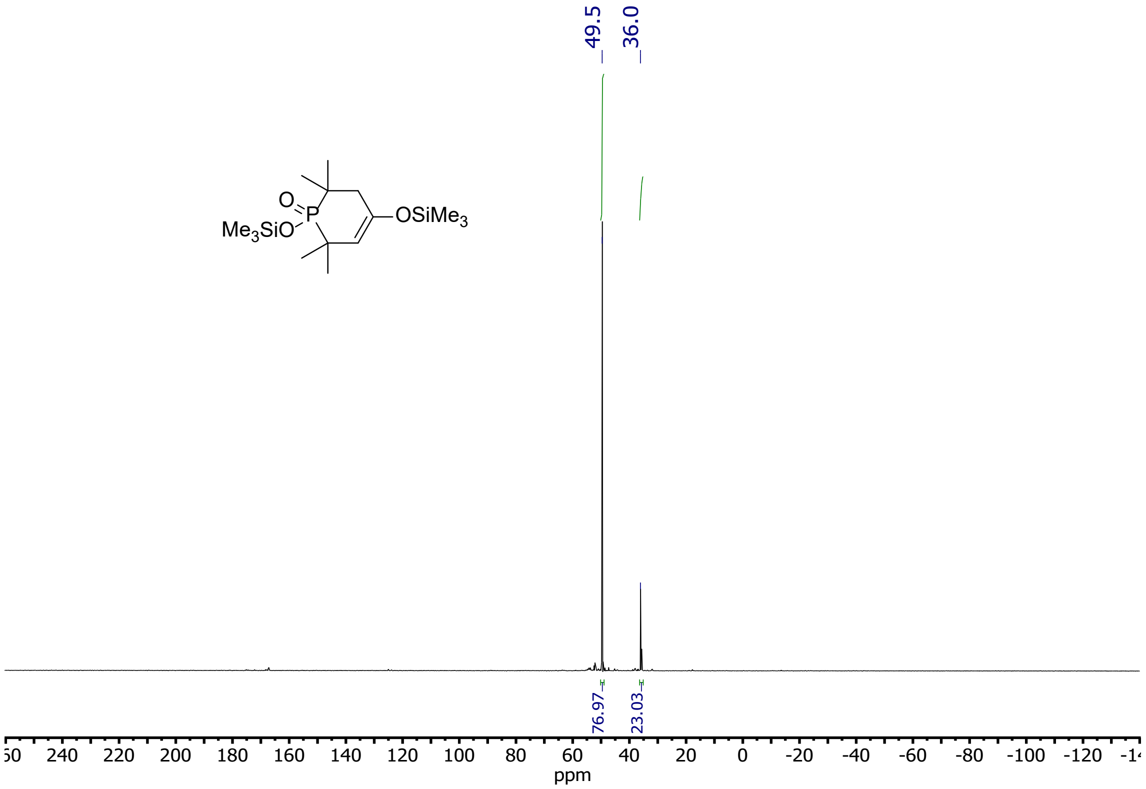

Supplementary Figure 49. ^{31}P NMR (400 MHz, $CDCl_3$) spectrum of 2,2,6,6-tetramethyl-1,4-bis((trimethylsilyl)oxy)-2,3,6-trihydrophosphinine 1-oxide, compound **4**.

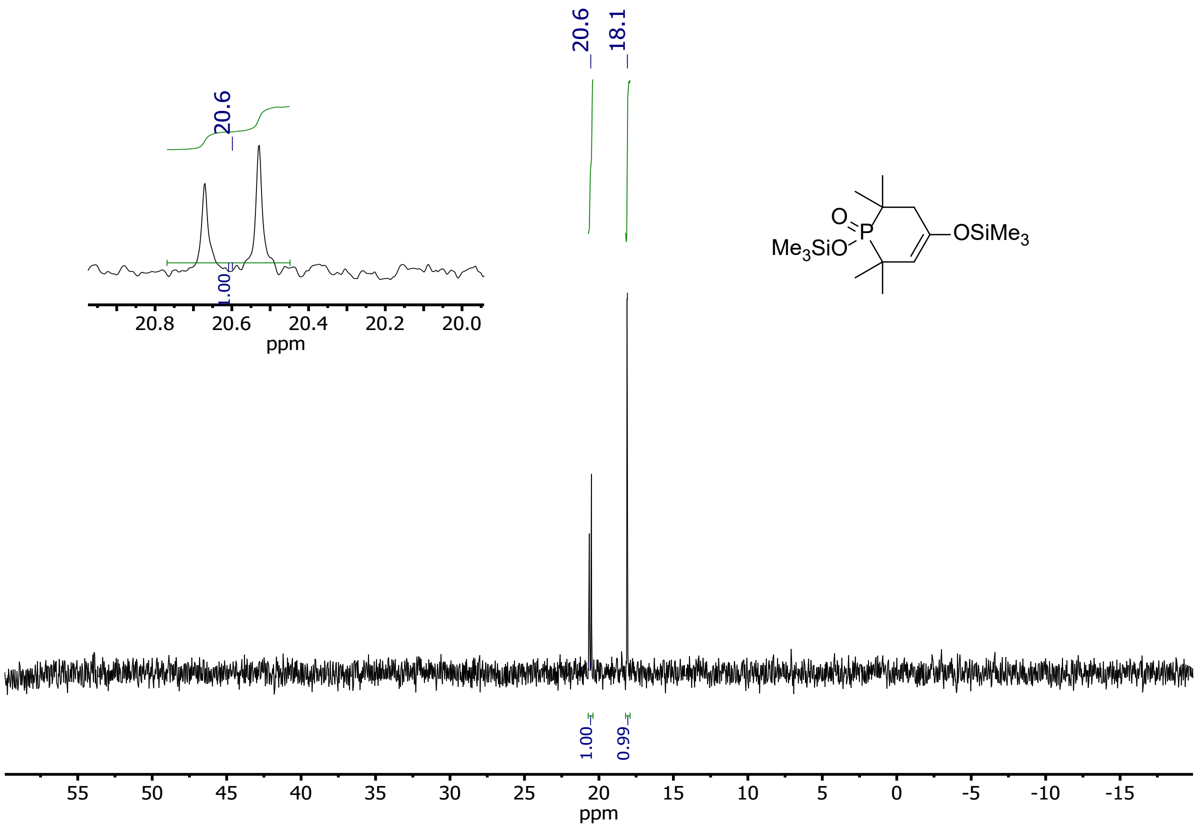

Supplementary Figure 50. $^{29}Si\{^1H\}$ NMR (60 MHz, $CDCl_3$) spectrum of 2,2,6,6-tetramethyl-1,4-bis((trimethylsilyl)oxy)-2,3,6-trihydrophosphinine 1-oxide, compound **4**.

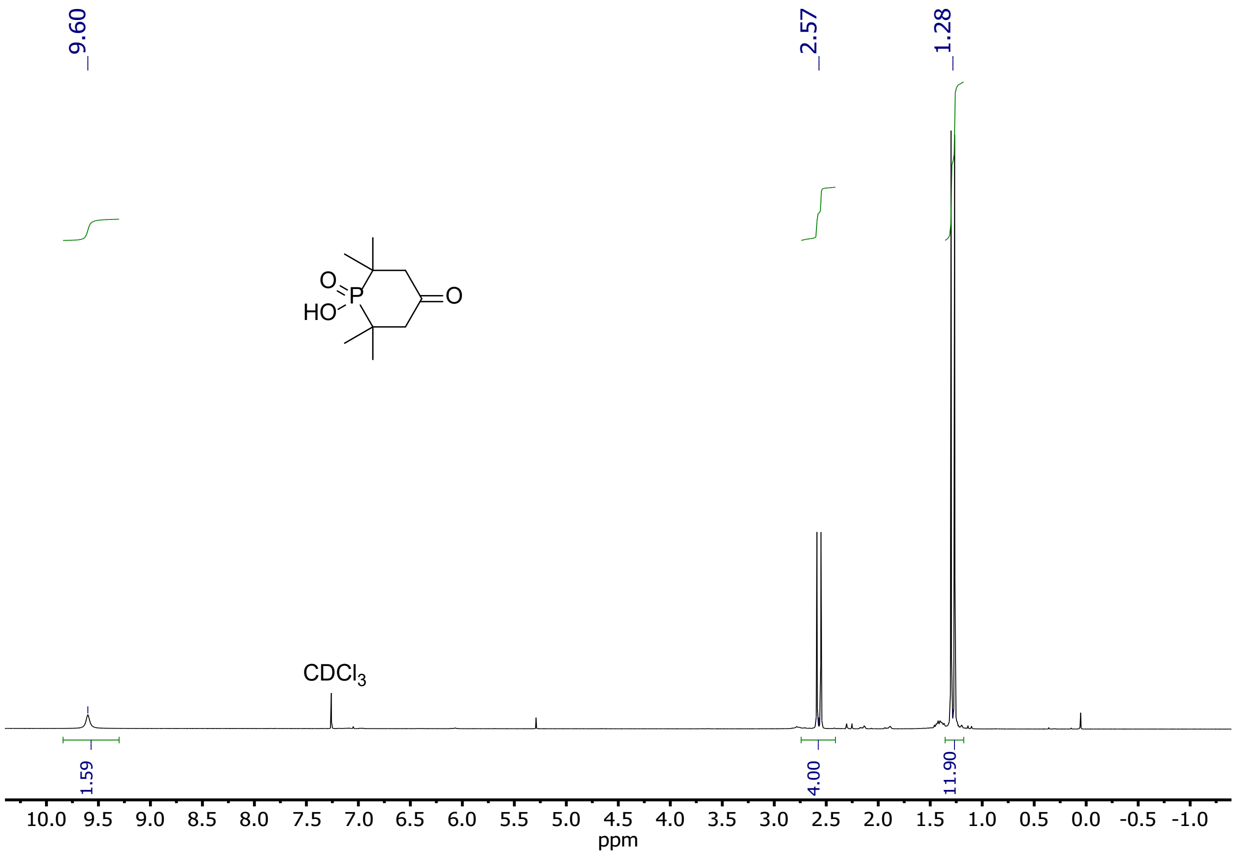

Supplementary Figure 51. ^1H NMR (400 MHz, $CDCl_3$) spectrum of 2,2,6,6-tetramethylphosphorinic acid, compound **5**.

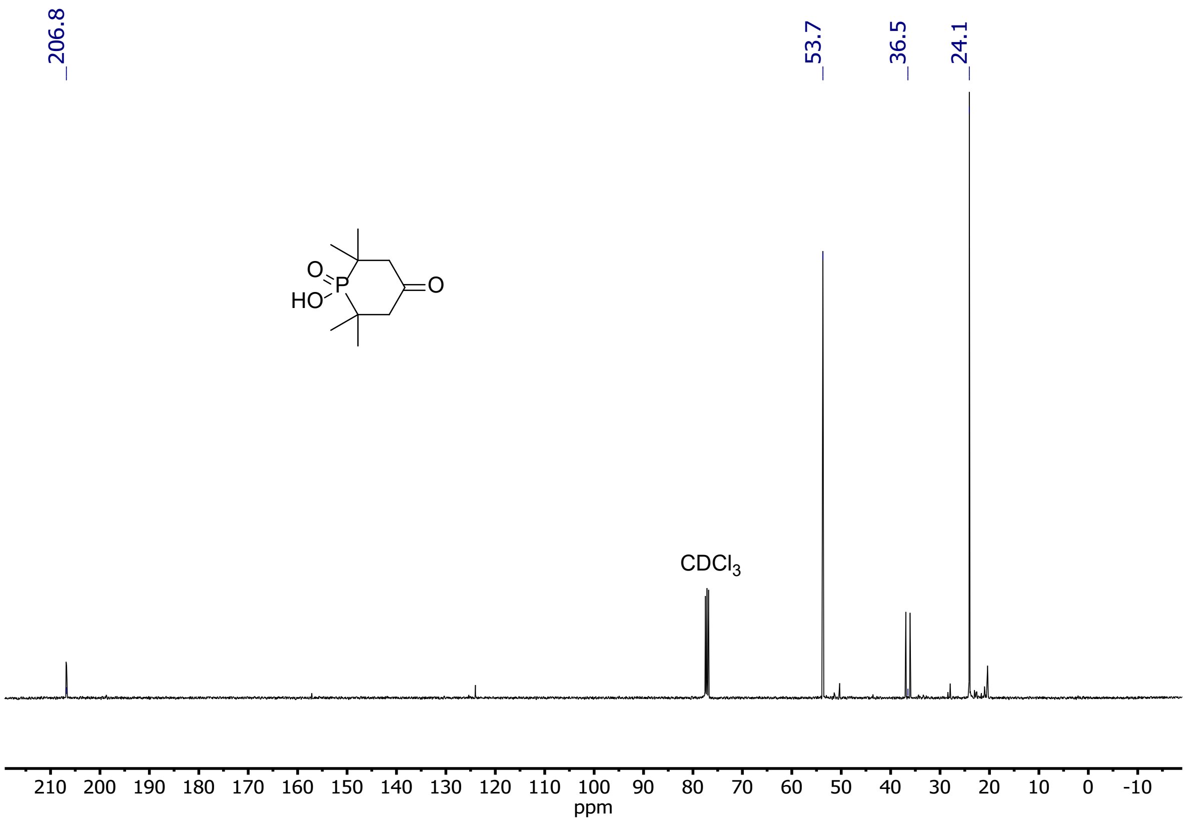

Supplementary Figure 52. $^{13}C\{^1H\}$ NMR (101 MHz, $CDCl_3$) spectrum of 2,2,6,6-tetramethylphosphorinic acid, compound **5**.

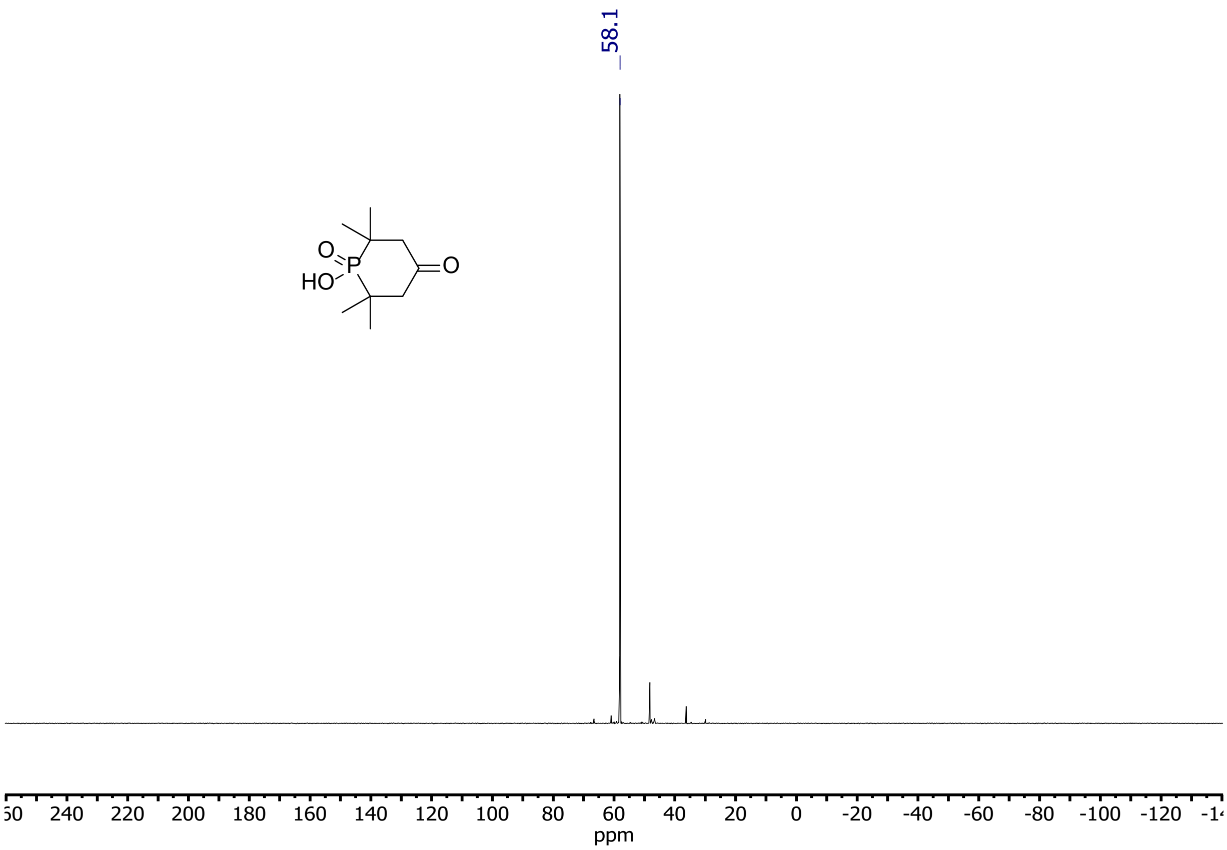

Supplementary Figure 53. $^{31}P\{^1H\}$ NMR (162 MHz, $CDCl_3$) spectrum of 2,2,6,6-tetramethylphosphorinic acid, compound **5**.

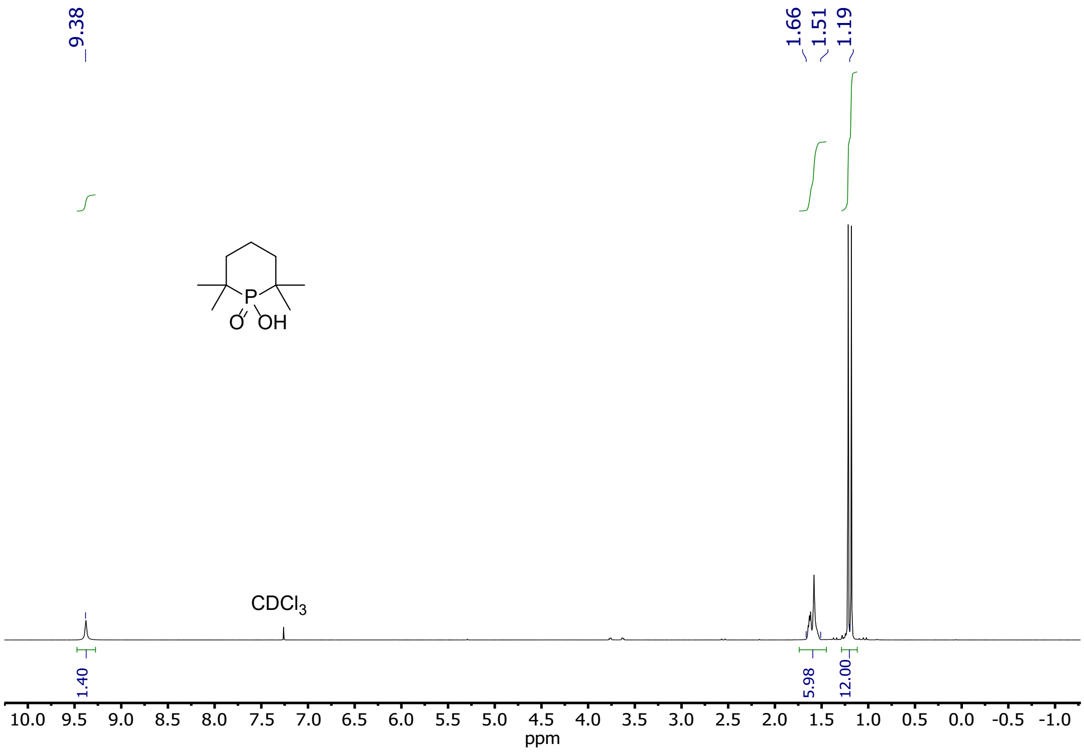

Supplementary Figure 54. ^{1}H NMR (400 MHz, $CDCl_3$) spectrum of 2,2,6,6-tetramethylphosphaninic acid, compound **6**.

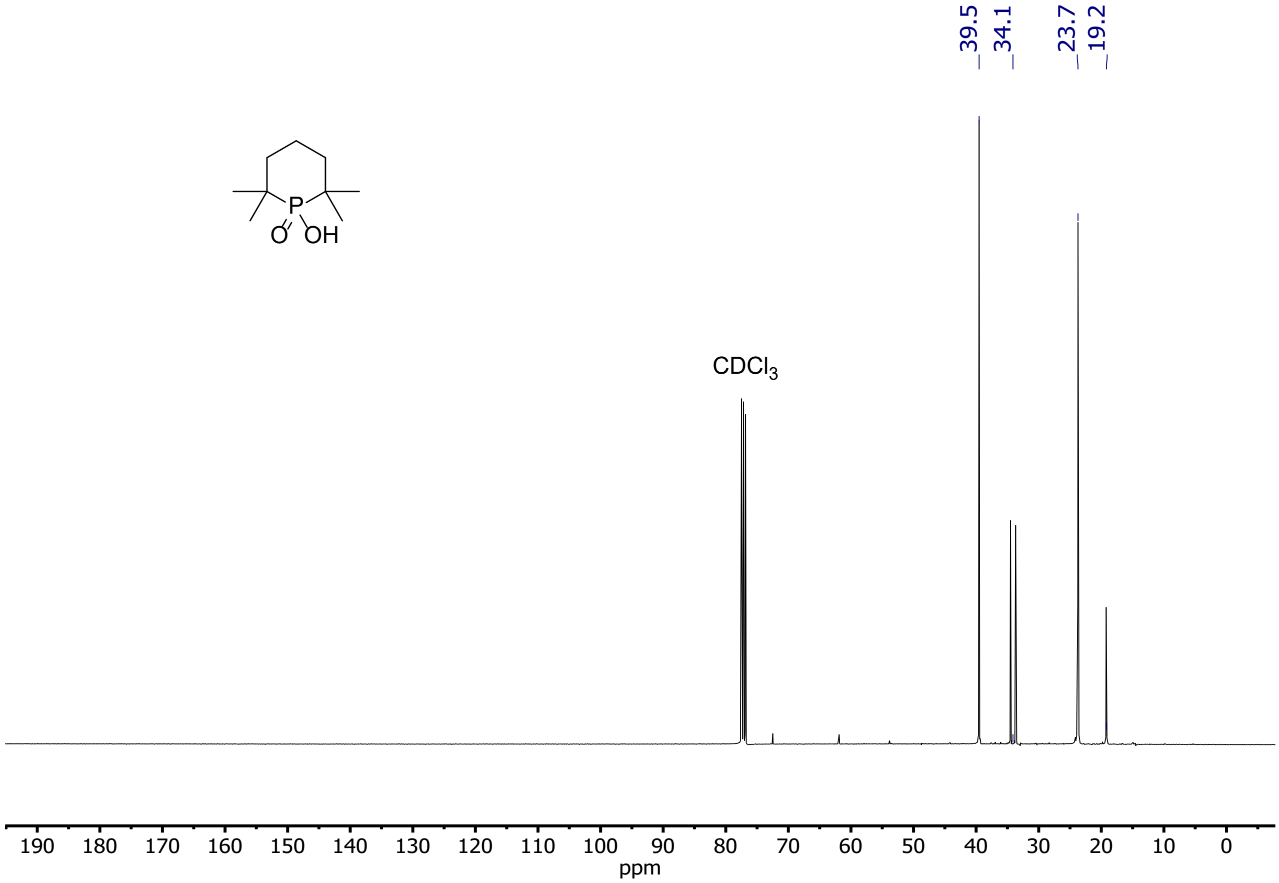

Supplementary Figure 55. $^{13}C\{^{1}H\}$ NMR (101 MHz, $CDCl_3$) spectrum of 2,2,6,6-tetramethylphosphaninic acid, compound **6**.

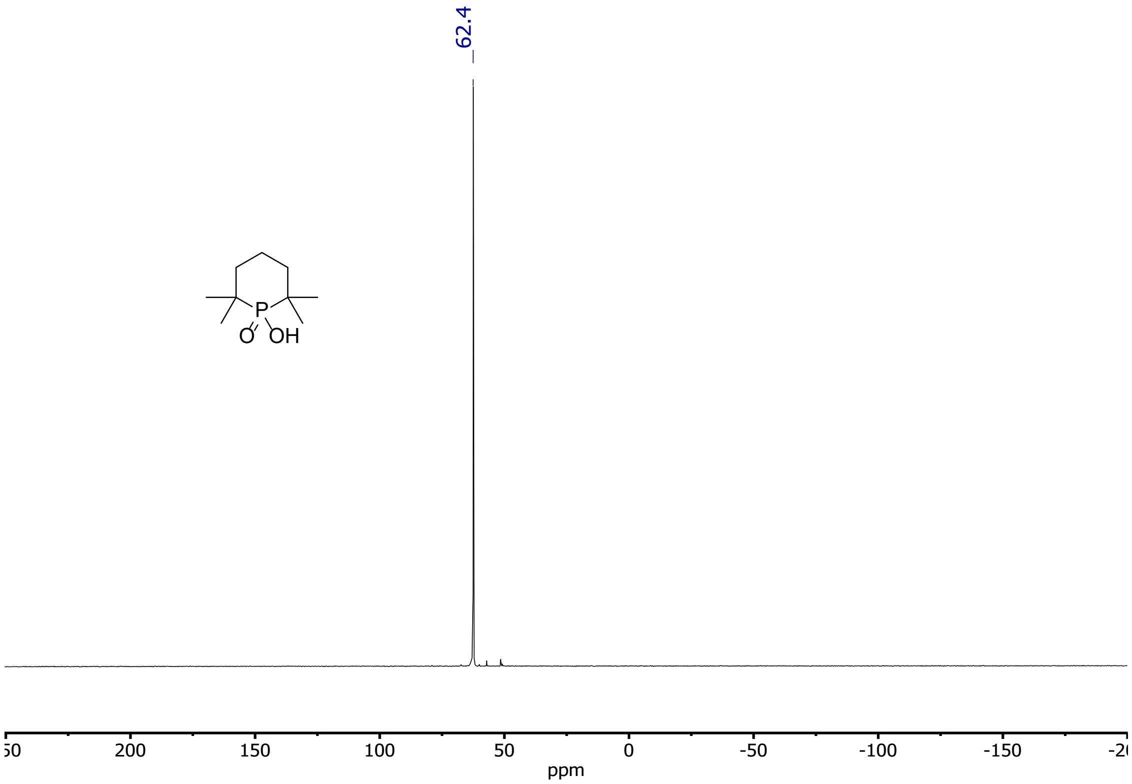

Supplementary Figure 56. $^{31}P\{^{1}H\}$ NMR (162 MHz, $CDCl_3$) spectrum of 2,2,6,6-tetramethylphosphaninic acid, compound **6**.

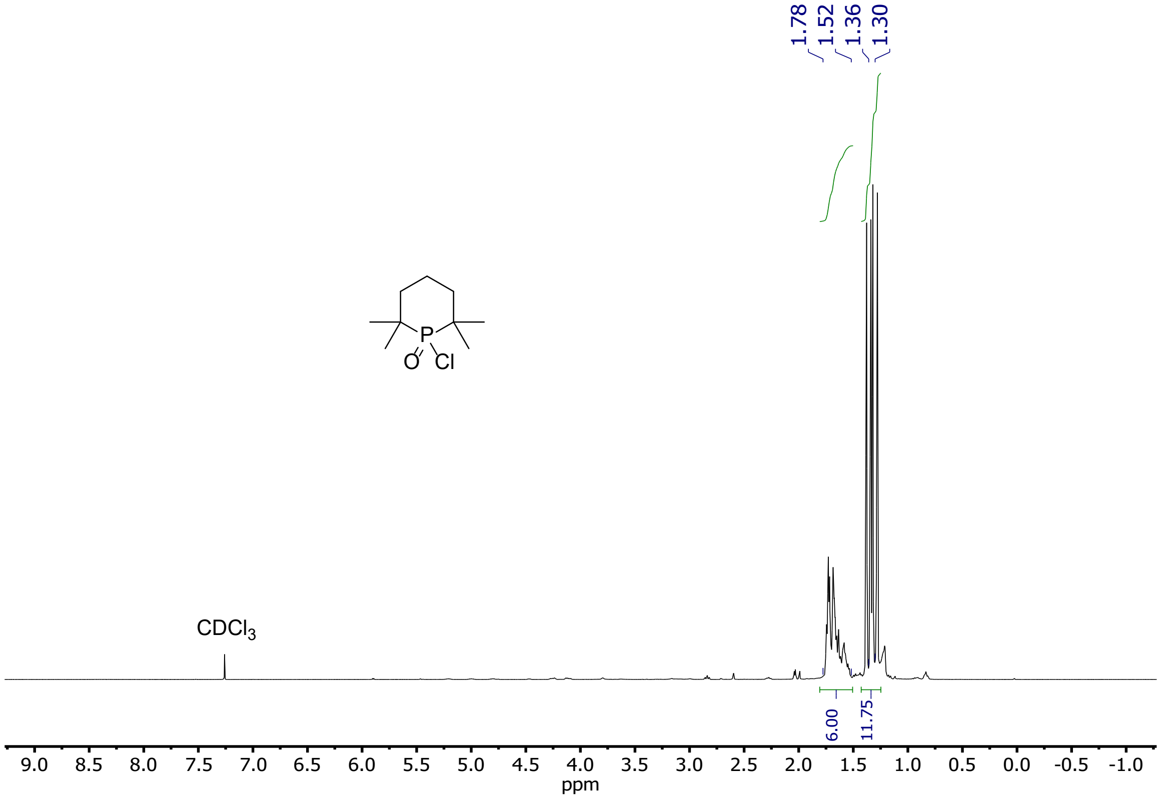

Supplementary Figure 57. ^{1}H NMR (400 MHz, $CDCl_3$) spectrum of 2,2,6,6-tetramethylphosphaninic chloride, compound **7**.

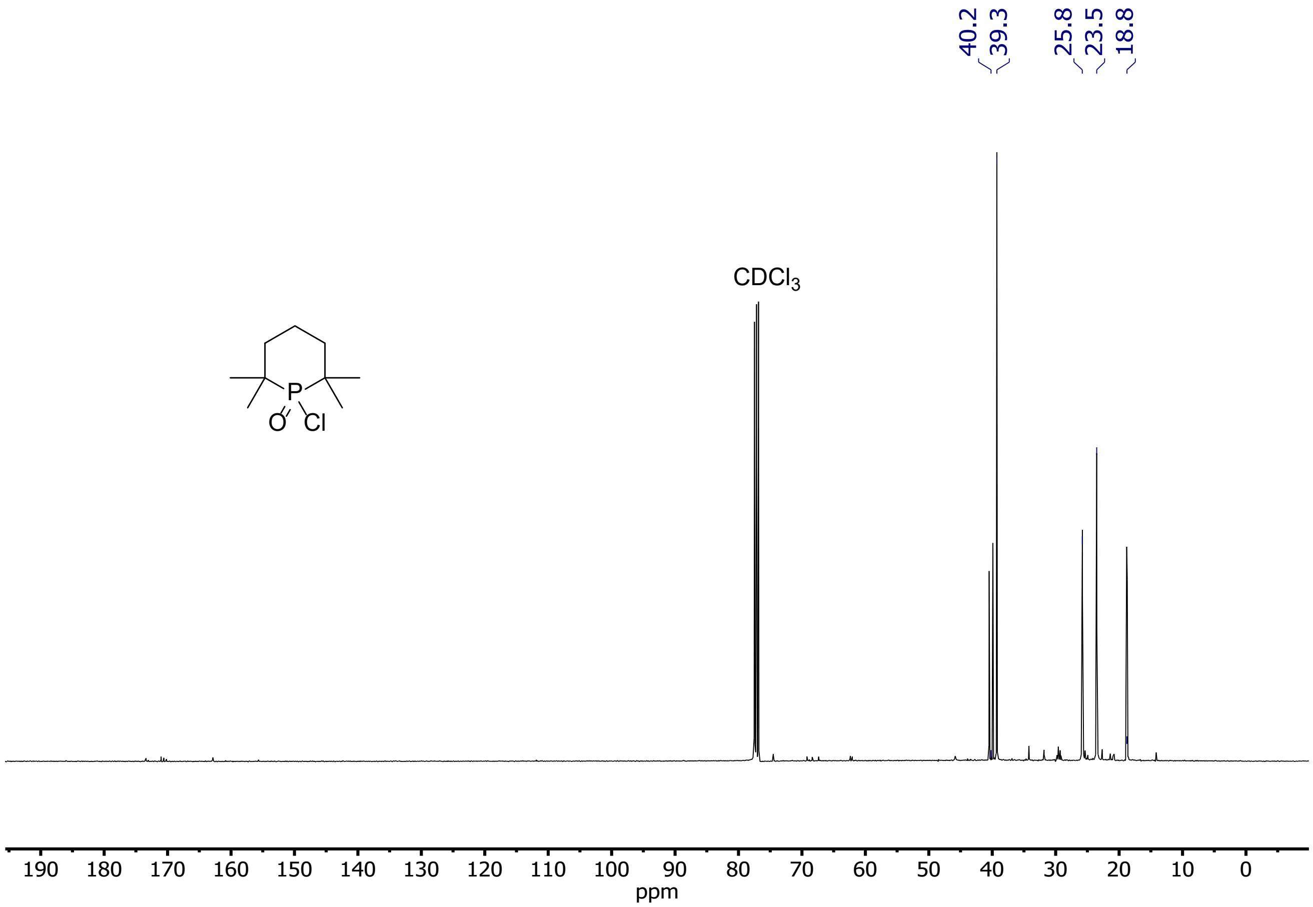

Supplementary Figure 58. $^{13}C\{^{1}H\}$ NMR (101 MHz, $CDCl_3$) spectrum of 2,2,6,6-tetramethylphosphaninic chloride, compound **7**.

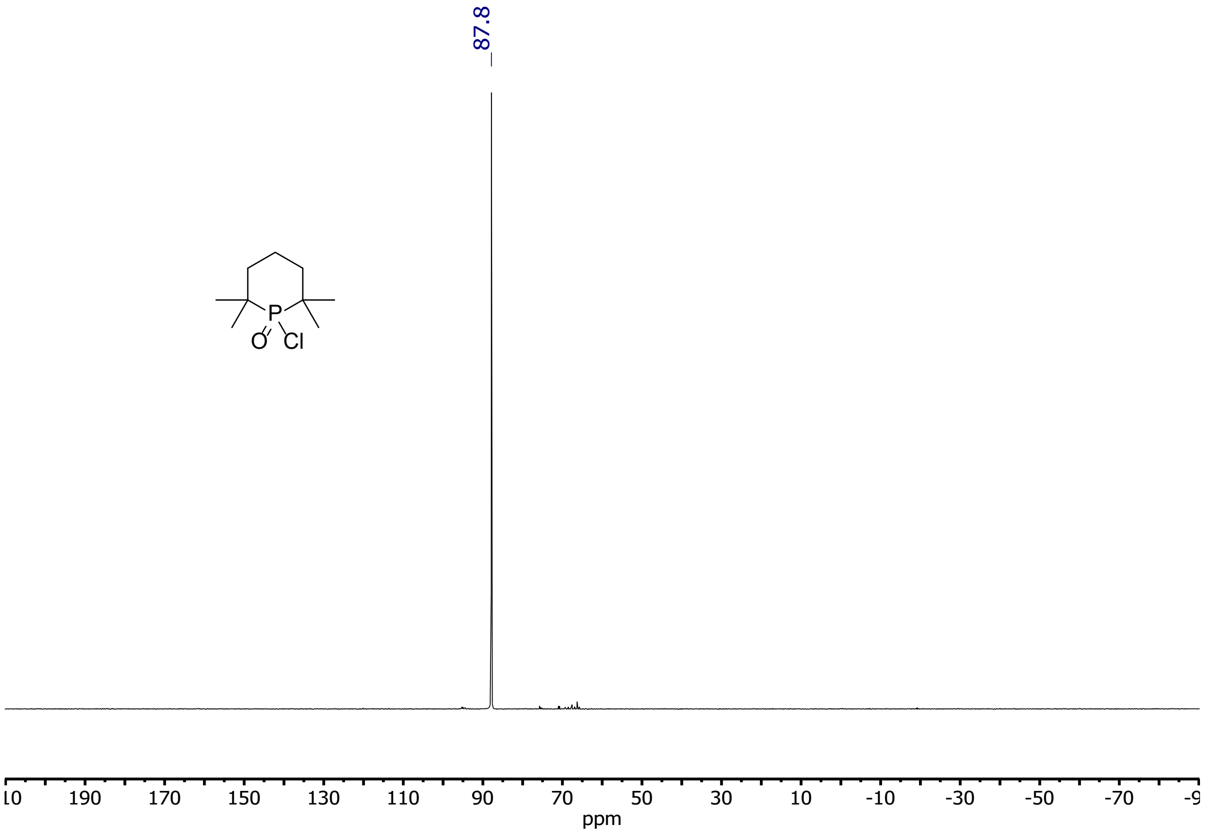

Supplementary Figure 59. $^{31}P\{^{1}H\}$ NMR (162 MHz, $CDCl_3$) spectrum of 2,2,6,6-tetramethylphosphaninic chloride, compound **7**.

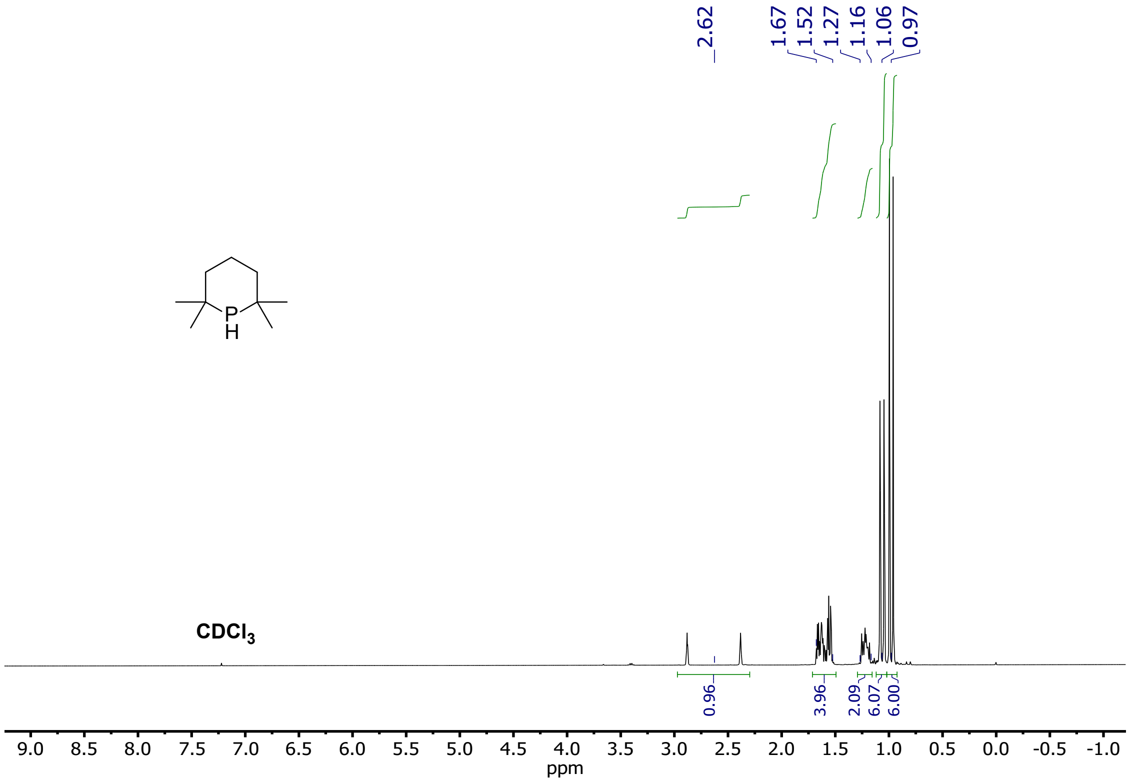

Supplementary Figure 60. ^{1}H NMR (400 MHz, $CDCl_3$) spectrum of 2,2,6,6-tetramethylphosphinane (**TMPhos**), compound **8**.

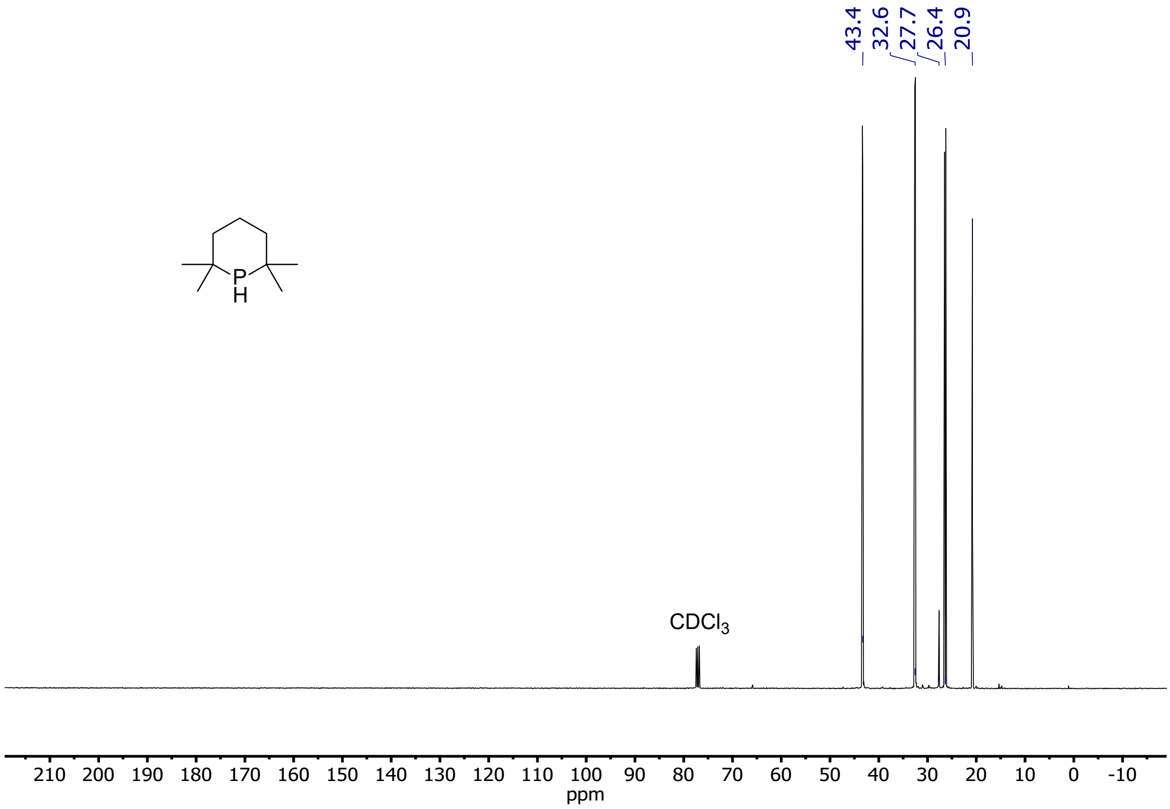

Supplementary Figure 61. $^{13}C\{^{1}H\}$ NMR (101 MHz, $CDCl_3$) spectrum of 2,2,6,6-tetramethylphosphinane (**TMPhos**), compound **8**.

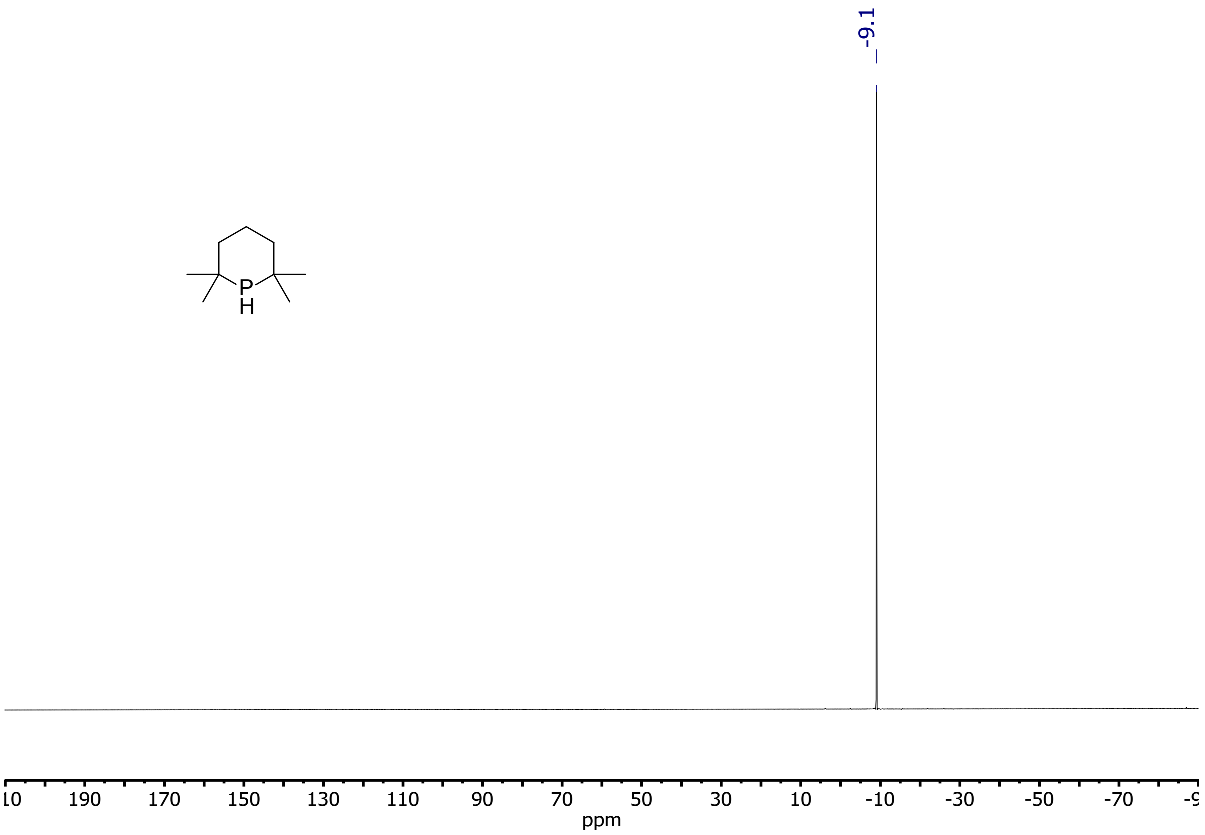

Supplementary Figure 62. $^{31}P\{^{1}H\}$ NMR (162 MHz, $CDCl_3$) spectrum of 2,2,6,6-tetramethylphosphinane (**TMPhos**), compound **8**.

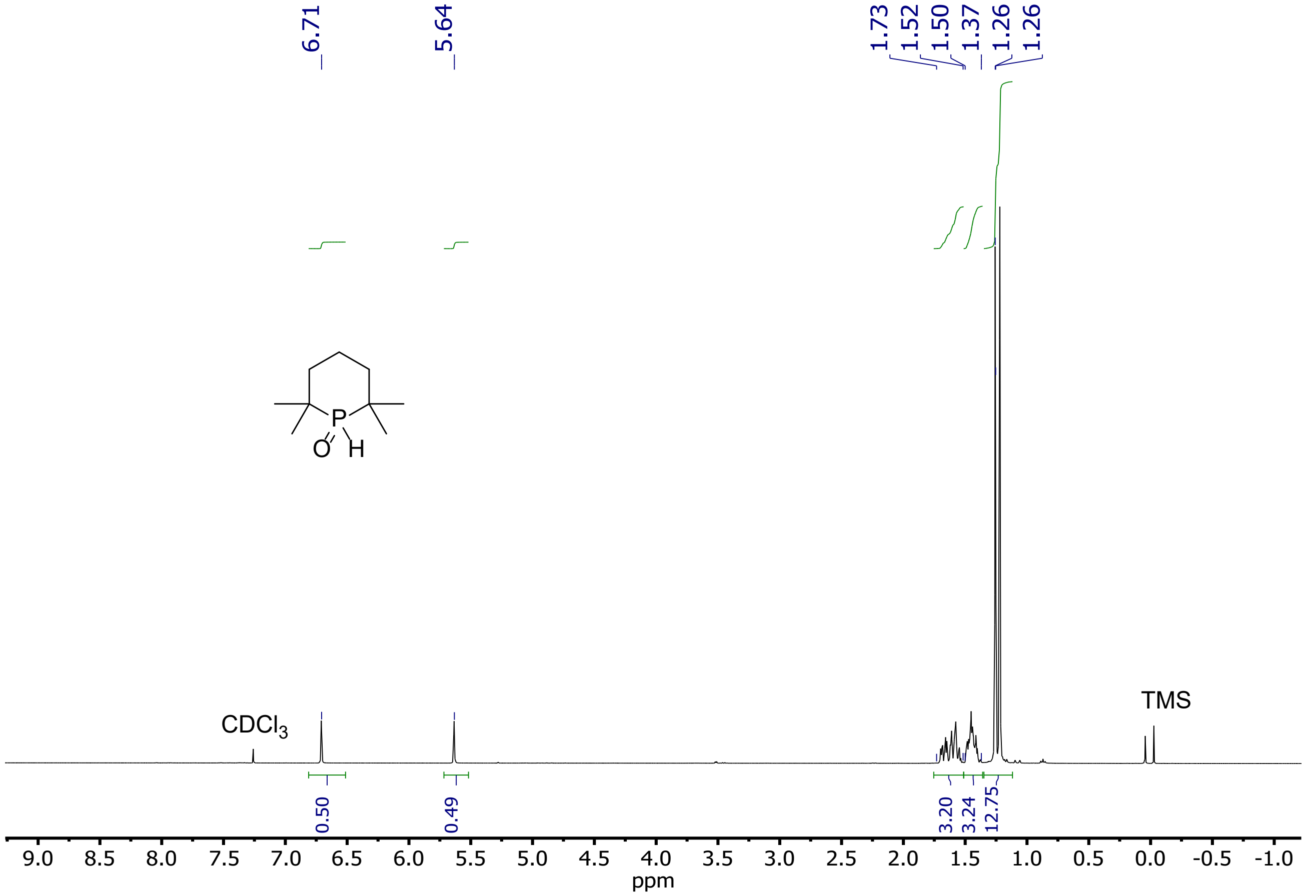

Supplementary Figure 63. ^{1}H NMR (400 MHz, $CDCl_3$) spectrum of 2,2,6,6-tetramethylphosphinane 1-oxide compound **9**.

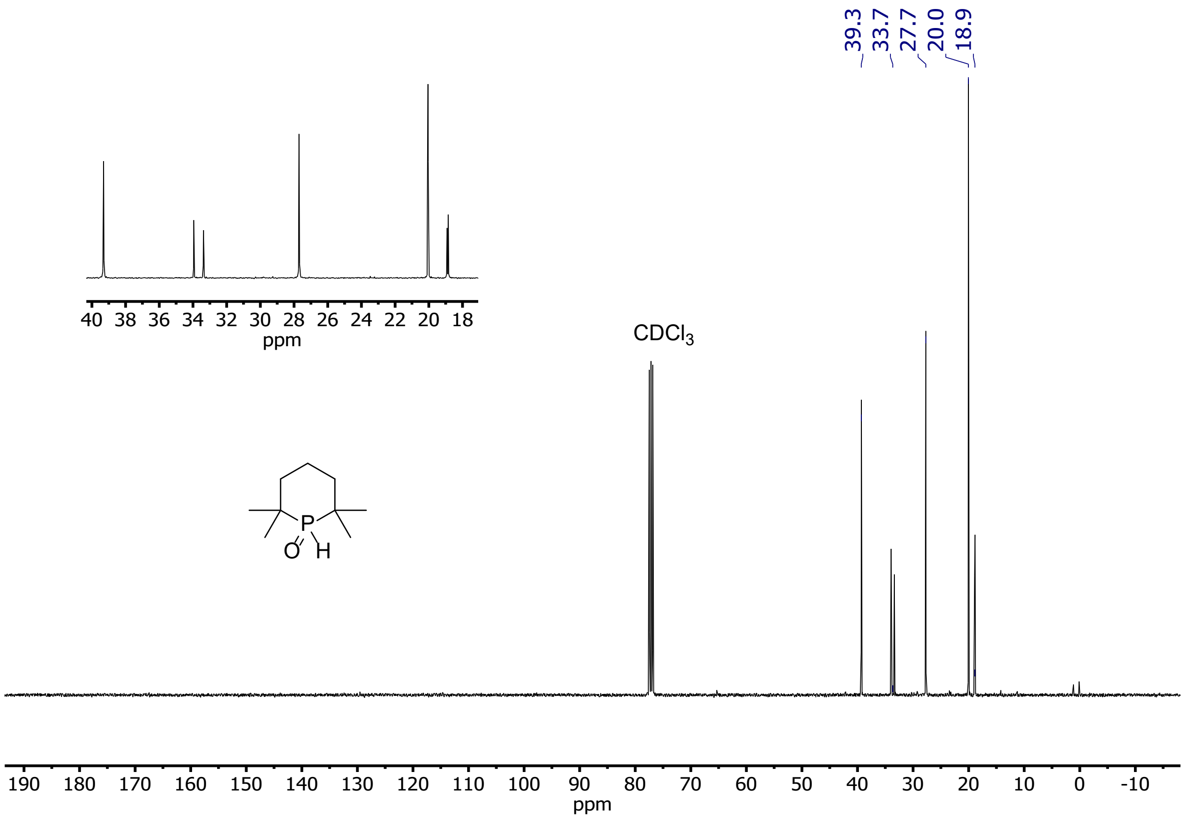

Supplementary Figure 64. $^{13}C\{^1H\}$ NMR (101 MHz, $CDCl_3$) spectrum of 2,2,6,6-tetramethylphosphinane 1-oxide compound **9**.

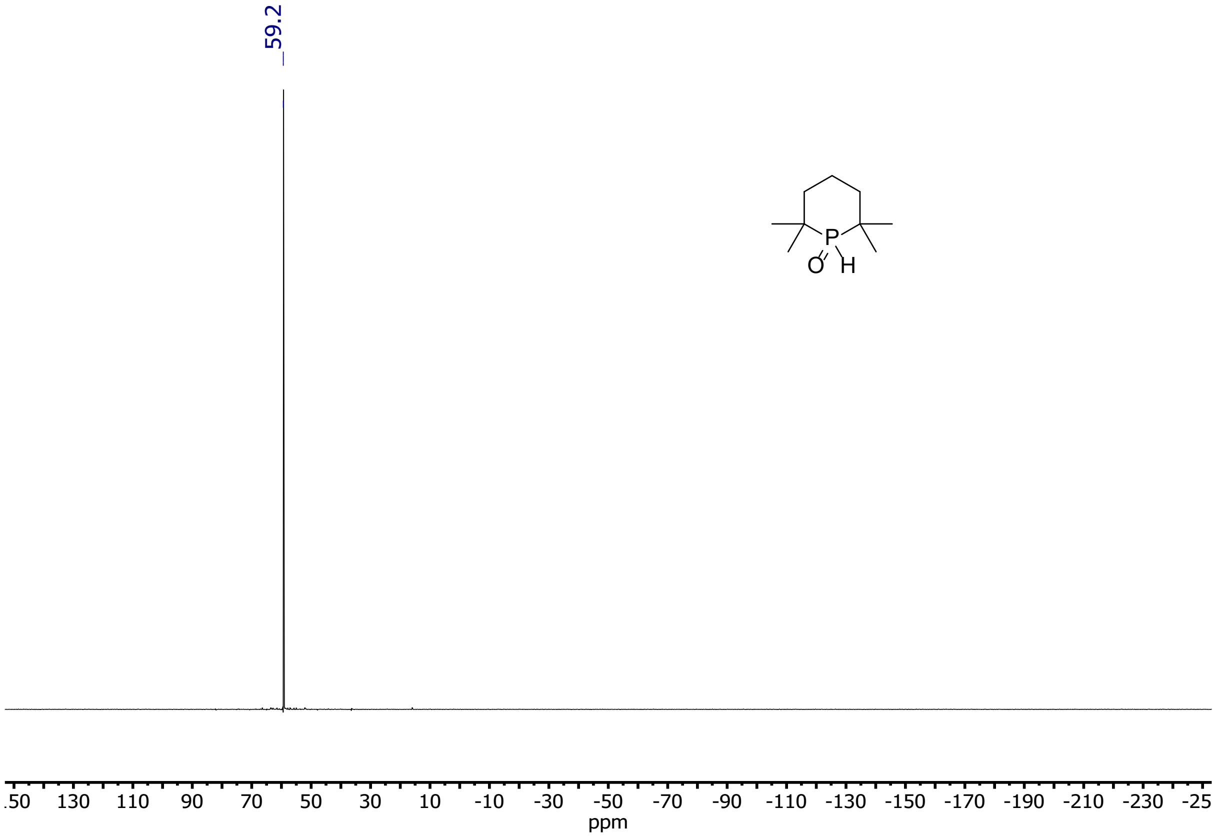

Supplementary Figure 65. $^{31}P\{^1H\}$ NMR (162 MHz, $CDCl_3$) spectrum of 2,2,6,6-tetramethylphosphinane 1-oxide compound **9**.

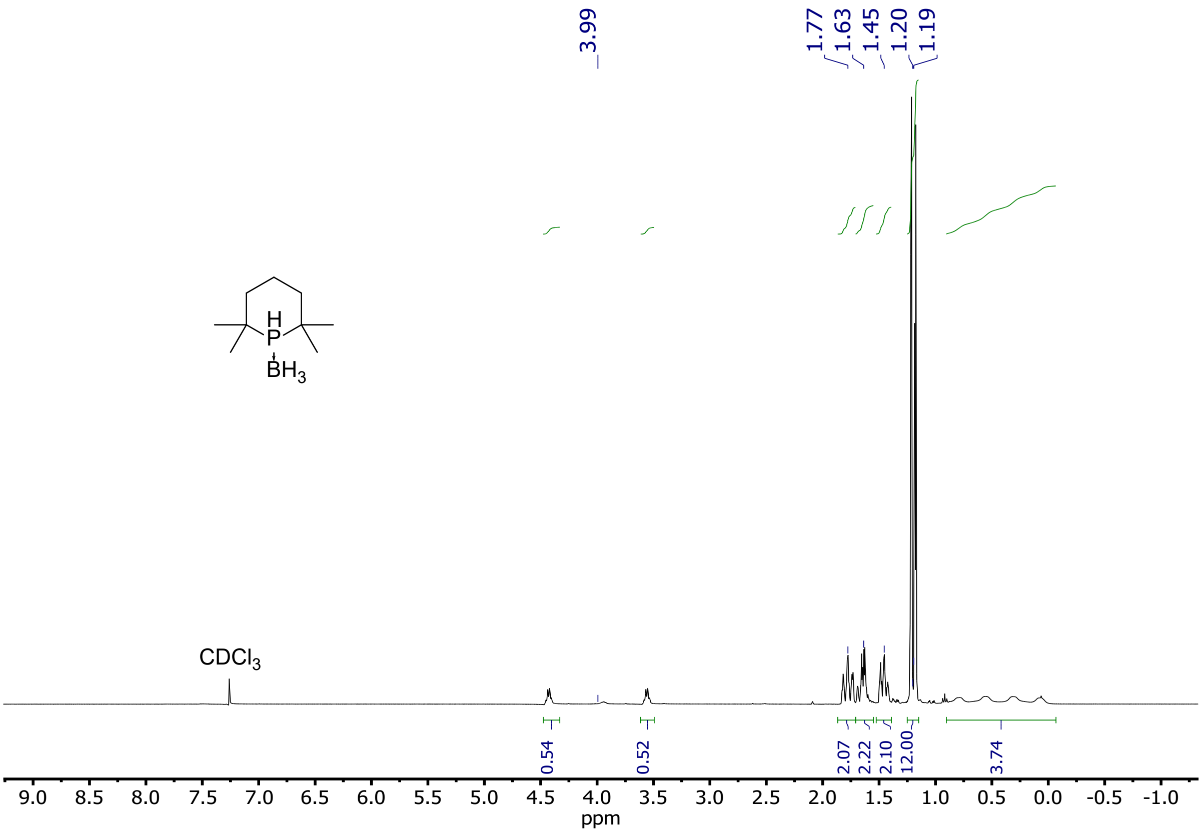

Supplementary Figure 66. ^{1}H NMR (400 MHz, $CDCl_3$) spectrum of 2,2,6,6-tetramethylphosphinane borane complex, compound **10**.

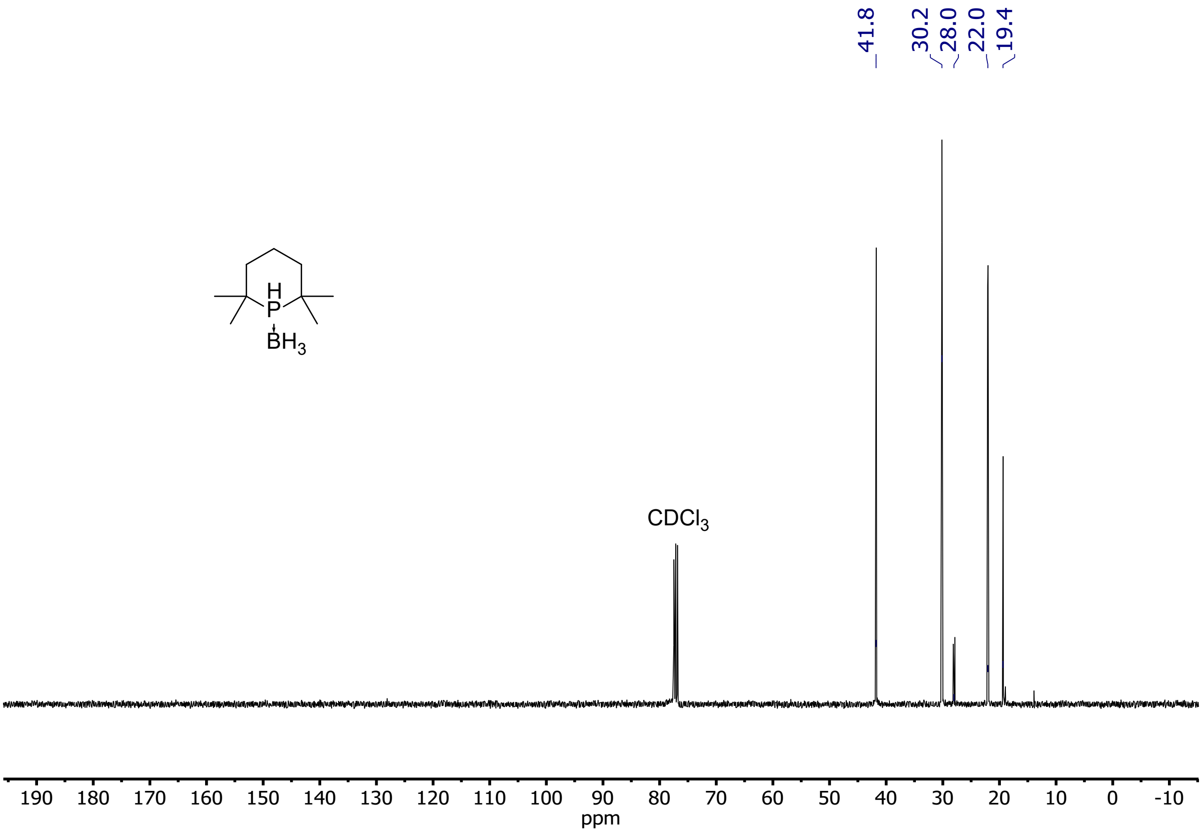

Supplementary Figure 67. $^{13}C\{^{1}H\}$ NMR (101 MHz, $CDCl_3$) spectrum of 2,2,6,6-tetramethylphosphinane borane complex, compound **10**.

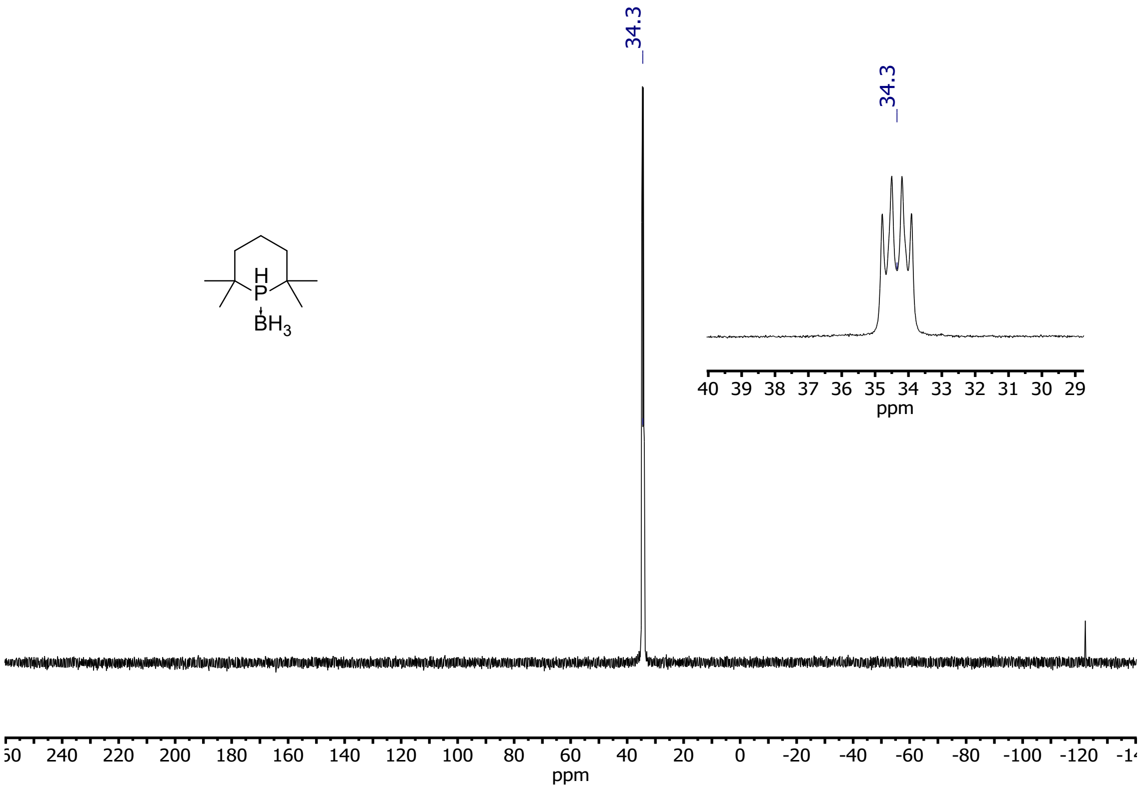

Supplementary Figure 68. $^{31}P\{^{1}H\}$ NMR (162 MHz, $CDCl_3$) spectrum of 2,2,6,6-tetramethylphosphinane borane complex, compound **10**.

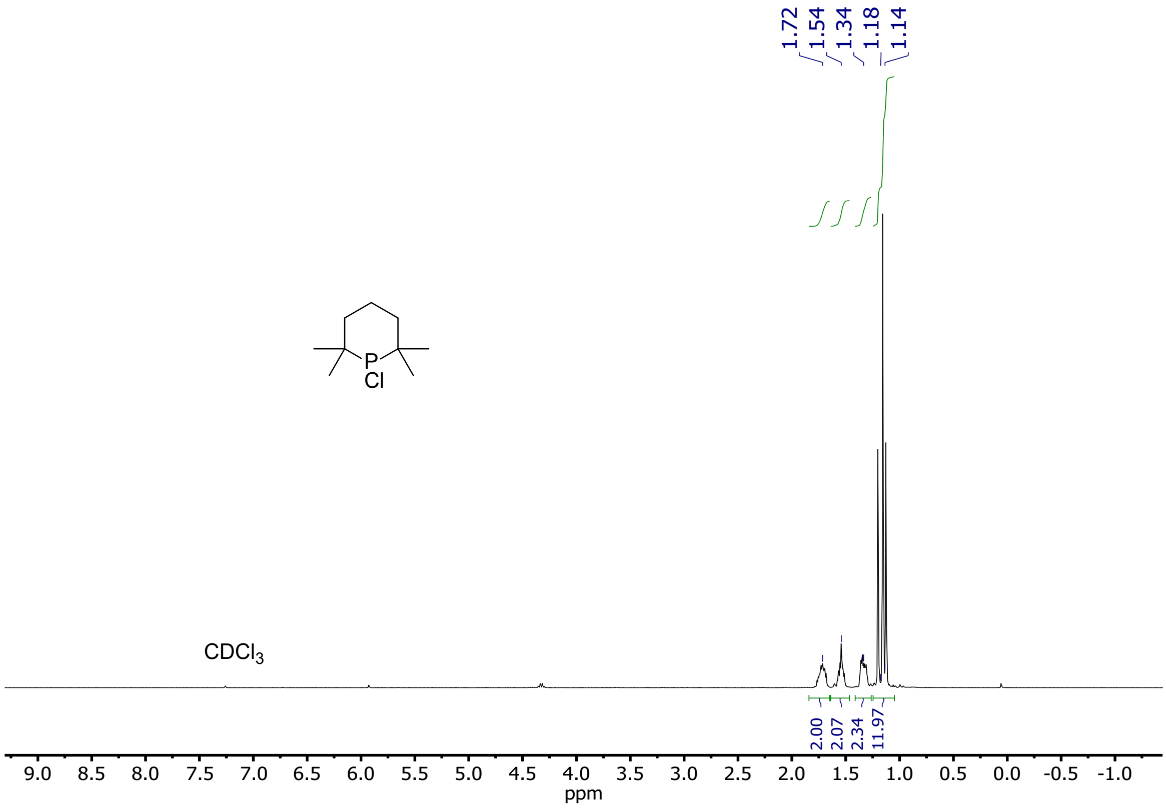

Supplementary Figure 69. ^{1}H NMR (400 MHz, $CDCl_3$) spectrum of 1-chloro-2,2,6,6-tetramethylphosphinane, compound **11**.

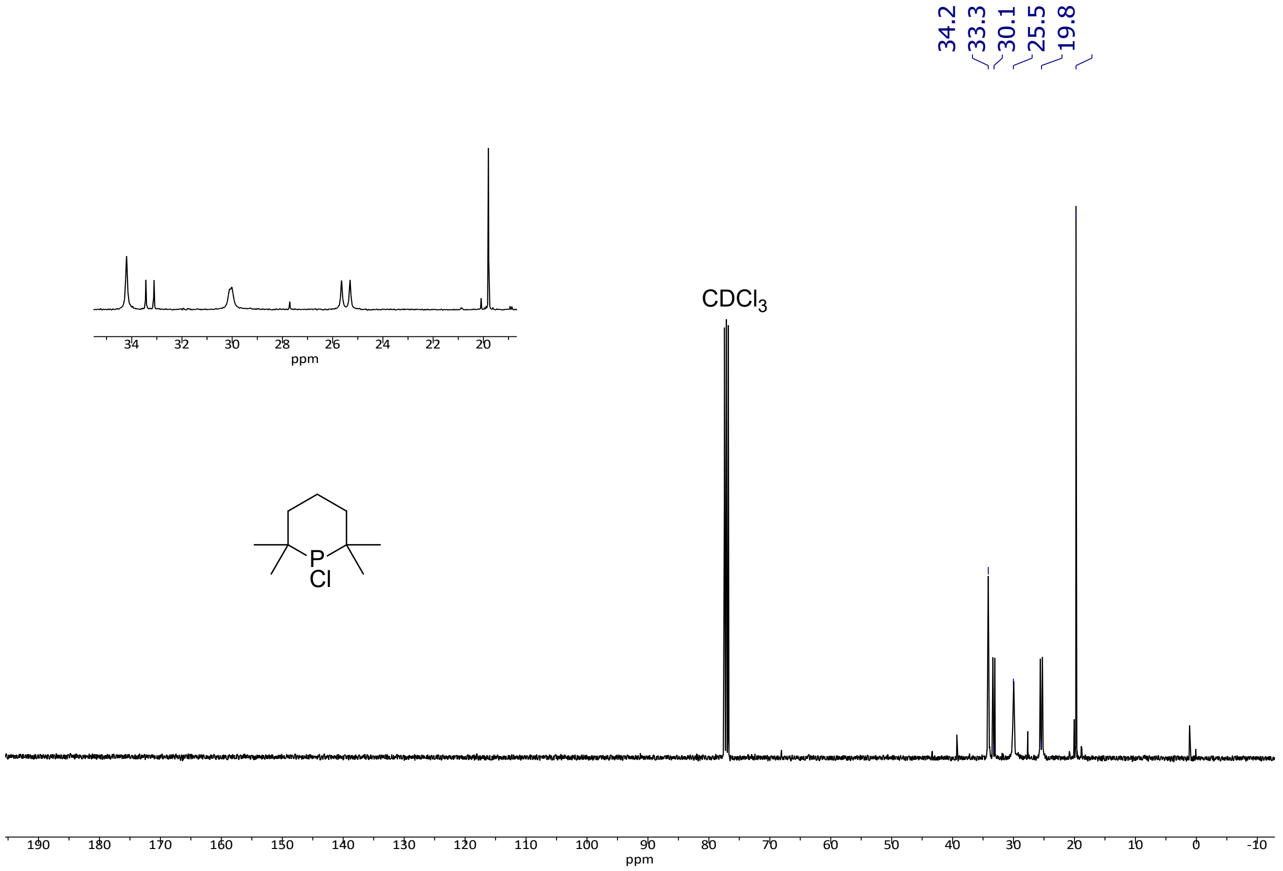

Supplementary Figure 70. $^{13}C\{^1H\}$ NMR (101 MHz, $CDCl_3$) spectrum of 1-chloro-2,2,6,6-tetramethylphosphinane, compound **11**.

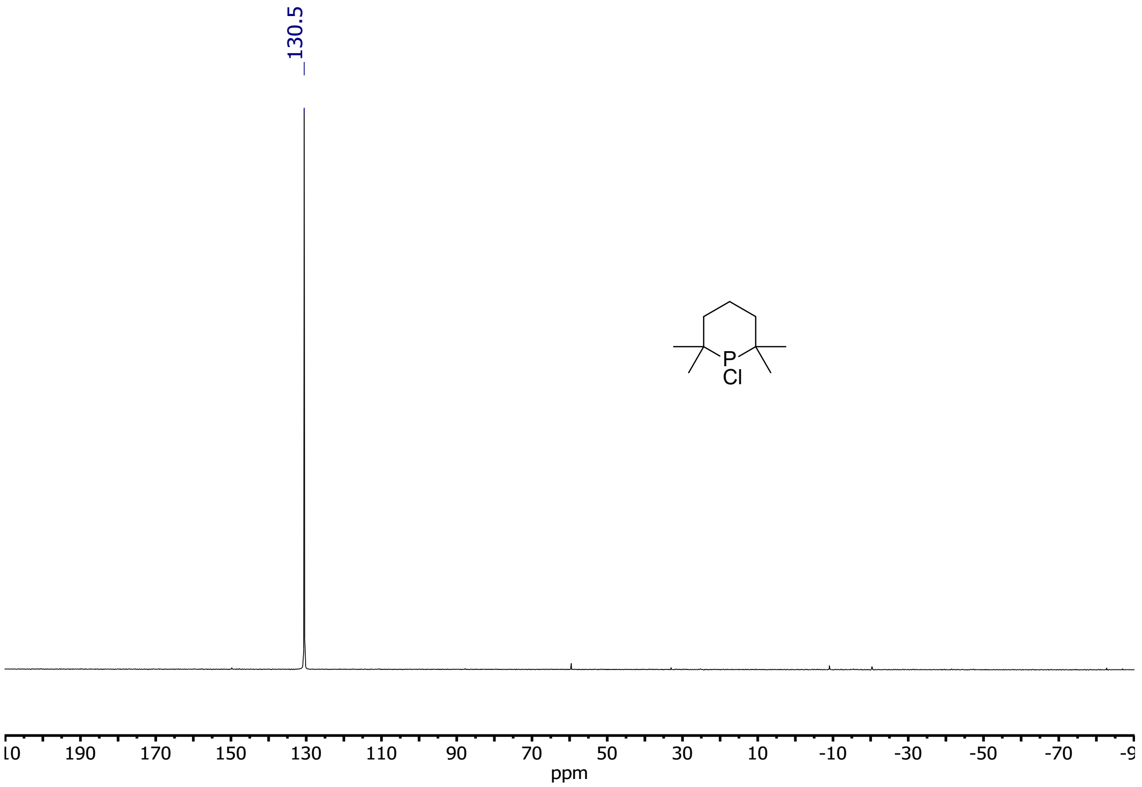

Supplementary Figure 71. $^{31}P\{^1H\}$ NMR (162 MHz, $CDCl_3$) spectrum of 1-chloro-2,2,6,6-tetramethylphosphinane, compound **11**.

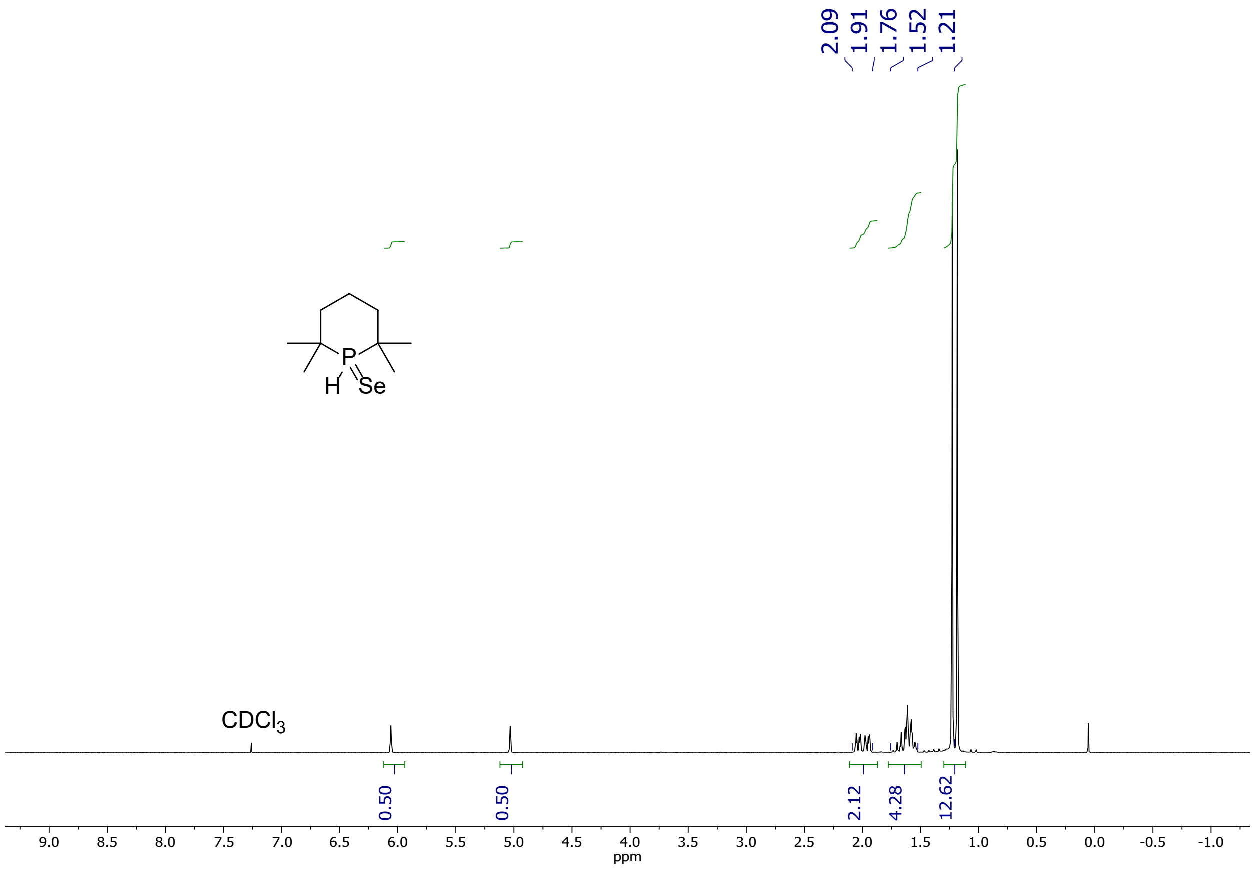

Supplementary Figure 72. ^{1}H NMR (400 MHz, $CDCl_3$) spectrum of 2,2,6,6-tetramethylphosphinane 1-selenide, compound **12**.

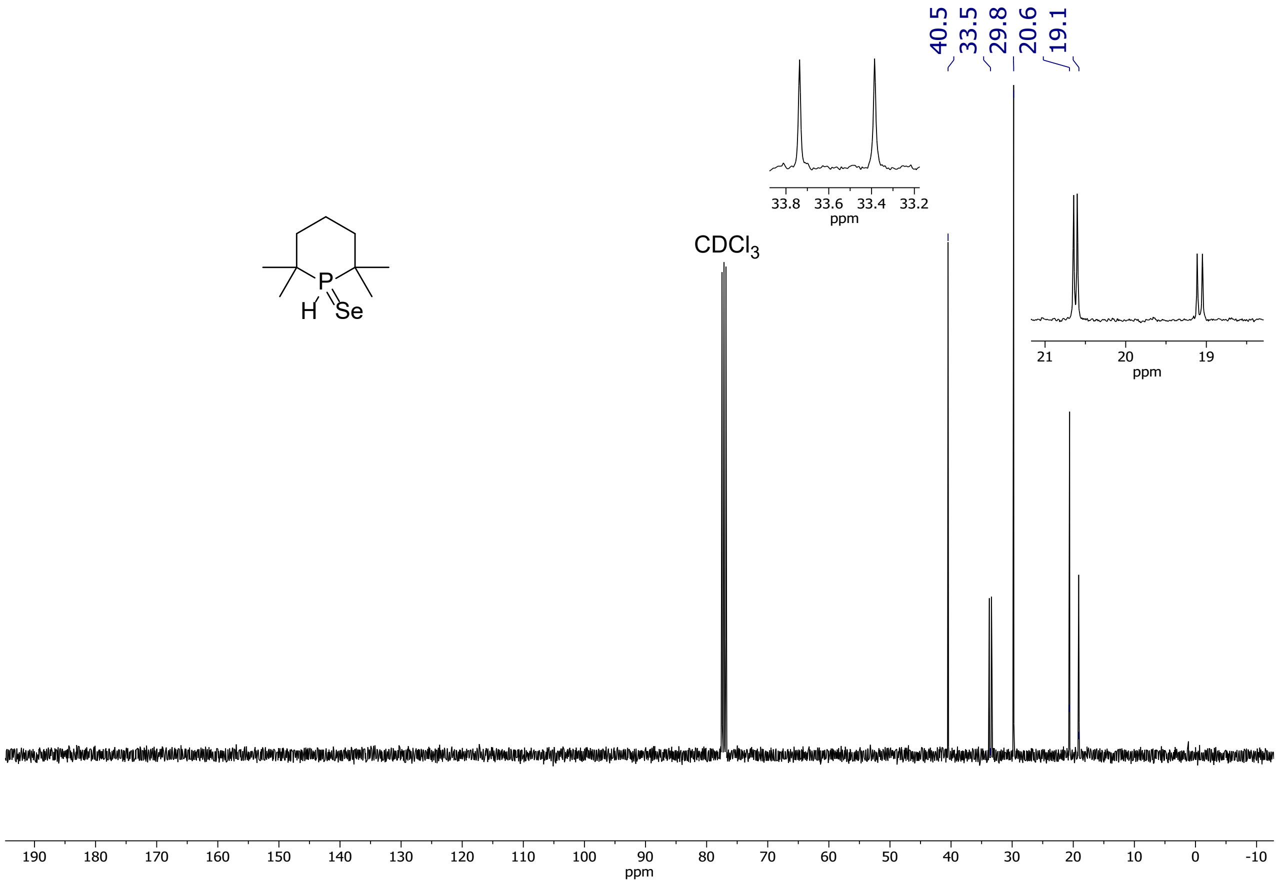

Supplementary Figure 73. $^{13}C\{^{1}H\}$ NMR (101 MHz, $CDCl_3$) spectrum of 2,2,6,6-tetramethylphosphinane 1-selenide, compound **12**.

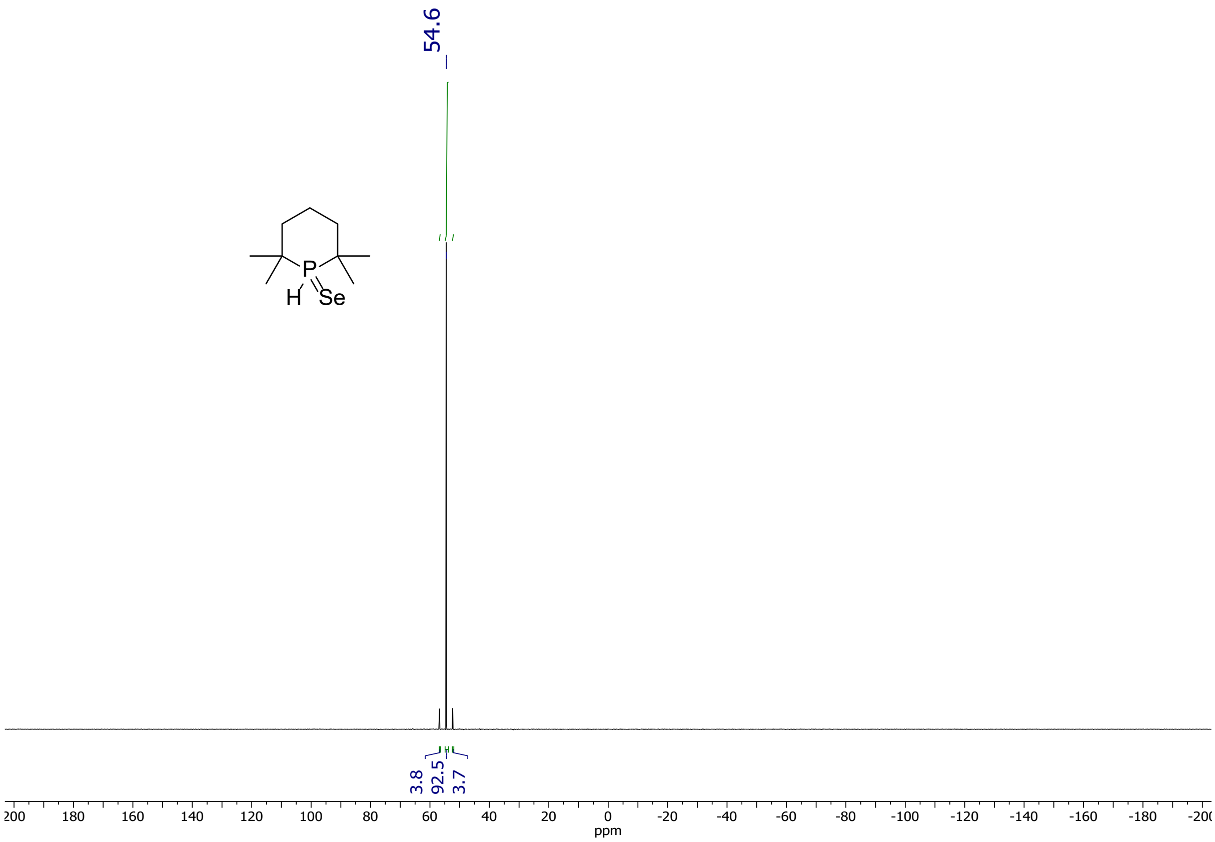

Supplementary Figure 74. $^{31}P\{^{1}H\}$ NMR (162 MHz, $CDCl_3$) spectrum of 2,2,6,6-tetramethylphosphinane 1-selenide, compound **12**.

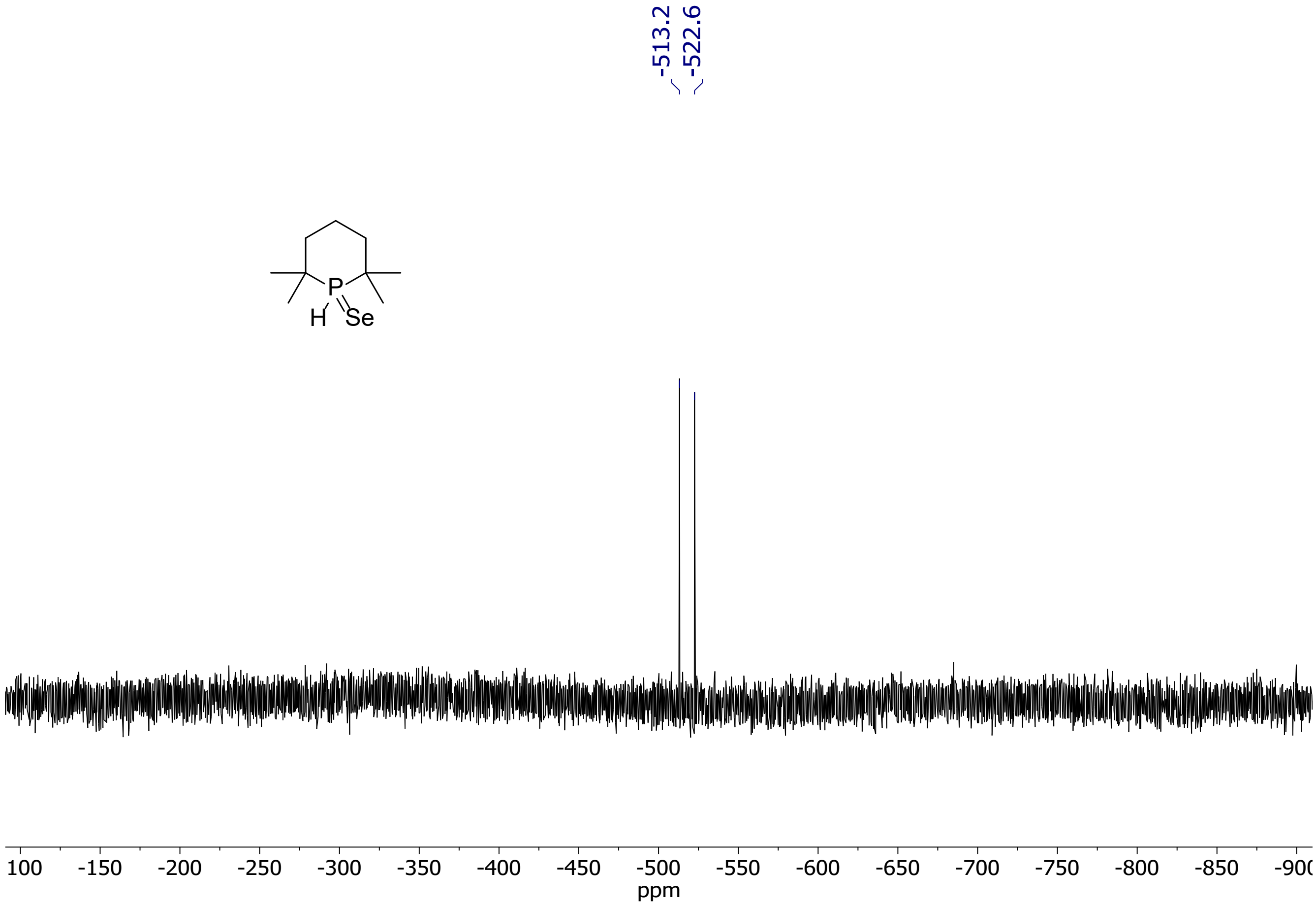

Supplementary Figure 75. $^{77}Se\{^{1}H\}$ NMR (76.3 MHz, $CDCl_3$) spectrum of 2,2,6,6-tetramethylphosphinane 1-selenide, compound **12**.

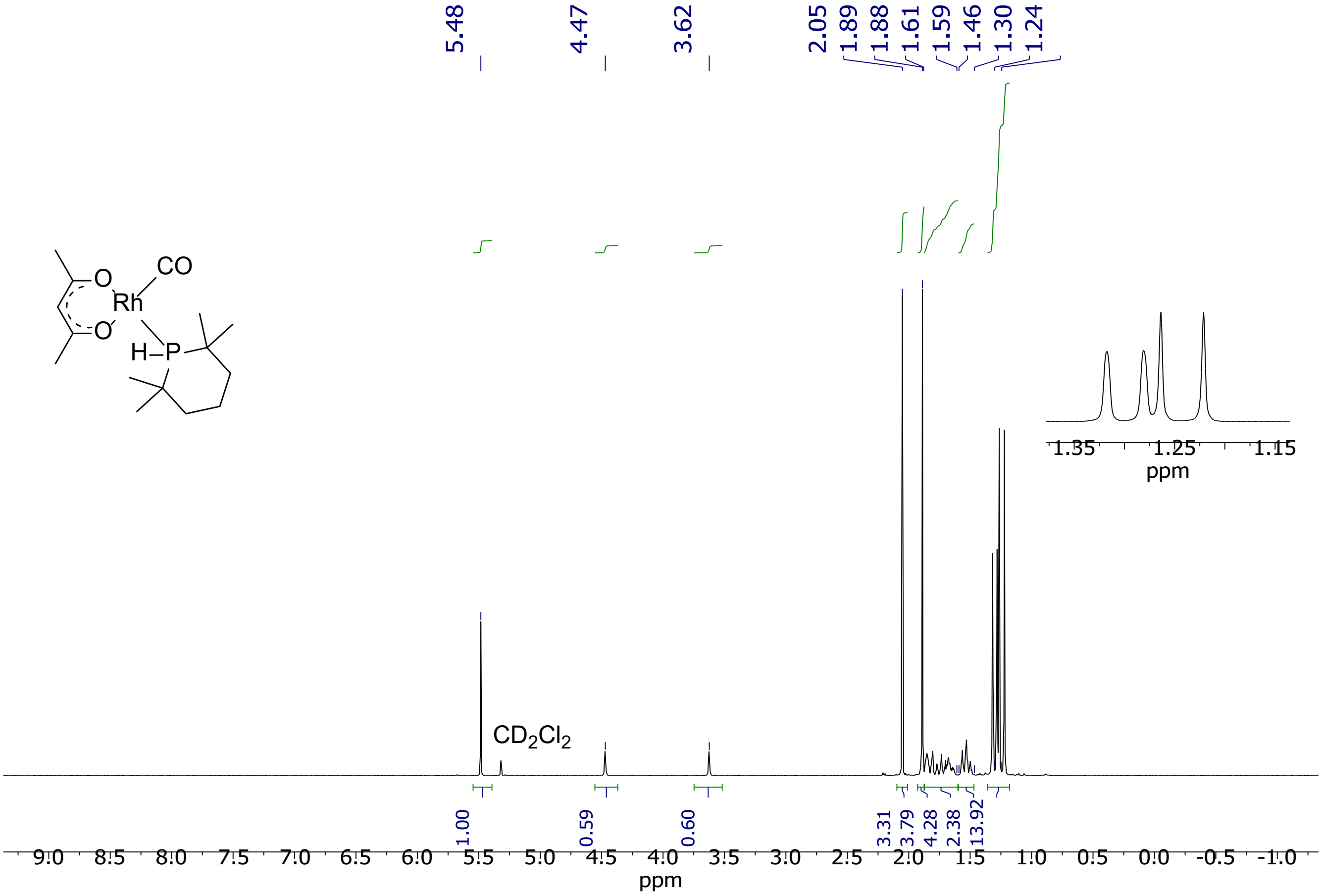

Supplementary Figure 76. ^{1}H NMR (400 MHz, $CDCl_3$) spectrum of [(acac)Rh(CO)(TMPhos)], compound **13**.

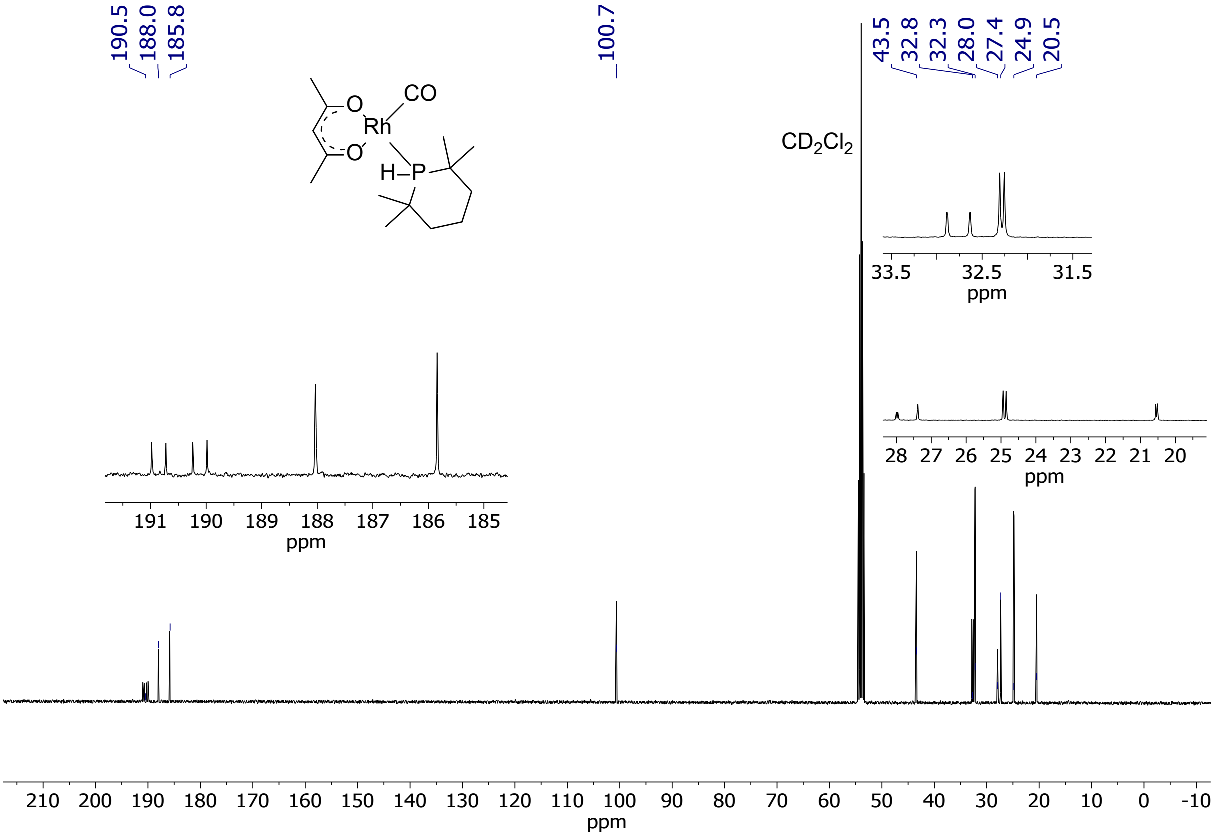

Supplementary Figure 77. $^{13}C\{^{1}H\}$ NMR (101 MHz, $CDCl_3$) spectrum of [(acac)Rh(CO)(TMPhos)], compound **13**.

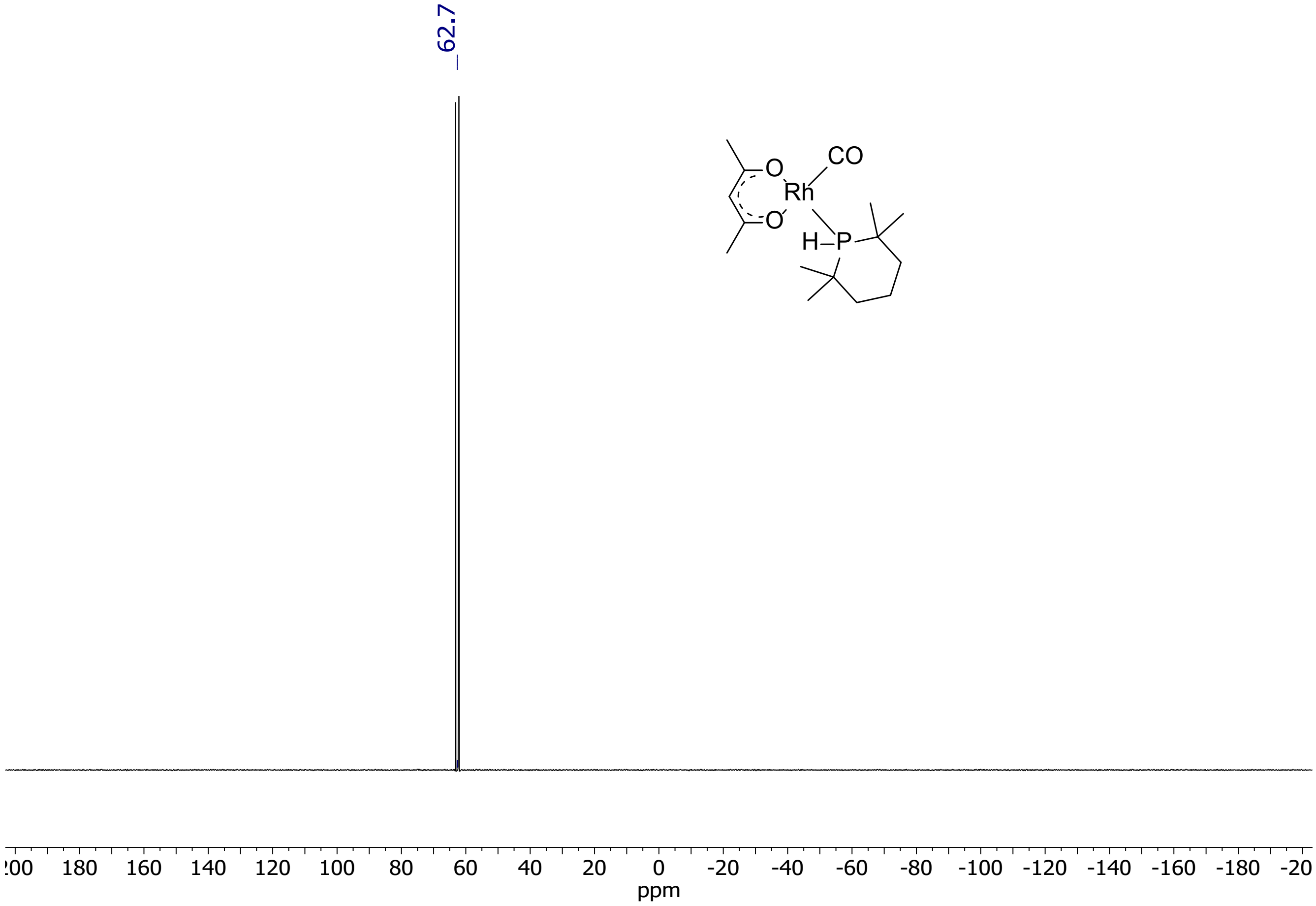

Supplementary Figure 78. $^{31}P\{^{1}H\}$ NMR (162 MHz, $CDCl_3$) spectrum of [(acac)Rh(CO)(TMPhos)], compound **13**.

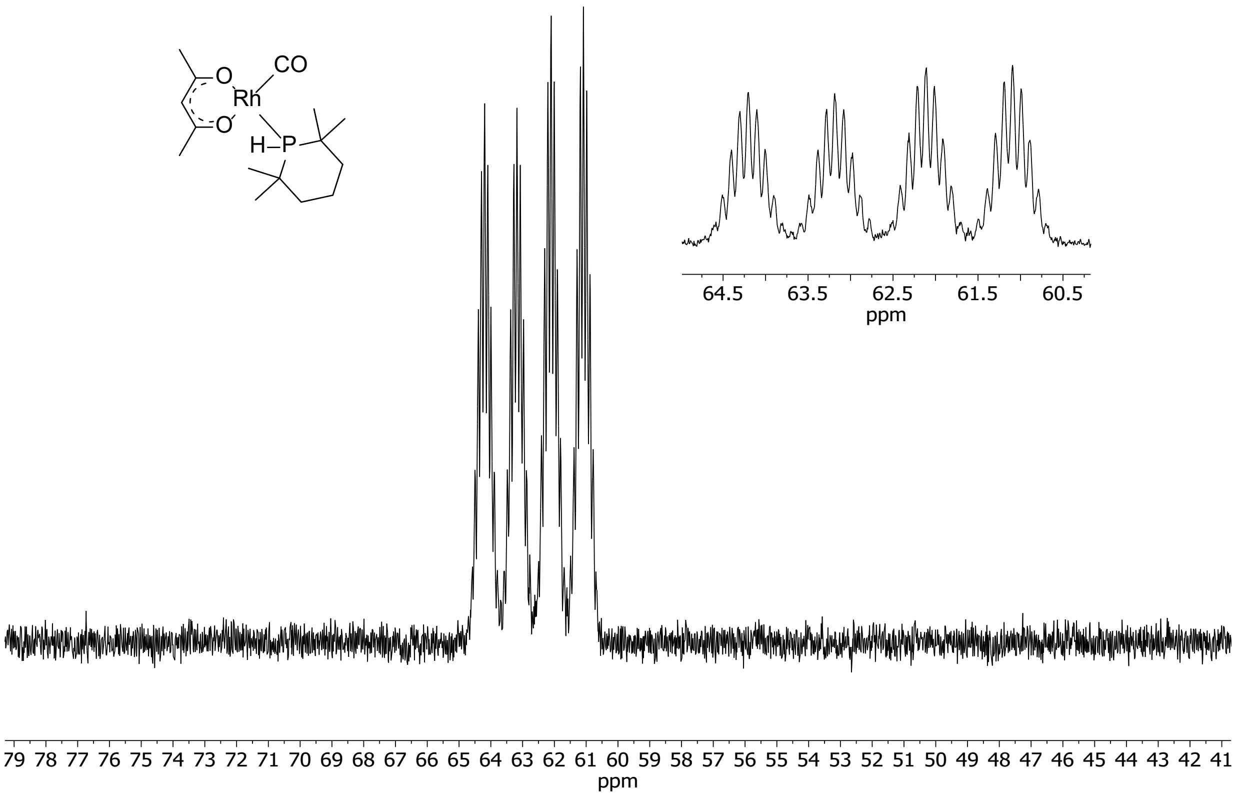

Supplementary Figure 79. ^{31}PNMR (162 MHz, $CDCl_3$) spectrum of [(acac)Rh(CO)(TMPhos)], compound **13**.

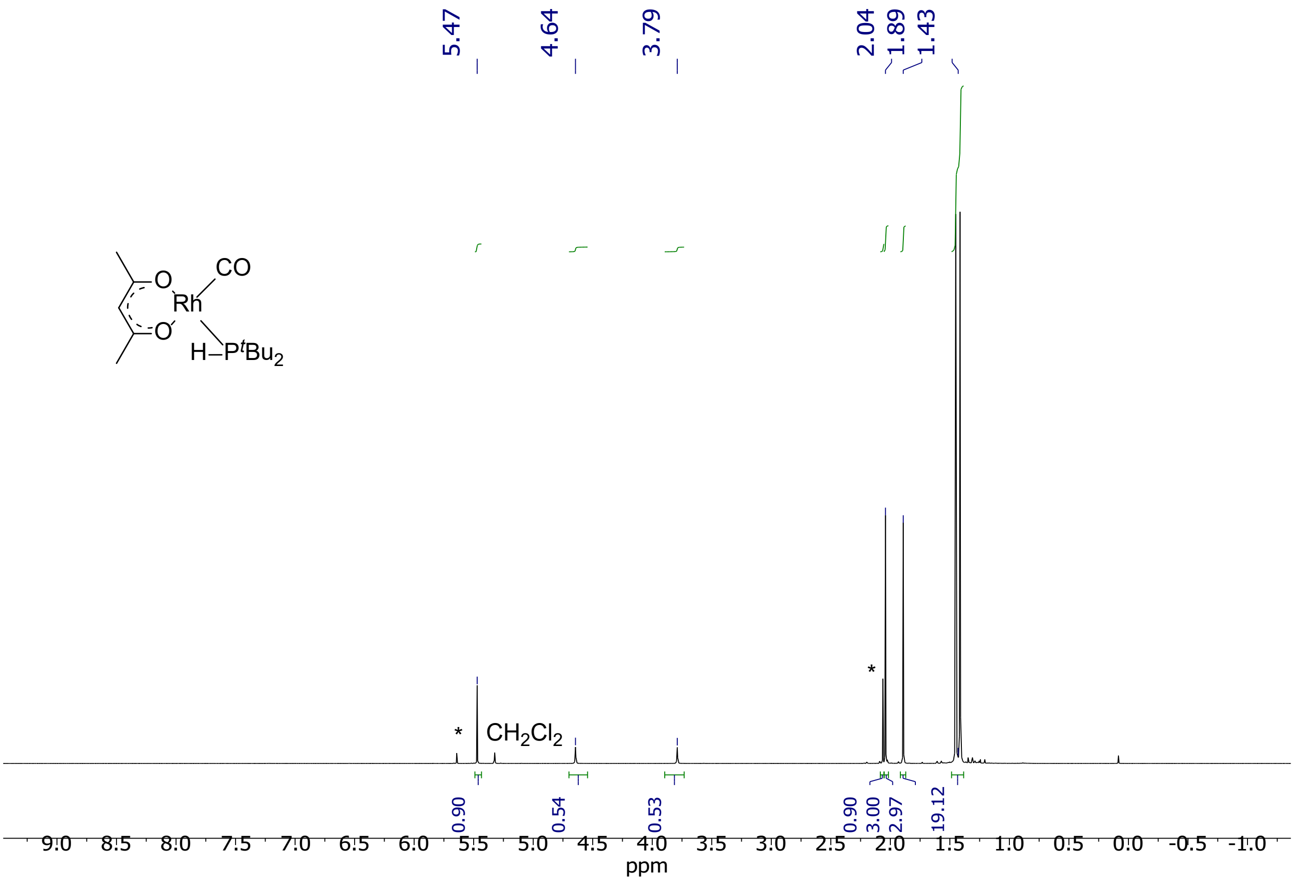

Supplementary Figure 80. ^{1}H NMR (400 MHz, $CDCl_3$) spectrum of [(acac)Rh(CO)(HP^{t}Bu$_2$)]. *[(acac)Rh(CO)$_2$] impurity

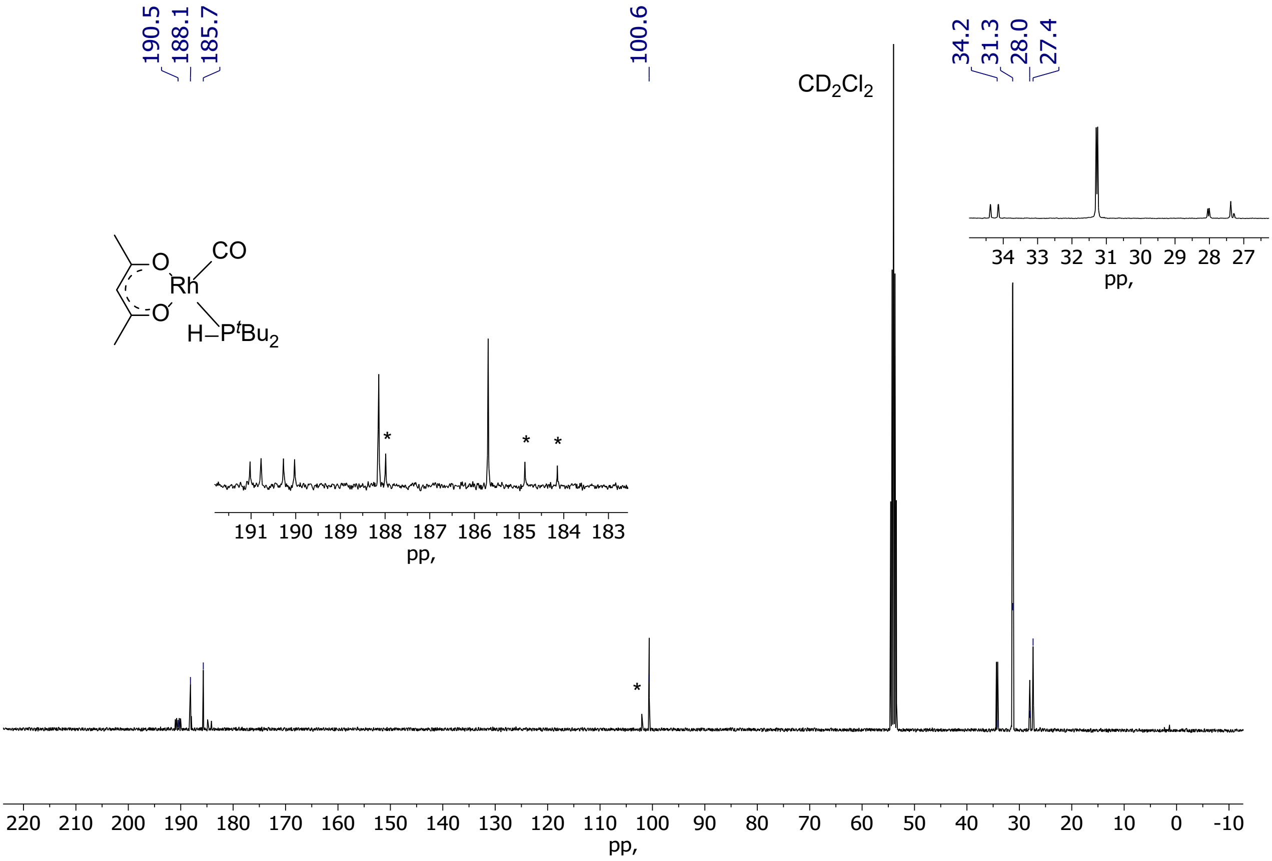

Supplementary Figure 81. $^{13}C\{^{1}H\}$ NMR (101 MHz, $CDCl_3$) spectrum of [(acac)Rh(CO)(HP^{t}Bu$_2$)].*[(acac)Rh(CO)$_2$] impurity

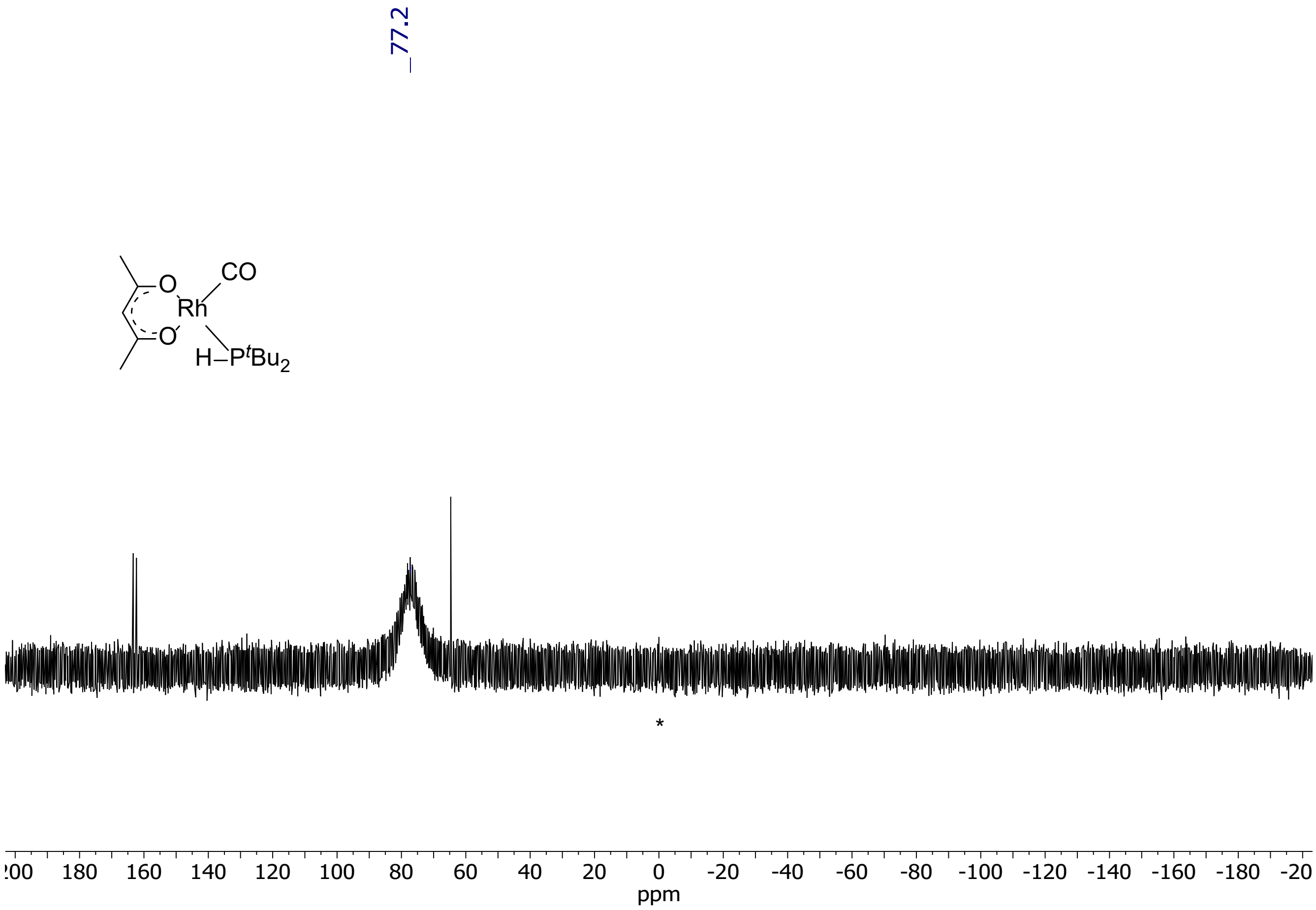

Supplementary Figure 82. $^{31}P\{^1H\}$ NMR (162 MHz, $CDCl_3$) spectrum of [(acac)Rh(CO)(HP^{t}Bu$_2$)].

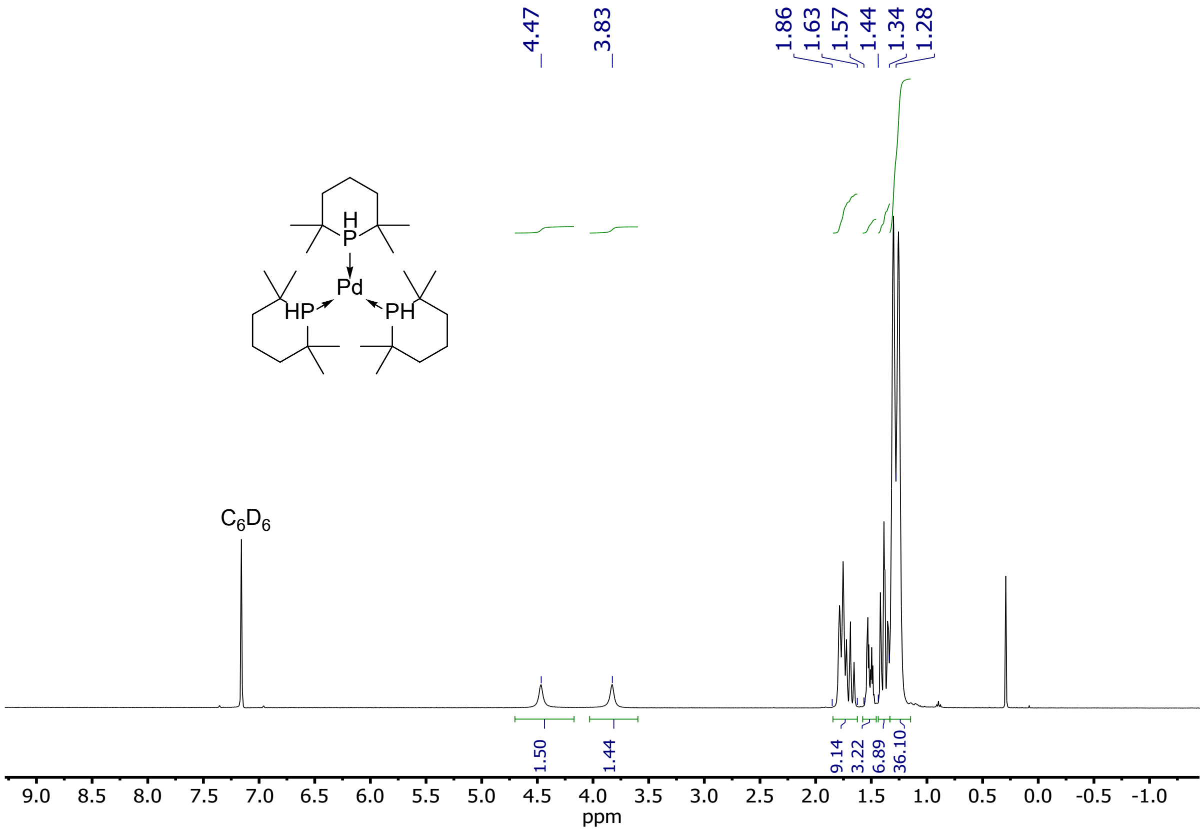

Supplementary Figure 83. ^1H NMR (400 MHz, C_6D_6) spectrum of [(**TMPhos**)$_3$Pd$^{(0)}$], compound **14**.

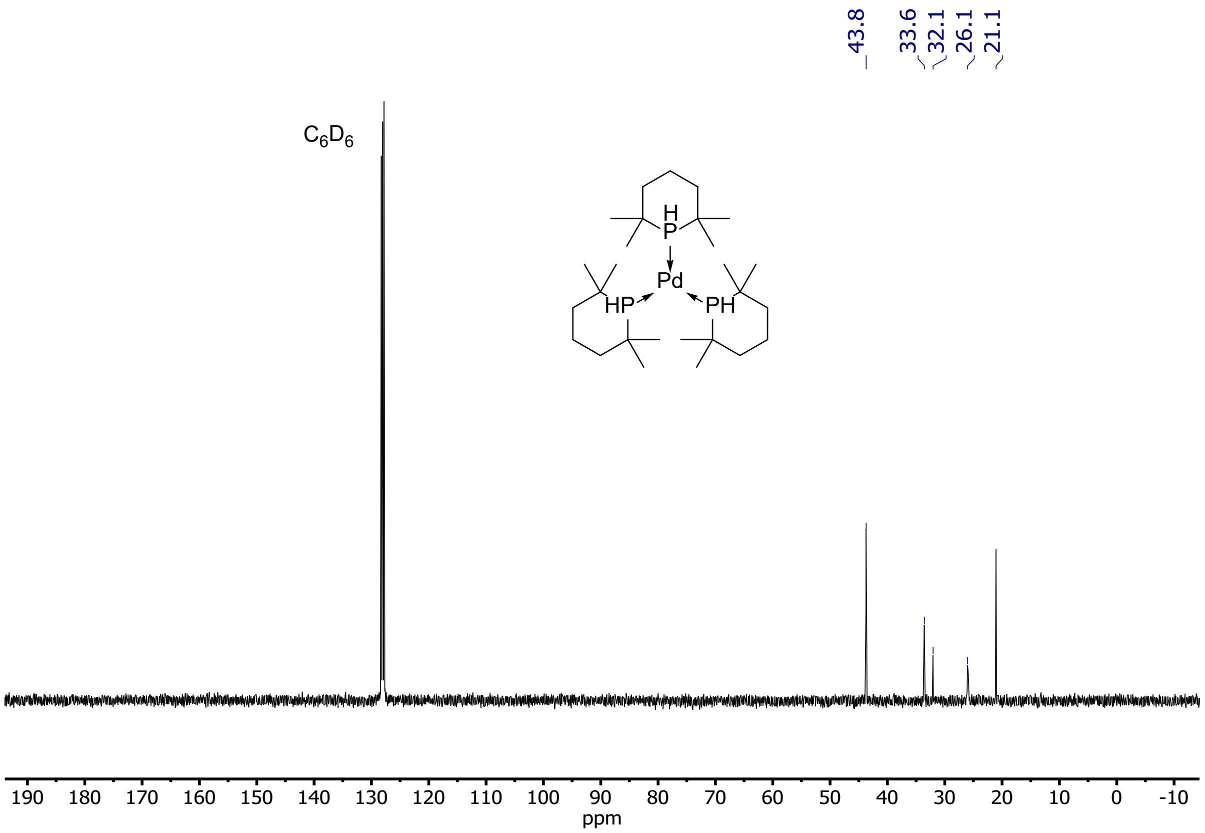

Supplementary Figure 84. $^{13}C\{^{1}H\}$ NMR (101 MHz, $CDCl_3$) spectrum of [(**TMPhos**)$_3$Pd$^{(0)}$], compound **14**.

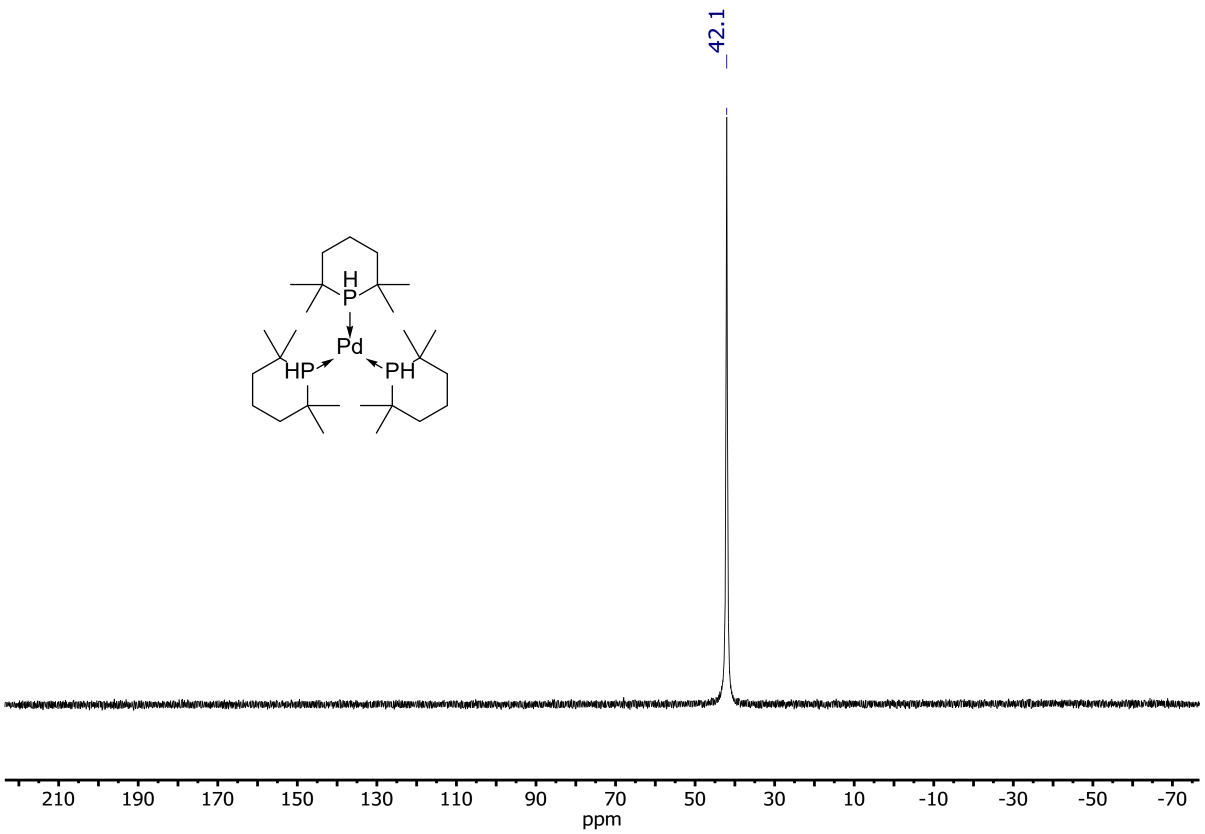

Supplementary Figure 85. $^{31}P\{^{1}H\}$ NMR (162 MHz, C_6D_6) spectrum of [(**TMPhos**)$_3$Pd$^{(0)}$], compound **14**.

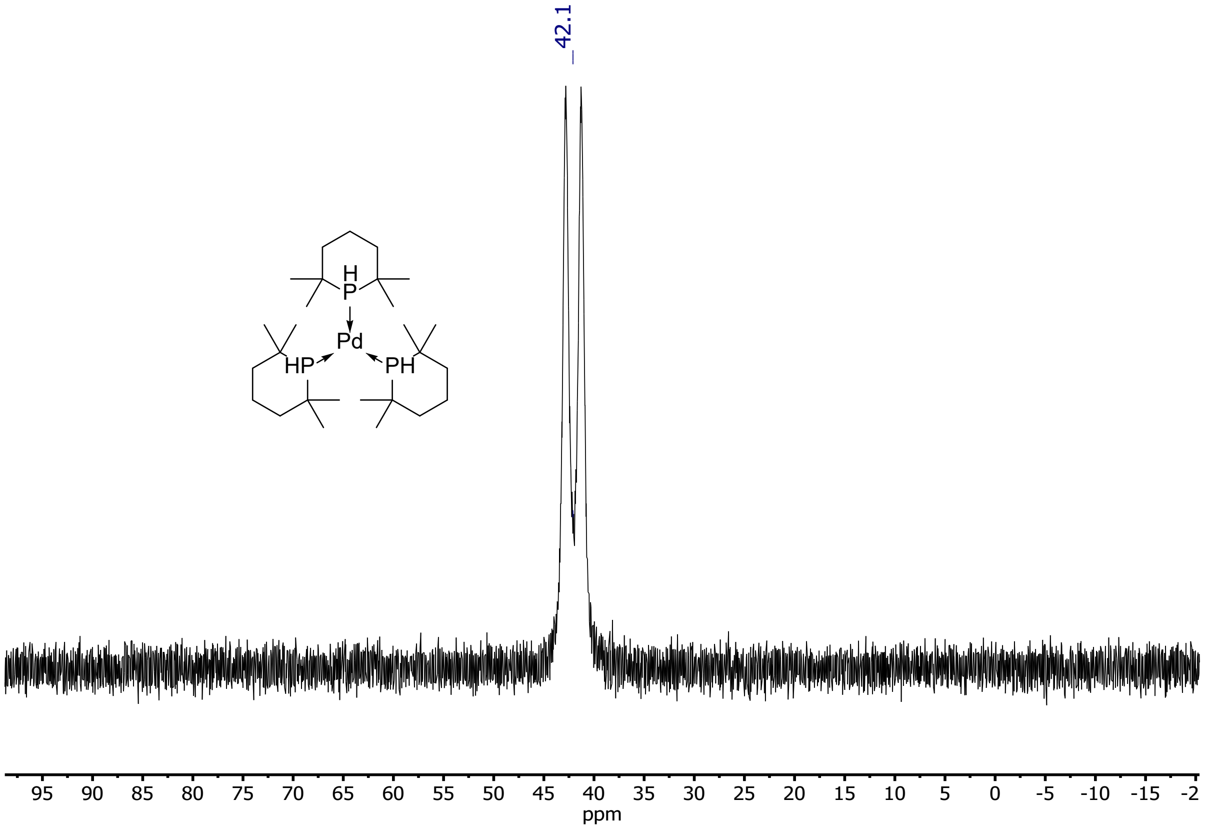

Supplementary Figure 86. ^{31}P NMR (162 MHz, C_6D_6) spectrum of [(**TMPhos**)$_3$Pd$^{(0)}$], compound **14**.

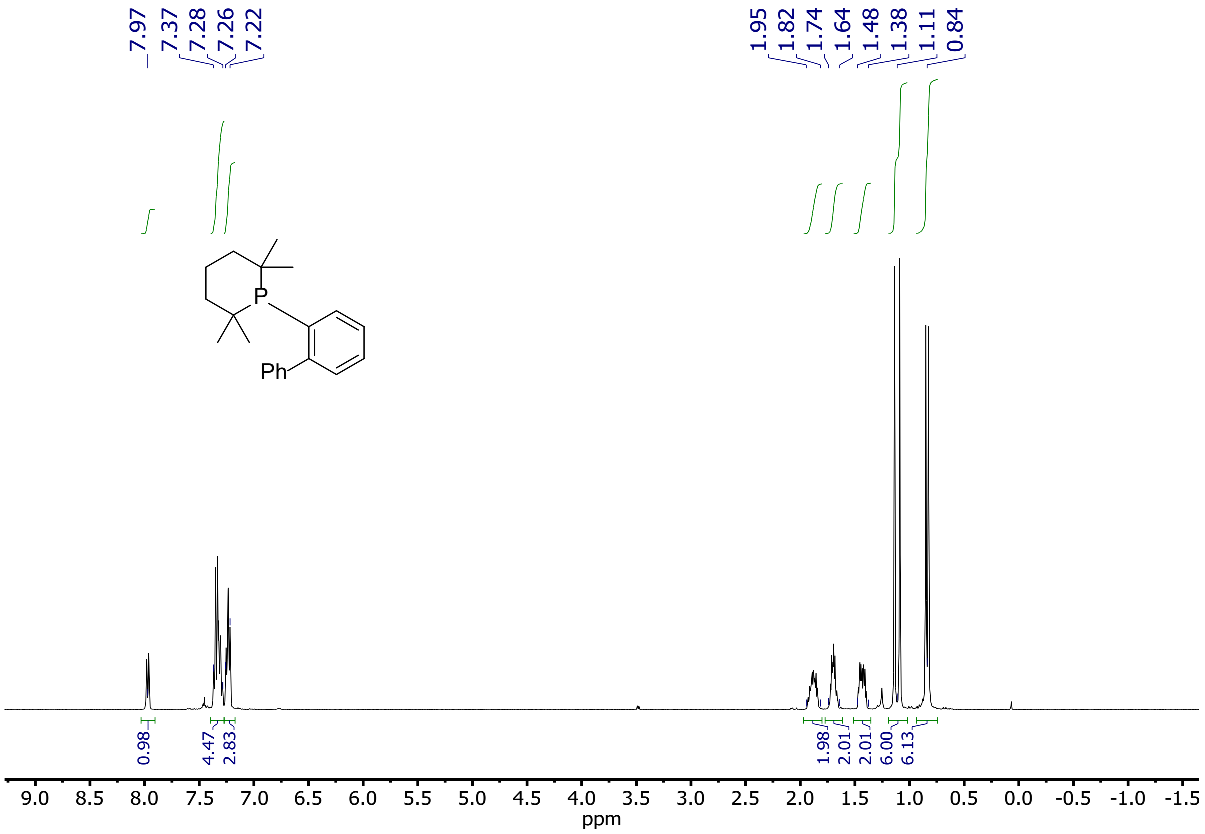

Supplementary Figure 87. ^{1}H NMR (400 MHz, $CDCl_3$) spectrum of 1-([1,1'-biphenyl]-2-yl)-2,2,6,6-tetramethylphosphinane, ^{TMPhos}**(Biphenyl)**, compound **16**.

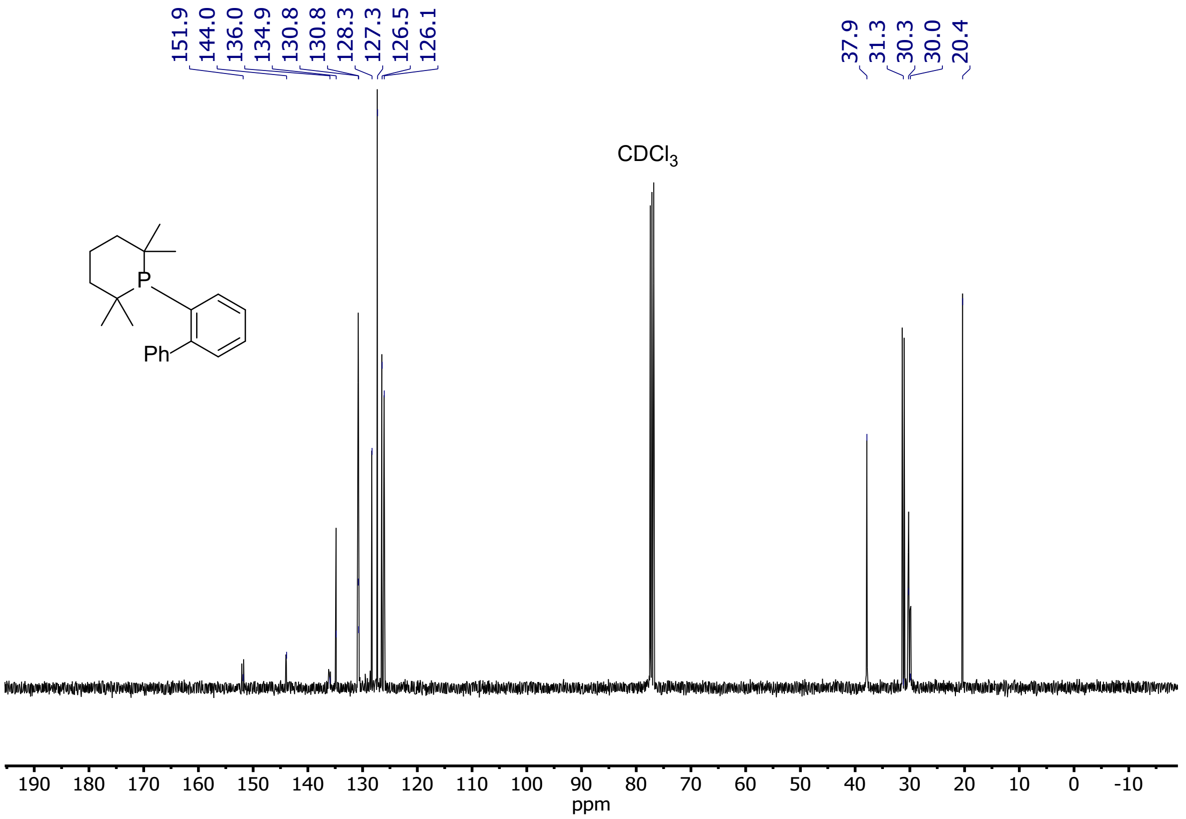

Supplementary Figure 88. $^{13}C\{^1H\}$ NMR (101 MHz, $CDCl_3$) spectrum of 1-([1,1'-biphenyl]-2-yl)-2,2,6,6-tetramethylphosphinane, **TMPhos(Biphenyl)**, compound **16**.

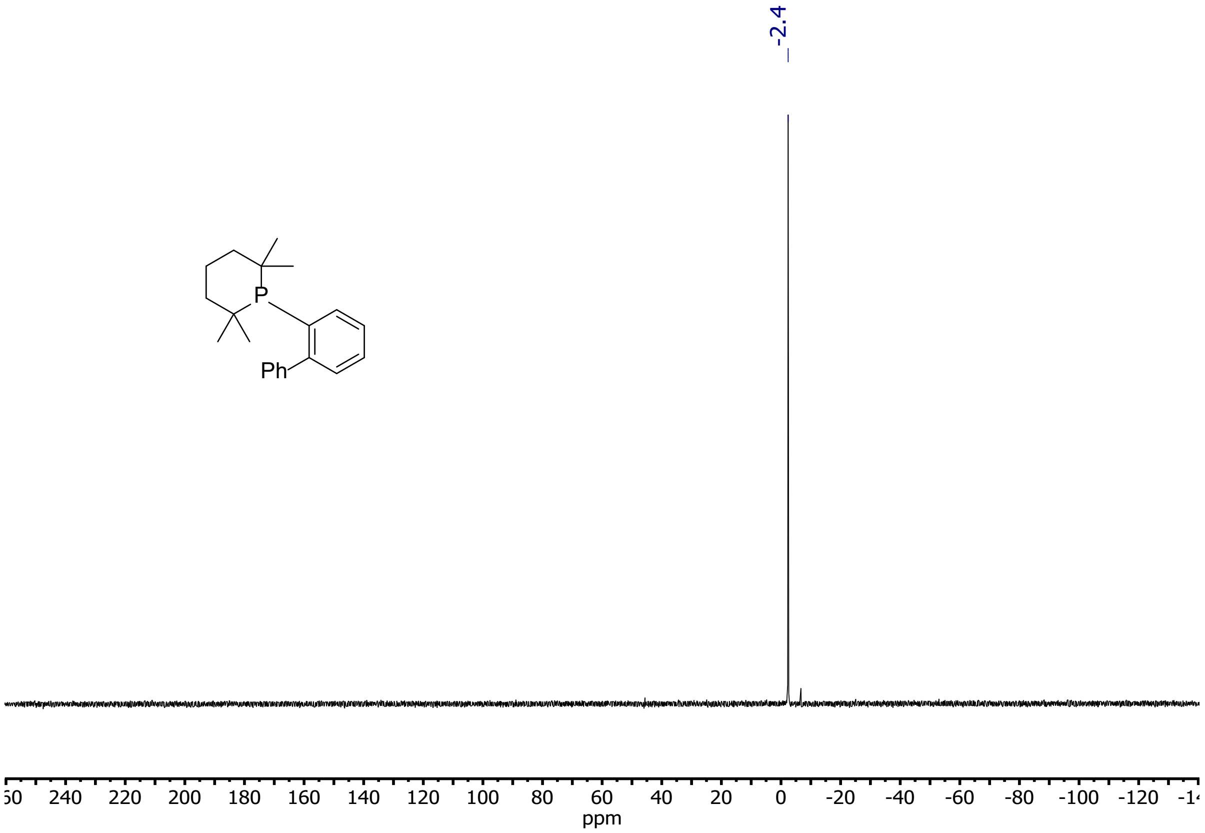

Supplementary Figure 89. $^{31}P\{^1H\}$ NMR (162 MHz, $CDCl_3$) spectrum of 1-([1,1'-biphenyl]-2-yl)-2,2,6,6-tetramethylphosphinane, **TMPhos(Biphenyl)**, compound **16**.

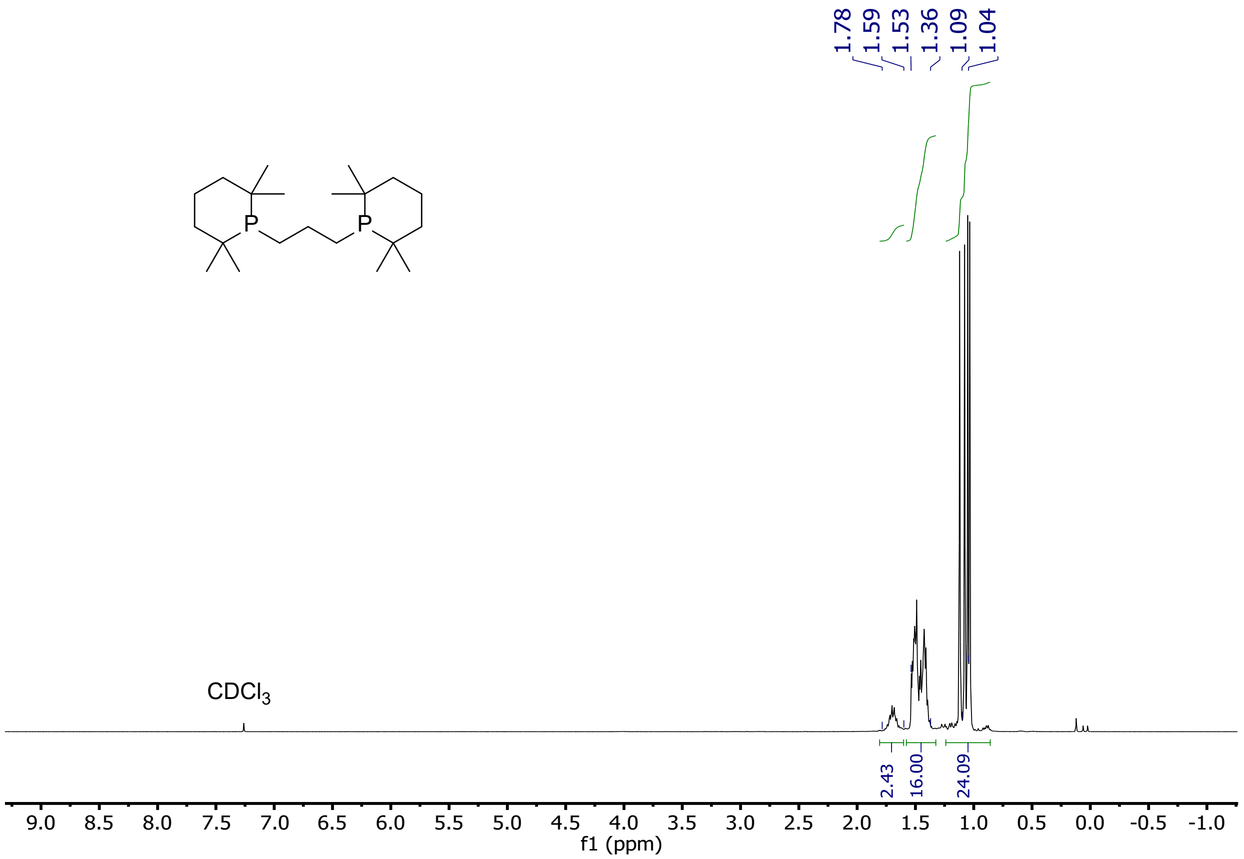

Supplementary Figure 90. ^{1}H NMR (400 MHz, $CDCl_3$) spectrum of 1,3-bis(2,2,6,6-tetramethylphosphinan-1-yl)propane, **BTMPPr**, compound **17**.

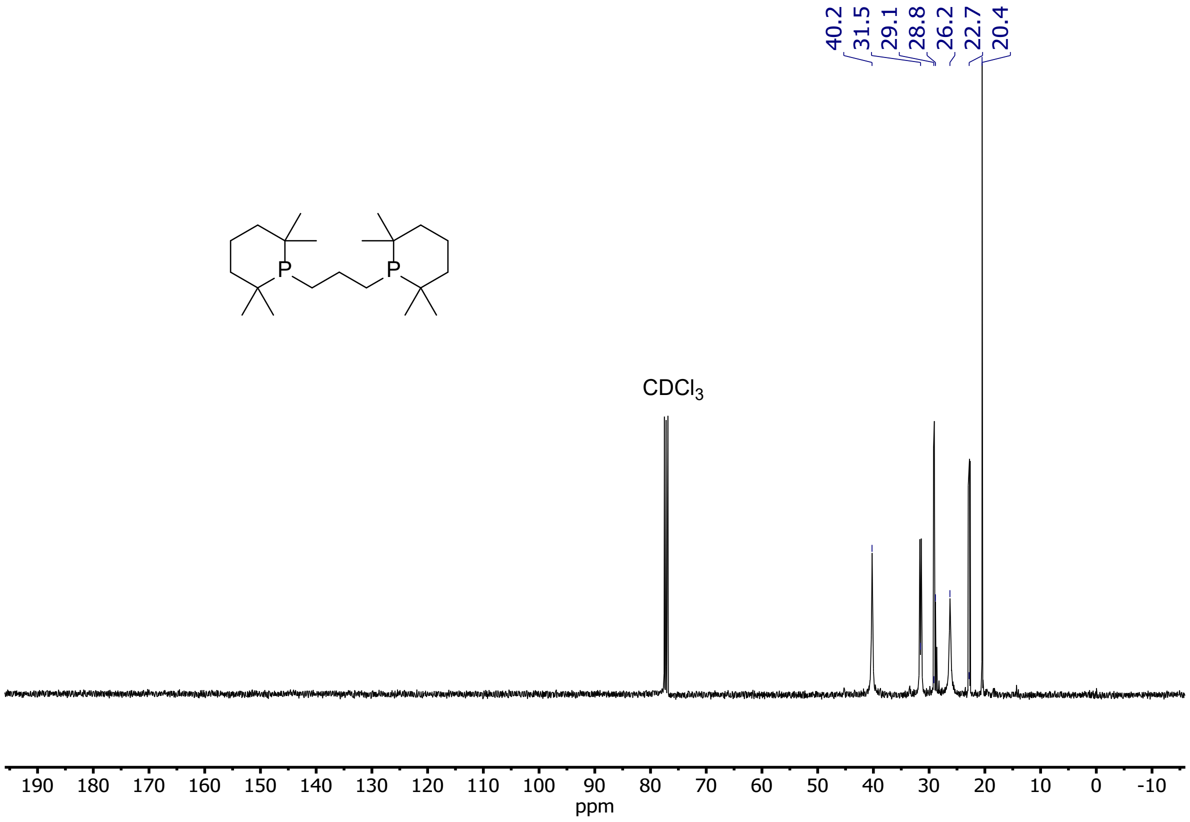

Supplementary Figure 91. $^{13}C\{^{1}H\}$ NMR (101 MHz, $CDCl_3$) spectrum of 1,3-bis(2,2,6,6-tetramethylphosphinan-1-yl)propane, **BTMPPr**, compound **17**.

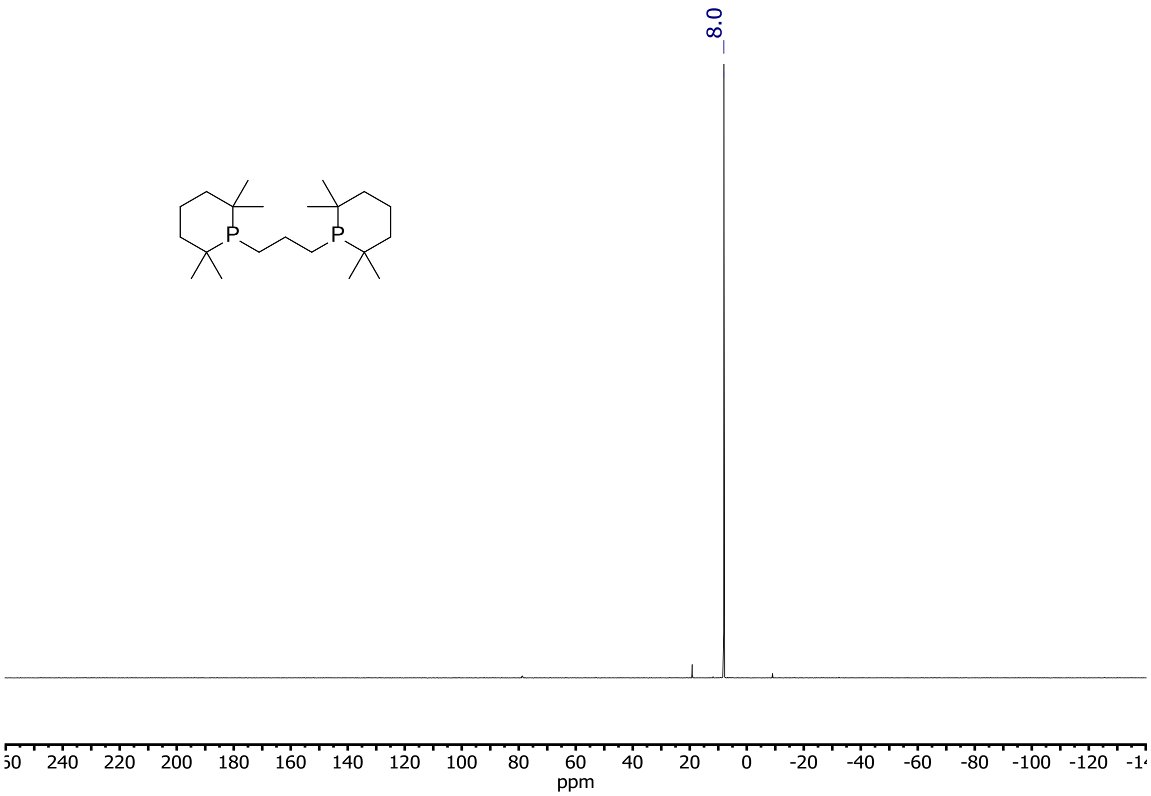

Supplementary Figure 92. $^{31}P\{^{1}H\}$ NMR (162 MHz, $CDCl_3$) spectrum of 1,3-bis(2,2,6,6-tetramethylphosphinan-1-yl)propane, **BTMPPr**, compound **17**.

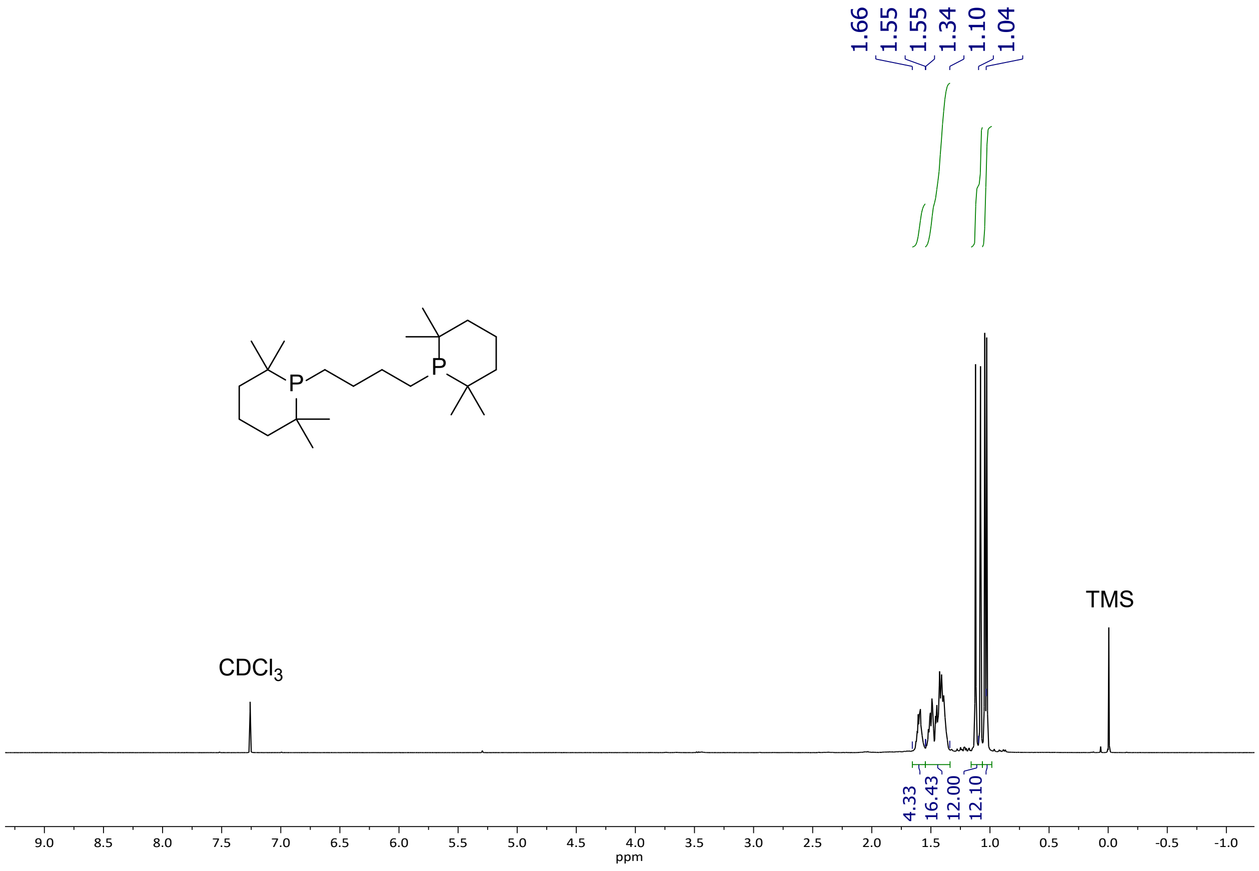

Supplementary Figure 93. ^{1}H NMR (400 MHz, $CDCl_3$) spectrum of 1,4-Bis(2,2,6,6-tetramethylphosphinan-1-yl)butane, **BTMPBu**, compound **18**.

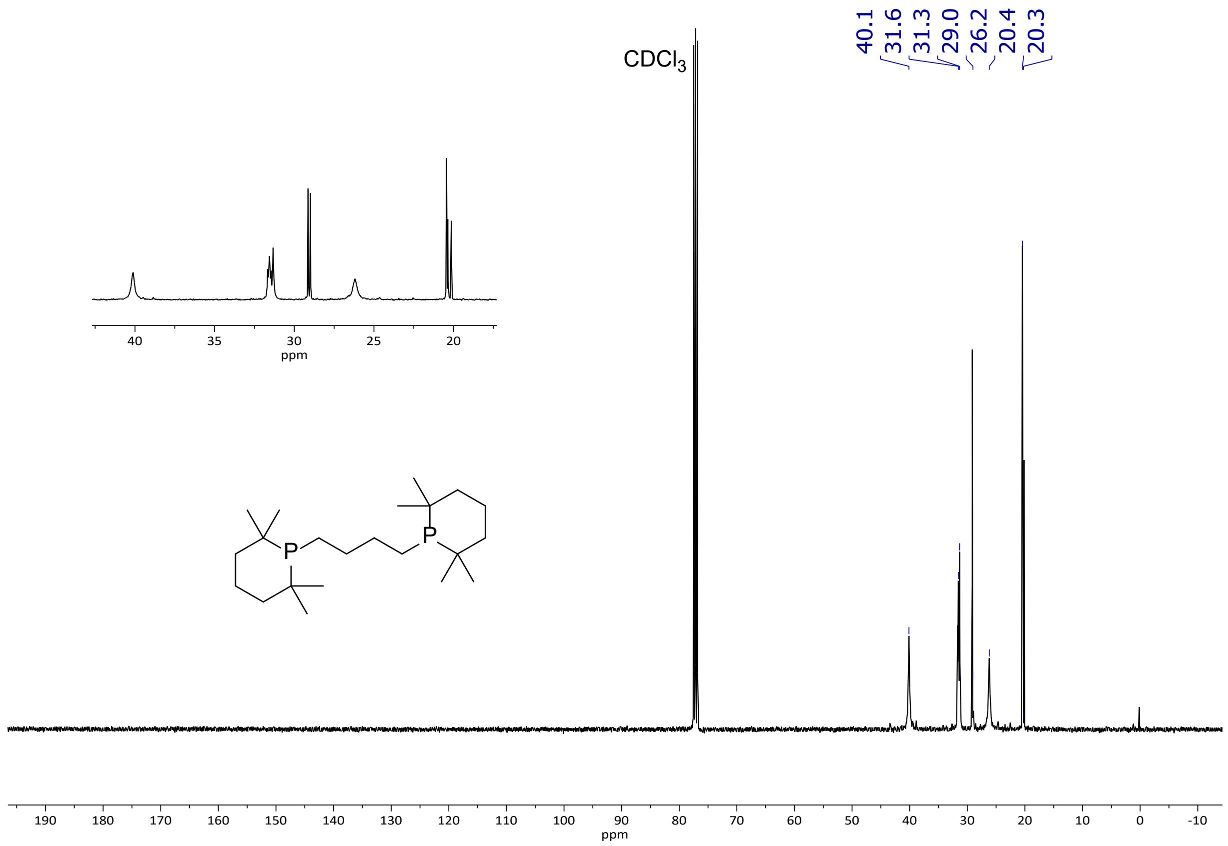

Supplementary Figure 94. $^{13}C\{^{1}H\}$ NMR (101 MHz, $CDCl_3$) spectrum of 1,4-Bis(2,2,6,6-tetramethylphosphinan-1-yl)butane, **BTMPBu**, compound **18**.

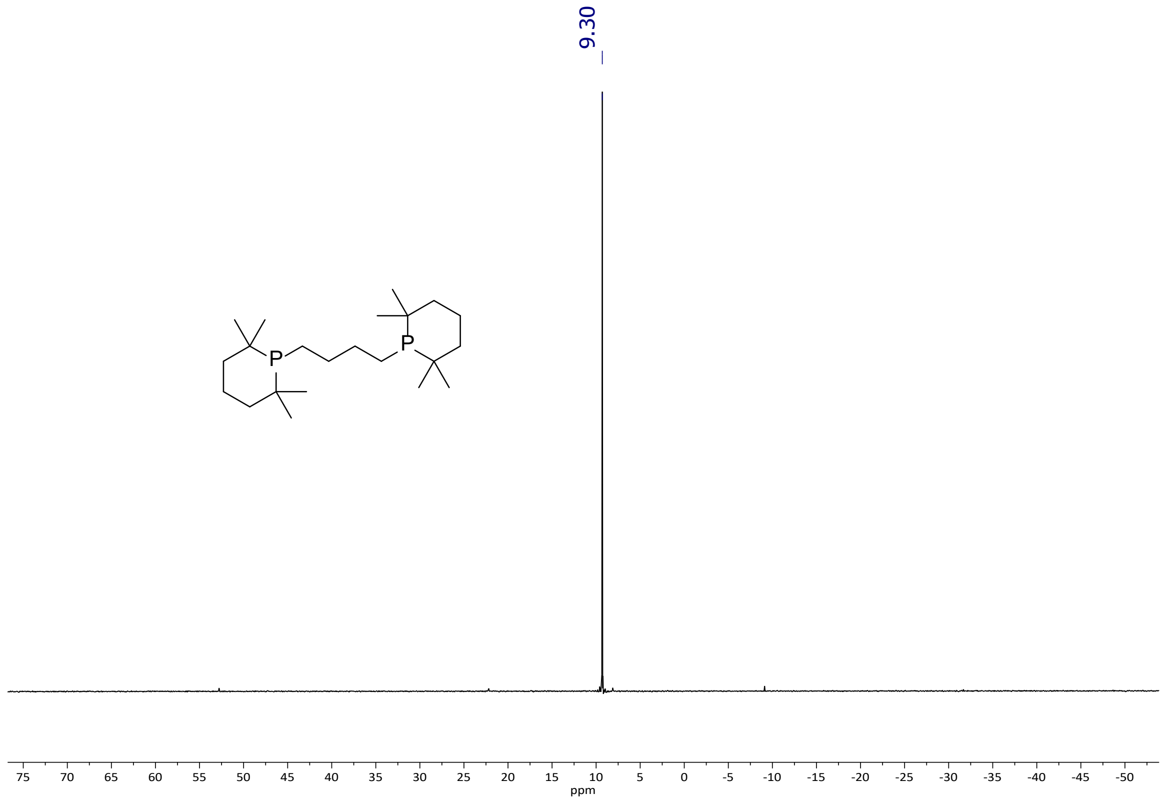

Supplementary Figure 95. $^{31}P\{^{1}H\}$ NMR (162 MHz, $CDCl_3$) spectrum of 1,4-Bis(2,2,6,6-tetramethylphosphinan-1-yl)butane, **BTMPBu**, compound **18**.

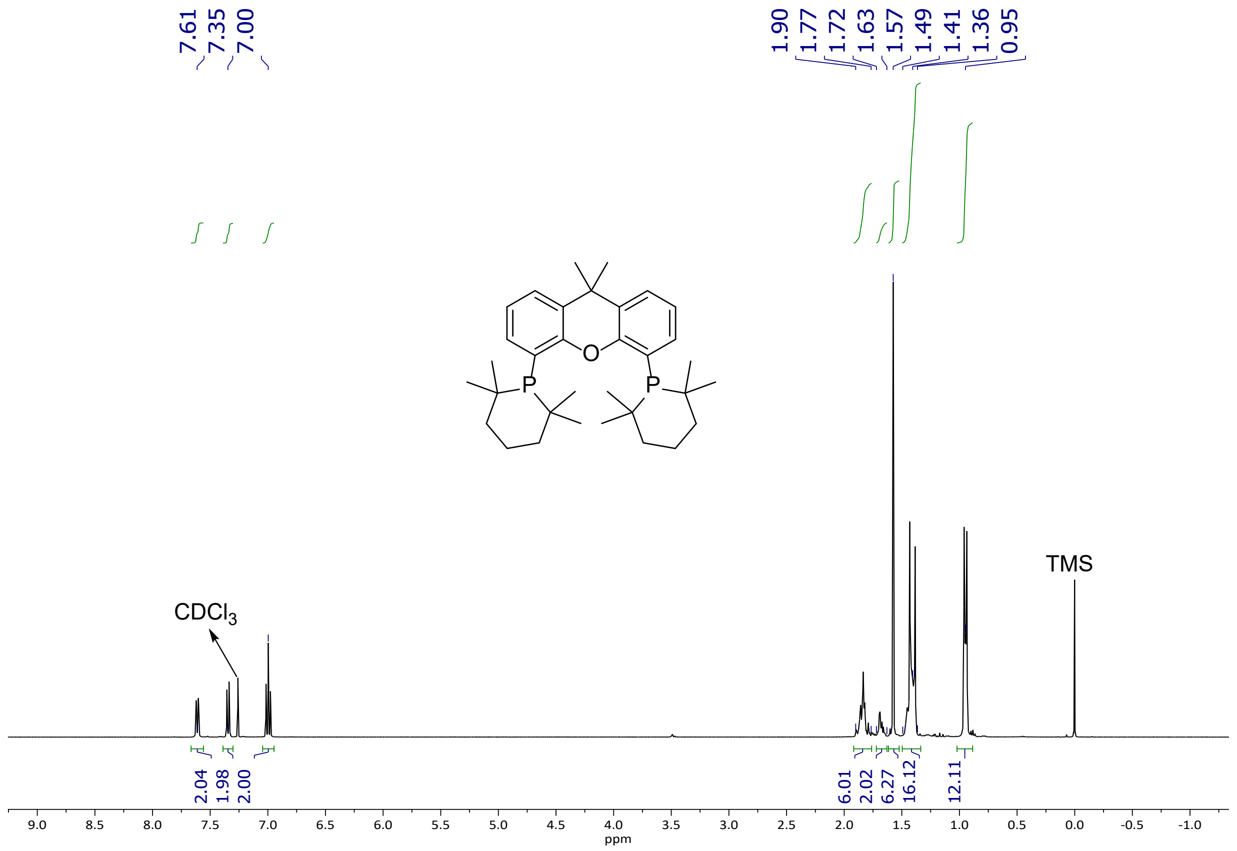

Supplementary Figure 96. ^{1}H NMR (400 MHz, $CDCl_3$) spectrum of **^{TMPhos}(Xantphos)**, compound **19**.

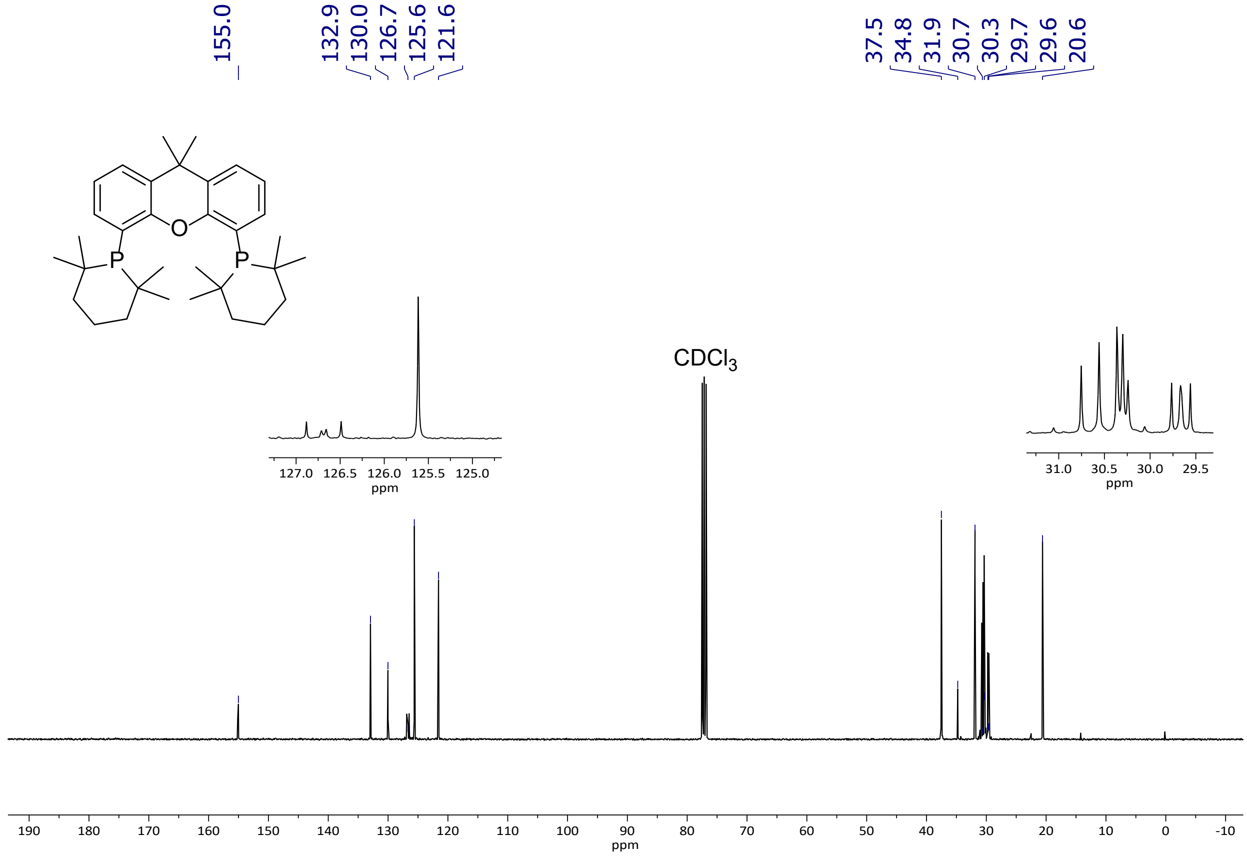

Supplementary Figure 97. $^{13}C\{^{1}H\}$ NMR (101 MHz, $CDCl_3$) spectrum of **^{TMPhos}(Xantphos)**, compound **19.**

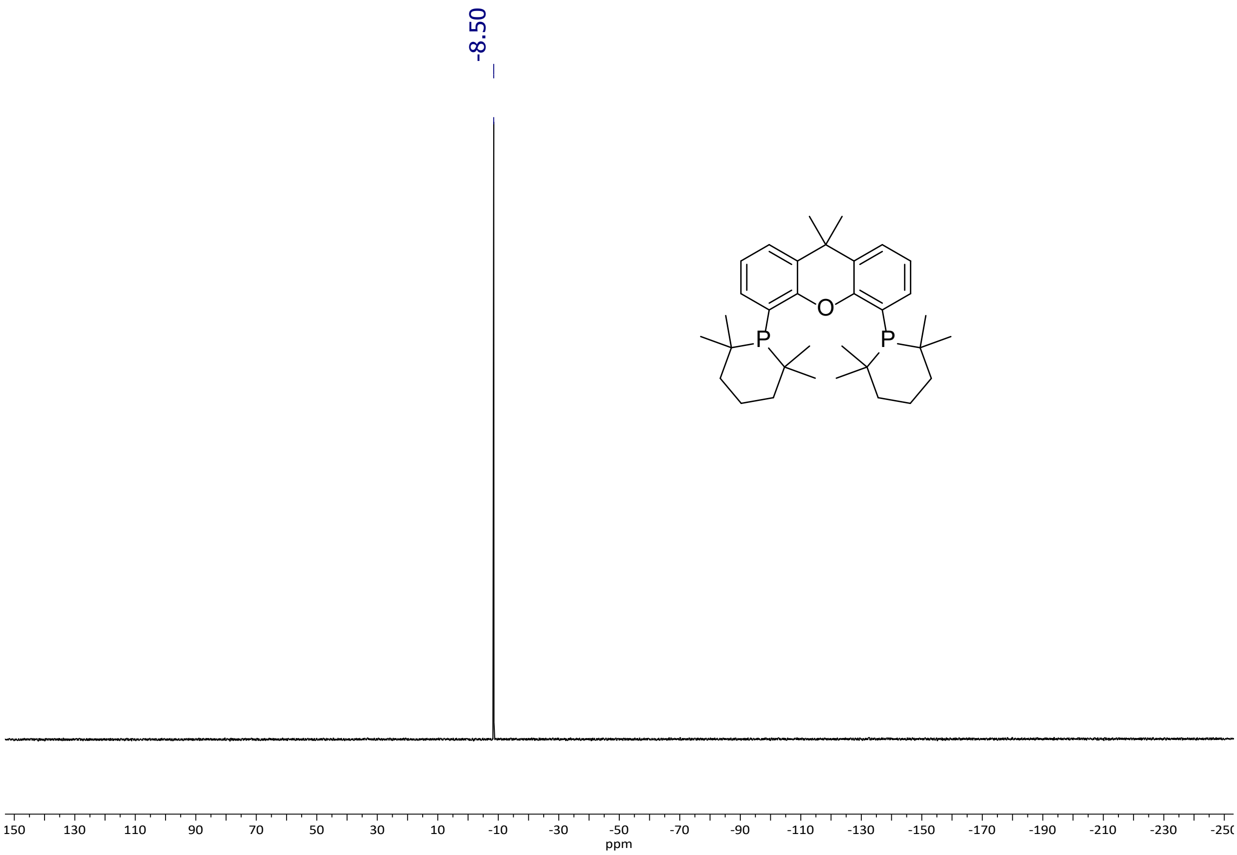

Supplementary Figure 98. $^{31}P\{^1H\}$ NMR (162 MHz, $CDCl_3$) spectrum of **TMPhos(Xantphos)**, compound **19**.

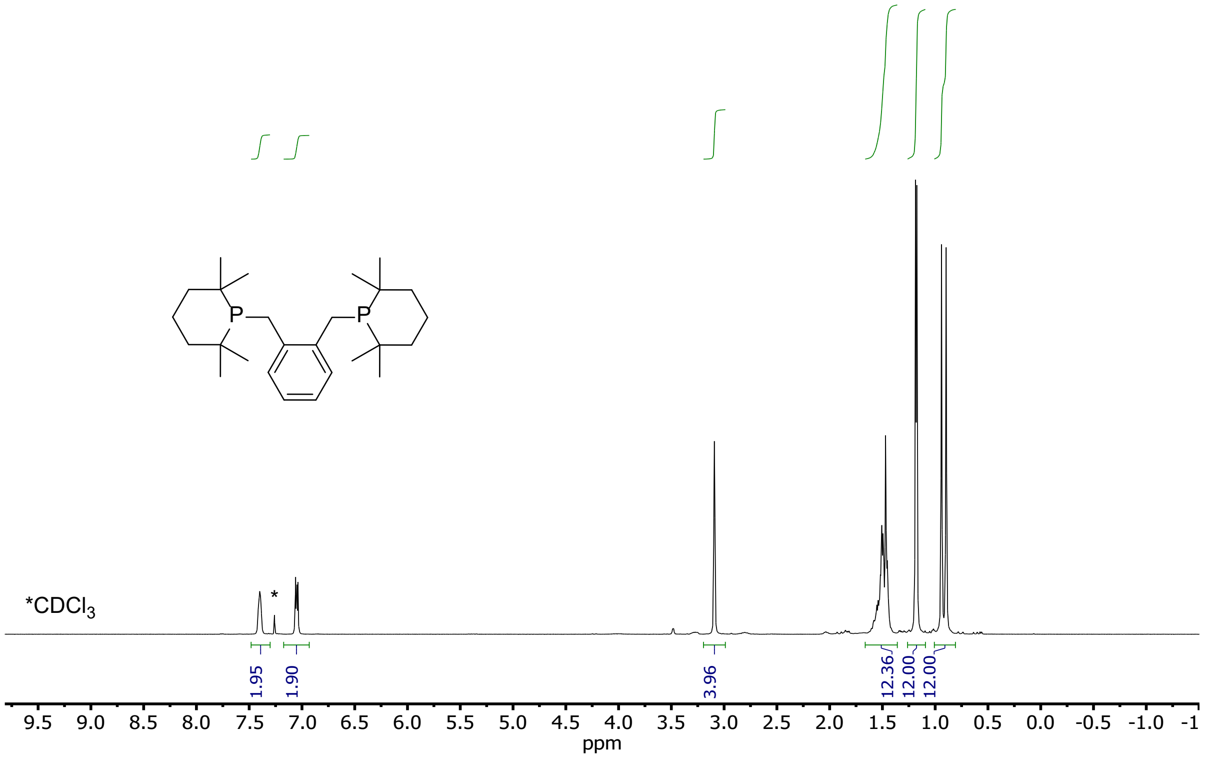

Supplementary Figure 99. ^1H NMR (400 MHz, $CDCl_3$) spectrum of 1,2-bis((2,2,6,6-tetramethylphosphinan-1-yl)methyl)benzene, **BTMPX**, compound **20**.

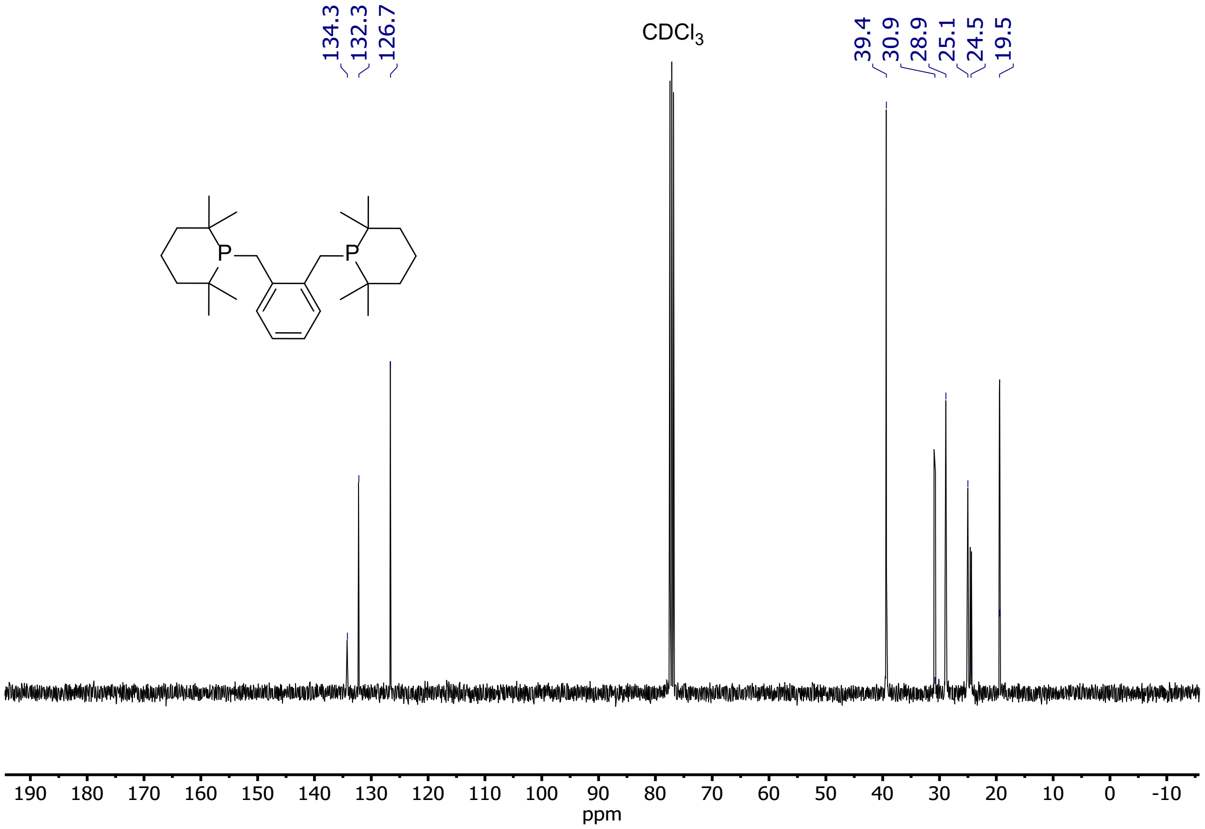

Supplementary Figure 100. $^{13}C\{^1H\}$ NMR (101 MHz, $CDCl_3$) spectrum of 1,2-bis((2,2,6,6-tetramethylphosphinan-1-yl)methyl)benzene, **BTMPX**, compound **20**.

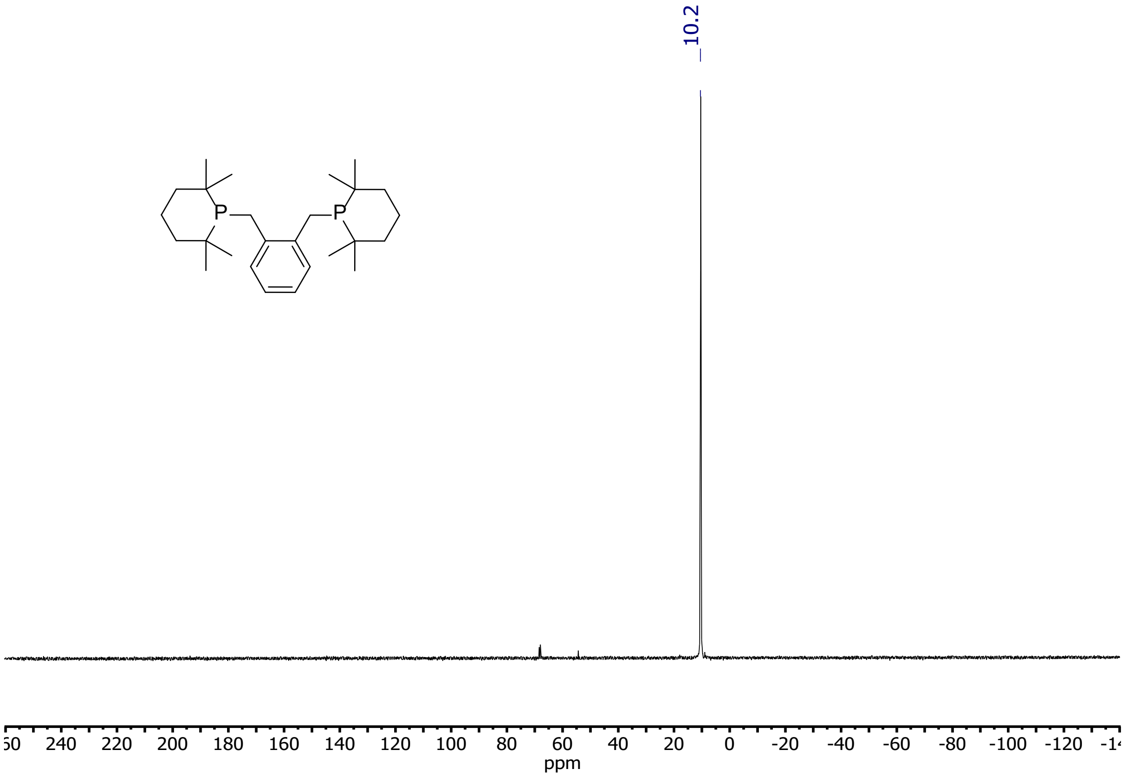

Supplementary Figure 101. $^{31}P\{^1H\}$ NMR (162 MHz, $CDCl_3$) spectrum of 1,2-bis((2,2,6,6-tetramethylphosphinan-1-yl)methyl)benzene, **BTMPX**, compound **20**.

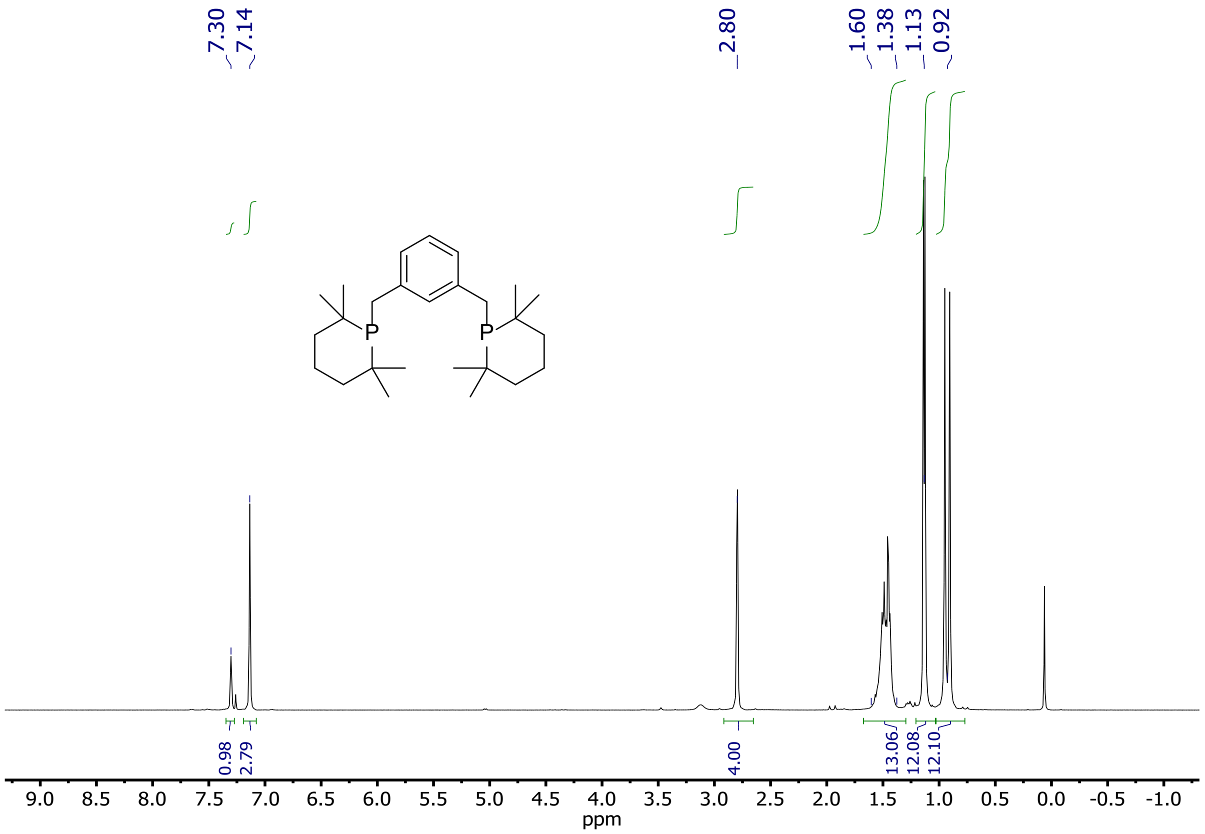

Supplementary Figure 102. ^{1}H NMR (400 MHz, $CDCl_3$) spectrum of 1,3-bis((2,2,6,6-tetramethylphosphinan-1-yl)methyl)benzene, $^{\mathbf{TMPhos}}$**(PCP)**, compound **21**.

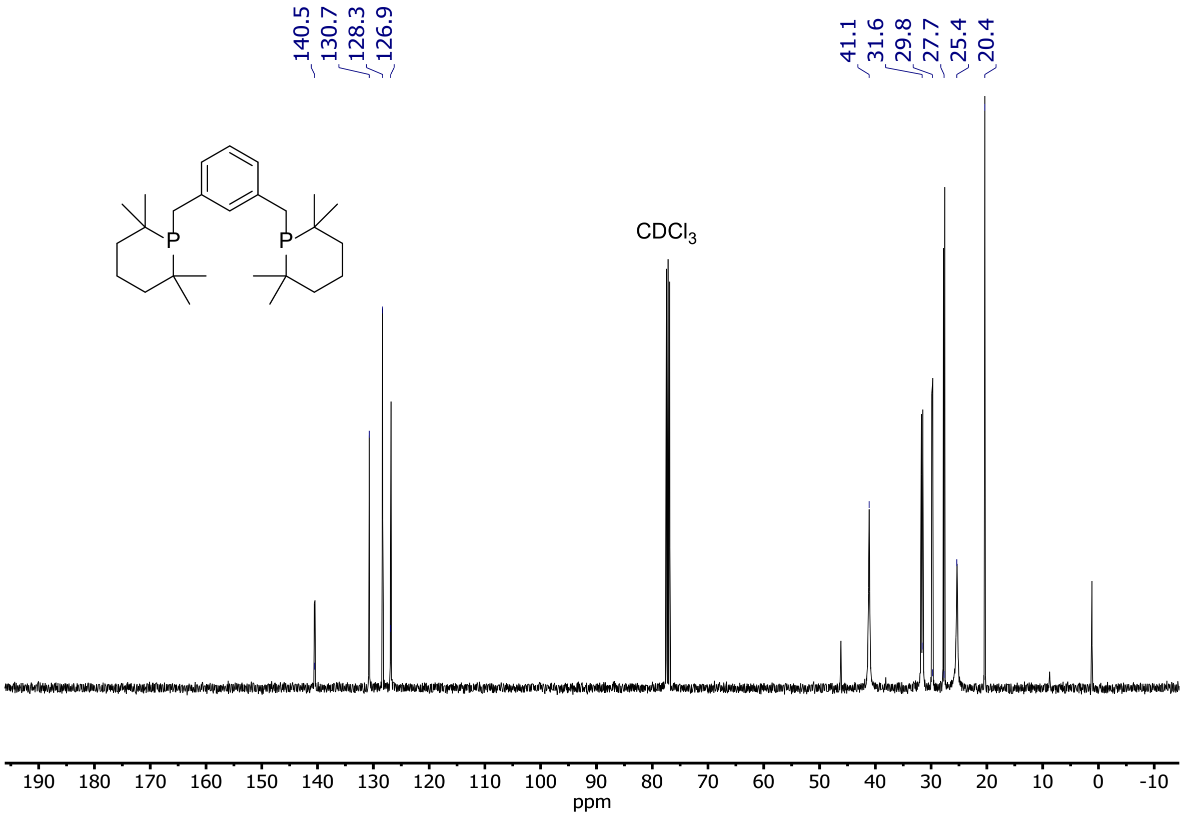

Supplementary Figure 103. ^{13}C{^{1}H} NMR (101 MHz, $CDCl_3$) spectrum of 1,3-bis((2,2,6,6-tetramethylphosphinan-1-yl)methyl)benzene, $^{\mathbf{TMPhos}}$**(PCP)**, compound **21**.

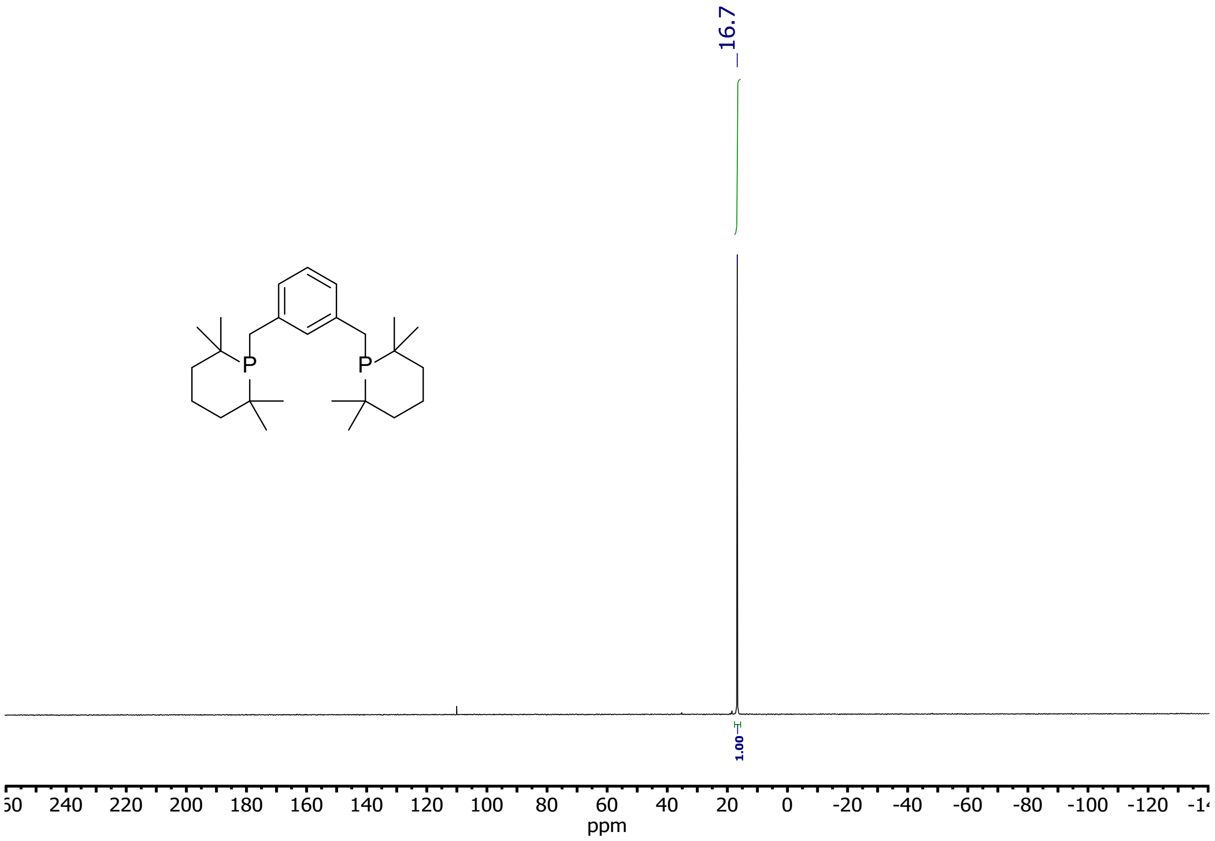

Supplementary Figure 104. ^{31}P{^{1}H} NMR (162 MHz, $CDCl_3$) spectrum of 1,3-bis((2,2,6,6-tetramethylphosphinan-1-yl)methyl)benzene, **^{TMPhos}(PCP)**, compound **21**.

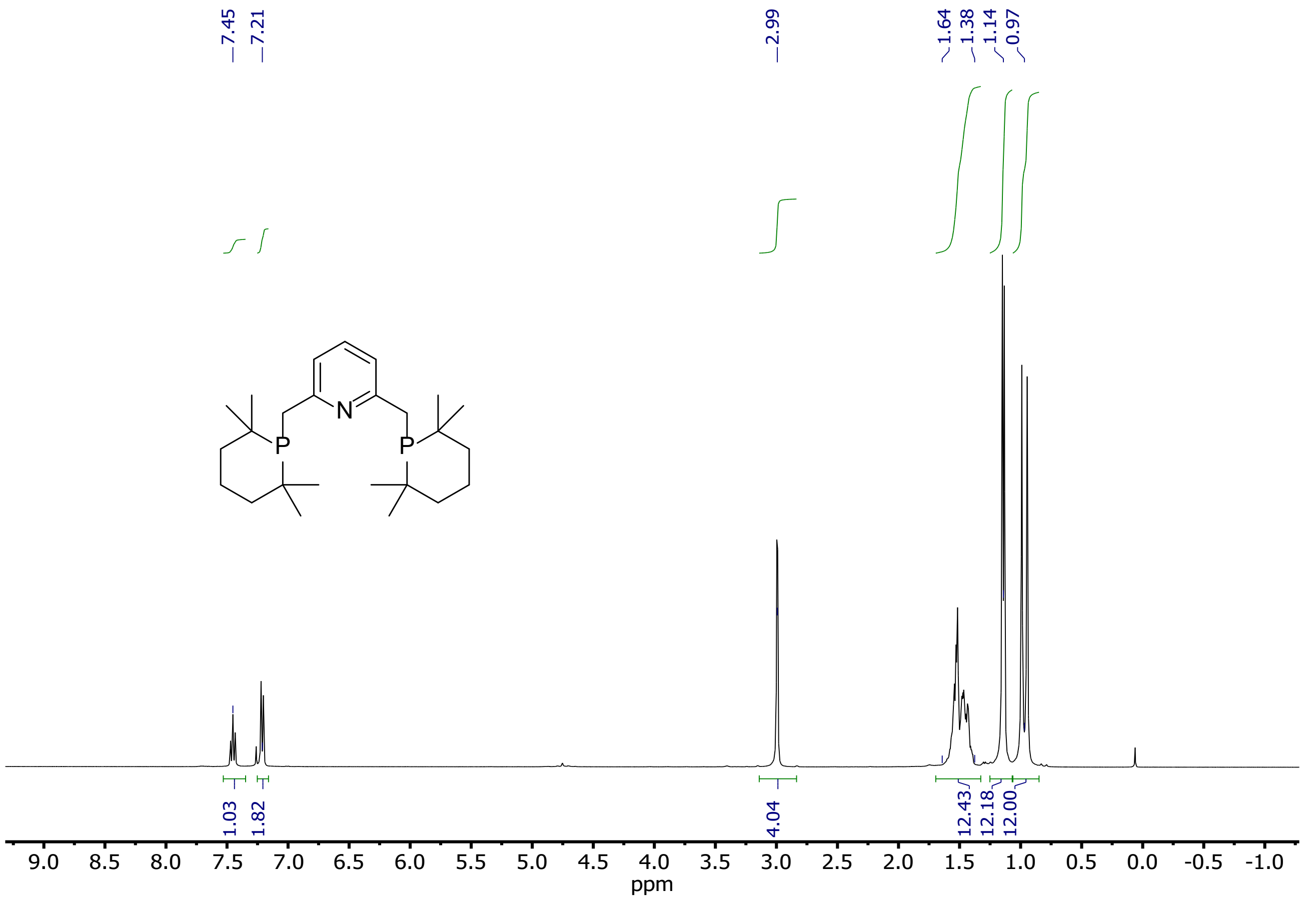

Supplementary Figure 105. ^{1}H NMR (400 MHz, $CDCl_3$) spectrum of 2,6-Bis((2,2,6,6-tetramethylphosphinan-1-yl)methyl)pyridine, **^{TMPhos}(PNP)**, compound **22**.

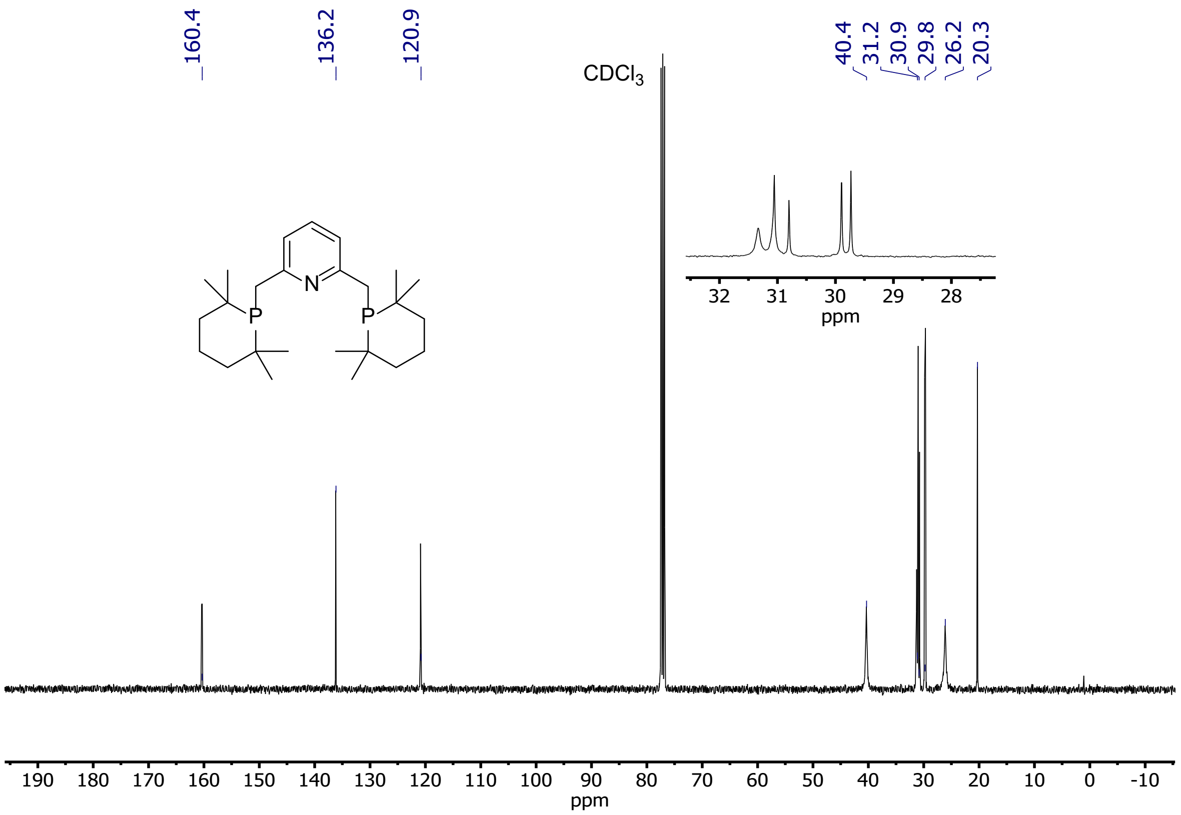

Supplementary Figure 106. $^{13}C\{^1H\}$ NMR (101 MHz, $CDCl_3$) spectrum of 2,6-Bis((2,2,6,6-tetramethylphosphinan-1-yl)methyl)pyridine, **TMPhos(PNP)**, compound **22**.

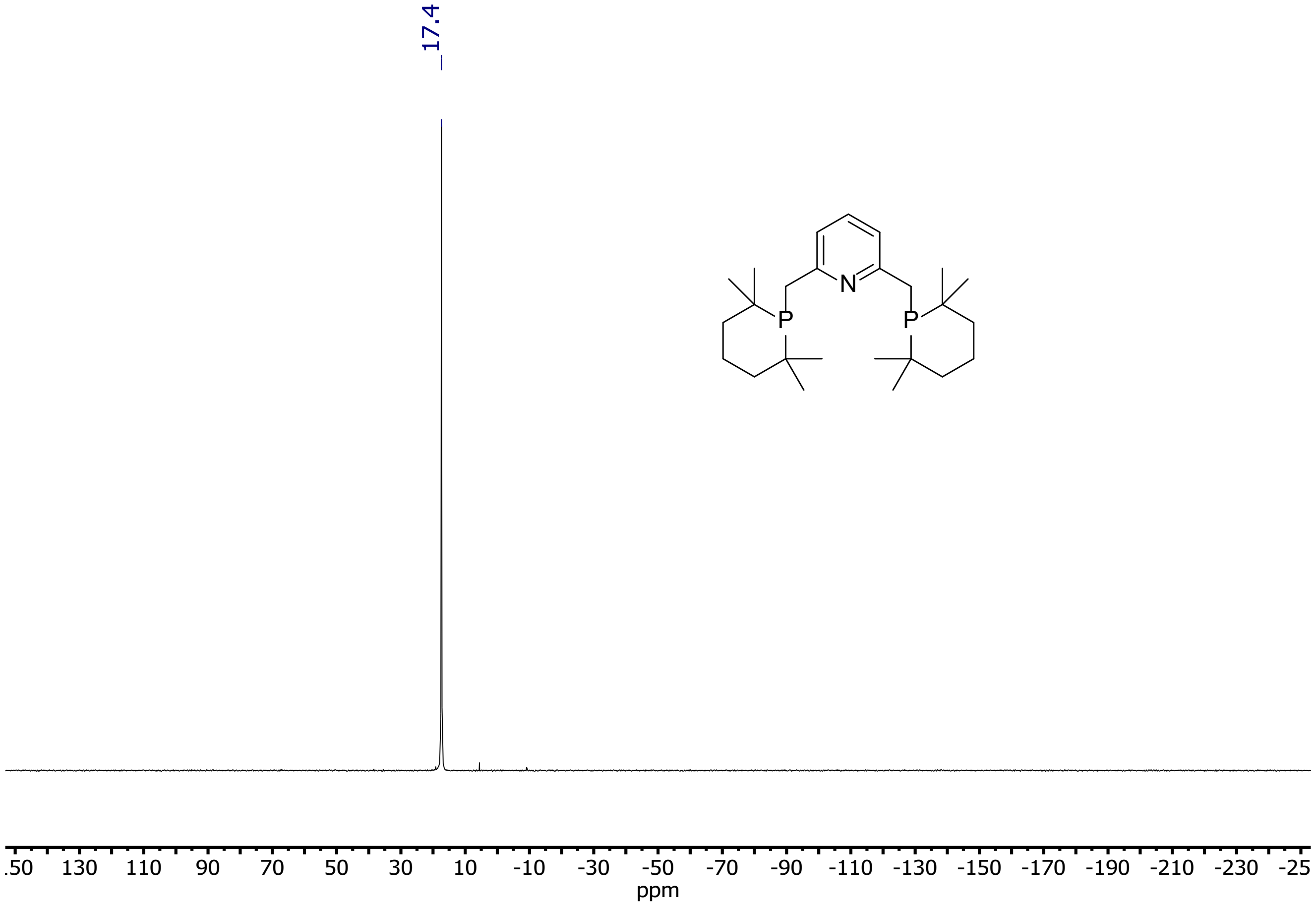

Supplementary Figure 107. $^{31}P\{^1H\}$ NMR (162 MHz, $CDCl_3$) spectrum of 2,6-Bis((2,2,6,6-tetramethylphosphinan-1-yl)methyl)pyridine, **TMPhos(PNP)**, compound **22**.

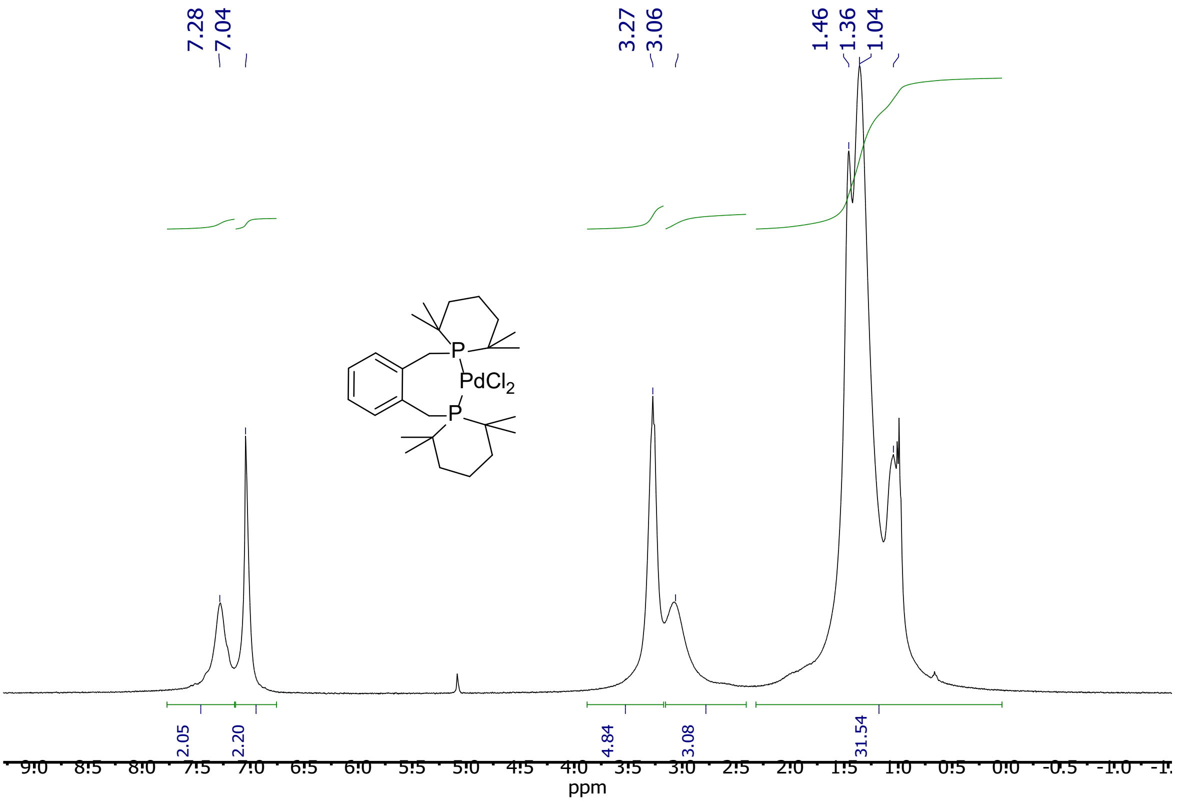

Supplementary Figure 108. ^{1}H NMR (400 MHz, $CDCl_3$) spectrum of [(**BTMPX**)$PdCl_2$].

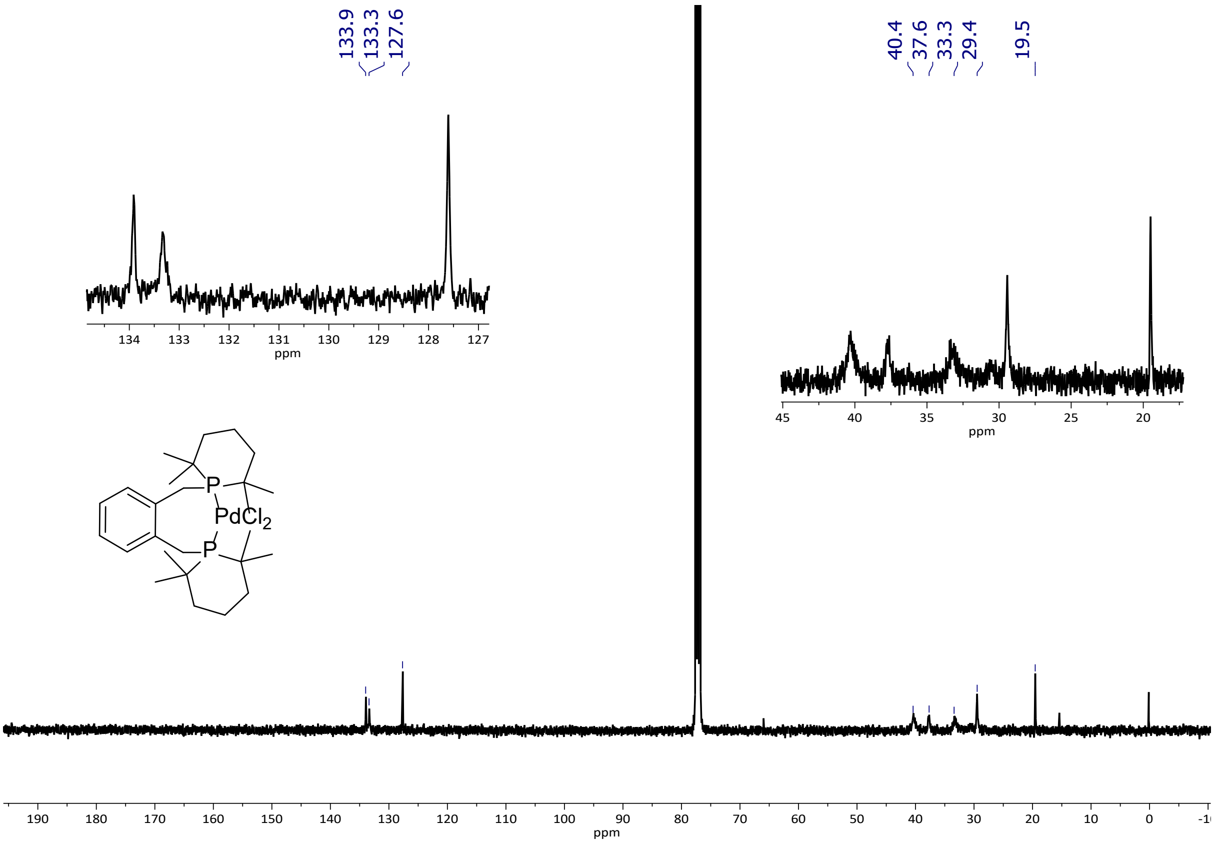

Supplementary Figure 109. ^{13}C{^{1}H} NMR (101 MHz, $CDCl_3$) spectrum of [(**BTMPX**)$PdCl_2$].

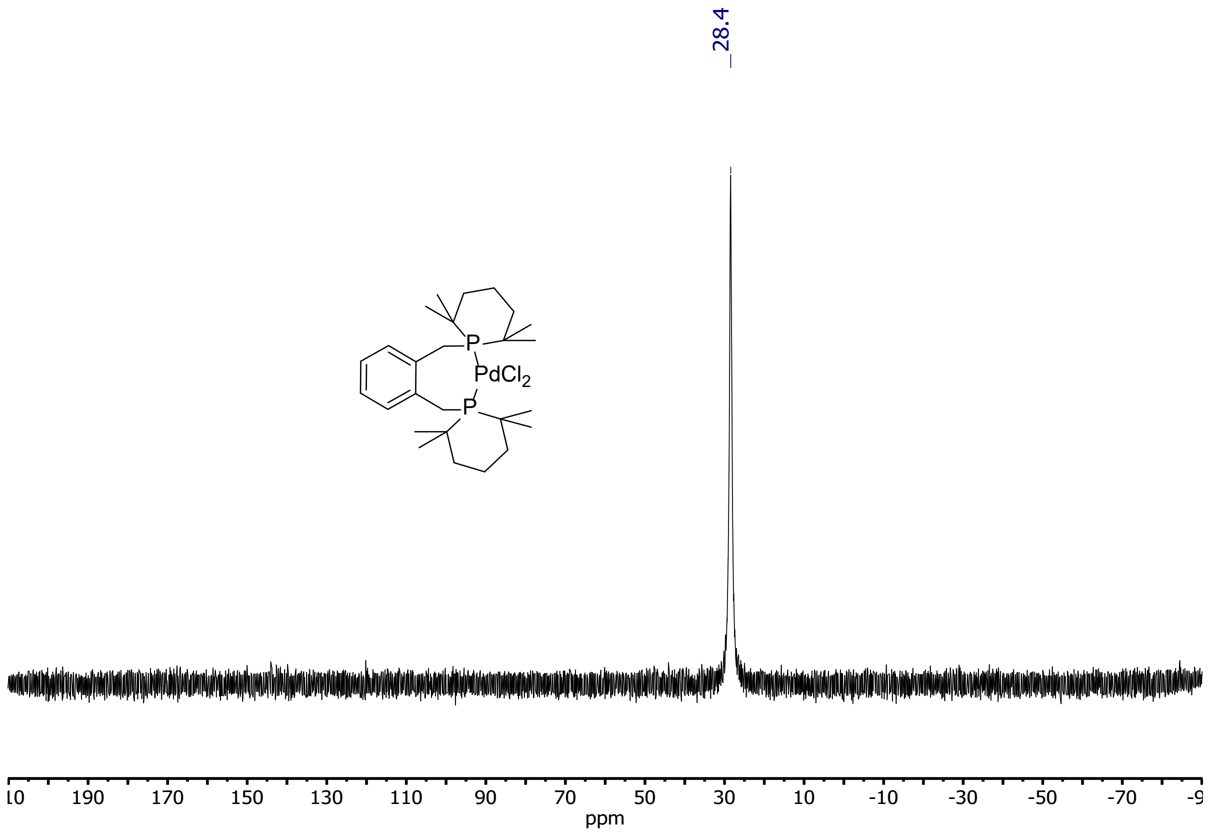

Supplementary Figure 110. $^{31}P\{^{1}H\}$ NMR (162 MHz, $CDCl_3$) spectrum of [(**BTMPX**)$PdCl_2$].

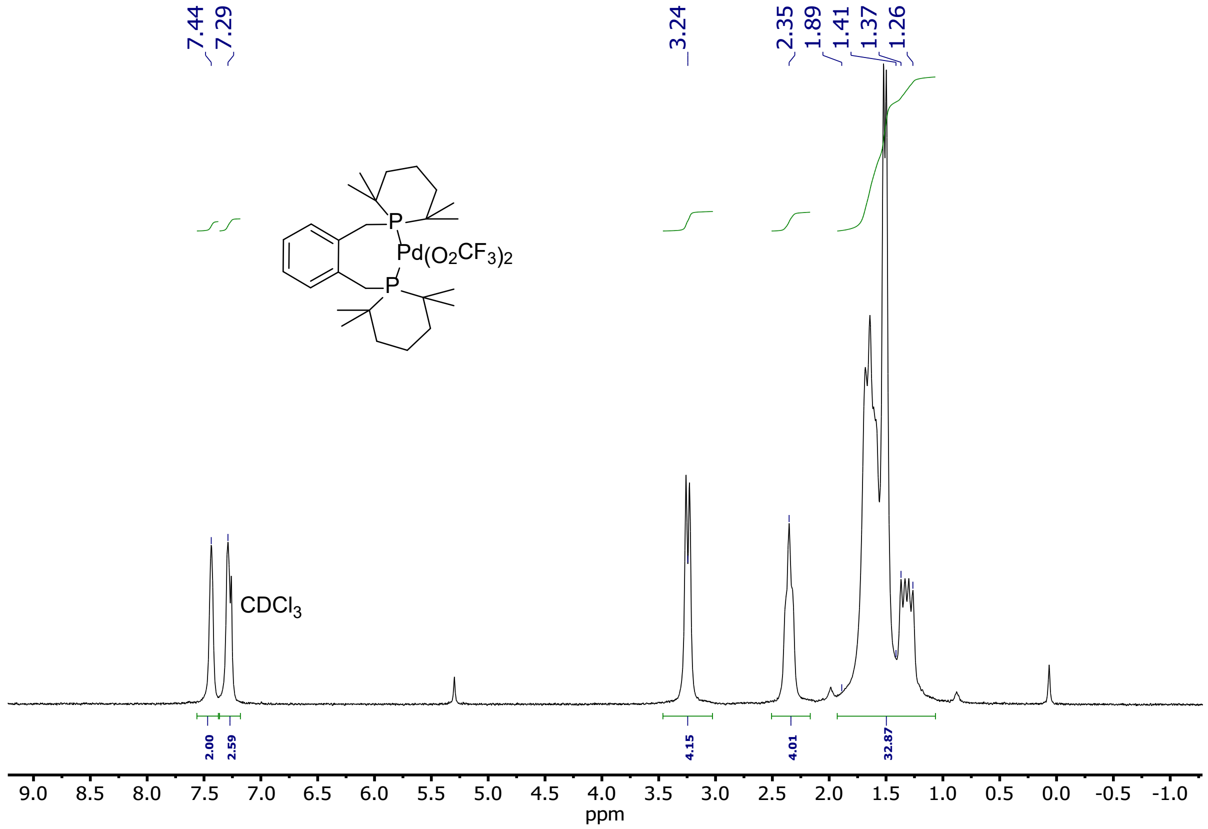

Supplementary Figure 111. ^{1}H NMR (400 MHz, $CDCl_3$) spectrum of [(**BTMPX**)$Pd(O_2CF_3)_2$], compound **23**.

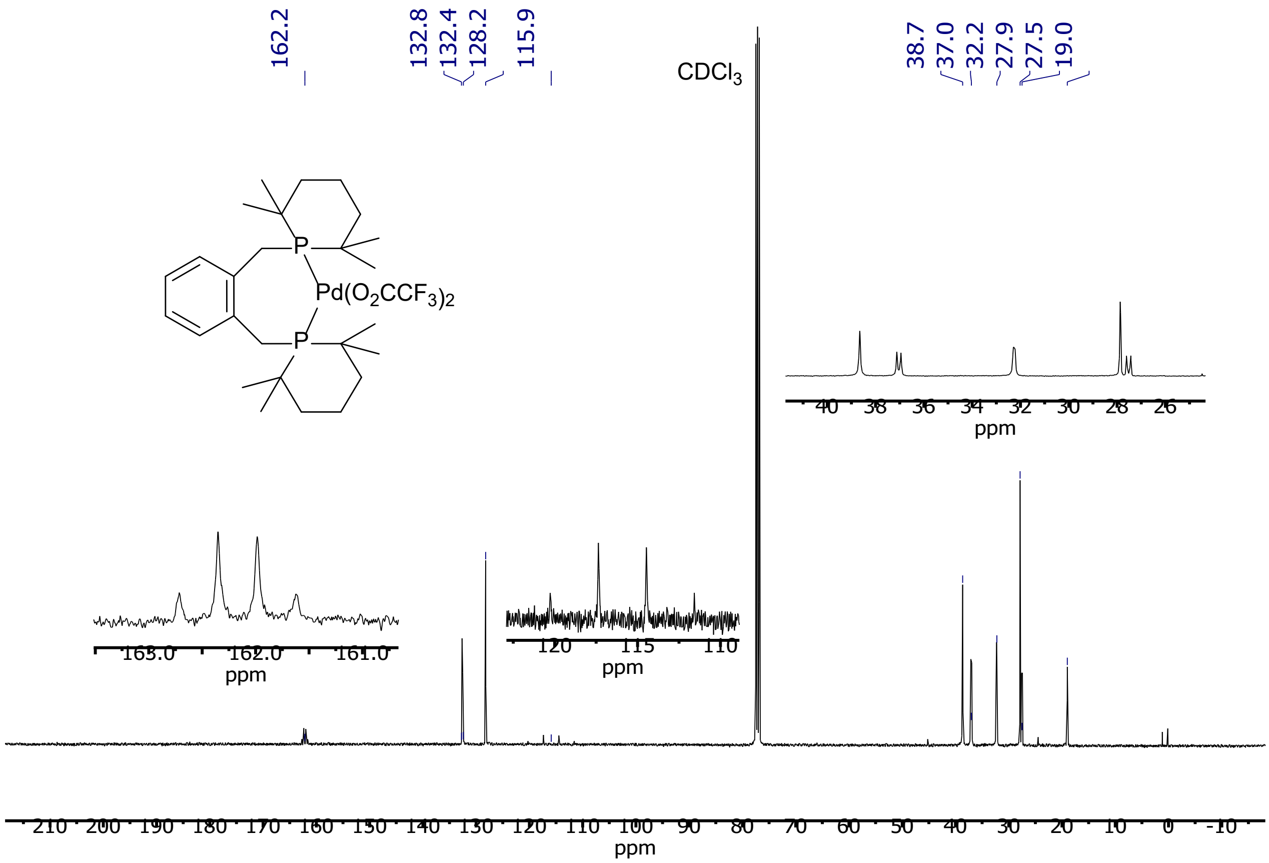

Supplementary Figure 112. $^{13}C\{^{1}H\}$ NMR (101 MHz, $CDCl_3$) spectrum of [(**BTMPX**)Pd(O_2CF_3)$_2$], compound **23**.

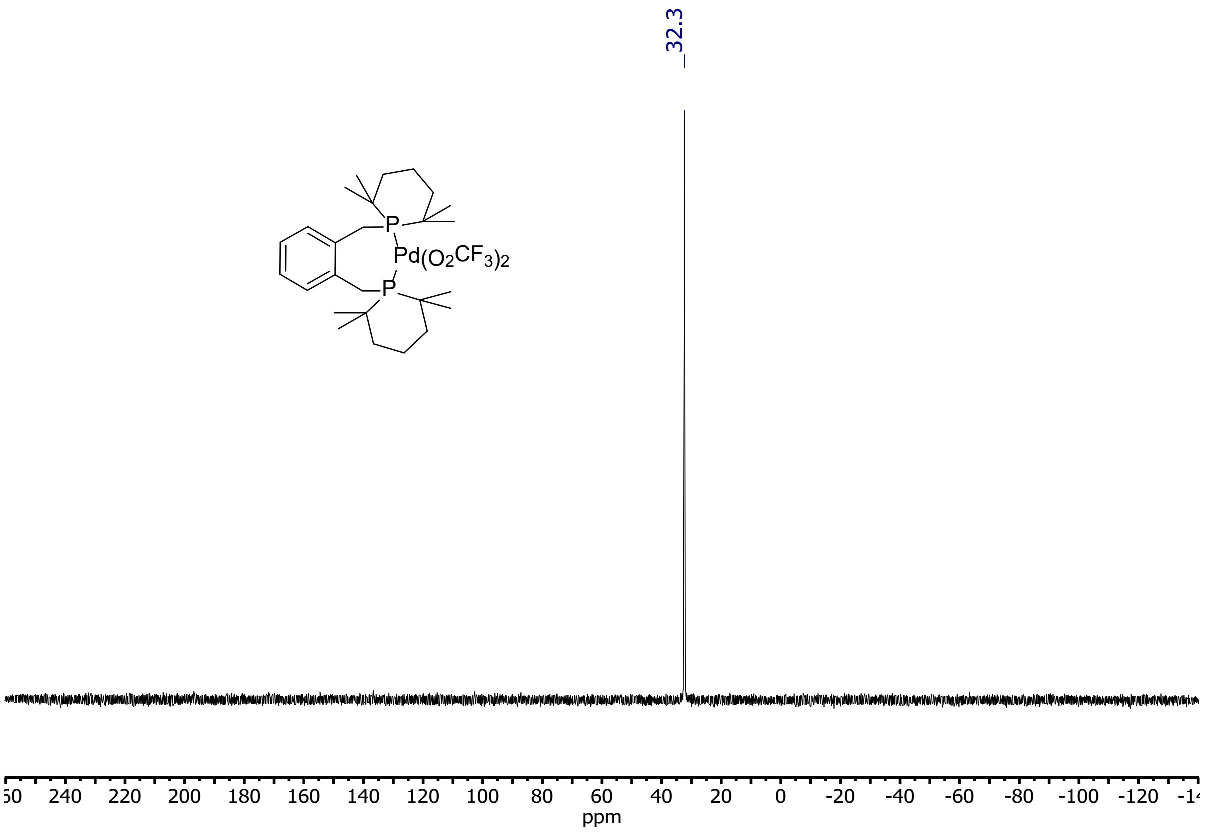

Supplementary Figure 113. $^{31}P\{^{1}H\}$ NMR (162 MHz, $CDCl_3$) spectrum of [(**BTMPX**)Pd(O_2CF_3)$_2$], compound **23**.

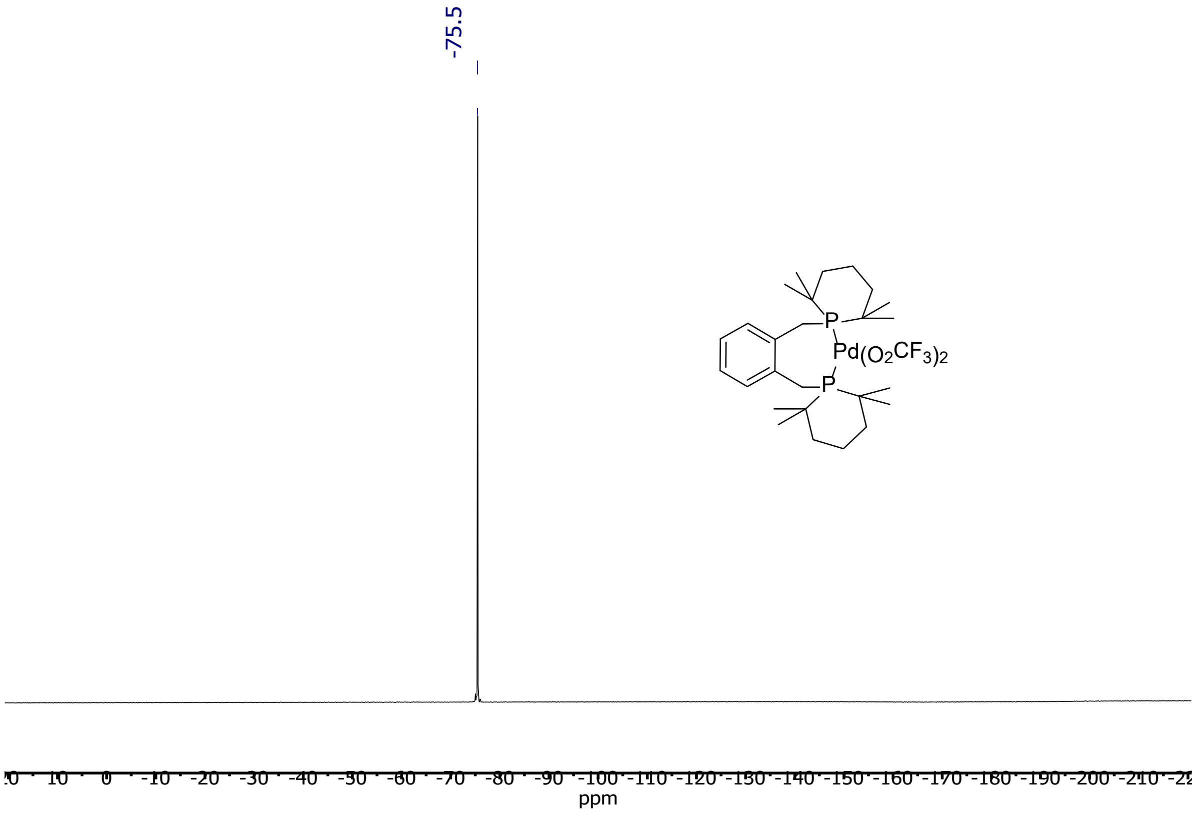

Supplementary Figure 114. ^{19}F NMR (376 MHz, $CDCl_3$) spectrum of [(**BTMPX**)Pd(O_2CF_3)$_2$], compound **23**.

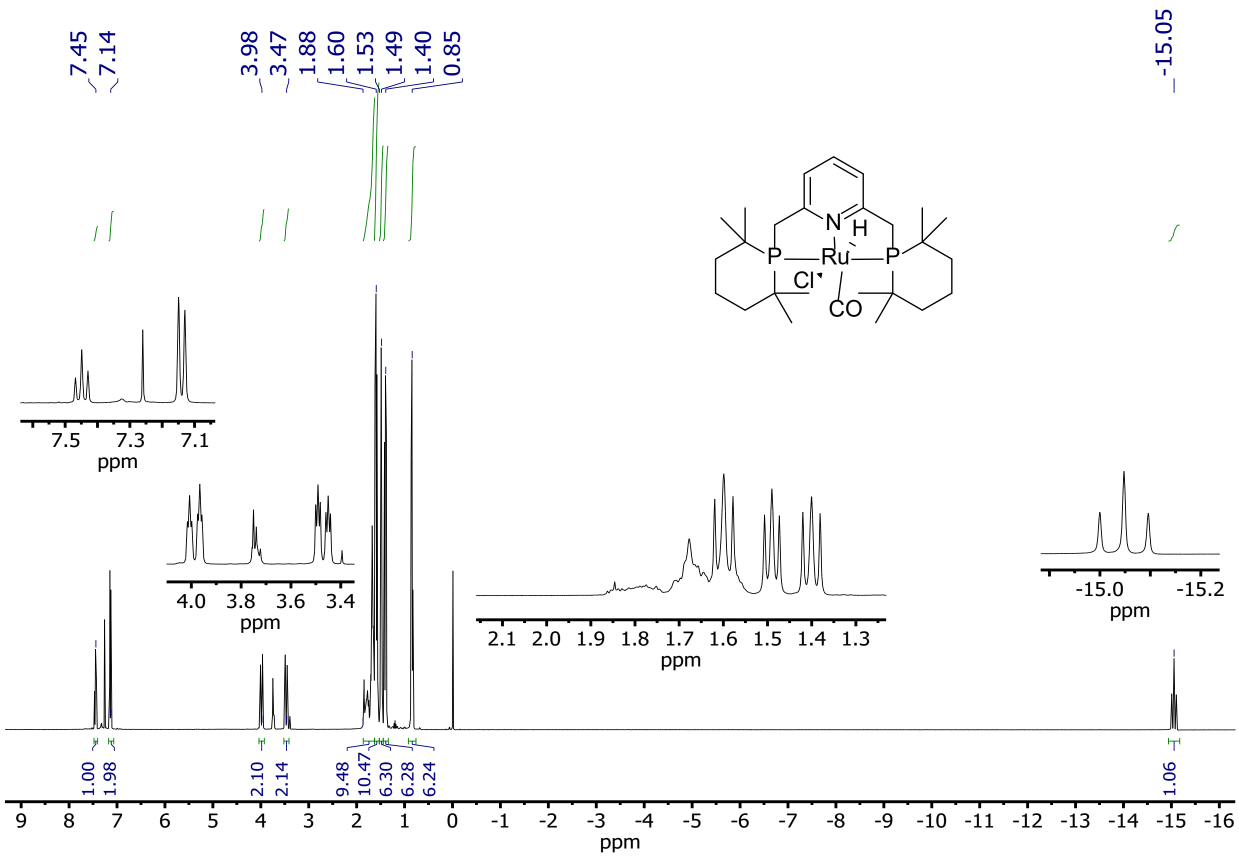

Supplementary Figure 115. ^{1}H NMR (400 MHz, $CDCl_3$) spectrum of **[^{TMPhos}(PNP)Ru(CO)(Cl)H]**, complex **24**.

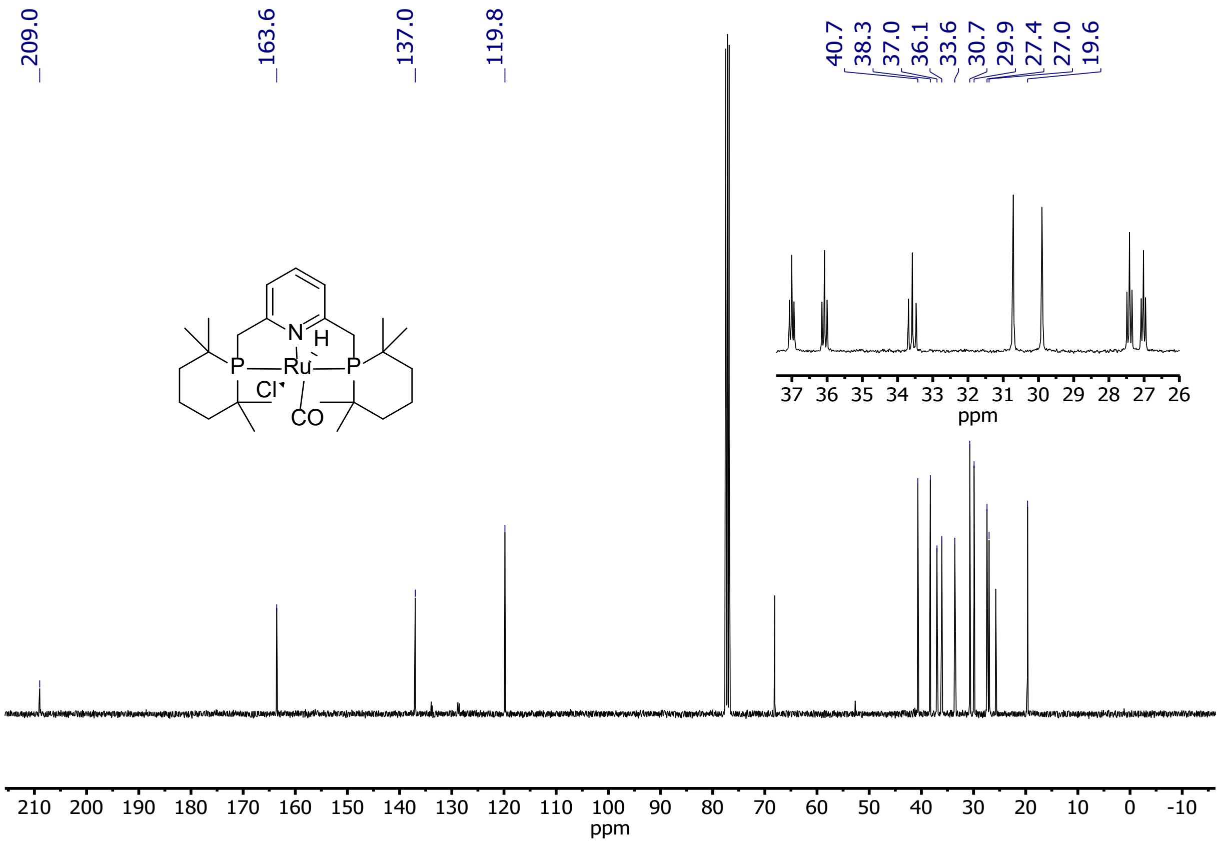

Supplementary Figure 116. $^{13}C\{^1H\}$ NMR (101 MHz, $CDCl_3$) spectrum of **[^{TMPhos}(PNP)Ru(CO)(Cl)H]**, complex **24**.

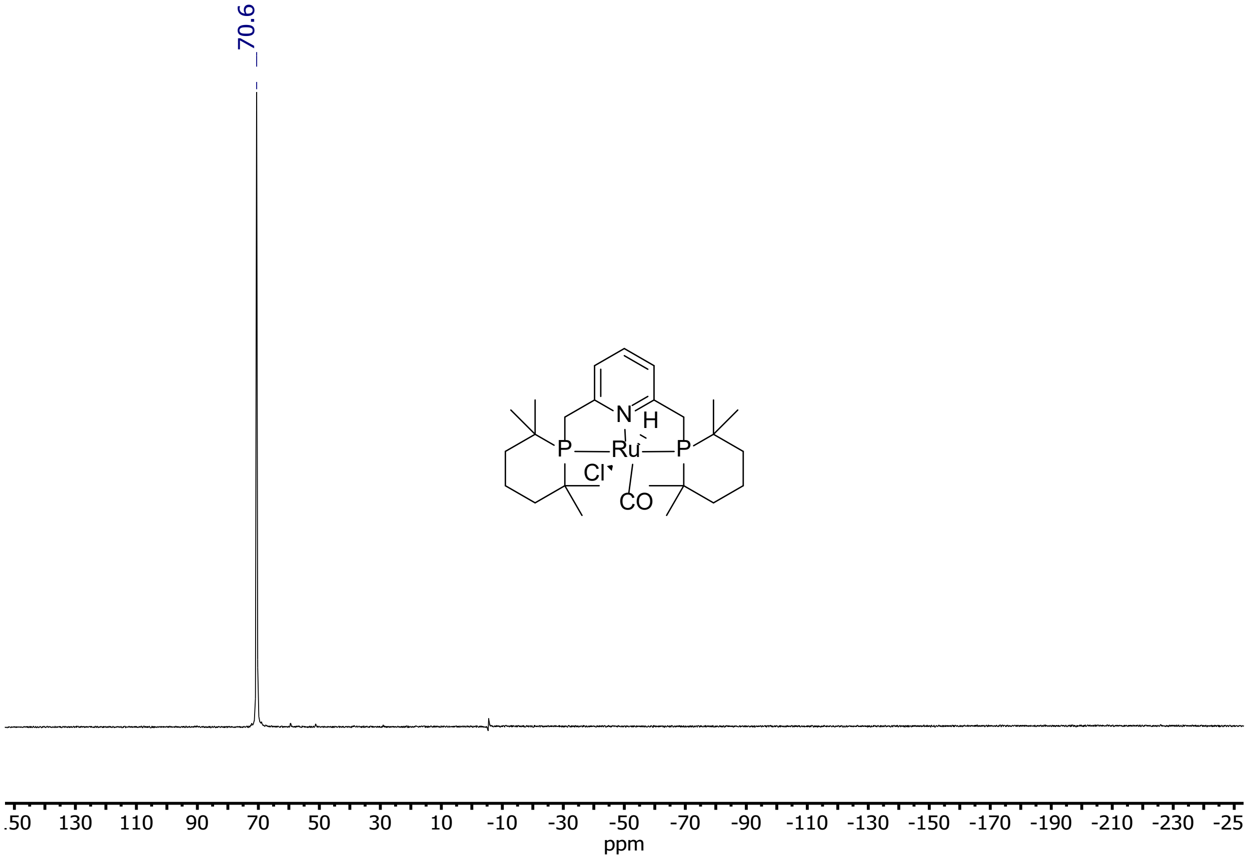

Supplementary Figure 117. $^{31}P\{^1H\}$ NMR (162 MHz, $CDCl_3$) spectrum of **[^{TMPhos}(PNP)Ru(CO)(Cl)H]**, complex **24**.

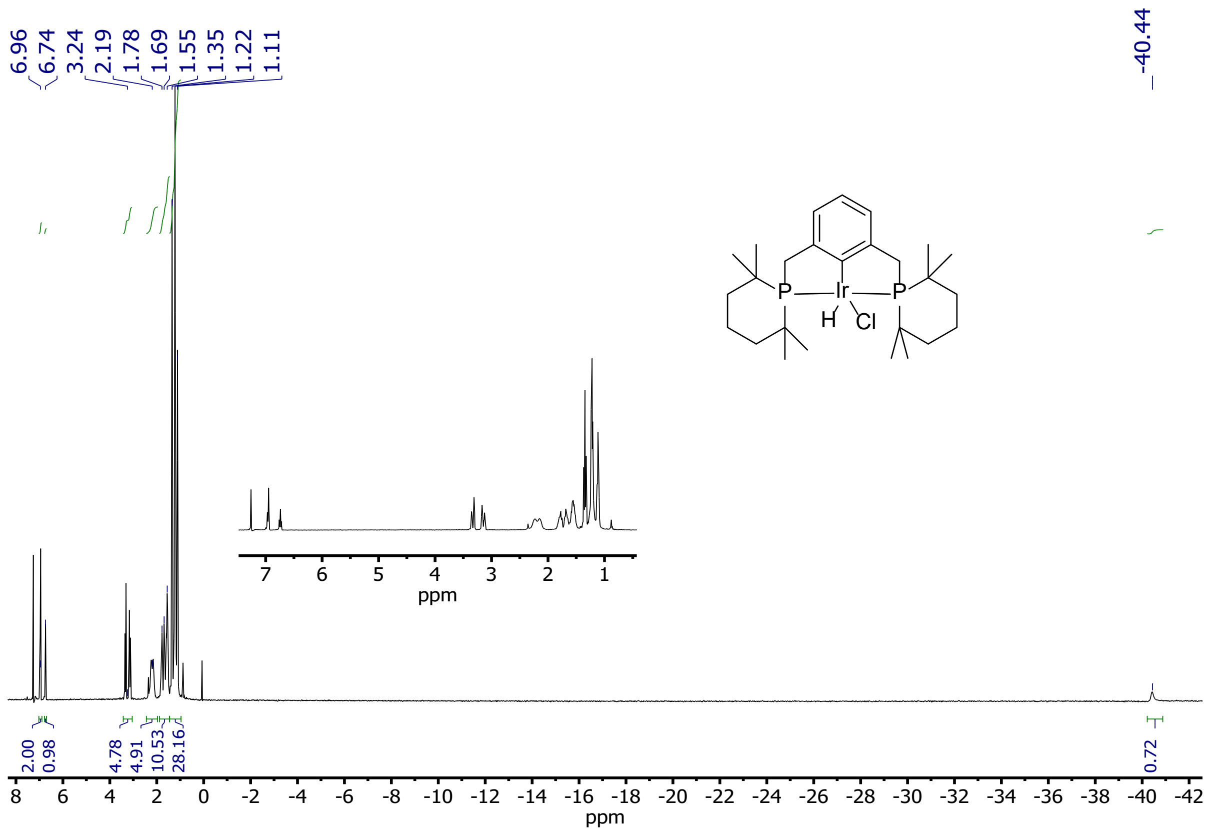

Supplementary Figure 118. ^{1}H NMR (400 MHz, $CDCl_3$) spectrum of [^{TMPhos}**(PCP)**Ir(H)Cl], compound **25**.

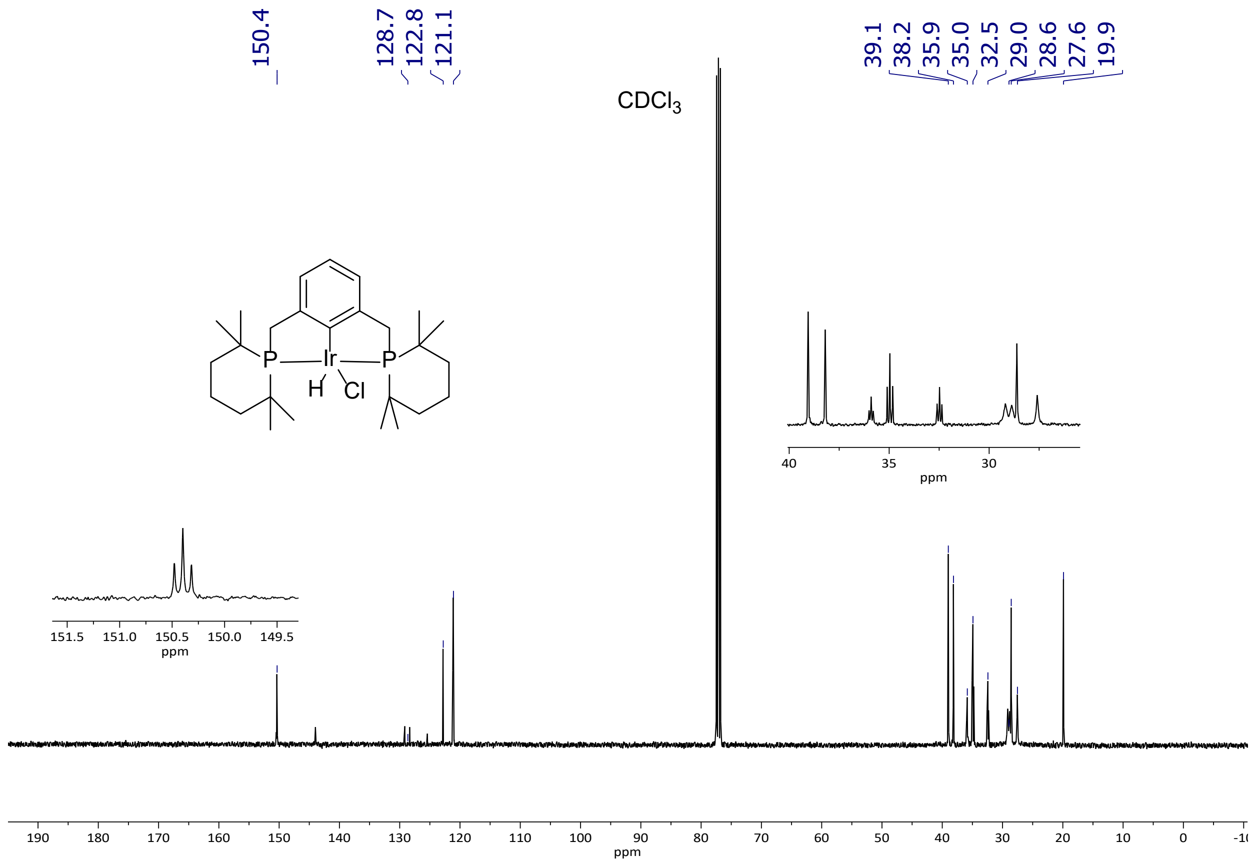

Supplementary Figure 119. $^{13}C\{^{1}H\}$ NMR (101 MHz, $CDCl_3$) spectrum of [^{TMPhos}**(PCP)**Ir(H)Cl], compound **25**.

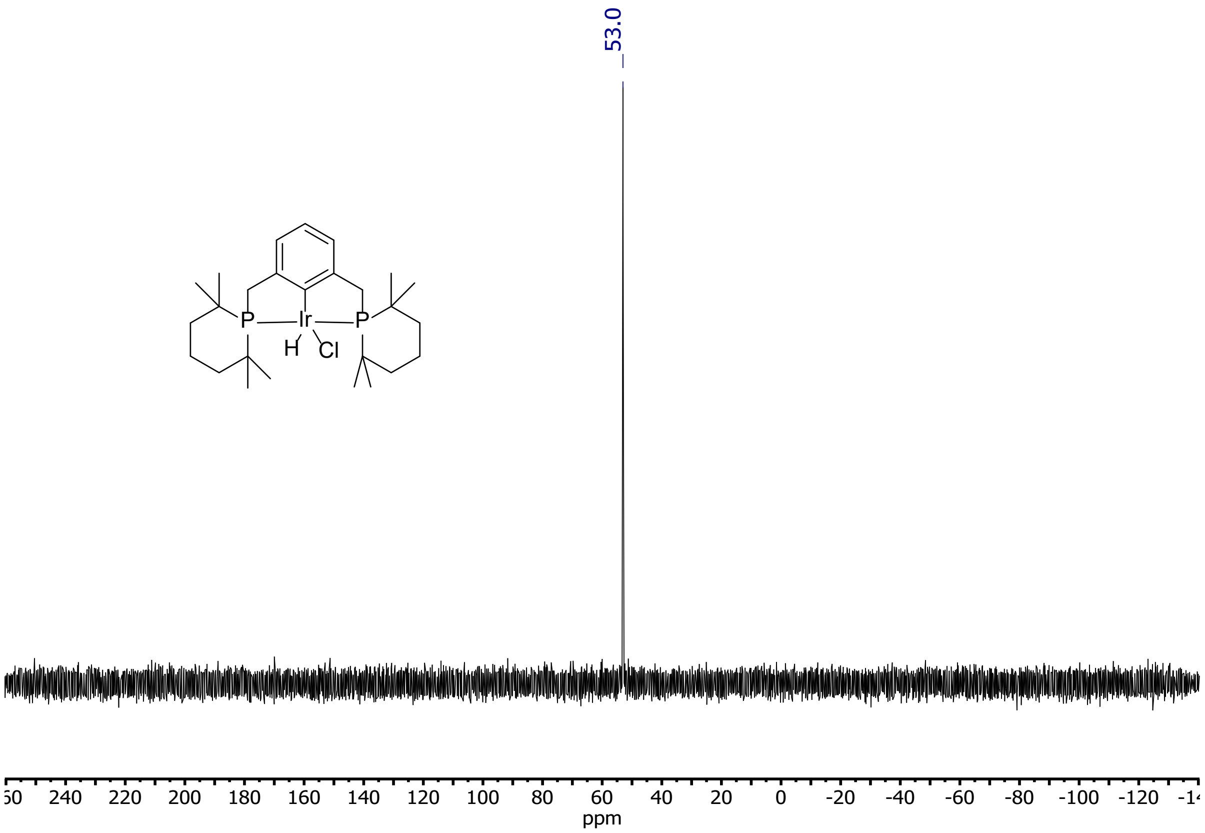

Supplementary Figure 120. $^{31}P\{^{1}H\}$ NMR (162 MHz, $CDCl_3$) spectrum of [^{TMPhos}**(PCP)**Ir(H)Cl], compound **25**.

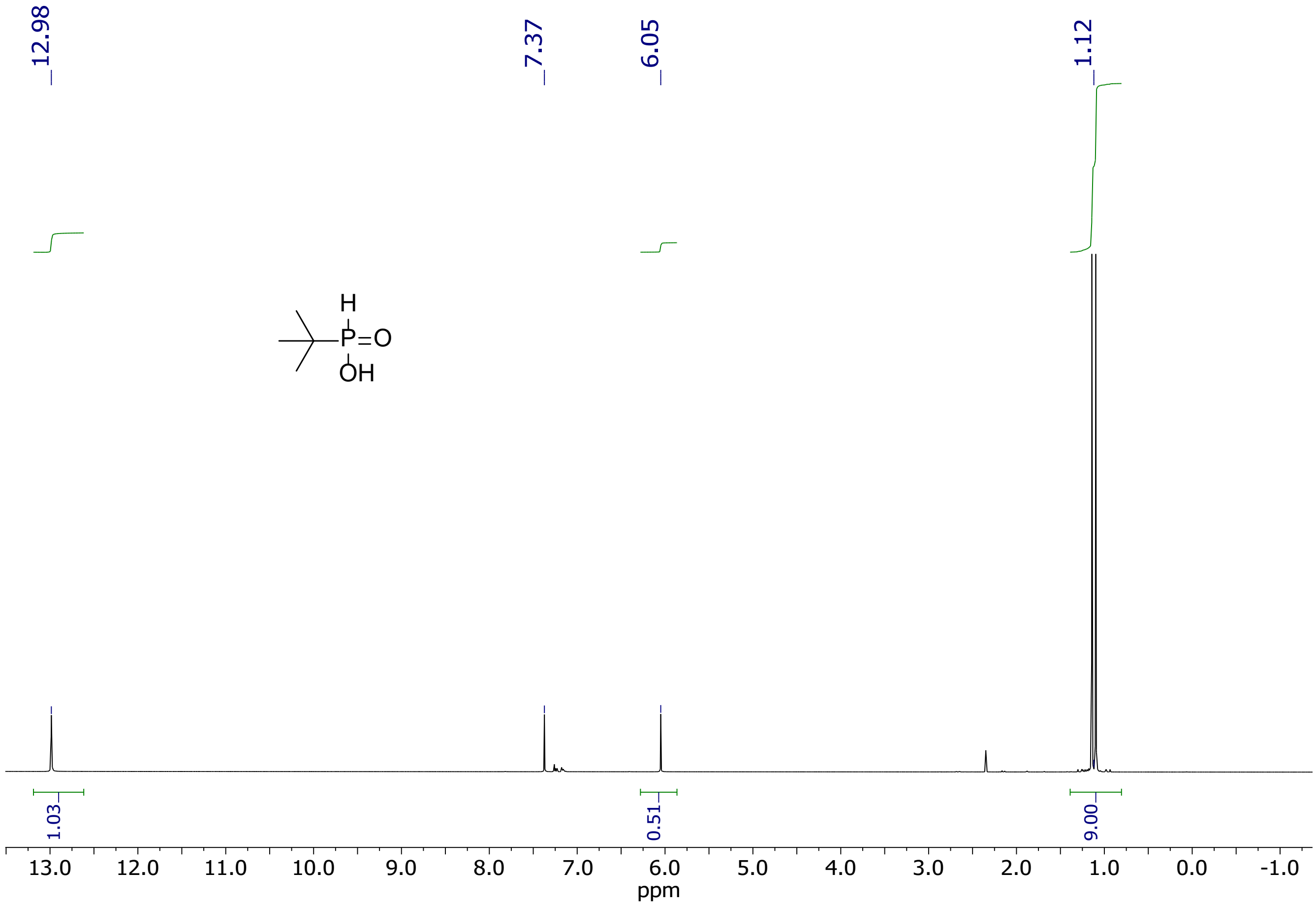

Supplementary Figure 121. ^{1}H NMR (400 MHz, $CDCl_3$) spectrum of *tert*-butylphosphinic acid.

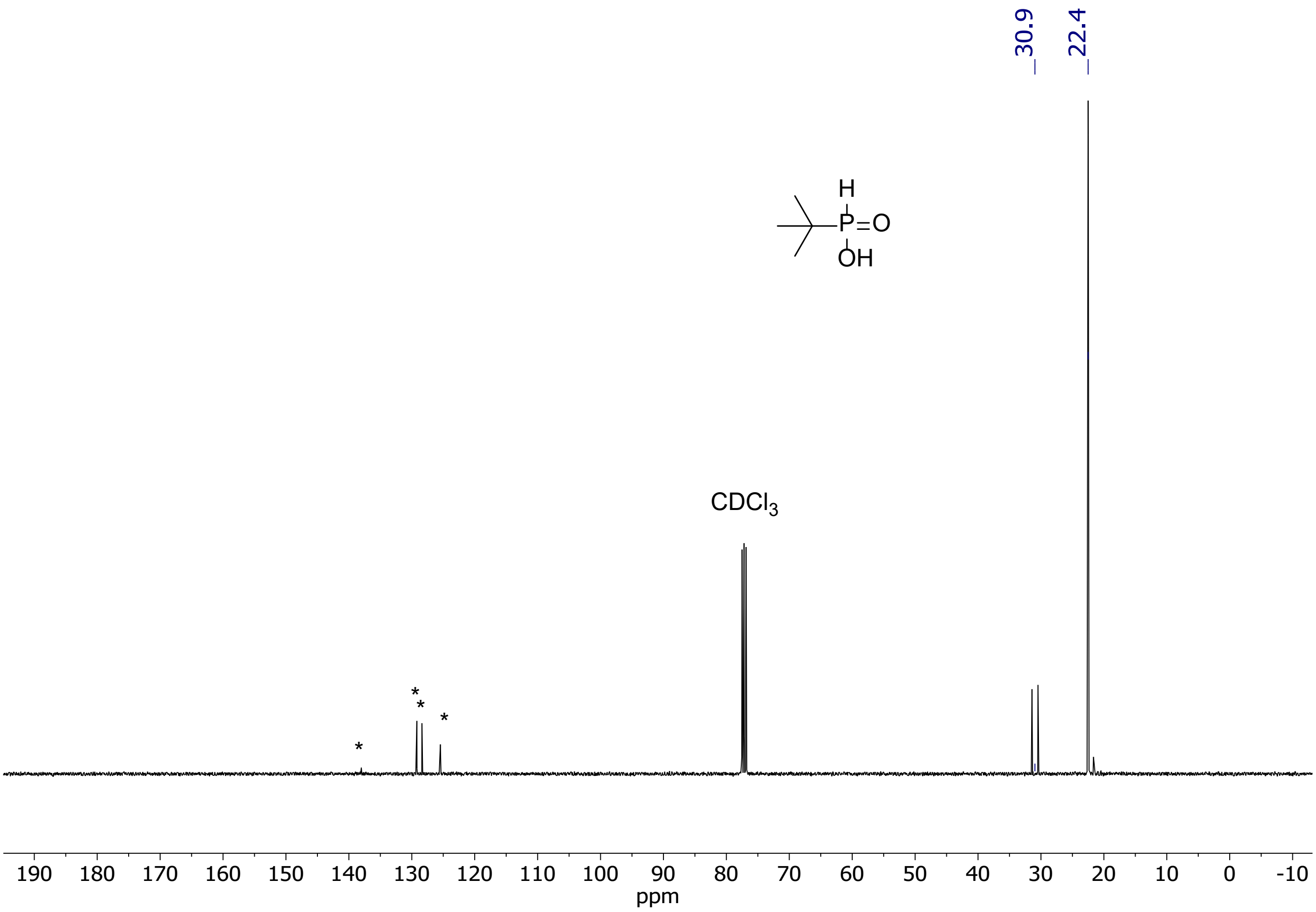

Supplementary Figure 122. $^{13}C\{^{1}H\}$ NMR (101 MHz, $CDCl_3$) spectrum of *tert*-butylphosphinic acid. *residual toluene impurity

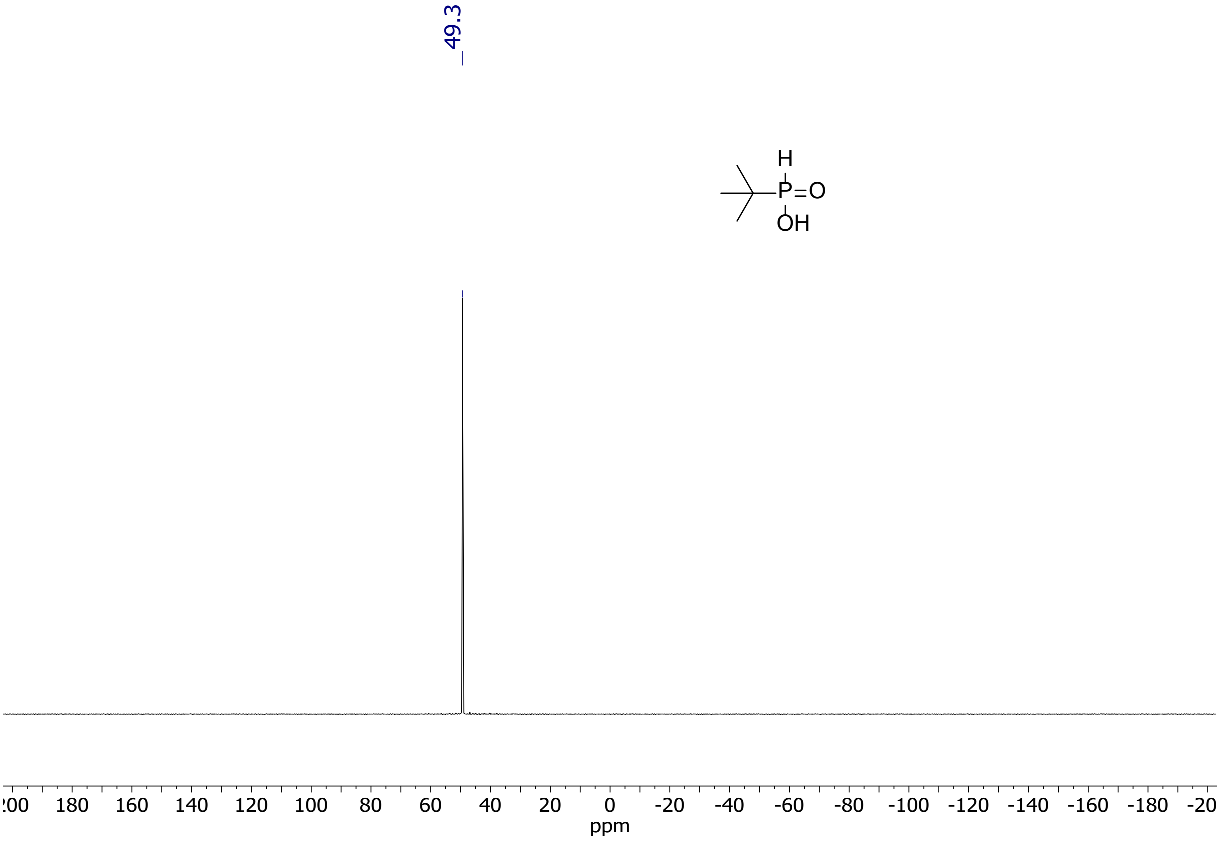

Supplementary Figure 123. $^{31}P\{^{1}H\}$ NMR (162 MHz, $CDCl_3$) spectrum of *tert*-butylphosphinic acid.
