# Supplementary material for: Tetramethylphosphinane as a new secondary phosphine synthon
Source: Commun Chem. 2023 Apr 29;6:85. doi: 10.1038/s42004-023-00876-8 (PMC10148838; doi:10.1038/s42004-023-00876-8)
Supplement: Supplementary file 10 — Supplementary Information [file 42004_2023_876_MOESM10_ESM.pdf]

Supplementary Information

Tetramethylphosphinane as a new secondary phosphine synthon

James D. Nobbs, Sigit Sugiarto, Xin Yi See, Choon Boon Cheong, Srinivasulu Aitipamula, Ludger P. Stubbs and Martin van Meurs*

Institute of Sustainability for Chemicals, Energy and Environment (ISCE^2), Agency for Science, Technology and Research (A*STAR), 1 Pesek Road, Jurong Island, Singapore 627833, Republic of Singapore.

Table of Contents

Supplementary Methods 2
1. Experimental Methods 2
2. Air oxidation of TMPhos vs ^{t}Bu$_2$PH (NMR Spectra) 13
3. Single Crystal X-ray Diffraction 15
4. Buried Volume Calculations 27
5. Calculation of Tolman Cone Angles 34
Supplementary References 35

Supplementary Methods

1. Experimental Methods

General Procedures

Air- or moisture-sensitive reactions were carried out under an atmosphere of purified nitrogen or argon, and compounds were stored in an argon-filled glove box. Solvents were dried using a Glass Contour solvent purification system where the solvents were passed through oxy- and moisture traps under an atmosphere of purified argon. CO gas was purified by passing through oxy- and moisture traps. NMR spectra were recorded on a Bruker Av Neo 400 MHz spectrometer equipped with an RT BBFO probe at a temperature of 298.0 K. The ^{1}H chemical shifts were referenced to residual protonated isomer in the NMR solvent used; ^{13}C chemical shifts were referenced to the ^{13}C chemical shift of the NMR solvent used; ^{31}P chemical shifts were unreferenced. Mass spectra were recorded on an Agilent G1969A/6210 TOF-MS. Elemental analyses were determined using an Organic Elemental Analysis Flash 2000 CHNS/O Elemental Analyzer. In several cases the experimental elemental analysis values obtained were outside the 0.4% tolerance. Therefore, NMR spectra of all new compounds have been included in the ESI to demonstrate the absence of detectable contaminants. The source of the commercial compounds are as follows: [(acac)Rh(CO)$_2$] (STREM, 99%), [allyl(cyclopentadienyl)palladium(II)] (STREM, 98%), 2,6-bis(bromomethyl)pyridine (MERCK, 98%), borane dimethyl sulfide complex (MERCK, 2.0 M in THF), *tert*-butyldichlorophosphine (MERCK, 98%), ^{n}BuLi (MERCK, 2.0 M in cyclohexane), [carbonylchlorohydridotris(triphenylphosphine)ruthenium(II)] (STREM, 99%), *m*-CPBA (Alfa Aesar, 70%, aq), chloro-1,5-cyclooctadiene iridium(I) dimer (STREM, 99%), 1,4-dibromobutane (MERCK, 99%), 1,3-dibromopropane (MERCK, 97%), α,α-dibromo-*m*-xylene (MERCK, 97%), α,α-dibromo-*o*-xylene, (MERCK, 98%), diethylene glycol (MERCK, 99%), 9,9-dimethylxanthene (MERCK, 96%), HP^{t}Bu$_2$ (STREM, 98%, 10wt% in hexane), HMDS (MERCK, ≥99%), Hünigs base (MERCK, 99.5%), hydrazine monohydrate (MERCK, 65 wt%, aq), lithium (MERCK, ≥99%, granules), $LiAlH_4$ (MERCK, 95%, powder), 2,6-lutidine (MERCK, ≥99%,), methyl trichloroacetate (MERCK, 99%), $NH_4H_2PO_2$ (Biosynth, purity not specified), oxalyl chloride (MERCK, 98%), [Pd(OAc)$_2$] (STREM, ≥99%), [Pd(dba)$_2$], (STREM, 99%) phenylphosphine (ABCR, 99%), phorone (ABCR, 95%), selenium (MERCK, ≥99.5% trace metals basis), [Ag(O$_2$CCF$_3$)$_2$] (STREM, 98%), NEt$_3$ (MERCK, 99%) and TMSCl (MERCK, ≥99%). The following compounds were synthesized according to literature procedures. Methyl hypophosphite,[1] [1,1'-biphenyl]-2-yl trifluoromethanesulfonate,[2] 1,5-dihydrobenzo[e][1,3,2]dioxathiepine 3,3-dioxide[3] and 2,6-bis(tosyloxymethyl)pyridine[4] were synthesized according to literature procedures.

2,2,6,6-Tetramethyl-1-phenylphosphinan-4-one, (i).

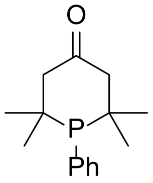

Phenylphosphine (6.25 g, 56.8 mmol) and phorone (7.85 g, 56.8 mmol) were heated together at 120°C for 2 hours, then the temperature was increased to 160°C for a further 16 hours. Upon cooling a yellow solid precipitated which was dried *in vacuo*. Heptane (20 mL) was added and the mixture was recrystallized by heating it to 60°C and then cooling to RT. After filtration and drying a white solid was obtained. Yield = 8.1 g, (57%). ^{1}H NMR (400 MHz, CDCl$_3$): δ 7.76 – 7.67 (2H, m, Ar*H*), 7.45 – 7.35 (3H, m, Ar*H*), 2.90 (2H, dd, $^{2}J_{HH}$ = 12.9 Hz and $^{3}J_{HP}$ = 1.3 Hz, C*H*$_2$), 2.29 (2H, dd, $^{2}J_{HH}$ = 12.9 Hz and $^{3}J_{HP}$ = 5.2 Hz, C*H*$_2$), 1.30 (6H, d, $^{3}J_{HP}$ = 17.7 Hz, C*H*$_3$) and 0.91 ppm (6H, d, $^{3}J_{HP}$ = 11.2 Hz, C*H*$_3$). ^{13}C{^{1}H} NMR (101 MHz, CDCl$_3$): δ 211.7 (s, *C*=O), 135.9 (d, $^{2}J_{CP}$ = 23.0 Hz, Ar*C*H), 135.8 (d, $^{1}J_{CP}$ = 24.3 Hz, Ar*C*$_q$P), 129.8 (d, $^{4}J_{CP}$ = 0.95 Hz, Ar*C*H), 128.5 (d, $^{3}J_{CP}$ = 8.5 Hz, Ar*C*H), 53.1 (d, $^{2}J_{CP}$ = 2.9 Hz, *C*H$_2$), 35.4 (d, $^{1}J_{CP}$ = 18.1 Hz, *C*(CH$_3$)$_2$), 31.2 (d, $^{2}J_{CP}$ = 30.9 Hz, C(*C*H$_3$)$_2$) and 30.2 ppm (d, $^{2}J_{CP}$ = 9.2 Hz, C(*C*H$_3$)$_2$). ^{31}P{^{1}H} NMR (162 MHz, CDCl$_3$): δ 16.6 ppm. HR-MS (+ve ESI): m/z (calc.) [M+H]$^+$ 249.1408; found 249.1401. Elem. Anal. Calcd for $C_{15}H_{21}OP$: C, 72.56; H, 8.52. Found: C, 72.04; H, 8.19.

2,2,6,6-Tetramethyl-1-phenylphosphinane, (ii).

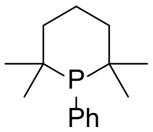

2,2,6,6-Tetramethyl-1-phenylphosphinan-4-one (8.12 g, 32.7 mmol) was dissolved in diethylene glycol (20 mL). KOH (9.17 g, 163.5 mmol) and hydrazine monohydrate (65 wt% in water, 12.2 mL, 163.5 mmol) were added. The mixture was heated gradually to 170°C over 1 hour and water was removed using a Dean–Stark apparatus, after which the temperature was then increased to 210°C and maintained for 16 hours. Then, the mixture was extracted with pentane (4 × 40 mL) and the combined extracts dried ($MgSO_4$) and evaporated. The colourless oil obtained was filtered over a small pad of silica. Colourless liquid. Yield = 3.6 g (47 %). ^{1}H NMR (400

MHz, $CDCl_3$): δ 7.77 – 7.67 (2H, m, Ar*H*), 7.39 – 7.27 (3H, m, Ar*H*), 1.92 – 1.68 (4H, m, C*H*$_2$), 1.58 – 1.46 (2H, m, C*H*$_2$), 1.28 (6H, d, $^3J_{HP}$ = 18.3 Hz, C*H*$_3$) and 0.80 ppm (6H, d, $^3J_{HP}$ = 10.7 Hz, C*H*$_3$). $^{13}C\{^1H\}$ NMR (101 MHz, $CDCl_3$): δ. 137.5 (d, $^1J_{CP}$ = 24.1 Hz, Ar*C*$_q$), 137.0 – 136.0 (m, Ar*C*), 129.0 (s, Ar*C*), 127.8 (d, J_{CP} = 8.3 Hz, Ar*C*), 37.6 (s, $CH_2CH_2CH_2$), 30.6 (d, $^2J_{CP}$ = 9.0 Hz, *C*H$_3$), 30.4 (d, $^2J_{CP}$ = 32.0 Hz, *C*H$_3$), 29.4 (d, $^1J_{CP}$ = 14.0 Hz, *C*(CH$_3$)$_2$) and 20.4 ppm (s, $CH_2CH_2CH_2$). $^{31}P\{^1H\}$ NMR (162 MHz, $CDCl_3$): δ 19.2 ppm. HR-MS (+ve ESI): m/z (calc.) $[M+H]^+$ 235.1616; found 235.1610. Elem. Anal. Calcd for $C_{15}H_{23}P$: C, 76.89; H, 9.89. Found: C, 76.59; H, 9.70.

2,2,6,6-Tetramethylphosphinane (TMPhos), (iii), from Li cleavage of phenyl phosphinane.

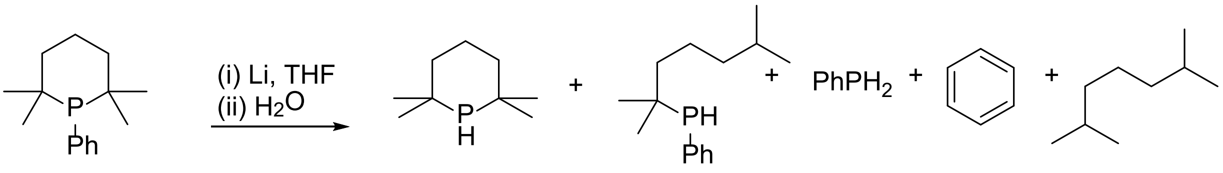

(ii) **(iii)** **(iii)b**

Li pieces (631 mg, 90.9 mmol) and 2,2,6,6-tetramethyl-1-phenylphosphinane, **(ii)**, (5.0 g, 21.3 mmol) in THF (40 mL) were heated to reflux then cooled to RT and stirred vigorously. After 72 h the mixture was cooled to 0°C and quenched carefully with degassed H_2O (50 mL). The mixture was extracted with Et_2O (4 × 40 mL), dried ($MgSO_4$) and the Et_2O/THF was distilled off. Analysis of the raffinate by ^{31}P NMR spectroscopy showed the presence of TMPhos, **(iii)** (^{31}P δ = −9.0 ppm, minor product, ~30% of the integral area) and **(iii)b** (−11.0 ppm, major product, ~50%), as well as residual $PhPH_2$ (~1%) and some unreacted **(ii)**/phosphine oxides (~10%). TMPhos, **(iii)** was separated from **(iii)b** and unreacted **(ii)** by vacuum distillation, but dimethylheptane and benzene also were also still present in the crude mixture. Yield = 0.80 g (26%) approx. 90% pure. ^1H NMR (400 MHz, $CDCl_3$): δ 2.60 (1H, dsept., $^1J_{PH}$ = 200.1 Hz, and $^3J_{HP}$ = 1.5 Hz, P*H*), 1.64 – 1.50 (4H, m, C*H*$_2$), 1.23 – 1.13 (2H, m, C*H*$_2$), 1.03 (6H, dd, $^3J_{PH}$ = 15.3 Hz and $^4J_{HH}$ = 1.2 Hz, C*H*$_3$) and 0.94 ppm (6H, d, $^3J_{PH}$ = 14.1 Hz, C*H*$_3$). $^{13}C\{^1H\}$ NMR (101 MHz, $CDCl_3$): δ 43.4 (d, $^2J_{CP}$ = 6.4 Hz, C_qCH_2), 32.6 (d, $^2J_{CP}$ = 13.7 Hz, *C*H$_3$). 27.7 (d, $^1J_{CP}$ = 8.9 Hz, *C*(CH$_3$)$_2$), 26.4 (d, $^2J_{CP}$ = 25.1 Hz, *C*H$_3$) and 20.9 ppm (s, -*C*H$_2$-). $^{31}P\{^1H\}$ NMR (162 MHz, $CDCl_3$): δ −9.1 ppm.

2,2,6,6-Tetramethylphosphinane borane complex, (iv).

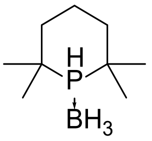

Borane dimethyl sulfide complex (2.0 M in THF, 2.5 mL, 4.9 mmol) was added to the crude TMPhos from the previous reaction (0.77 g, 4.90 mmol) in THF (5 mL). After stirring for 2.5 hours the volatiles were removed *in vacuo* to yield a white solid. Yield = 0.50 g (59%). ^1H NMR (400 MHz, $CDCl_3$): δ 3.98 (1H, d of m, $^1J_{HP}$ = 318.1 Hz, P*H*), 1.83 – 1.55 (4H, m, C*H*$_2$CH$_2$C*H*$_2$), 1.50 – 1.40 (2H, m, CH$_2$C*H*$_2$CH$_2$), 1.20 (6H, d, $^3J_{HP}$ = 14.2 Hz, C*H*$_3$), 1.19 (6H, d, $^3J_{HP}$ = 15.5 Hz, C*H*$_3$) and 0.43 ppm (3H, br. q, $^1J_{HB}$ = 99.2 Hz, B*H*$_3$). $^{13}C\{^1H\}$ NMR (101 MHz, $CDCl_3$): δ 41.8 (d, $^2J_{CP}$ = 5.2 Hz, C_qCH_2), 30.2 (d, $^2J_{CP}$ = 1.7 Hz, *C*H$_3$), 28.0 (d, $^1J_{CP}$ = 29.4 Hz, *C*(CH$_3$)$_2$), 22.0 (d, $^2J_{CP}$ = 7.4 Hz, *C*H$_3$) and 19.4 ppm (d, $^3J_{CP}$ = 4.8 Hz, CH$_2$*C*H$_2$CH$_2$). $^{31}P\{^1H\}$ NMR (162 MHz, $CDCl_3$): δ 34.3 ppm (q, $^1J_{HB}$ = 46.1 Hz).

Attempted synthesis of 1-methoxy-2,2,6,6-tetramethylphosphinan-4-one 1-oxide, (v).

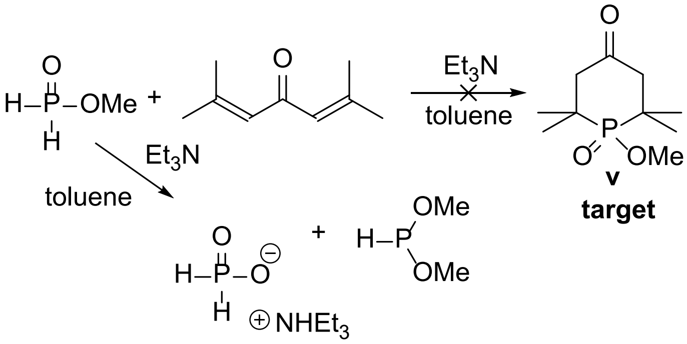

Methyl hypophosphite[1] solution in THF:toluene (1:1, 5.0 mL, containing ~5.0 mmol of H_2PO_2Me), phorone (0.59 g, 4.26 mmol), toluene (1.0 mL) and NEt_3 (0.50 mL, 5.0 mmol) were stirred at RT and monitored by ^{31}P NMR spectroscopy over the course of 4 days. No reaction with phorone was observed but instead the formation of phosphorus acid triethylamine salt and dimethyl phosphonite. ^{31}P NMR (162 MHz, THF/d_8-tol): 171.8 ppm (d of sept, $^1J_{PH}$ = 199.1 Hz, $^3J_{PH}$ = 9.3 Hz, H*P*(OMe)$_2$) and −0.7 ppm (t, J = 488.2 Hz, $[H_2PO_2]^-$ $[HNEt_3]^+$).

Trimethylsilyl (Z)-(2,6-dimethyl-4-((trimethylsilyl)oxy)hepta-3,5-dien-2-yl)phosphinate, 1.

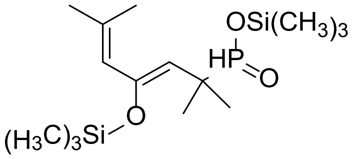

A small aliquot of the reaction mixture for the synthesis of compound **2** was extracted prior to acid hydrolysis and the volatiles were removed *in vacuo* giving a colorless oil. ^{1}H NMR (400 MHz, $CDCl_3$): δ 6.69 (1H, d, $^1J_{HP}$ = 544.2 Hz, P*H*), 5.51 – 5.46 (1H, m, $(CH_3)_3C=CH$), 4.16 (1H, dd, $^3J_{HP}$ = 9.3 Hz, $^4J_{HH}$ = 1.0 Hz, $(CH_3)_3SiOC=CH$), 1.77 (3H, d, $^4J_{HH}$ = 1.5 Hz, $(CH_3)_3C=CH$), 1.72 (3H, d, $^4J_{HH}$ = 1.5 Hz, $(CH_3)_3C=CH$), 1.32 (3H, d, $^3J_{HP}$ = 18.1 Hz, CH_3CP), 1.24 (3H, d, $^3J_{CP}$ = 17.4 Hz, CH_3CP), 0.26 (9H, s, $POSi(CH_3)_3$) and 0.12 ppm (9H, s, $OSi(CH_3)_3$). $^{13}C\{^1H\}$ NMR (101 MHz, $CDCl_3$): δ 149.3 (d, $^2J_{SiC}$ = 11.7 Hz, OC_q), 137.7 (d, $^4J_{SiC}$ = 3.3 Hz, $(CH_3)_2C=CH$), 123.6 (d, $^3J_{SiC}$ = 3.5 Hz, $(CH_3)_2C=CH$), 110.6 (d, $^2J_{CP}$ = 5.1 Hz, $(CH_3)_3SiOC=CH$), 36.4 (d, $^1J_{CP}$ = 95.1 Hz, $PC(CH_3)_2$), 25.7 (s, $(CH_3)_3C=CH$), 21.6 (d, $^2J_{CP}$ = 1.4 Hz, $PCCH_3$), 21.5 (s, $PCCH_3$), 19.9 (s, $(CH_3)_3C=CH$), 1.1 (s, $POSi(CH_3)_3$) and 1.0 ppm (s, $OSi(CH_3)_3$). $^{31}P\{^1H\}$ NMR (162 MHz, $CDCl_3$): δ 31.9 ppm. $^{29}Si\{^1H\}$ NMR (79 MHz, $CDCl_3$): δ 22.4 (d, $^2J_{SiP}$ = 10.9 Hz, $POSiMe_3$) and 17.6 ppm (s, -$COSiMe_3$).

(2,6-Dimethyl-4-oxohept-5-en-2-yl)phosphinic acid, 2.

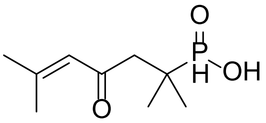

Method A: Hexamethyldisilazane (47.2 g, 292.5 mmol) and ammonium hypophosphite (23.4 g, 281.3 mmol) were stirred for 3 hours at 110°C until evolution of ammonia had finished. This resulted in a clear solution with traces of white solid that had sublimed on the top half of the flask. The formation of $HP(OSiMe_3)_2$ was confirmed by ^{31}P NMR (CD_2Cl_2): δ 139.9 (d, $^1J_{HP}$ = 178.7 Hz). The mixture was cooled to RT and phorone (31.1 g, 225.0 mmol) dissolved in CH_2Cl_2 (120 mL) was added and the mixture stirred at RT for 24 hours. After this time HCl (aq) (2.0 M, 100 mL) was added and the mixture stirred vigorously, which dissolved the white insoluble salts that had precipitated. The organic layer was then separated and dried ($MgSO_4$) to give a yellow oil. Yield = 42.7 g (93%). ^{1}H NMR (400 MHz, $CDCl_3$): δ 12.05 (1H, s, OH), 7.07 (1H, d, $^1J_{HP}$ = 565.0 Hz, P*H*), 6.01 (1H, s, C=C*H*), 2.62 (2H, d, $^2J_{HP}$ = 14.1 Hz, CH_2), 2.09 (3H, s, $=CCH_3$), 1.84 (3H, s, $=CCH_3$) and 1.17 ppm (6H, d, $^2J_{HP}$ = 17.8 Hz, $PC(CH_3)_2$). $^{13}C\{^1H\}$ NMR (101 MHz, $CDCl_3$): δ 198.7 (s, *C*=O), 156.6 (s, $(CH_3)_2C=CH$-), 124.1 (s, $(CH_3)_2C=CH$-), 50.0 (s, CH_2), 33.7 (d, $^1J_{CP}$ = 95.6 Hz, -$PC(CH_3)_2$), 27.8 (s, $(CH_3)_2C=CH$-), 20.6 (d, $^2J_{CP}$ = 56.6 Hz, $PC(CH_3)_2$) and 20.3 ppm (s, $(CH_3)_2C=CH$-). $^{31}P\{^1H\}$ NMR (162 MHz, $CDCl_3$): δ 46.8 ppm. HR-MS (+ve ESI): m/z (calc.) $[M+H]^+$ 205.0994; found 205.0984. Elem. Anal. Calcd for $C_9H_{17}O_3P$: C, 52.94; H, 8.39. Found: C, 52.37; H, 8.47. Single crystals of compound **2** were obtained after the yellow oil product was left to stand.

Method B: Ammonium hypophosphite (0.5 g, 6.0 mmol) in CH_2Cl_2 (20 mL) was cooled to 0 °C. *N,N*-Diisopropylethylamine (Hünigs base) (2.7 g, 3.5 mL, 21.1 mmol) was added and the mixture stirred at 0 °C for 1 hour. After this time trimethylsilyl chloride (2.3 g, 2.7 mL, 21.1 mmol) was added, HCl fumes were observed and the solution became colorless. After stirring for 4 hours, phorone (0.83 g, 0.92 mL, 6.0 mmol) in CH_2Cl_2 (1.0 mL) was added and stirred at 0 °C for 2 hours before being allowed to warm to RT. After 48 hours the mixture was passed through celite and washed with HCl (10%, aq) (20 mL × 2) followed by DI H_2O (20 mL). The organic fraction was separated and dried ($MgSO_4$), then the volatiles were removed *in vacuo* to give a yellow oil. Yield = 0.44 g (36%). The compound characterization was consistent with that obtained using method A.

Bis(trimethylsilyl) phosphonite, S1.

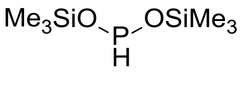

A small portion of $HP(OSiMe_3)_2$ was characterized by NMR spectroscopy, synthesized according to the procedure for compound **2**. ^{1}H NMR (400 MHz, d_8-tol): δ 7.62 (1H, d, $^1J_{HP}$ = 174.9 Hz, P*H*) and 0.16 ppm (18H, s, $OSiMe_3$). $^{13}C\{^1H\}$ NMR (101 MHz, d_8-tol): δ 1.1 ppm (d, $^1J_{SiC}$ = 2.4 Hz, $OSiMe_3$). ^{31}P NMR (162 MHz, d_8-tol): δ 141.7 ppm (d, 175.4 Hz). $^{29}Si\{^1H\}$ NMR (79 MHz, d_8-tol): δ 18.3 ppm (d, $^2J_{SiP}$ = 5.9 Hz).

Trimethylsilyl (2,6-dimethyl-4-oxohept-5-en-2-yl)phosphinate, 3.

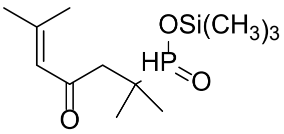

(2,6-Dimethyl-4-oxohept-5-en-2-yl)phosphinic acid, **2** (1.0 g, 4.90 mmol) was dissolved in CH_2Cl_2 (20 mL) and hexamethyldisilazane (0.90 g, 1.12 mL, 5.39 mmol) were added and the mixture stirred for 1 hour. After which time the volatiles were removed *in vacuo* to yield the product as an oil. Yield = 1.2 g (89%). ^{1}H NMR (400 MHz, $CDCl_3$): δ 7.09 (1H, d, $^1J_{HP}$ = 553.3 Hz, P*H*), 6.01 (1H, br s. (C*H*=C(CH_3)$_2$), 2.70 (1H, dd, $^2J_{HH}$ = 16.4 Hz, $^3J_{HP}$ = 11.4 Hz, C*H*$_2$), 2.49 (1H, t, $^2J_{HH}$ = 16.4 Hz, C*H*$_2$), 2.11 (3H, d, $^4J_{HH}$ = 1.1 Hz, CH=C(C*H*$_3$)$_2$), 1.85 (3H, d, $^4J_{HH}$ = 1.3 Hz, CH=C(C*H*$_3$)$_2$), 1.19 – 1.11 (6H, m, PC(C*H*$_3$)$_3$ and 0.28 ppm (9H, s, Si(C*H*$_3$)$_3$). ^{13}C{^{1}H} NMR (101 MHz, $CDCl_3$): δ 198.6 (d, $^3J_{CP}$ = 8.2 Hz, *C*=O), 156.3 (s, (CH_3)$_2$*C*=CH), 124.3 (s, (CH_3)$_2$C=*C*H), 50.0 (s, *C*H$_2$), 34.3 (d, $^1J_{CP}$ = 97.6 Hz, (CH_3)$_2$*C*P), 27.9 (s, (*C*H$_3$)$_2$C=CH), 20.9 (s, (*C*H$_3$)$_2$C=CH), 20.6 (s, (*C*H$_3$)$_2$CP), 20.4 (d, $^2J_{CP}$ = 1.6 Hz, (*C*H$_3$)$_2$CP) and 1.1 ppm (s, OSi(*C*H$_3$)$_3$). ^{31}P{^{1}H} NMR (162 MHz, $CDCl_3$): δ 35.9 ppm.

2,2,6,6-Tetramethyl-1,4-bis((trimethylsilyl)oxy)-2,3,6-trihydrophosphinine 1-oxide, 4.

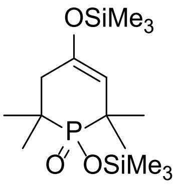

A small aliquot of the reaction mixture for the synthesis of compound **4** was extracted prior to acid hydrolysis and the volatiles were removed *in vacuo* giving a colorless oil. ^{1}H NMR (400 MHz, $CDCl_3$): δ 4.63 (1H, dd, $^2J_{HP}$ = 26.8 Hz, $^3J_{HH}$ = 1.8 Hz, C=C*H*), 2.32 (1H, ddd, $^2J_{HP}$ = 8.0 Hz, $^2J_{HH}$ = 17.0Hz, $^3J_{HH}$ = 1.5 Hz, C*H*$_2$) and 2.05 (1H, dd, $^2J_{HP}$ = 23.0 Hz, $^2J_{HH}$ = 17.0 Hz, C*H*$_2$), 1.30 (6H, d, $^3J_{HP}$ = 13.6 Hz, C(C*H*$_3$)$_3$), 1.16 (6H, d, $^3J_{HP}$ = 12.7 Hz, C(C*H*$_3$)$_3$), 0.30 (9H, s, OSi*Me*$_3$) and 0.18 ppm (9H, s, OSi*Me*$_3$). ^{13}C{^{1}H} NMR (101 MHz, $CDCl_3$): δ 146.6 (d, $^2J_{SiC}$ = 12.8 Hz, CH=*C*O), 115.3 (s, *C*H=CO), 45.1 (s, *C*H$_2$), 33.1 (d, $^1J_{CP}$ = 29.0 Hz, *C*(CH_3)$_2$) and 32.2 (d, $^1J_{CP}$ = 28.6 Hz, *C*(CH_3)$_2$), 26.4 (d, $^2J_{CP}$ = Hz, C(*C*H$_3$)$_2$), 26.1 (s, C(*C*H$_3$)$_2$), 23.2 – 23.1 (m, C(*C*H$_3$)$_2$), (1.6 (s, OSi*Me*$_3$) and 0.3 ppm (s, OSi*Me*$_3$). ^{31}P{^{1}H} NMR (162 MHz, $CDCl_3$): δ 49.4 ppm. ^{29}Si{^{1}H} NMR (79 MHz, $CDCl_3$): δ 20.6 (d, $^2J_{SiP}$ = 11.2 Hz, PO*Si*Me$_3$) and 18.1 ppm (s, CO*Si*Me$_3$).

2,2,6,6-Tetramethylphosphorinic acid, 5.

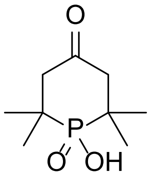

Method A: (2,6-Dimethyl-4-oxohept-5-en-2-yl)phosphinic acid (22.1 g, 108.0 mmol) and hexamethyldisilazane (20.9 g, 130.0 mmol) was stirred in degassed xylenes (500 mL) for 72 hours at 125 °C, giving a clear pale yellow solution. After this time the reaction mixture was cooled to RT. Subsequently HCl (aq, 2.0 M) (200 mL) was added and the mixture stirred vigorously. The aqueous layer was then separated and the organic layer was washed a second time with HCl (aq) (2.0 M, 50 mL). The combined aqueous layer was backwashed with CH_2Cl_2 (5 × 50 mL). The combined CH_2Cl_2 extracts were dried over $MgSO_4$ to give an off-white solid. The xylene phase was evaporated to dryness to give an off-white solid. Total combined yield from both organic phases = 12.9 g (58%). ^{1}H NMR (400 MHz, $CDCl_3$): δ 9.6 (1H, s, O*H*), 2.57 (4H, d, $^3J_{HP}$ = 15.7 Hz, C*H*$_2$) and 1.28 ppm (12H, d, $^3J_{HP}$ = 13.7 Hz, C*H*$_3$). ^{13}C{^{1}H} NMR (101 MHz, $CDCl_3$): δ 206.8 (d, $^3J_{CP}$ = 9.9 Hz, *C*=O), 53.7 (s, *C*H$_2$), 36.5 (d, $^1J_{CP}$ = 86.0 Hz, *C*(CH_3)$_2$ and 24.1 ppm (d, $^2J_{CP}$ = 1.8 Hz, *C*H$_3$). ^{31}P{^{1}H} NMR (162 MHz, $CDCl_3$): δ 58.1 ppm. HR-MS (+ve ESI): m/z (calc.) $[M+H]^+$ 205.0994; found 205.0985. Elem. Anal. Calcd for $C_9H_{17}O_3P$: C, 52.94; H, 8.39. Found: C, 52.40; H, 8.33. Single crystals of compound **5** were obtained by evaporation of a solution of the compound in acetone.

Method B: (*microwave conditions*) (2,6-Dimethyl-4-oxohept-5-en-2-yl)phosphinic acid (470 mg, 2.30 mmol) was dissolved in xylenes (4.5 mL) and placed inside a 10 mL microwave vial. Hexamethyldisilazane (743 mg, 0.96 mL, 4.6 mmol) was added and the mixture heated in a microwave reactor for 90 minutes at 220°C. The mixture was then hydrolyzed with HCl (aq, 2.0 M, 10 mL), the organic layer was separated and the aqueous portion extracted with CH_2Cl_2 (3 × 10 mL). The combined organics were evaporated to dryness. Yield = 188 mg (40%).

2,2,6,6-Tetramethylphosphaninic acid, 6.

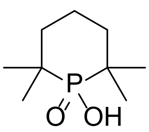

2,2,6,6-Tetramethylphosphorinic acid (8.5 g, 41.8 mmol) was suspended in diethylene glycol (120 mL). KOH (11.7 g, 209.1 mmol) and hydrazine monohydrate (65 wt%, aq) (16.1 g, 15.6 mL) were added and the mixture heated to 150 °C using a dean & stark trap to remove H_2O. The temperature was gradually increased to 210 °C and maintained for 16 hours. Upon cooling the mixture was diluted with DI H_2O (100 mL), acidified with HCl (aq, 2.0 M) to pH 1 and then extracted with CH_2Cl_2 (3 × 80 mL). The combined organic extracts were then washed with brine, dried and evaporated to give a white solid. Yield = 6.1 g (77 %). ^{1}H NMR (400 MHz, $CDCl_3$): δ 9.38 (1H,

br. s, O*H*), 1.67 – 1.51 (6H, m, C*H₂*) and 1.20 ppm (12H, d, $^3J_{HP}$ = 13.8 Hz, C*H₃*). $^{13}C\{^1H\}$ NMR (101 MHz, $CDCl_3$): δ 39.5 (s, C_q*C*H_2), 34.1 (d, $^1J_{CP}$ = 82.7 Hz, *C*$(CH_3)_2$), 23.7 (s, *C*H_3) and 19.2 ppm (d, $^3J_{CP}$ = 9.0 Hz, CH_2*C*H_2CH_2). $^{31}P\{^1H\}$ NMR (162 MHz, $CDCl_3$): δ 62.4 ppm. HR-MS (+ve ESI): m/z (calc.) $[M+H]^+$ 191.1201; found 191.1197. Elem. Anal. Calcd for $C_9H_{19}O_2P$: C, 56.83; H, 10.07. Found: C, 56.43; H, 9.87.

2,2,6,6-Tetramethylphosphaninic chloride, 7.

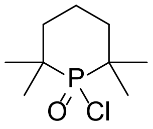

2,2,6,6-Tetramethylphosphaninic acid (1.25 g, 6.6 mmol) was dissolved in degassed CH_2Cl_2 (15 mL). DMF (5 drops) was added and oxalyl chloride (4.2 g, 32.8 mmol) was added dropwise into the reaction flask, during which time effervescence was observed. The reaction mixture was then stirred for 48 hours to give a clear yellow solution. The reaction mixture was dried *in vacuo* to obtain a brown oil. The product was extracted with pentane to give a brown oil. Yield = 1.1 g (81%). ^1H NMR (400 MHz, $CDCl_3$): δ 1.81 – 1.51 (6H, m, -*C*H_2*C*H_2*C*H_2-), 1.36 (6H, d, $^3J_{HP}$ = 15.8 Hz, C*H₃*) and 2.60 ppm (6H, d, $^3J_{HP}$ = 16.7 Hz, C*H₃*). $^{13}C\{^1H\}$ NMR (101 MHz, $CDCl_3$): δ 40.2 (d, $^1J_{CP}$ = 59.7 Hz, *C*$(CH_3)_2$), 39.3 (s, C_q*C*H_2), 25.8 (d, $^2J_{CP}$ = 1.9 Hz, *C*H_3), 23.5 (s, *C*H_3) and 18.8 ppm (d, $^3J_{CP}$ = 8.9 Hz, CH_2*C*H_2CH_2). $^{31}P\{^1H\}$ NMR (162 MHz, $CDCl_3$): δ 87.8 ppm. HR-MS (+ve ESI): m/z (calc.) $[M+H]^+$ 209.0862; found 209.0854. Elem. Anal. Calcd for $C_9H_{18}ClOP$: C, 51.80; H, 8.70. Found: C, 48.41; H, 8.52.

2,2,6,6-Tetramethylphosphinane (TMPhos), 8.

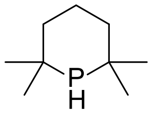

$LiAlH_4$ (4.1 g, 109.3 mmol) was suspended in Et_2O (120 mL) and cooled to 0 °C. 2,2,6,6-Tetramethylphosphaninic chloride (9.5 g, 45.5 mmol) in Et_2O (20 mL) was added dropwise over 15 minutes. After the addition the mixture was allowed to warm to RT and stirred for 16 hours. It was quenched by addition of NaOH (aq. 20%), (7 mL) at 0 °C, followed by addition of deionized H_2O (5 mL). Drying agent ($MgSO_4$) was added and the mixture was filtered. The Et_2O was removed by distillation under argon. Finally, the phosphine was purified *via* vacuum transfer giving a colourless liquid. Yield = 3.8 g (53%). ^1H NMR (400 MHz, $CDCl_3$): δ 2.60 (1H, dsept., $^1J_{PH}$ = 200.1 Hz, and $^3J_{HP}$ = 1.5 Hz, P*H*), 1.64 – 1.50 (4H, m, C*H₂*), 1.23 – 1.13 (2H, m, C*H₂*), 1.03 (6H, dd, $^3J_{PH}$ = 15.3 Hz and $^4J_{HH}$ = 1.2 Hz, C*H₃*) and 0.94 ppm (6H, d, $^3J_{PH}$ = 14.1 Hz, C*H₃*). $^{13}C\{^1H\}$ NMR (101 MHz, $CDCl_3$): δ 43.4 (d, $^2J_{CP}$ = 6.4 Hz, C_q*C*H_2), 32.6 (d, $^2J_{CP}$ = 13.7 Hz, *C*H_3). 27.7 (d, $^1J_{CP}$ = 8.9 Hz, *C*$(CH_3)_2$), 26.4 (d, $^2J_{CP}$ = 25.1 Hz, *C*H_3) and 20.9 ppm (s, -*C*H_2-). $^{31}P\{^1H\}$ NMR (162 MHz, $CDCl_3$): δ −9.1 ppm. HR-MS (+ve ESI): m/z (calc.) $[M+H]^+$ 159.1303; found 159.1297.

2,2,6,6-Tetramethylphosphinane 1-oxide, 9.

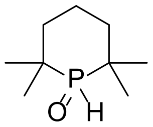

TMPhos (0.20 g, 1.26 mmol) was dissolved in CH_2Cl_2 (3 mL) and cooled to 0 °C. *m*-CPBA (70%, aq) (0.22 g, 1.26 mmol) in CH_2Cl_2 (5 mL) was added slowly. After 1 hour the mixture was allowed to warm to RT and stirred for an additional hour. After this time an aliquot of CH_2Cl_2 (10 mL) was added and the mixture washed with saturated $NaHCO_3$ (aq) (2 × 10 mL). The combined aqueous portion was then backwashed with CH_2Cl_2 (15 mL). The combined organic phases were dried over $MgSO_4$, filtered and evaporated to yield hydroscopic colourless platelets. Yield = 0.15 g (68%). ^1H NMR (400 MHz, $CDCl_3$): δ 6.18 (1H, d, $^1J_{HP}$ = 429.8 Hz, P*H*), 1.71 – 1.36 (6H, m, C*H₂*), 1.24 (6H, s, $^3J_{HP}$ = 15.3 Hz, C*H₃*) and 1.24 ppm (6H, d, $^3J_{HP}$ = 12.3 Hz, C*H₃*). $^{13}C\{^1H\}$ NMR (101 MHz, $CDCl_3$): δ 39.3 (d, $^2J_{CP}$ = 2.1 Hz, C_q*C*H_2), 33.7 (d, $^1J_{CP}$ = 58.0 Hz, *C*$(CH_3)_2$), 27.7 (d, $^2J_{CP}$ = 1.2 Hz, *C*H_3), 20.0 (s, *C*H_3) and 18.9 ppm (d, $^3J_{CP}$ = 7.6 Hz, CH_2*C*H_2CH_2). $^{31}P\{^1H\}$ NMR (162 MHz, $CDCl_3$): δ 59.2 ppm. HR-MS (+ve ESI): m/z (calc.) $[M+H]^+$ 175.1252; found 175.1242. Elem. Anal. Calcd for $C_9H_{19}OP$: C, 62.05; H, 10.99. Found: C, 62.58; H, 10.77.

2,2,6,6-Tetramethylphosphinane borane complex, 10.

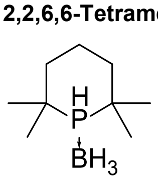

Borane dimethyl sulfide complex (2.0 M in THF, 2.0 mL, 4.0 mmol) was added to TMPhos (0.60 g, 3.80 mmol) in THF (10 mL). After stirring for 2 hours the volatiles were removed *in vacuo* to yield a white solid. Yield = 0.6 g (92%). ^1H NMR (400 MHz, $CDCl_3$): δ 3.98 (1H, d of m, $^1J_{HP}$ = 318.1 Hz, P*H*), 1.83 – 1.55 (4H, m, C*H₂*CH_2C*H₂*), 1.50 – 1.40 (2H, m, CH_2C*H₂*CH_2), 1.20 (6H, d, $^3J_{HP}$ = 14.2 Hz, C*H₃*), 1.19 (6H, d, $^3J_{HP}$ = 15.5 Hz, C*H₃*) and 0.43 ppm (3H, br. q, $^1J_{HB}$ = 99.2 Hz, B*H₃*). $^{13}C\{^1H\}$ NMR (101 MHz, $CDCl_3$): δ 41.8 (d, $^2J_{CP}$

= 5.2 Hz, C_qCH_2), 30.2 (d, $^2J_{CP}$ = 1.7 Hz, CH_3), 28.0 (d, $^1J_{CP}$ = 29.4 Hz, $C(CH_3)_2$), 22.0 (d, $^2J_{CP}$ = 7.4 Hz, CH_3) and 19.4 ppm (d, $^3J_{CP}$ = 4.8 Hz, $CH_2CH_2CH_2$). $^{31}P\{^1H\}$ NMR (162 MHz, $CDCl_3$): δ 34.3 ppm (q, $^1J_{HB}$ = 46.1 Hz). HR-MS (+ve ESI): m/z (calc.) $[M+H]^+$ 173.1630; found 173.1644. Elem. Anal. Calcd for $C_9H_{22}BP$: C, 62.83; H, 12.89. Found: C, 61.50; H, 12.60.

1-Chloro-2,2,6,6-tetramethylphosphinane, 11.

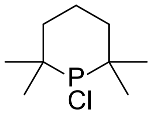

TMPhos (1.70 g, 10.7 mmol) was dissolved in THF (5.0 mL) and methyl trichloroacetate (1.91 g, 10.7 mmol) was added. The mixture was stirred for 16 hours whereupon the by-product methyl 2,2-dichloroacetate was removed *in vacuo* to give a colorless oil. Yield = 1.5 g (72%). ^1H NMR (400 MHz, $CDCl_3$): δ 1.79 – 1.65 (2H, m, C*H₂*), 1.64 – 1.47 (2H, m, CH_2), 1.39 – 1.29 (2H, m, CH_2), 1.18 (6H, d, $^3J_{HP}$ = 18.1 Hz, CH_3) and 1.15 ppm (6H, d, $^3J_{HP}$ = 11.5 Hz, CH_3). $^{13}C\{^1H\}$ NMR (101 MHz, $CDCl_3$): δ 34.2 (br. s, C_qCH_2), 33.3 (d, $^1J_{CP}$ = 32.7 Hz, $C(CH_3)_2$), 30.1 (d, $^2J_{CP}$ = 8.4 Hz, CH_3), 25.5 (d, $^2J_{CP}$ = 33.6 Hz, CH_3) and 19.8 ppm (s, $-CH_2CH_2CH_2-$). $^{31}P\{^1H\}$ NMR (162 MHz, $CDCl_3$): δ 130.5 ppm. HR-MS (+ve ESI): m/z (calc.) $[M+H]^+$ 193.0913; found 193.0903. Elem. Anal. Calcd for $C_9H_{18}ClP$: C, 56.11; H, 9.42. Found: C, 54.53; H, 9.11.

2,2,6,6-Tetramethylphosphinane 1-selenide, 12.

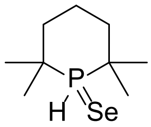

TMPhos (19.1 mg, 0.12 mmol) and Se powder (16.0 mg, 0.20 mmol) were stirred in toluene (2 mL) for 30 mins at RT. After this time the mixture was filtered and the volatiles removed *in vacuo* to give a white solid. Yield = *quantitative*. ^1H NMR (400 MHz, $CDCl_3$): δ 5.54 (1H, d, $^1J_{PH}$ = 411.0 Hz, P*H*), 2.09 – 1.91 (2H, m, CH_2), 1.76 – 1.52 (4H, m, CH_2) and 1.21 ppm (12H, d, $^3J_{HP}$ = 17.8 Hz, CH_3).$^{13}C\{^1H\}$ NMR (101 MHz, $CDCl_3$): δ 40.5 (s, C_qCH_2), 33.5 (d, J_{CP} = 35.3 Hz, $C(CH_3)_2$), 29.8 (s, CH_3), 20.6 (d, J_{CP} = 4.6 Hz, CH_3) and 19.1 ppm (d, J_{CP} = 6.5 Hz, $CH_2CH_2CH_2$). $^{31}P\{^1H\}$ NMR (162 MHz, $CDCl_3$): δ 54.6 ppm (s, $^1J_{PSe}$ = 717.2 Hz)*. $^{77}Se\{1H\}$ NMR (76.3 MHz, $CDCl_3$): δ −517.9 ppm (d, $^1J_{SeP}$ = 717.2 Hz). *Coupling constant is based on the P-^{77}Se satellite (92.5:7.5) which is consistent with selenium natural abundance.

[(acac)Rh(CO)(TMPhos)], 13.

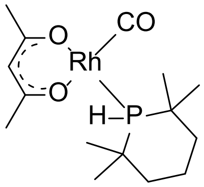

$[(acac)Rh(CO)_2]$ (176 mg, 0.68 mmol) was dissolved in CH_2Cl_2 (5 mL) and TMPhos (108 mg, 0.68 mmol) in CH_2Cl_2 (4 mL) was added slowly with stirring. The solution turned yellow with evolution of gas observed. After stirring for 1 hour the mixture was filtered and heptane (5 mL) was added. The mixture was reduced in volume to 3 mL and a yellow solid precipitated, which was filtered, washed with heptane and dried *in vacuo*. Yield = 140 mg (53%). ^1H NMR (400 MHz, CD_2Cl_2): δ 5.48 (1H, s, *acac* C*H*), 4.05 (1H, d, $^1J_{HP}$ = 338.6 Hz, P*H*), 2.05 (3H, s, *acac* CH_3), 1.89 (3H, s, *acac* CH_3), 1.88 – 1.61 (4H, m, CH_2), 1.59 – 1.46 (2H, m, CH_2), 1.30 (6H, d, $^3J_{HP}$ = 14.7 Hz, CH_3) and 1.24 ppm (6H, d, $^3J_{HP}$ = 17.0 Hz, CH_3). $^{13}C\{^1H\}$ NMR (101 MHz, CD_2Cl_2): δ 190.5 (dd, $^1J_{RhC}$ = 74.6 Hz, $^2J_{RhP}$ = 25.6 Hz, Rh*C*O), 188.0 (d, $^2J_{RhC}$ = 0.5 Hz, *acac* *C*O), 185.8 (s, *acac* *C*O), 100.7 (d, $^3J_{RhC}$ = 2.5 Hz, *acac* *C*H), 43.5 (dd, $^2J_{CP}$ = 4.3 Hz, $^3J_{CRh}$ = 0.7 Hz, $CH_2CH_2CH_2$), 32.8 (dd, $^1J_{CP}$ = 25.4 Hz, $^2J_{CRh}$ = 0.8 Hz, $C(CH_3)_2$), 32.3 (d, $^2J_{CP}$ = 5.1 Hz, $C(CH_3)_2$), 28.0 (dd, $^3J_{RhC}$ = 5.3 Hz, $^4J_{CP}$ = 1.1 Hz, *acac* CH_3), 27.4 (s, *acac* CH_3), 24.9 (d, $^2J_{CP}$ = 8.6 Hz, $C(CH_3)_2$) and 20.5 ppm (d, $^3J_{CP}$ = 4.5 Hz, $CH_2CH_2CH_2$). $^{31}P\{^1H\}$ NMR (162 MHz, CD_2Cl_2): δ 62.7 ppm (d, $^1J_{RhP}$ = 165.2 Hz).

[(acac)Rh(CO)(HP^{t}Bu₂)], S2.

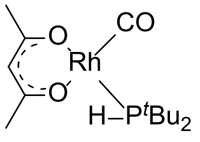

$[(acac)Rh(CO)_2]$ (176 mg, 0.68 mmol) was dissolved in CH_2Cl_2 (5 mL) and HP^{t}Bu$_2$ (100 mg, 0.68 mmol) in CH_2Cl_2 (4 mL) was added slowly with stirring. The solution turned yellow with evolution of gas observed. After stirring for 1 hour the mixture was filtered and heptane (5 mL) was added. The volatiles were removed *in vacuo* to give a yellow solid. Yield = 150 mg (58%). ^1H NMR (400 MHz, $CDCl_3$): δ 5.47 (1H, s, *acac* C*H*), 4.22 (1H, d, $^1J_{PH}$ = 342.1 Hz, P*H*), 2.04 (3H, s, *acac* CH_3), 1.89 (3H, s, *acac* CH_3) and 1.43 ppm (18H,

d, $^3J_{HP}$ = 14.5 Hz, ^{t}Bu). $^{13}C\{^1H\}$ NMR (101 MHz, $CDCl_3$): δ 190.5 (dd, $^1J_{CRh}$ = 75.3 Hz, $^2J_{RhP}$ = 25.0 Hz, Rh*C*O), 188.1 (d, $^2J_{CRh}$ = 0.6 Hz, *acac* CO), 185.7 (s, *acac* CO), 100.6 (d, $^3J_{CRh}$ = 2.5 Hz, *acac* CH), 34.3 (dd, $^1J_{CP}$ = 23.1 Hz, $^2J_{CRh}$ = 0.9 Hz, *C*(CH_3)$_3$), 31.3 (d, $^2J_{CP}$ = 4.3 Hz, C(CH_3)$_3$), 28.0 (dd, $^3J_{CRh}$ = 5.1 Hz, $^4J_{CP}$ = 1.1 Hz, *acac* CH_3), 27.4 ppm (s, *acac* CH_3). $^{31}P\{^1H\}$ NMR (162 MHz, $CDCl_3$): δ 76.5 ppm (br. s).

[(TMPhos)$_3$Pd$^{(0)}$], 14.

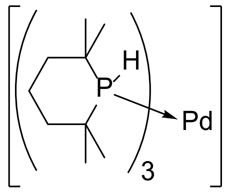

TMPhos in acetone (1 mL) was cooled to 0 °C. A deep red solution of allyl(cyclopentadienyl)palladium(II) in acetone (1 mL) was added dropwise with a colour change to yellow. After the addition the mixture was stirred for 30 mins. The yellow solid that had formed was filtered and dried *in vacuo*. Yield = 90 mg (67%). ^{1}H NMR (400 MHz, C_6D_6): δ 4.15 (3H, d, $^1J_{PH}$ = 255.7 Hz, P*H*), 1.84 – 1.63 (9H, m, C*H$_2$*), 1.56 – 1.46 (3H, m, C*H$_2$*), 1.44 – 1.33 (6H, m, C*H$_2$*) and 1.35 – 1.18 ppm (36H, m, C*H$_3$*). $^{13}C\{^1H\}$ NMR (101 MHz, C_6D_6): δ 43.8 (s, C_qCH_2), 33.6 (s, CH_3), 32.1 (s, CH_3), 26.0 (d, $^1J_{CP}$ = 9.9 Hz, *C*(CH_3)$_2$) and 21.1 ppm (s, $CH_2CH_2CH_2$). ^{31}P NMR (162 MHz, C_6D_6): δ 42.1 ($^1J_{PH}$ = 256 Hz) ppm. Elem. Anal. Calcd for $C_{27}H_{57}P_3Pd$: C, 55.81; H, 9.89. Found: C, 55.54; H, 9.49.

Complex 15.

A solution of **14** kept at RT formed red single crystals of bimetallic complex **15**, which were analysed by single crystal X-ray diffraction.

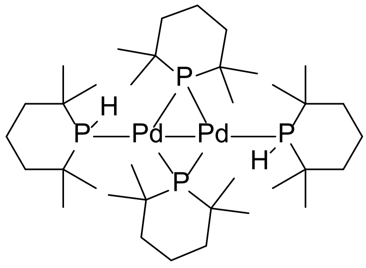

1-([1,1'-Biphenyl]-2-yl)-2,2,6,6-tetramethylphosphinane, ^{TMPhos}(Biphenyl), 16.

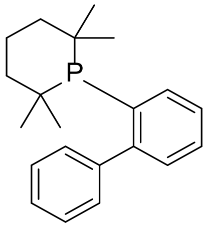

TMPhos (0.37 g, 2.23 mmol), [1,1'-biphenyl]-2-yl dihydrobenzo [2] (0.50 g, 1.65 mmol), DBU (0.50 g, 3.31 mmol), Pd(OAc)$_2$ (2.0 mg, 0.01 mmol) were weighed and dissolved in toluene (5 mL) and heated at 110 °C for 16 hours. Toluene (5 mL) was added to the resulting solution, and it was washed three times with degassed DI water and dried over $MgSO_4$. The filtrate was evacuated to dryness *in vacuo* to give a yellow semi-solid. The crude product was recrystallized from MeOH at −20 °C (freezer) over 1 hour. The resulting crystals were dried *in vacuo. Yield* = 0.22 g (43%). ^{1}H NMR (400 MHz, $CDCl_3$): δ 7.97 (1H, dt, $^3J_{HH}$ = 7.4 Hz, $^4J_{HP}$ = 1.6 Hz, Ar*H*), 7.38 – 7.19 (8H, m, ArH), 1.95 – 1.82 (2H, m, C_qC*H$_2$*), 1.77 – 1.64 (2H, m, -$CH_2CH_2CH_2$-), 1.48 – 1.37 (2H, m, C_qC*H$_2$*), 1.11 (6H, d, C*H$_3$*, $^3J_{PH}$ = 19.2 Hz) and 0.87 ppm (6H, d, $^3J_{PH}$ = 9.4 Hz, C*H$_3$*). $^{13}C\{^1H\}$ NMR (101 MHz, $CDCl_3$): δ 151.9 (d, $^2J_{CP}$ = 33.9 Hz, Ar*C$_q$*), 144.0 (d, $^3J_{CP}$ = 7.9 Hz, Ar*C$_q$*), 136.0 (d, $^1J_{CP}$ = 31.4 Hz, Ar*C$_q$*), 134.9 (d, $^3J_{CP}$ = 4.2 Hz, Ar*C*H), 130.9 (d, $^2J_{CP}$ = 4.2 Hz, Ar*C*H), 130.8 (d, $^3J_{CP}$ = 5.1 Hz, ArCH), 128.3 (s, ArCH), 127.3 (s, Ar*C*H), 126.5 (s, Ar*C*H), 126.1 (s, Ar*C*H), 37.9 (s, C_qCH_2), 31.3 (d, $^2J_{CP}$ = 35.7 Hz, CH_3), 30.3 (d, $^2J_{CP}$ = 6.3 Hz, CH_3), 30.0 (d, $^1J_{CP}$ = 18.4 Hz, *C*(CH_3)$_2$) and 20.4 ppm (s, $CH_2CH_2CH_2$). $^{31}P\{^1H\}$ NMR (162 MHz, $CDCl_3$): δ −2.4 ppm. HR-MS (+ve ESI): m/z (calc.) $[M+H]^+$ 311.1929; found 311.1922. Elem. Anal. Calcd for $C_{21}H_{27}P$: C, 81.25; H, 8.77. Found: C, 80.31; H, 8.27.

1,3-Bis(2,2,6,6-tetramethylphosphinan-1-yl)propane, BTMPPr, 17.

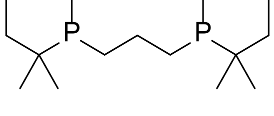

TMPhos (1.0 g, 6.32 mmol) was dissolved in THF (15 mL). ^{n}BuLi (2.0 M in cyclohexane, 3.5 mL, 6.95 mmol) was added dropwise at −78 °C and allowed to warm to RT and stirred for 1 hour. After this time 1,3-dibromopropane (0.57 g, 2.84 mmol) was dissolved in THF and added dropwise at −78 °C and allowed to warm to RT and stirred for 16 hours. After this time the volatiles were removed *in vacuo* to give a dark olive-green oil. The crude product was extracted with pentane (2 × 20 mL), passed through a glass filter and the volatiles removed *in vacuo* to give an off-white waxy solid. The product was then dissolved in CH_2Cl_2 (10 mL) and filtered through a plug of silica. The volatiles were removed *in vacuo* to yield a translucent oil. Yield = 0.96 g (95%). ^{1}H NMR (400 MHz, $CDCl_3$): δ 2.08 – 1.33 (18H, m, CH_2), 1.10 (12H, d, $^{3}J_{HP}$ = 17.0 Hz, CH_3) and 1.04 ppm (12H, d, $^{3}J_{HP}$ = 6.7 Hz, CH_3). ^{13}C{^{1}H} NMR (101 MHz, $CDCl_3$): δ 40.2 (br s, C_qCH_2), 31.5 (d, $^{2}J_{CP}$ = 26.4 Hz, CH_3), 29.1 (d, $^{2}J_{CP}$ = 14.7 Hz, $C(CH_3)_2$), 28.8 (t, $^{2}J_{CP}$ = 22.8 Hz, -CH_2CH_2P), 26.2 (br s, CH_3), 22.7 (dd, $^{1}J_{CP}$ = 22.4 Hz, $^{3}J_{CP}$ = 12.6 Hz, CH_2P) and 20.4 ppm (s, $C_qCH_2CH_2CH_2$). ^{31}P{^{1}H} NMR (162 MHz, $CDCl_3$): δ 8.04 ppm. HR-MS (+ve ESI): m/z (calc.) $[M+H]^+$ 357.2840; found 357.2834. Elem. Anal. Calcd for $C_{21}H_{42}P_2$: C, 70.75; H, 11.87. Found: C, 70.56; H, 11.84.

1,4-Bis(2,2,6,6-tetramethylphosphinan-1-yl)butane, BTMPBu, 18.

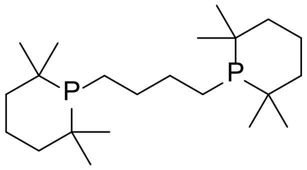

TMPhos (0.50 g, 3.16 mmol) in THF (5 mL) was cooled to −78 ˚C. *n*-BuLi (2.0 M in CH, 1.58 mL, 3.16 mmol) was added dropwise, and the mixture slowly warmed to RT for 1 h. The mixture was cooled down again to −78 ˚C, then 1,4-dibromobutane (0.325 g, 1.51 mmol) in THF (3 mL) was added dropwise. After the addition, the solution was stirred for 16 h at RT. The volatiles were then removed *in vacuo*, and the crude product was dissolved in CH_2Cl_2 (5 mL) and washed with H_2O (2 × 5 mL) and dried ($MgSO_4$). Concentration *in vacuo* yielded a white solid. Yield = 0.34 g (61%). ^{1}H NMR (400 MHz, $CDCl_3$): δ 1.66 – 1.55 (4H, m, CH_2), 1.55 – 1.34 (16H, m, CH_2), 1.11 (12H, d, $^{3}J_{HP}$ = 17.0 Hz, CH_3) and 1.04 ppm (12H, d, $^{3}J_{HP}$ = 6.7 Hz, CH_3). ^{13}C{^{1}H} NMR (101 MHz, $CDCl_3$): δ 40.1 (br. s, $PC(CH_3)_2CH_2$), 31.6 (dd, $^{2}J_{CP}$ = 16.2 Hz, $^{3}J_{CP}$ = 8.7 Hz, PCH_2CH_2), 31.5 (d, $^{2}J_{CP}$ = 22.8 Hz, CH_3), 29.1 (d, $^{1}J_{CP}$ = 15.1 Hz, $PC(CH_3)_2$), 26.2 (br. s, CH_3), 20.5 (s, $CH_2CH_2CH_2$) and 20.3 ppm (d, $^{1}J_{CP}$ = 22.0 Hz, PCH_2). ^{31}P{^{1}H} NMR (162 MHz, $CDCl_3$): δ 9.30 ppm. HR-MS (+ve ESI): m/z (calc.) $[M+H]^+$ 371.2997; found 371.2988. Elem. Anal. Calcd for $C_{22}H_{44}P_2$: C, 71.31; H, 11.97. Found: C, 71.09; H, 11.69.

1,1'-(9,9-Dimethyl-9H-xanthene-4,5-diyl)bis(2,2,6,6-tetramethylphosphinane), ^{TMPhos}(Xantphos), 19.

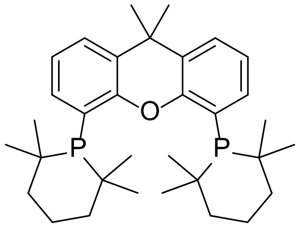

9,9-Dimethylxanthene (0.200 g, 0.95 mmol) and TMEDA (0.36 mL, 0.28 g, 2.40 mmol) were dissolved in heptane (6 mL). A solution of *n*-BuLi in cyclohexane (1.6 M, 1.7 mL, 2.90 mmol) was added dropwise at RT. The reaction mixture was stirred for 16 hours, then TMPhos (0.58 g, 3.00 mmol) was added dropwise at RT. The volatiles was removed *in vacuo*, and the residue was dissolved in CH_2Cl_2. This solution was washed with H_2O (2 × 3 mL), dried ($MgSO_4$) , and evaporated to dryness. The resulting solid was washed with pentane (2 × 3 mL) and dried *in vacuo* to afford a white solid. Yield = 57 mg (11%). ^{1}H NMR (400 MHz, $CDCl_3$): δ 7.61 (2H, d, $^{3}J_{HP}$ = 7.3 Hz, Ar*H*), 7.34 (2H, dd, $^{3}J_{HH}$ = 7.7, $^{4}J_{HH}$ = 1.5 Hz, Ar*H*), 7.00 (2H, t, $^{3}J_{HH}$ = 7.6 Hz, Ar*H*), 1.90 – 1.77 (6H, m, CH_2), 1.72 – 1.63 (2H, m, CH_2), 1.57 (6H, s, $ArC(CH_3)_2$), 1.49 – 1.36 (4H, m, CH_2), 1.41 (12H, m, CH_3) and 0.97 – 0.92 ppm (m, 12H, $PC(CH_3)_2$). ^{13}C{^{1}H} NMR (101 MHz, $CDCl_3$): δ 155.0 (t, $|^{2}J_{CP} + {}^{4}J_{CP}|$ = 11.8 Hz, OC_q), 132.9 (t, $|^{2}J_{CP} + {}^{6}J_{CP}|$ = 1.7 Hz, ArCH), 130.0 (t, $|^{3}J_{CP} + {}^{5}J_{CP}|$ = 1.5 Hz, ArC_q), 126.7 (dd, $^{1}J_{CP}$ = 22.5 Hz, $^{5}J_{CP}$ = 17.1 Hz, $PArC_q$), 125.6 (s, ArCH), 121.6 (d, $^{3}J_{CP}$ = 0.8 Hz, ArCH), 37.5 (s, $PC(CH_3)_2CH_2$), 34.8 (t, $^{4}J_{CP}$ = 1.4 Hz, $ArC(CH_3)_2$), 31.9 (s, $ArC(CH_3)_2$), 30.6 (t, $^{2}J_{CP}$ = 19.6 Hz, $PC(CH_3)_2$), 30.3 (t, $^{2}J_{CP}$ = 5.9 Hz, $PC(CH_3)_2$), 29.7 (m, $PC(CH_3)_2$) and 20.6 ppm (s, $CH_2CH_2CH_2$). ^{31}P{^{1}H} NMR (162 MHz, $CDCl_3$): δ −8.50 (s) ppm. HR-MS (+ve ESI): m/z (calc.) $[M+H]^+$ 523.3259; found 523.3243. Elem. Anal. Calcd for $C_{33}H_{48}OP_2$: C, 75.83; H, 9.26. Found: C, 75.86; H, 9.28.

1,2-Bis((2,2,6,6-tetramethylphosphinan-1-yl)methyl)benzene, BTMPX, 20.

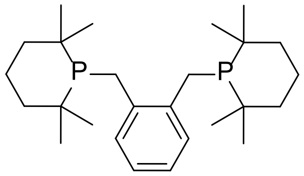

Method A: ^{n}BuLi (2.0 M in cyclohexane, 1.9 mL, 3.84 mmol) was slowly added to 2,2,6,6-tetramethylphosphinane borane complex (0.6 g, 3.49 mmol) at −78 °C and allowed to warm to RT. After stirring for 1 hour, 1,5-dihydrobenzo[e][1,3,2]dioxathiepine 3,3-dioxide[3] was dissolved in THF (2.5 mL) and added dropwise into the reaction mixture at −78 °C , the reaction mixture was allowed to warm to RT and stirred for 16 hours. The resulting yellow solution was dried *in vacuo* to give a yellow-white solid. The crude product was washed with methanol and dried *in vacuo.* Pyrrolidine (5 mL) was added and then the mixture was heated to 55 °C for 16 hours. The volatiles were removed *in vacuo* to give a pale yellow solid. The product was then recrystallized from MeOH to yield pale yellow crystals. Yield = 0.49 g (75 %). ^{1}H NMR (400 MHz, $CDCl_3$): δ 7.44 – 7.37 (2H, m, ArH), 7.09 – 7.01 (2H, m, ArH), 3.09 (4H, s, C*H*$_2$), 1.62 – 1.40 (12H, m, CH_2), 1.18 (12H, d, $^{3}J_{HP}$ = 4.88 Hz, C*H*$_3$) and 0.92 ppm (12H, d, $^{3}J_{HP}$ = 17.5 Hz, C*H*$_3$). ^{13}C{^{1}H} NMR (101 MHz, $CDCl_3$): δ 138.1 (dd, $^{2}J_{CP}$ = 7.5 Hz, $^{3}J_{CP}$ = 2.9 Hz, ArC$_q$), 131.2 (d, $^{3}J_{CP}$ = 40.4 Hz, Ar*C*), 125.6 (d, $^{4}J_{CP}$ = 1.7 Hz, Ar*C*), 41.8 (br. s., *C*H$_2$CH$_2$CH$_2$), 32.0 (d, $^{2}J_{CP}$ = 25.8 Hz, *C*H$_3$), 30.1 (d, $^{1}J_{CP}$ = 17.8 Hz, *C*(CH$_3$)$_2$), 26.1 (dd, $^{1}J_{CP}$ = 26.8 Hz, $^{4}J_{CP}$ = 7.4 Hz, *C*H$_2$P), 24.9 (br. s., *C*H$_3$) and 20.4 ppm (d, $^{3}J_{CP}$ = 1.8 Hz, CH$_2$*C*H$_2$CH$_2$). ^{31}P{^{1}H} NMR (162 MHz, $CDCl_3$): δ 10.2 ppm. HR-MS (+ve ESI): m/z (calc.) $[M+H]^+$ 419.2997; found 419.2993. Elem. Anal. Calcd for $C_{26}H_{44}P_2$: C, 74.61; H, 10.60. Found: C, 74.03; H, 10.42. Single crystals suitable for X-ray crystallography of compound **18** were obtained from a saturated solution of the compound in MeOH.

Method B: TMPhos (0.90 g, 5.69 mmol) and α,α-dibromo-*o*-xylene (0.69 g, 2.61 mmol) were mixed together in MeOH (20 mL). After stirring for 16 hours, NEt_3 (0.60 g, 0.78 mL, 5.93 mmol) was added. The solvent was evaporated *in vacuo*, giving a white solid as the crude product which was washed with MeOH (3 × 10 mL). Yield = 0.34 g (31%). NMR spectra were consistent with that obtained using Method **A**. HR-MS (+ve ESI): m/z (calc.) $[M+H]^+$ 419.2997; found 419.2990. Elem. Anal. Calcd for $C_{26}H_{44}P_2$: C, 74.61; H, 10.60. Found: C, 74.60; H, 10.42.

1,3-Bis((2,2,6,6-tetramethylphosphinan-1-yl)methyl)benzene, ^{TMPhos}(PCP), 21.

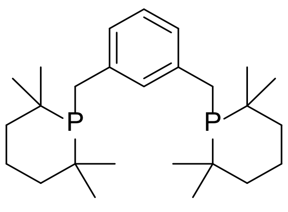

TMPhos (0.90 g, 5.69 mmol) and α,α-dibromo-*m*-xylene (0.69 g, 2.61 mmol) were mixed together in MeOH (20 mL). After stirring for 16 hours, NEt_3 (0.60 g, 0.78 mL, 5.93 mmol) was added giving a white precipitate which was filtered and dried. A second crop was obtained by concentrating the filtrate and placing it in the freezer at −20 °C. The combined crops were dissolved in CH_2Cl_2, washed with water, dried over $MgSO_4$ and dried in vacuo. Yield = 0.87 g (79%). ^{1}H NMR (400 MHz, $CDCl_3$): δ 7.30 (1H, br s, Ar*H*), 7.14 (3H, br s, Ar*H*), 2.80 (4H, d, $^{2}J_{HP}$ = 1.9 Hz, C*H*$_2$), 1.66 – 1.34 (12H, m, C*H*$_2$), 1.13 (12H, d, $^{3}J_{HP}$ = 5.3 Hz, C*H*$_3$), 0.93 (12H, d, $^{3}J_{HP}$ = 17.4 Hz, C*H*$_3$). ^{13}C{^{1}H} NMR (101 MHz, $CDCl_3$): δ 140.5 (d, $^{3}J_{CP}$ = 9.8 Hz, Ar*C*), 130.7 (t, $^{4}J_{CP}$ = 7.2 Hz, Ar*C*), 128.3 (s, Ar*C*), 126.9 (dd, $^{2}J_{CP}$ = 8.1 Hz, $^{4}J_{CP}$ = 2.1 Hz, Ar*C*$_q$), 41.1 (br s, *C*H$_2$), 31.6 (d, $^{1}J_{CP}$ = 25.8 Hz, *C*(CH$_3$)$_2$), 29.8 (d, $^{2}J_{CP}$ = 16.9 Hz, *C*H$_3$), 27.7 (d, $^{1}J_{CP}$ = 25.2 Hz, *C*H$_2$P), 25.4 (br s, *C*H$_3$) and 20.4 ppm (d, $^{3}J_{CP}$ = 1.6 Hz, CH$_2$CH$_2$CH$_2$). ^{31}P{^{1}H} NMR (162 MHz, $CDCl_3$): δ 16.7 ppm. HR-MS (+ve ESI): m/z (calc.) $[M+H]^+$ 419.2297; found 419.2290. Elem. Anal. Calcd for $C_{26}H_{44}P_2$: C, 74.61; H, 10.60. Found: C, 73.81; H, 10.43.

2,6-Bis((2,2,6,6-tetramethylphosphinan-1-yl)methyl)pyridine, ^{TMPhos}(PNP), 22.

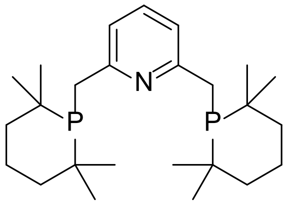

Method A: 2,6-Lutidine (0.13 g, 0.14 mL, 1.2 mmol) was dissolved in Et_2O (2 mL) and the mixture cooled to 0 °C. ^{n}BuLi (2.0 M in hexanes, 1.4 mL, 2.8 mmol) was added dropwise. The orange mixture was heated at 40 °C for 20 hours. After this time it was cooled to −78 °C and 1-chloro-2,2,6,6-tetramethylphosphinane (0.50 g, 2.6 mmol) in Et_2O (1 mL) was added dropwise. The dark brown mixture was allowed to warm to RT. MeOH (6 mL) was added then the volatiles were removed *in vacuo*. Et_2O was added (4 mL) and the mixture was then passed through a glass filter, then evaporated to yield a crude orange solid. ^{31}P{^{1}H} NMR (162 MHz, $CDCl_3$): δ 17.4 ppm. HR-MS (+ve ESI): m/z (calc.) $[M+H]^+$ 420.2949; found 420.2941.

Method B: *n*-BuLi (2.0 M in cyclohexane) was added dropwise to TMPhos (0.40 g, 2.53 mmol) in THF (4 mL) at −78 °C and stirred at RT for 1 hour. After this time the mixture was again cooled to −78 °C and 2,6-bis(tosyloxymethyl)pyridine[4] (0.51 g, 1.14 mmol) was added dropwise. The mixture was then allowed to warm to RT and stirred for 16 hours. After this time the mixture was evaporated to dryness, washed with MeOH (3 × 10 mL) and dried *in vacuo*. Yield = 0.27 g (57%). ^{1}H NMR (400 MHz, $CDCl_3$): δ 7.45 (1H, t, $^{3}J_{HH}$ = 7.7 Hz, Ar*H*), 7.21 (2H, d, $^{3}J_{HH}$ = 7.7 Hz, Ar*H*), 2.99 (4H, d, $^{2}J_{CP}$ = 2.8 Hz, C*H*$_2$P), 1.65 – 1.37 (12H, m, C*H*$_2$), 1.14 (12H, d, $^{3}J_{HP}$ = 6.2 Hz, C*H*$_3$) and 0.97 ppm (12H, d, $^{3}J_{HP}$ = 17.5 Hz, C*H*$_3$). ^{31}P{^{1}H} NMR (162 MHz, $CDCl_3$): δ 17.4 ppm. ^{13}C{^{1}H} NMR (101 MHz, $CDCl_3$): δ 160.4 (d, $^{2}J_{CP}$ = 12.0 Hz, Ar*C*$_q$), 136.2 (s, Ar*C*), 120.9 (dd, $^{3}J_{CP}$ = 9.5 Hz, $^{5}J_{CP}$ = 1.3 Hz, Ar*C*), 40.4 (br. s, *C*CH$_2$), 31.2 (d, $^{2}J_{CP}$ = 27.8 Hz, *C*H$_3$), 30.9 (d, $^{1}J_{CP}$ = 25.6 Hz, *C*H$_2$P) 29.8 (d, $^{1}J_{CP}$ = 16.3 Hz, *C*(CH$_3$)$_2$), 26.2 (br. s, *C*H$_3$) and 20.4 ppm (d, $^{3}J_{CP}$ = 0.9 Hz, CH$_2$*C*H$_2$CH$_2$).

HR-MS (+ve ESI): m/z (calc.) $[M+H]^+$ 420.2949; found 420.2945. Elem. Anal. Calcd for $C_{25}H_{43}NP_2$: C, 71.57; H, 10.33; N, 3.34. Found: C, 68.82; H, 9.27; N, 3.19.

[(BTMPX)PdCl$_2$], S3.

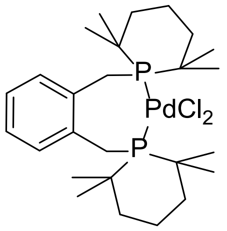

BTMPX (0.20 g, 0.50 mmol) and [Pd(dba)$_2$] (0.27 g, 0.50 mmol) were mixed together in CH_2Cl_2 (5 mL). After 90 minutes HCl (2.0 M in Et_2O, 0.5 mL) was added and air was introduced into the reaction flask. After stirring for 16 hours the mixture was passed through a glass filter and reduced in volume to ~3 mL. Et_2O (10 mL) was added which precipitated a yellow solid which was filtered and washed with Et_2O (2 × 10 mL) and then dried *in vacuo*. Yield = 130 mg (44%). ^{1}H NMR (400 MHz, $CDCl_3$): δ 7.61 – 7.16 (2H, m, Ar*H*), 7.16 – 6.72 (2H, m, Ar*H*), 3.89 – 3.05 (4H, m, C*H$_2$*), 3.35 –2.38 (4H, m, C*H$_2$*), 2.31 – 0.06 ppm (24H, m). ^{13}C{^{1}H} NMR (101 MHz, $CDCl_3$): δ 133.9 (br. s, Ar*C*$_q$), 133.3 (br. s, Ar*C*H), 127.6 (br. s, Ar*C*H), 40.4 (br. s, *C*H$_2$CH$_2$*C*H$_2$), 37.6 (br. s, *C*(CH$_3$)$_2$), 33.3 (br. s, *C*H$_3$), 29.4 (br. s, *C*H$_3$) and 19.5 ppm (br. s, CH$_2$*C*H$_2$CH$_2$). ^{31}P{^{1}H} NMR (162 MHz, $CDCl_3$): δ br. s 28.4 ppm. Elem. Anal. Calcd for $C_{26}H_{44}Cl_2P_2Pd$: C, 52.41; H, 7.44. Found: C, 51.32; H, 7.30. Single crystals suitable for X-ray diffraction were grown *via* a vapour diffusion of pentane into a saturated solution of the compound in CH_2Cl_2.

[(BTMPX)Pd(O$_2$CCF$_3$)$_2$], 23.

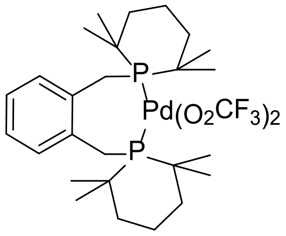

[(BTMPX)PdCl$_2$] (0.10 g, 0.17 mmol) was dissolved in CH_2Cl_2 (5 mL) to giver a clear deep orange solution. To this was added [Ag(O$_2$CCF$_3$)$_2$] (0.089 g, 0.41 mmol) which gave a yellow solution. After 16 hours the mixture was passed through a glass filter and reduced in volume to ~1 mL. Pentane (3 mL) was added which precipitated a yellow powder which was dried *in vacuo*. Yield = 80 mg (65%). ^{1}H NMR (400 MHz, $CDCl_3$): δ 7.50 – 7.40 (2H, m, Ar*H*), 7.40 – 7.20 (2H, m, Ar*H*), 3.24 (4H, d, $^2J_{HP}$ = 12.1 Hz, C*H$_2$*P), 2.35 (4H, m, C*H$_2$*) and 1.94 – 1.06 ppm (32H, m). ^{13}C{^{1}H} NMR (101 MHz, $CDCl_3$): δ 162.2 (q, $^2J_{CF}$ = 36.36 Hz, *C*O$_2$CF$_3$), 132.8 – 132.4 (m, Ar*C*), 128.2 (s, ArC*H*), 115.9 (q, $^1J_{CP}$ = 291.3 Hz, *C*F$_3$), 38.7 (s, C$_q$*C*H$_2$), 37.0 (d, $^1J_{CP}$ = 17.2 Hz, *C*(CH$_3$)$_2$), 32.3 (br. s, *C*H$_3$), 27.9 (s, *C*H$_3$), 27.5 (d, $^1J_{CP}$ = 18.0 Hz, *C*H$_2$P) and 19.0 ppm (br. s, CH$_2$*C*H$_2$CH$_2$). ^{31}P{^{1}H} NMR (162 MHz, $CDCl_3$): δ 32.3 ppm. ^{19}F{^{1}H} NMR (376 MHz, $CDCl_3$): δ −75.5 ppm (s, C*F$_3$*). Single crystals of compound **22** suitable for X-ray diffraction were grown *via* a vapour diffusion of pentane into a saturated solution of the compound in CH_2Cl_2.

[^{TMPhos}(PNP)Ru(CO)(Cl)H], 24.

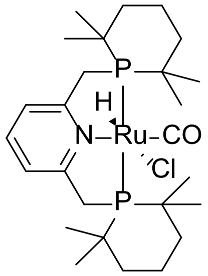

Carbonylchlorohydridotris(triphenylphosphine)ruthenium(II) (221 mg, 0.23 mmol) and ^{TMPhos}(PNP) ligand (120 mg, 0.29 mmol) were stirred at 65 °C for 16 hours. The mixture was then cooled to RT and reduced in volume to approximately 1 mL. Et_2O (5 mL) was added to precipitate a pale yellow solid which was washed with more Et_2O (2 × 5 mL) and dried *in vacuo*. Yield = 0.11 g (81%). ^{1}H NMR (400 MHz, $CDCl_3$): δ 7.45 (1H, t, $^3J_{HH}$ = 7.7 Hz, Ar*H*), 7.14 (2H, d, $^3J_{HH}$ = 7.7 Hz, Ar*H*), 3.98 (2H, dt, $^2J_{HH}$ = 16.5 Hz, $|^2J_{HP} + ^4J_{HP}|$ = 3.4 Hz, C*H$_2$*P), 3.47 (2H, dt, $^2J_{HH}$ = 16.5 Hz, $|^2J_{HP} + ^4J_{HP}|$ = 3.6 Hz, C*H$_2$*P), 1.88 – 1.53 (12H, m, C*H$_2$*), 1.59 (6H, t, $|^3J_{HP} + ^5J_{HP}|$ = 8.2 Hz, C*H$_3$*), 1.49 (6H, $|^3J_{HP} + ^5J_{HP}|$ = 6.7 Hz, C*H$_3$*), 1.40 (6H, $|^3J_{HP} + ^5J_{HP}|$ = 7.8 Hz, C*H$_3$*), 0.85 (6H, $|^3J_{HP} + ^5J_{HP}|$ = 6.6 Hz, C*H$_3$*) and −15.05 ppm (1H, t, $^2J_{HP}$ = 19.2 Hz, Ru-*H*). ^{13}C{^{1}H} NMR (101 MHz, $CDCl_3$): δ 209.0 (t, $^2J_{CP}$ = 11.7 Hz, *C*=O), 163.6 (t, $^2J_{CP}$ = 5.2 Hz, Ar*C$_q$*), 137.0 (s, Ar*C*), 119.8 (t, $^3J_{CP}$ = 4.5 Hz, Ar*C*), 40.7 (s, C$_q$*CH$_2$*), 38.3 (s, C$_q$*CH$_2$*), 37.0 (t, $^1J_{CP}$ = 6.6 Hz, C*H$_2$*P), 36.1 (t, $^1J_{CP}$ = 7.3 Hz, *C*(CH$_3$)$_2$), 33.6 (t, $^1J_{CP}$ = 11.0 Hz, *C*(CH$_3$)$_2$), 30.7 (s, *C*H$_3$), 29.9 (s, *C*H$_3$), 27.4 (t, $^2J_{CP}$ = 7.2 Hz, *C*H$_3$), 27.0 (t, $^2J_{CP}$ = 6.4 Hz, *C*H$_3$) and 19.6 ppm (s,

$CH_2CH_2CH_2$). $^{31}P\{^1H\}$ NMR (162 MHz, $CDCl_3$): δ 70.6 ppm. HR-MS (+ve ESI): m/z (*calc.*) $[M-Cl]^+$ 550.1942; found 550.1944. Elem. Anal. Calcd for $C_{26}H_{44}ClNOP_2Ru$: C, 53.37; H, 7.58; N, 2.39. Found: C, 51.85; H, 7.085; N, 1.93. Single crystals of compound **23** suitable for X-ray diffraction were grown *via* a vapour diffusion of pentane into a saturated solution of the compound in CH_2Cl_2.

[^{TMPhos}(PCP)Ir(H)Cl], 25.

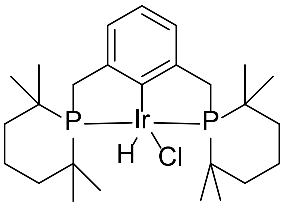

^{TMPhos}(PCP) (130 mg, 0.31 mmol) and $[(COD)IrCl]_2$ (100.0 mg, 0.148 mmol) were dissolved in toluene (5 mL) and the flask was flushed with H_2 gas. The mixture was heated to 110 °C for 96 hours giving a deep red solution. The volatiles were removed *in vacuo* and the red solid obtained was washed with pentane (3 × 4 mL) and dried. Yield = 86 mg (43%). ^1H NMR (400 MHz, $CDCl_3$): δ 6.95 (2H, d, $^3J_{HH}$ = 6.9 Hz, ArC*H*), 6.74 (1H, t, $^3J_{HH}$ = 7.6 Hz, ArC*H*), 3.33 (2H, dt, $^2J_{HH}$ = 17.2 Hz, $^2J_{HP}$ = 3.8 Hz, PCH_2), 3.15 (2H, dt, $^2J_{HH}$ = 17.2 Hz, $^2J_{HP}$ = 4.3 Hz, PCH_2), 2.35 – 2.02 (4H, m, $CH_2CH_2CH_2$), *1*.89 – 1.43 (8H, m, $CH_2CH_2CH_2$), 1.35 (6H, t, $|^3J_{HP} + {}^5J_{HP}|$ = 8.2 Hz, $C(CH_3)_2$), 1.22 (12H, t, $|^3J_{HP} + {}^5J_{HP}|$ = 5.9 Hz, $C(CH_3)_2$), 1.12 (6H, t, $|^3J_{HP} + {}^5J_{HP}|$ = 6.0 Hz, $C(CH_3)_2$) and −40.44 ppm (1H, s, Ir-*H*). $^{13}C\{^1H\}$ NMR (101 MHz, $CDCl_3$): δ 150.4 (t, $|^2J_{CP} + {}^4J_{CP}|$, Ar$C_q$$CH_2$), 144.0 (br. s, Ar*C*-Ir), 122.8 (s, Ar*C*H), 121.1 (t, $|^3J_{CP} + {}^5J_{CP}|$ = 7.8 Hz, Ar*C*H), 39.1 (s, $CH_2CH_2CH_2$) , 38.2 (s, $CH_2CH_2CH_2$), 35.9 (t, $|^1J_{CP} + {}^3J_{CP}|$ = 10.5 Hz, $C(CH_3)_2$), 35.0 (t, $|^1J_{CP} + {}^3J_{CP}|$ = 13.8 Hz, CH_2P), 32.5 (t, $|^1J_{CP} + {}^3J_{CP}|$ = 12.1 Hz, $C(CH_3)_2$), 29.0 (d, $^2J_{CP}$ = 31.4 Hz, $C(CH_3)_2$), 28.6 (s, $C(CH_3)_2$) , 27.6 (br. s, $C(CH_3)_2$) and 19.9 (s, $CH_2CH_2CH_2$) ppm. $^{31}P\{^1H\}$ NMR (162 MHz, $CDCl_3$): δ 32.3 ppm. HR-MS (+ve ESI): m/z (calc.) $[M-Cl]^+$ 611.2548; found 611.2537. Elem. Anal. Calcd for $C_{26}H_{44}ClIrP_2$: C, 48.32; H, 6.86. Found: C, 48.02; H, 6.37.

***tert*-Butylphosphinic acid, S4.**

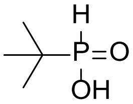

Tert-Butyldichlorophosphine (0.5 g, 3.15 mmol) was stirred in H_2O (10 mL) for 16 hours. After this time the volatiles were removed *in vacuo*. Toluene was added (5 mL) and the volatiles again removed *in vacuo* to give a white solid. Yield = xx g (%). ^1H NMR (400 MHz, $CDCl_3$): δ 13.0 (1H, s, O*H*), 6.71 (1H, d, $^1J_{PH}$ = 529.9 Hz, P*H*), and 1.12 ppm (9H, d, $^3J_{HP}$ = 17.9 Hz, CH_3). $^{13}C\{^1H\}$ NMR (101 MHz, $CDCl_3$): δ 30.9 (d, $^1J_{CP}$ = 96.1 Hz, $C(CH_3)_3$) and 22.5 ppm (d, $^2J_{CP}$ = 1.9 Hz, $C(CH_3)_3$). $^{31}P\{^1H\}$ NMR (162 MHz, $CDCl_3$): δ 49.3 ppm.

2. Air oxidation of TMPhos vs $^{t}Bu_2PH$ (NMR Spectra)

The relevant phosphine (0.13 mmol) was dissolved in 0.8 mL of $CDCl_3$ and placed in a normal NMR tube. ^{31}P NMR spectra were recorded periodically and the results are shown below.

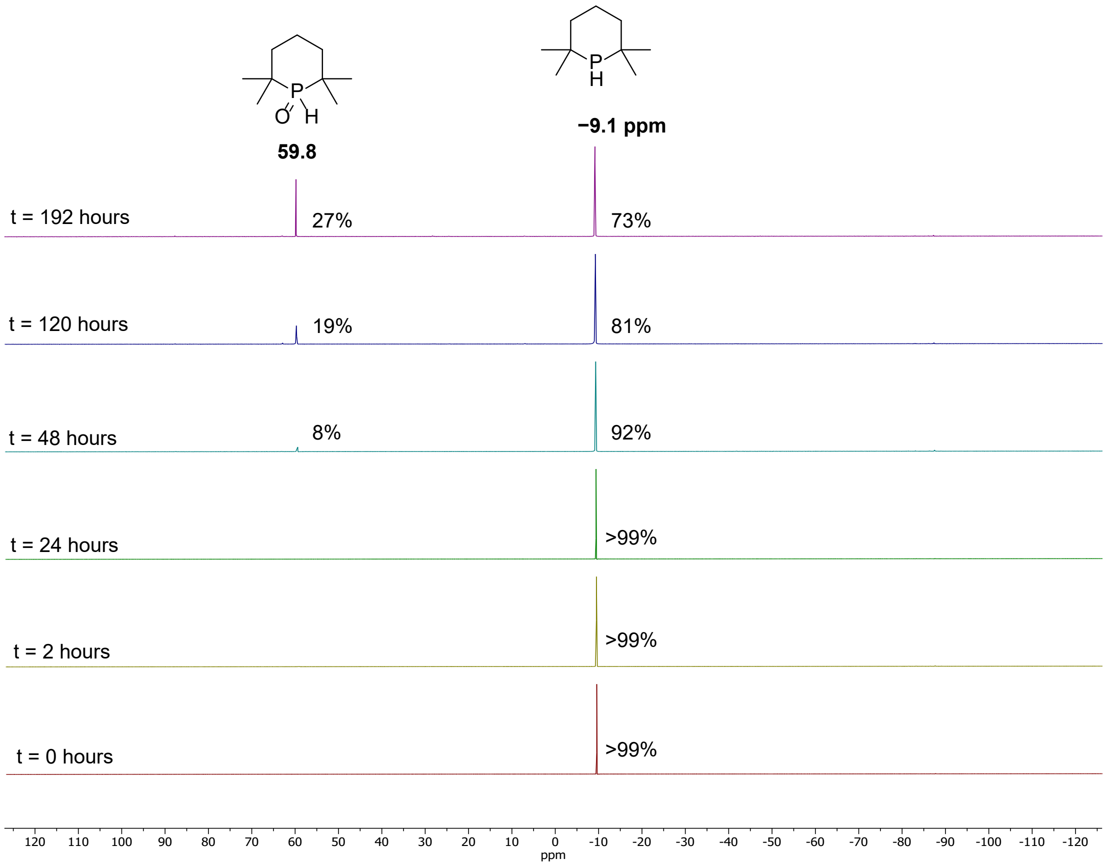

Supplementary Figure 1. $^{31}P\{^1H\}$ NMR (162 MHz, $CDCl_3$) spectra of a $CDCl_3$ solution of **TMPhos** exposed to air over a period of 192 hours.

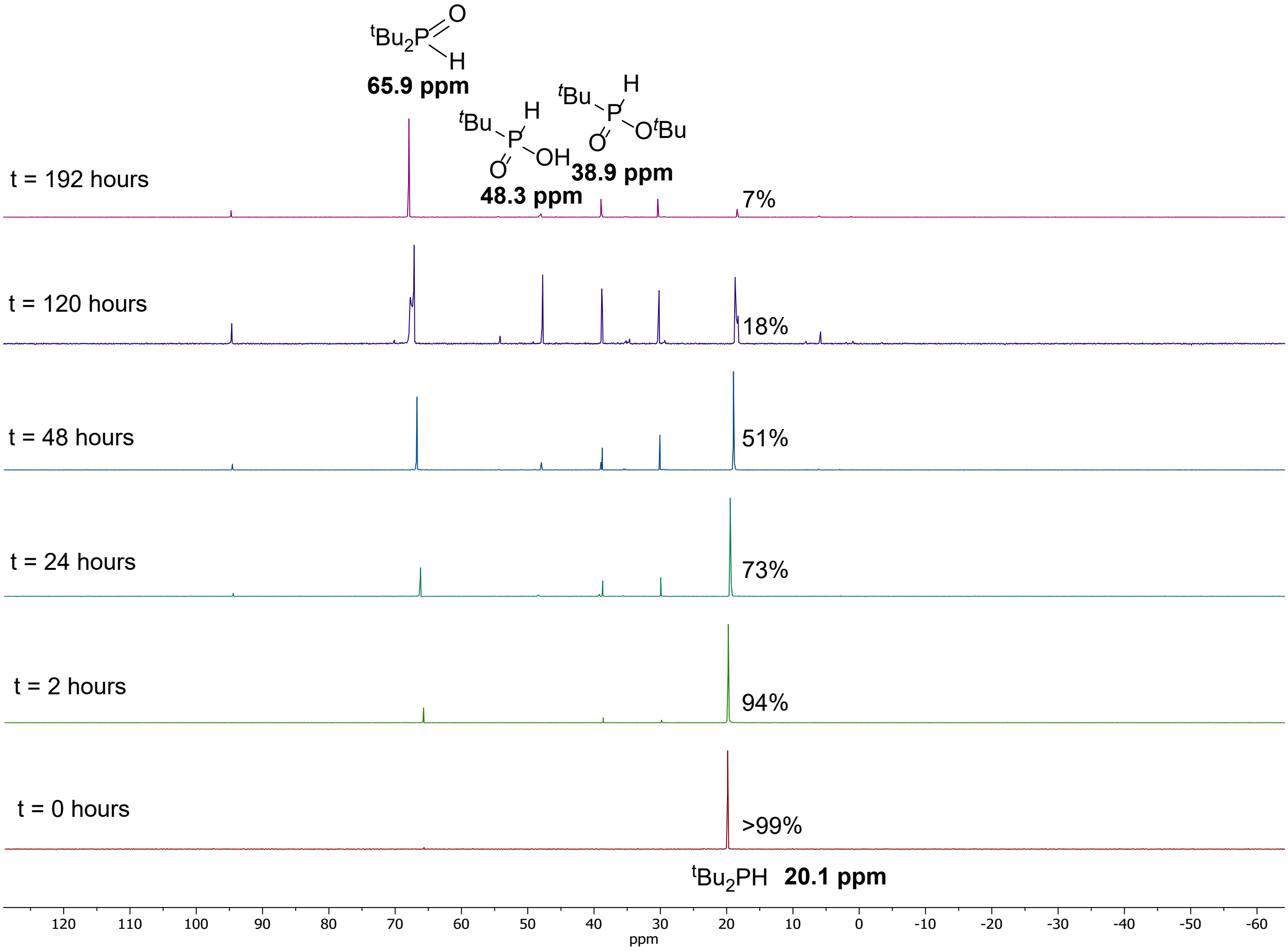

Supplementary Figure 2. $^{31}P\{^{1}H\}$ NMR (162 MHz, $CDCl_3$) spectra of a $CDCl_3$ solution of $^{t}Bu_2PH$ exposed to air over a period of 192 hours.

3. Single Crystal X-ray Diffraction.

Data for compound **15** were collected on a Bruker-D8 Venture diffractometer using graphite monochromated Mo-Kα radiation (λ = 0.71073 Å). The APEX 4 software was used for the unit cell determination and data collection.[5] The data reduction and global cell refinement were done using the Bruker SAINT (V8.32B) software package[6] and a numerical absorption correction was performed with SADABS.[7] For the remaining compounds, diffraction data were collected on an Agilent Technologies Dual Source Supernova, four-circle diffractometer fitted with CCD detector, with graphite monochromated Mo−Kα radiation (λ = 0.71073 Å) for compounds **5** and **23**, and Cu−Kα radiation (λ = 1.54184 Å) for the remaining compounds. For all the structures, CrysAlisPro software[8] was used for data collection and reduction and absorption correction using face indexing and Gaussian corrections. Structure solution and refinement were carried out using Intrinsic Phasing in SHELXT-2015[9] and refinement by full matrix least-squares on F2 was performed using SHELXL-2015,[10] both implemented in the Olex2 software.[11] Non-hydrogen atoms in the structures were refined with anisotropic displacement parameters. Hydrogen atoms on the Ru atoms in compound **24** were located from difference Fourier map and refined freely, maintaining isotropic displacement parameters (Uiso), while the remaining hydrogen atoms were fixed in idealized positions with their displacement parameters riding on the values of their parent atoms. All the crystal data were deposited in the Cambridge Crystallographic Data Centre (CCDC) with deposition numbers 2182393-2182399. These data can be obtained free of charge from the CCDC via http://www.ccdc.cam.ac.uk/conts/retrieving.html and deposited as Supporting Notes.

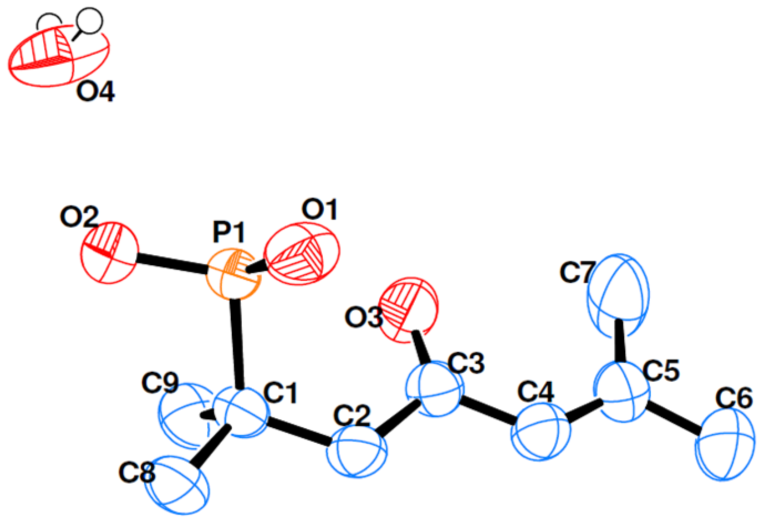

Supplementary Figure 3. Molecular structure of (2,6-dimethyl-4-oxohept-5-en-2-yl)phosphinic acid, **2·H₂O**. (thermal ellipsoids are drawn at 50% probability).

Supplementary Table 1. Crystal data and structure refinement for **2·H_2O**.

CDCC code	2182394	Absorption coefficient	1.884 mm^{-1}
Empirical formula	$C_9H_{17}O_3P.H_2O$	F(000)	480.0
Formula weight	222.21	Crystal size	0.544 × 0.44 × 0.23 mm
Temperature	293 K	Theta range for data collection	8.71 to 145.51°
Wavelength	1.54184 Å (CuKα)	Limiting indices	−12<=h<=12, −7<=k<=7, −25<=l<=25
Crystal system, space group	Monoclinic, P 2_1/c	Reflections collected / unique	14627 / 2486 [R(int) = 0.0352]
Unit cell dimensions	a = 9.9213(2) Å α = 90°	Data / restraints / parameters	2486 / 0 / 147
	b = 6.23820(10) Å β = 95.314(2)°	Goodness-of-fit on F^2	1.051
	c = 20.3925(3) Å γ = 90°	Final R indices [I>2σ(I)]	R_1 = 0.0514, wR_2 = 0.1457
Volume	1256.69(4) $Å^3$	R indices (all data)	R_1 = 0.0530, wR_2 = 0.1485
Z, Calculated density	4, 1.174 g/cm^3	Largest diff. peak and hole	0.23 and −0.35 e.$Å^{-3}$

Supplementary Table 2. Bond lengths [Å] and angles [°] for **2.H₂O**.

P(1)-O(2)	1.5489(15)	O(1)-P(1)-C(1)	112.58(9)
P(1)-C(1)	1.4780(14)	C(2)-C(1)-P(1)	110.82(12)
P(1)-C(1)	1.8147(17)	C(2)-C(1)-C(8)	107.51(15)
O(3)-C(3)	1.221(3)	C(2)-C(1)-C(9)	112.01(16)
C(1)-C(2)	1.530(3)	C(8)-C(1)-P(1)	107.25(12)
C(1)-C(8)	1.543(3)	C(9)-C(1)-P(1)	109.20(14)
C(1)-C(9)	1.534(3)	C(9)-C(1)-C(8)	109.92(17)
C(3)-C(2)	1.513(2)	O(3)-C(3)-C(2)	120.56(17)
C(3)-C(4)	1.460(3)	O(3)-C(3)-C(4)	124.51(17)
C(5)-C(4)	1.340(3)	C(4)-C(3)-C(2)	114.93(17)
C(5)-C(6)	1.493(3)	C(3)-C(2)-C(1)	118.46(15)
C(5)-C(7)	1.471(4)	C(4)-C(5)-C(6)	120.1(2)
		C(4)-C(5)-C(7)	124.7(2)
O(2)-P(1)-C(1)	104.14(8)	C(7)-C(5)-C(6)	115.2(2)
O(1)-P(1)-O(2)	115.84(9)	C(5)-C(4)-C(3)	127.3(2)

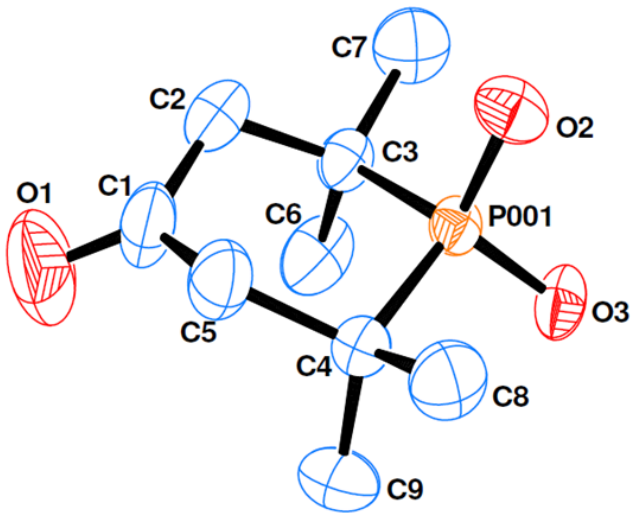

Supplementary Figure 4. Molecular structure of 2,2,6,6-tetramethylphosphorinic acid, **5**. (Thermal ellipsoids are drawn at 50% probability).

Supplementary Table 3. Crystal data and structure refinement for compound **5**.

CCDC code	2182395	Absorption coefficient	0.229 mm^{-1}
Empirical formula	$C_9H_{17}O_3P$	F(000)	880.0
Formula weight	204.19	Crystal size	0.257 × 0.162 × 0.031 mm
Temperature	293(2) K	Theta range for data collection	5.458 to 59.798°
Wavelength	0.71073 Å (MoKα)	Limiting indices	−15<=h<=15, -9<=k<=8, −20<=l<=20
Crystal system, space group	Monoclinic, C2/c	Reflections collected / unique	14554 / 2925 [R(int) = 0.0534]
Unit cell dimensions	a = 22.5884(15) Å α = 90°	Data / restraints / parameters	2925 / 0 / 126
	b = 6.4343(4) Å β = 105.593(7)°	Goodness-of-fit on F^2	1.112
	c = 15.4993(13) Å γ = 90°	Final R indices [I>2σ(I)]	R_1 = 0.0606, wR_2 = 0.1492
Volume	2169.8(3) Å^3	R indices (all data)	R_1 = 0.0927, wR_2 = 0.1635
Z, Calculated density	8, 1.250 g/cm^3		
Largest diff. peak and hole	0.32 and −0.21 e.A^{-3}		

Supplementary Table 4. Bond lengths [Å] and angles [°] for compound **5**.

P(001)-O(3)	1.5522(19)	C(3)-P(001)-C(4)	108.84(13)
P(001)-O(2)	1.4936(18)	C(2)-C(3)-P(001)	107.45(19)
P(001)-C(3)	1.821(3)	C(6)-C(3)-P(001)	113.4(2)
P(001)-C(4)	1.828(3)	C(6)-C(3)-C(2)	110.4(2)
C(3)-C(2)	1.546(4)	C(6)-C(3)-C(7)	108.2(3)
C(3)-C(6)	1.528(4)	C(7)-C(3)-P(001)	107.66(19)
C(3)-C(7)	1.533(4)	C(7)-C(3)-C(2)	109.7(3)
C(4)-C(5)	1.549(4)	C(5)-C(4)-P(001)	108.3(2)
C(4)-C(8)	1.535(4)	C(8)-C(4)-P(001)	108.2(2)
C(4)-C(9)	1.528(4)	C(8)-C(4)-C(5)	109.7(2)
C(2)-C(1)	1.508(5)	C(9)-C(4)-P(001)	112.1(2)
C(5)-C(1)	1.516(5)	C(9)-C(4)-C(5)	110.7(3)
O(1)-C(1)	1.206(4)	C(9)-C(4)-C(8)	107.8(3)
		C(1)-C(2)-H(3)	113.3(3)
O(3)-P(001)-C(3)	105.56(12)	C(1)-C(5)-C(4)	113.9(3)
O(3)-P(001)-C(4)	108.73(12)	C(2)-C(1)-C(5)	116.3(3)
O(2)-P(001)-O(3)	114.24(11)	O(1)-C(1)-C(2)	121.8(3)
O(2)-P(001)-C(3)	108.62(12)	O(1)-C(1)-C(5)	121.9(3)
O(2)-P(001)-C(4)	110.62(12)		

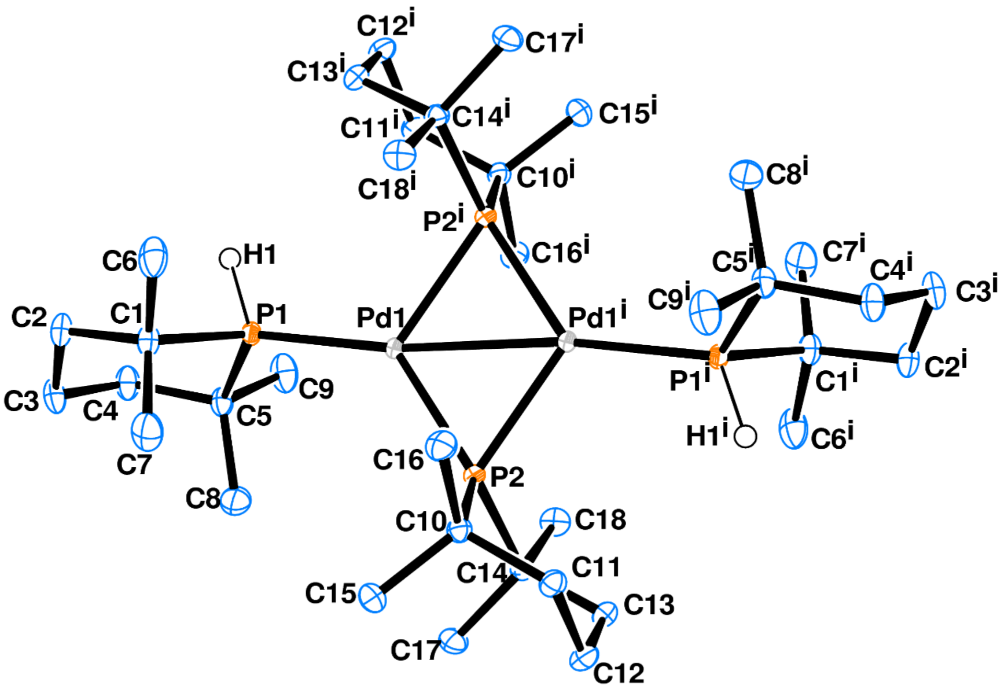

Supplementary Figure 5. Molecular structure of bimetallic complex **15**,(thermal ellipsoids are drawn at 50% probability).

Supplementary Table 5. Crystal data and structure refinement for of bimetallic complex **15**.

CCDC code	2182396	Absorption coefficient	1.086 mm^{-1}
Empirical formula	$C_{36}H_{74}P_4Pd_2$	F(000)	442
Formula weight	843.63	Crystal size	0.293 x 0.147 x 0.125 mm
Temperature	100(2) K	Theta range for data collection	3.512 to 30.516°
Wavelength	0.71073 Å (CuKα)	Limiting indices	−13<=h<=13, -15<=k<=15, −15<=l<=15
Crystal system, space group	triclinic, P-1	Reflections collected / unique	59130 / 5962 [R(int) = 0.0389]
Unit cell dimensions	a = 9.3402(4) Å α = 27.242(2)°	Data / restraints / parameters	5962 / 0 / 203
	b = 10.8747(5) Å β = 70.2930(10)°	Goodness-of-fit on F^2	1.086
	c = 11.0311(5) Å γ = 77.625(2)°	Final R indices [I>2σ(I)]	R_1 = 0.0158, wR_2 = 0.0397
Volume	996.77(8) Å^3	R indices (all data)	R_1 = 0.0161, wR_2 = 0.0399
Z, Calculated density	1, 1.405 g/cm^3	Largest diff. peak and hole	0.447 and −0.442 e.A^{-3}

Supplementary Table 6. Bond lengths [Å] and angles [°] for bimetallic complex **15**.

Pd(1)-P(1)	2.2686(3)		
Pd(1)-P(2)	2.3124(3)	P(1)-Pd(1)-P(2)	131.267(9)
Pd(1)-P(2)#1	2.3426(3)	P(1)-Pd(1)-P(2)#1	116.715(9)
Pd(1)-Pd(1)#1	2.60259(16)	P(2)-Pd(1)-P(2)#1	112.017(7)
P(1)-C(5)	1.8677(10)	P(1)-Pd(1)-Pd(1)#1	172.166(8)
P(1)-C(1)	1.8696(10)	P(2)-Pd(1)-Pd(1)#1	56.562(7)
P(1)-H(1)	1.280(16)	P(2)#1-Pd(1)-Pd(1)#1	55.455(7)
P(2)-C(10)	1.8698(10)	C(5)-P(1)-C(1)	105.34(5)
P(2)-C(14)	1.8712(10)	C(5)-P(1)-Pd(1)	117.48(3)
C(1)-C(7)	1.5314(16)	C(1)-P(1)-Pd(1)	120.48(3)
C(1)-C(6)	1.5351(16)	C(5)-P(1)-H(1)	95.8(7)
C(1)-C(2)	1.5396(14)	C(1)-P(1)-H(1)	95.7(7)
C(2)-C(3)	1.5235(16)	Pd(1)-P(1)-H(1)	117.5(7)
C(2)-H(2A)	0.9900	C(10)-P(2)-C(14)	104.64(4)
C(2)-H(2B)	0.9900	C(10)-P(2)-Pd(1)	123.98(3)
C(3)-C(4)	1.5244(15)	C(14)-P(2)-Pd(1)	127.01(3)
C(3)-H(3A)	0.9900	C(10)-P(2)-Pd(1)#1	113.75(3)
C(3)-H(3B)	0.9900	C(14)-P(2)-Pd(1)#1	112.77(3)
C(4)-C(5)	1.5369(14)	Pd(1)-P(2)-Pd(1)#1	67.982(7)
C(4)-H(4A)	0.9900	C(7)-C(1)-C(6)	107.95(10)
C(4)-H(4B)	0.9900	C(7)-C(1)-C(2)	111.48(9)
C(5)-C(8)	1.5310(16)	C(6)-C(1)-C(2)	108.52(9)
C(5)-C(9)	1.5334(15)	C(7)-C(1)-P(1)	111.31(7)
C(6)-H(6A)	0.9800	C(6)-C(1)-P(1)	107.33(7)
C(6)-H(6B)	0.9800	C(2)-C(1)-P(1)	110.10(7)
C(6)-H(6C)	0.9800	C(3)-C(2)-C(1)	115.32(9)
C(7)-H(7A)	0.9800	C(3)-C(2)-H(2A)	108.4
C(7)-H(7B)	0.9800	C(1)-C(2)-H(2A)	108.4
C(7)-H(7C)	0.9800	C(3)-C(2)-H(2B)	108.4
C(8)-H(8A)	0.9800	C(1)-C(2)-H(2B)	108.4
C(8)-H(8B)	0.9800	H(2A)-C(2)-H(2B)	107.5
C(8)-H(8C)	0.9800	C(2)-C(3)-C(4)	113.24(9)
C(9)-H(9A)	0.9800	C(2)-C(3)-H(3A)	108.9
C(9)-H(9B)	0.9800	C(4)-C(3)-H(3A)	108.9
C(9)-H(9C)	0.9800	C(2)-C(3)-H(3B)	108.9
C(10)-C(16)	1.5342(14)	C(4)-C(3)-H(3B)	108.9
C(10)-C(15)	1.5372(14)	H(3A)-C(3)-H(3B)	107.7
C(10)-C(11)	1.5380(14)	C(3)-C(4)-C(5)	115.01(9)
C(11)-C(12)	1.5292(14)	C(3)-C(4)-H(4A)	108.5
C(11)-H(11A)	0.9900	C(5)-C(4)-H(4A)	108.5
C(11)-H(11B)	0.9900	C(3)-C(4)-H(4B)	108.5
C(12)-C(13)	1.5326(14)	C(5)-C(4)-H(4B)	108.5
C(12)-H(12A)	0.9900	H(4A)-C(4)-H(4B)	107.5
C(12)-H(12B)	0.9900	C(8)-C(5)-C(9)	108.40(9)
C(13)-C(14)	1.5414(14)	C(8)-C(5)-C(4)	111.06(9)
C(13)-H(13A)	0.9900	C(9)-C(5)-C(4)	109.00(9)
C(13)-H(13B)	0.9900	C(8)-C(5)-P(1)	111.52(7)
C(14)-C(18)	1.5337(14)	C(9)-C(5)-P(1)	106.25(7)
C(14)-C(17)	1.5350(14)	C(4)-C(5)-P(1)	110.45(7)
C(15)-H(15A)	0.9800	C(1)-C(6)-H(6A)	109.5
C(15)-H(15B)	0.9800	C(1)-C(6)-H(6B)	109.5
C(15)-H(15C)	0.9800	H(6A)-C(6)-H(6B)	109.5
C(16)-H(16A)	0.9800	C(1)-C(6)-H(6C)	109.5
C(16)-H(16B)	0.9800	H(6A)-C(6)-H(6C)	109.5
C(16)-H(16C)	0.9800	H(6B)-C(6)-H(6C)	109.5
C(17)-H(17A)	0.9800	C(1)-C(7)-H(7A)	109.5
C(17)-H(17B)	0.9800	C(1)-C(7)-H(7B)	109.5
C(17)-H(17C)	0.9800	H(7A)-C(7)-H(7B)	109.5
C(18)-H(18A)	0.9800	C(1)-C(7)-H(7C)	109.5
C(18)-H(18B)	0.9800	H(7A)-C(7)-H(7C)	109.5
C(18)-H(18C)	0.9800	H(7B)-C(7)-H(7C)	109.5

C(5)-C(8)-H(8A)	109.5	C(12)-C(13)-H(13B)	108.5
C(5)-C(8)-H(8B)	109.5	C(14)-C(13)-H(13B)	108.5
H(8A)-C(8)-H(8B)	109.5	H(13A)-C(13)-H(13B)	107.5
C(5)-C(8)-H(8C)	109.5	C(18)-C(14)-C(17)	107.71(8)
H(8A)-C(8)-H(8C)	109.5	C(18)-C(14)-C(13)	109.83(8)
H(8B)-C(8)-H(8C)	109.5	C(17)-C(14)-C(13)	110.47(8)
C(5)-C(9)-H(9A)	109.5	C(18)-C(14)-P(2)	105.76(7)
C(5)-C(9)-H(9B)	109.5	C(17)-C(14)-P(2)	114.33(7)
H(9A)-C(9)-H(9B)	109.5	C(13)-C(14)-P(2)	108.59(6)
C(5)-C(9)-H(9C)	109.5	C(10)-C(15)-H(15A)	109.5
H(9A)-C(9)-H(9C)	109.5	C(10)-C(15)-H(15B)	109.5
H(9B)-C(9)-H(9C)	109.5	H(15A)-C(15)-H(15B)	109.5
C(16)-C(10)-C(15)	107.86(8)	C(10)-C(15)-H(15C)	109.5
C(16)-C(10)-C(11)	109.66(8)	H(15A)-C(15)-H(15C)	109.5
C(15)-C(10)-C(11)	110.92(8)	H(15B)-C(15)-H(15C)	109.5
C(16)-C(10)-P(2)	106.50(7)	C(10)-C(16)-H(16A)	109.5
C(15)-C(10)-P(2)	112.59(7)	C(10)-C(16)-H(16B)	109.5
C(11)-C(10)-P(2)	109.18(6)	H(16A)-C(16)-H(16B)	109.5
C(12)-C(11)-C(10)	115.21(8)	C(10)-C(16)-H(16C)	109.5
C(12)-C(11)-H(11A)	108.5	H(16A)-C(16)-H(16C)	109.5
C(10)-C(11)-H(11A)	108.5	H(16B)-C(16)-H(16C)	109.5
C(12)-C(11)-H(11B)	108.5	C(14)-C(17)-H(17A)	109.5
C(10)-C(11)-H(11B)	108.5	C(14)-C(17)-H(17B)	109.5
H(11A)-C(11)-H(11B)	107.5	H(17A)-C(17)-H(17B)	109.5
C(11)-C(12)-C(13)	113.65(8)	C(14)-C(17)-H(17C)	109.5
C(11)-C(12)-H(12A)	108.8	H(17A)-C(17)-H(17C)	109.5
C(13)-C(12)-H(12A)	108.8	H(17B)-C(17)-H(17C)	109.5
C(11)-C(12)-H(12B)	108.8	C(14)-C(18)-H(18A)	109.5
C(13)-C(12)-H(12B)	108.8	C(14)-C(18)-H(18B)	109.5
H(12A)-C(12)-H(12B)	107.7	H(18A)-C(18)-H(18B)	109.5
C(12)-C(13)-C(14)	115.07(8)	C(14)-C(18)-H(18C)	109.5
C(12)-C(13)-H(13A)	108.5	H(18A)-C(18)-H(18C)	109.5
C(14)-C(13)-H(13A)	108.5	H(18B)-C(18)-H(18C)	109.5

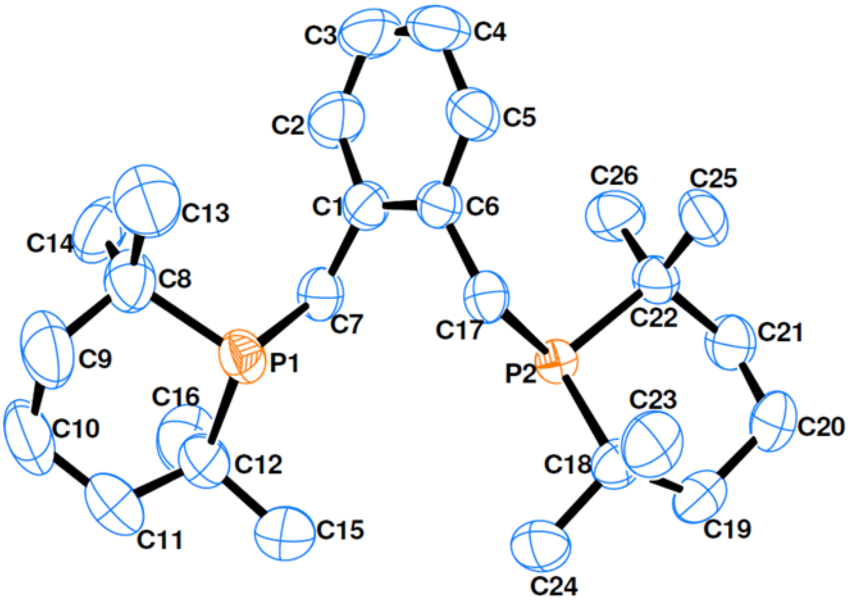

Supplementary Figure 6. Molecular structure of **BTMPX**, compound **20**, (thermal ellipsoids are drawn at 50% probability).

Supplementary Table 7. Crystal data and structure refinement for **BTMPX,** compound **20**.

CCDC code	2182397	Absorption coefficient	1.555 mm^{-1}
Empirical formula	$C_{26}H_{44}P_2$	F(000)	920.0
Formula weight	418.55	Crystal size	0.569 x 0.258 x 0.091 mm
Temperature	293(2) K	Theta range for data collection	6.338 to 145.222°
Wavelength	1.54184 Å (CuKα)	Limiting indices	−18<=h<=19, -9<=k<=7, −25<=l<=24
Crystal system, space group	monoclinic, $P2_1/n$	Reflections collected / unique	10205 / 5059 [R(int) = 0.0332]
Unit cell dimensions	a = 15.8774 (2) Å α = 90°	Data / restraints / parameters	5059 / 0 / 261
	b = 8.12930 (10) Å β = 103.618(2)°	Goodness-of-fit on F^2	1.044
	c = 20.7610 (3) Å γ = 90°	Final R indices [I>2σ(I)]	R_1 = 0.0637, wR_2 = 0.1693
Volume	2604.33(6) $Å^3$	R indices (all data)	R_1 = 0.0684, wR_2 = 0.1783
Z, Calculated density	4, 1.067 g/cm^3	Largest diff. peak and hole	0.47 and −0.57 $e.A^{-3}$

Supplementary Table 8. Bond lengths [Å] and angles [°] for **BTMPX**, **compound 20**.

P(2)-C(17)	1.8670(17)	C(5)-C(6)-C(1)	118.5(2)
P(2)-C(18)	1.8861(19)	C(6)-C(17)-P(2)	114.56(13)
P(2)-C(22)	1.8789(19)	C(6)-C(1)-C(7)	123.14(17)
P(1)-C(7)	1.856(2)	C(2)-C(1)-C(7)	118.8(2)
P(1)-C(8)	1.877(3)	C(2)-C(1)-C(6)	118.1(2)
P(1)-C(12)	1.886(3)	C(23)-C(18)-P(2)	116.62(16)
C(7)-C(1)	1.500(3)	C(23)-C(18)-C(19)	111.28(18)
C(6)-C(17)	1.510(3)	C(24)-C(18)-P(2)	106.06(14)
C(6)-C(1)	1.403(3)	C(24)-C(18)-C(23)	107.73(19)
C(6)-C(5)	1.395(3)	C(24)-C(18)-C(19)	108.25(19)
C(1)-C(2)	1.395(3)	C(19)-C(18)-P(2)	106.52(14)
C(18)-C(23)	1.534(3)	C(21)-C(22)-P(2)	106.16(14)
C(18)-C(24)	1.532(3)	C(25)-C(22)-P(2)	117.48(15)
C(18)-C(19)	1.542(3)	C(25)-C(22)-C(21)	109.79(18)
C(22)-C(21)	1.550(3)	C(25)-C(22)-C(26)	108.77(19)
C(22)-C(25)	1.528(30	C(26)-C(22)-P(2)	106.03(15)
C(22)-C(26)	1.533(3)	C(26)-C(22)-C(21)	108.23(18)
C(5)-C(4)	1.387(4)	C(4)-C(5)-C(6)	121.5(3)
C(2)-C(3)	1.364(4)	C(3)-C(2)-C(1)	122.9(3)
C(21)-C(20)	1.513(4)	C(20)-C(21)-C(22)	115.6(2)
C(19)-C(20)	1.520(4)	C(20)-C(19)-C(18)	115.74(19)
C(8)-C(13)	1.538(3)	C(21)-C(20)-C(19)	112.50(19)
C(8)-C(14)	1.531(4)	C(13)-C(8)-P(1)	106.88(19)
C(8)-C(9)	1.545(4)	C(13)-C(8)-C(9)	107.8(2)
C(4)-C(3)	1.378(5)	C(14)-C(8)-P(1)	117.39(18)
C(12)-C(11)	1.537(4)	C(14)-C(8)-C(13)	107.4(3)
C(12)-C(16)	1.532(5)	C(14)-C(8)-C(9)	110.9(2)
C(12)-C(15)	1.542(4)	C(9)-C(8)-P(1)	106.0(2)
C(10)-C(11)	1.507(5)	C(3)-C(4)-C(5)	119.7(3)
C(10)-C(9)	1.530(5)	C(2)-C(3)-C(4)	119.0(3)
C(17)-P(2)-C(18)	102.09(9)	C(11)-C(12)-P(1)	106.0(2)
C(17)-P(2)-C(22)	105.41(8)	C(11)-C(12)-C(15)	108.4(3)
C(22)-P(2)-C(18)	104.61(9)	C(16)-C(12)-P(1)	116.7(2)
C(7)-P(1)-C(8)	106.28(11)	C(16)-C(12)-C(11)	111.3(3)
C(7)-P(1)-C(12)	101.28(12)	C(16)-C(12)-C(15)	108.6(3)
C(8)-P(1)-C(12)	103.97(11)	C(15)-C(12)-P(1)	105.36(19)
C(1)-C(7)-P(1)	113.59(14)	C(11)-C(10)-C(9)	112.6(3)
C(1)-C(6)-C(17)	122.91(17)	C(10)-C(11)-C(12)	115.4(3)
C(5)-C(6)-C(17)	118.6(2)	C(10)-C(9)-C(8)	115.8(3)

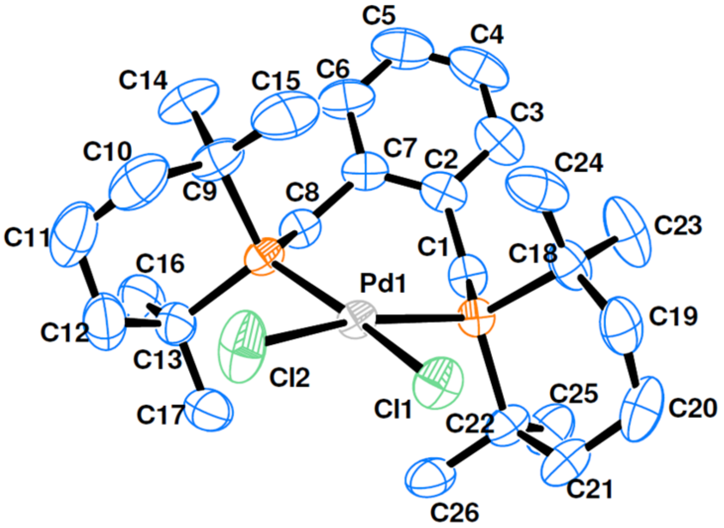

Supplementary Figure 7. Molecular structure of [(**BTMPX**)$PdCl_2$], (thermal ellipsoids are drawn at 50% probability).

Supplementary Table 9. Crystal data and structure refinement for [(**BTMPX**)$PdCl_2$]·CH_2Cl_2.

CCDC code	2182393	Absorption coefficient	8.992 mm^{-1}
Empirical formula	$C_{27}H_{46}Cl_4P_2Pd$	F(000)	1408.0
Formula weight	680.78	Crystal size	0.478 x 0.188 x 0.126 mm
Temperature	293(2) K	Theta range for data collection	7.098 to 145.536°
Wavelength	1.54184 Å (CuKα)	Limiting indices	−11<=h<=8, -30<=k<=30, −17<=l<=17
Crystal system, space group	monoclinic, $P2_1/n$	Reflections collected / unique	21547 / 6157 [R(int) = 0.0407]
Unit cell dimensions	a = 9.17882(12) Å α = 90°	Data / restraints / parameters	8047 / 6 / 450
	b = 24.9064(2) Å β = 108.1740(13)°	Goodness-of-fit on F^2	1.070
	c = 14.41276(17) Å γ = 90°	Final R indices [I>2σ(I)]	R_1 = 0.0504, wR_2 = 0.1414
Volume	3130.55(7) Å^3	R indices (all data)	R_1 = 0.0515, wR_2 = 0.1426
Z, Calculated density	4, 1.444 g/cm^3	Largest diff. peak and hole	1.53 and −0.86 e.A^{-3}

Supplementary Table 10. Bond lengths [Å] and angles [°] for [(**BTMPX**)$PdCl_2$]·CH_2Cl_2.

Pd(1)-P(1)	2.3259(10)	C(6)-C(7)	1.396(6)
Pd(1)-P(2)	2.3268(9)	C(18)-C(19)	1.537(7)
Pd(1)-Cl(1)	2.3549(10)	C(4)-C(3)	1.380(8)
Pd(1)-Cl(2)	2.3501(12)	C(4)-C(5)	1.369(10)
P(1)-C(22)	1.897(4)	C(12)-C(11)	1.530(10)
P(1)-C(1)	1.851(4)	C(9)-C(14)	1.533(7)
C(6)-C(5)	1.374(9)	C(6)-C(15)	1.533(9)
C(1)-C(2)	1.520(6)	C(9)-C(10)	1.547(9)
C(8)-C(7)	1.501(6)	C(3)-C(2)	1.396(7)
C(23)-C(18)	1.528(7)	C(7)-C(2)	1.385(7)
C(21)-C(20)	1.516(9)	C(20)-C(19)	1.514(9)
C(18)-C(24)	1.542(8)	C(11)-C(10)	1.514(11)
P(1)-C(18)	1.893(5)	Cl(3)-C(27)	1.669(10)
P(2)-C(13)	1.904(5)		
P(2)-C(8)	1.848(4)	P(1)-Pd(1)-P(2)	101.76(3)
P(2)-C(9)	1.889(5)	P(1)-Pd(1)-Cl(1)	88.64(4)
Cl(4)-C(27)	1.698(10)	P(1)-Pd(1)-Cl(2)	168.10(4)
C(22)-C(26)	1.540(7)	P(2)-Pd(1)-Cl(1)	169.21(4)
C(22)-C(21)	1.557(6)	P(2)-Pd(1)-Cl(2)	89.08(4)
C(22)-C(25)	1.542(7)	Cl(2)-Pd(1)-Cl(1)	80.83(4)
C(13)-C(16)	1.563(7)	C(22)-P(1)-Pd(1)	109.37(16)
C(13)-C(12)	1.524(8)	C(1)-P(1)-Pd(1)	118.82(14)
C(13)-C(17)	1.531(7)	C(1)-P(1)-C(22)	100.4(2)

C(1)-P(1)-C(18)	105.9(2)	C(13)-C(12)-C(11)	115.7(5)
C(18)-P(1)-Pd(1)	114.21(17)	C(14)-C(9)-P(2)	113.2(4)
C(18)-P(1)-C(22)	106.7(2)	C(14)-C(9)-C(15)	108.5(5)
C(13)-P(2)-Pd(1)	113.34(15)	C(14)-C(9)-C(10)	107.5(5)
C(8)-P(2)-Pd(1)	119.17(14)	C(15)-C(9)-P(2)	107.3(4)
C(8)-P(2)-C(13)	100.2(2)	C(15)-C(9)-C(10)	110.8(5)
C(8)-P(2)-C(9)	106.3(2)	C(10)-C(9)-P(2)	109.6(4)
C(9)-P(2)-Pd(1)	110.63(17)	C(4)-C(3)-C(2)	120.8(6)
C(9)-P(2)-C(13)	106.0(2)	C(6)-C(7)-C(8)	118.8(4)
C(26)-C(22)-P(1)	108.0(3)	C(2)-C(7)-C(6)	118.4(5)
C(26)-C(22)-C(21)	108.8(4)	C(2)-C(7)-C(8)	122.0(4)
C(26)-C(22)-C(25)	107.8(4)	C(3)-C(2)-C(1)	118.9(5)
C(21)-C(22)-P(1)	109.7(3)	C(7)-C(2)-C(1)	120.9(4)
C(25)-C(22)-P(1)	112.7(4)	C(7)-C(2)-C(3)	119.4(4)
C(25)-C(22)-C(21)	109.7(4)	C(19)-C(20)-C(21)	114.0(4)
C(16)-C(13)-P(2)	113.1(4)	C(12)-C(13)-P(2)	111.3(4)
C(2)-C(1)-P(1)	122.3(3)	C(12)-C(13)-C(16)	107.9(5)
C(7)-C(8)-P(2)	122.0(3)	C(12)-C(13)-C(17)	110.5(5)
C(20)-C(21)-C(22)	114.8(5)	C(17)-C(13)-P(2)	107.1(3)
C(23)-C(18)-P(1)	112.8(4)	C(17)-C(13)-C(16)	106.9(5)
C(23)-C(18)-C(24)	108.1(5)	C(5)-C(6)-C(7)	121.8(6)
C(23)-C(18)-C(19)	108.4(5)	C(4)-C(5)-C(6)	119.4(5)
C(24)-C(18)-P(1)	106.6(4)	C(10)-C(11)-C(12)	113.2(5)
C(19)-C(18)-P(1)	111.2(3)	C(20)-C(19)-C(18)	115.3(4)
C(19)-C(18)-C(24)	109.8(4)	C(11)-C(10)-C(9)	116.0(5)
C(5)-C(4)-C(3)	120.0(5)	Cl(3)-C(27)-Cl(4)	115.1(6)

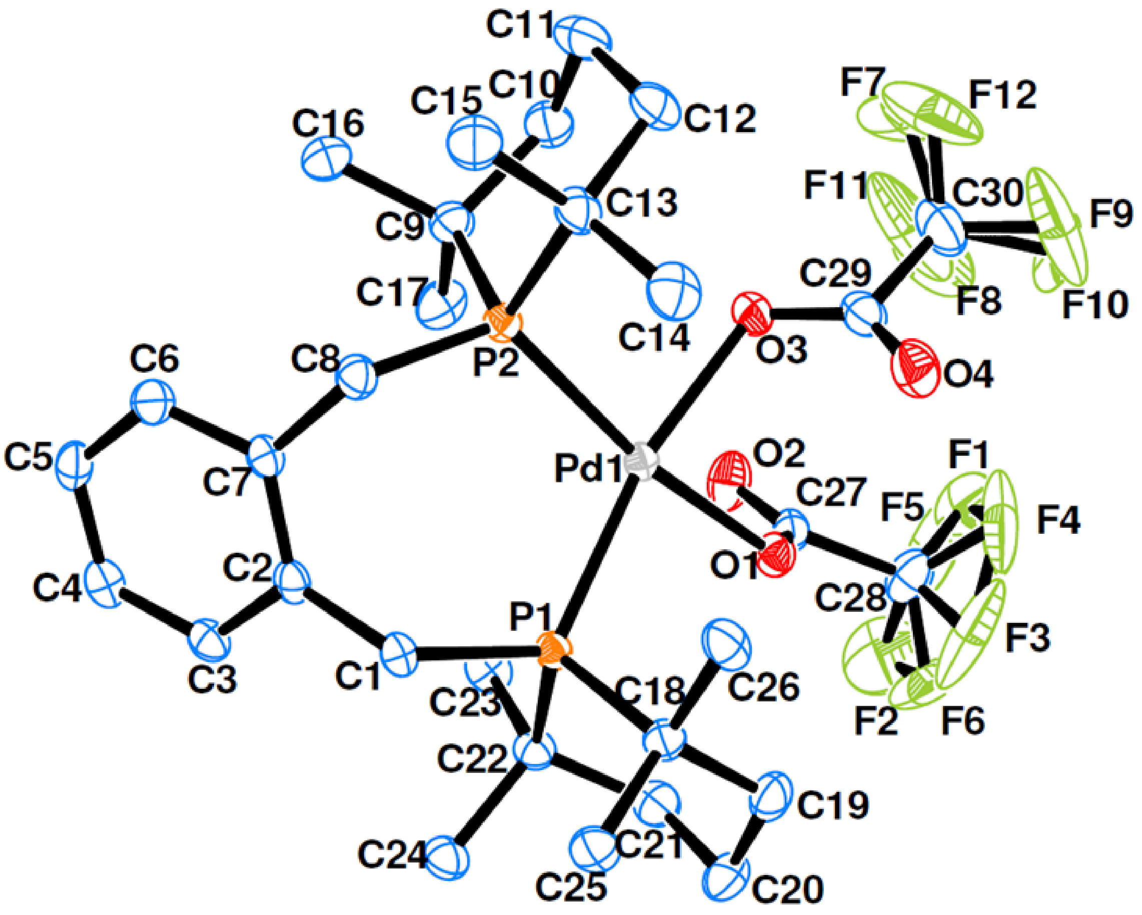

Supplementary Figure 8. Molecular structure of [(**BTMPX**)Pd(O_2CCF_3)$_2$], complex **23**, (thermal ellipsoids are drawn at 50% probability).

Supplementary Table 11. Crystal data and structure refinement for [(**BTMPX**)Pd(O_2CCF_3)$_2$], complex **23**.

CCDC code	2182398	Absorption coefficient	0.735 mm^{-1}
Empirical formula	$C_{30}H_{44}O_4F_6P_2Pd$	F(000)	3088.0
Formula weight	750.99	Crystal size	0.372 x 0.302 x 0.112 mm
Temperature	136.0(4) K	Theta range for data collection	5.48 to 59.658°
Wavelength	0.71073 Å (MoKα)	Limiting indices	−20<=h<=22, -26<=k<=28, −15<=l<=23
Crystal system, space group	orthorhombic, Pbcn	Reflections collected / unique	43377 / 8047 [R(int) = 0.0761]
Unit cell dimensions	a = 16.3896 (2) Å α = 90°	Data / restraints / parameters	8047 / 6 / 450
	b = 22.0768 (5) Å β = 90°	Goodness-of-fit on F^2	1.084
	c = 18.0167 (4) Å γ = 90°	Final R indices [I>2σ(I)]	R_1 = 0.0414, wR_2 = 0.1009
Volume	6519.0(3) Å^3	R indices (all data)	R_1 = 0.0525, wR_2 = 0.1118
Z, Calculated density	8, 1.530 g/cm^3	Largest diff. peak and hole	1.36 and −1.11 e.A^{-3}

Supplementary Table 12. Bond lengths [Å] and angles [°] for [(BTMPX)Pd(O_2CCF_3)$_2$].

Pd(1)P(1)	2.2964(6)	C(28)-F(3)	1.246(6)
Pd(1)-P(2)	2.3147(7)	C(28)-F(5)	1.280(6)
Pd(1)-O(1)	2.0698(17)	C(28)-F(4)	1.383(5)
Pd(1)-O(3)	2.0974(17)	C(28)-F(6)	1.243(6)
P(1)-C(1)	1.835(2)	C(30)-F(8)	1.296(9)
P(1)-C(22)	1.884(2)	C(30)-F(9)	1.298(7)
P(1)-C(18)	1.875(2)	C(30)-F(7)	1.297(9)
P(2)-C(8)	1.848(2)	C(30)-F(12)	1.309(14)
P(2)-C(9)	1.882(3)	C(30)-F(11)	1.278(17)
P(2)-C(13)	1.894(3)	C(30)-F(10)	1.303(11)
O(1)-C(27)	1.277(3)		
O(3)-C(29)	1.273(3)	P(1)-Pd(1)-P(2)	101.90(2)
O(4)-C(29)	1.215(3)	O(1)-Pd(1)-P(1)	87.64(5)
O(2)-C(27)	1.211(3)	O(1)-Pd(1)-P(2)	170.44(5)
C(1)-C(2)	1.508(3)	O(1)-Pd(1)-O(3)	81.12(7)
C(27)-C(28)	1.543(4)	O(3)-Pd(1)-P(1)	168.17(5)
C(22)-C(23)	1.538(3)	O(3)-Pd(1)-P(2)	89.39(5)
C(22)-C(21)	1.548(3)	C(1)-P(1)-Pd(1)	117.65(8)
C(22)-C(24)	1.546(3)	C(1)-P(1)-C(22)	105.74(10)
C(3)-C(2)	1.398(3)	C(1)-P(1)-C(18)	104.31(11)
C(3)-C(4)	1.378(4)	C(22)-P(1)-Pd(1)	115.39(8)
C(29)-C(30)	1.554(4)	C(18)-P(1)-Pd(1)	106.81(8)
C(2)-C(7)	1.399(3)	C(18)-P(1)-C(22)	105.80(11)
C(18)-C(26)	1.530(3)	C(8)-P(2)-Pd(1)	121.53(8)
C(18)-C(25)	1.537(3)	C(8)-P(2)-C(9)	107.69(12)
C(18)-C(19)	1.549(4)	C(8)-P(2)-C(13)	100.98(11)
C(21)-C(20)	1.524(4)	C(9)-P(2)-Pd(1)	105.63(8)
C(4)-C(5)	1.384(3)	C(9)-P(2)-C(13)	106.82(11)
C(16)-C(9)	1.541(4)	C(13)-P(2)-Pd(1)	113.37(9)
C(7)-C(8)	1.513(3)	C(27)-O(1)-Pd(1)	120.00(16)
C(7)-C(6)	1.393(3)	C(29)-O(3)-Pd(1)	116.83(16)
C(14)-C(13)	1.534(4)	C(2)-C(1)-P(1)	119.35(16)
C(19)-C(20)	1.519(4)	O(1)-C(27)-C(28)	110.8(2)
C(5)-C(6)	1.383(3)	O(2)-C(27)-O(1)	130.7(2)
C(9)-C(17)	1.532(4)	O(2)-C(27)-C(28)	118.5(2)
C(9)-C(10)	1.548(4)	C(23)-C(22)- P(1)	107.41(16)
C(15)-C(13)	1.544(4)	C(23)-C(22)-C(21)	109.7(2)
C(13)-C(12)	1.546(4)	C(23)-C(22)-C(24)	109.4(2)
C(10)-C(11)	1.523(4)	C(21)-C(22)-P(1)	110.37(16)
C(12)-C(11)	1.522(4)	C(24)-C(22)-P(1)	113.40(17)
C(28)-F(1)	1.243(6)	C(24)-C(22)-C(21)	106.6(2)
C(28)-F(2)	1.382(6)	C(4)-C(3)-C(2)	122.4(2)

O(3)-C(29)-C(30)	111.7(2)	C(15)-C(13)-P(2)	112.62(18)
O(4)-C(29)-O(3)	130.2(3)	C(15)-C(13)-C(12)	108.9(2)
O(4)-C(29)-C(30)	118.1(2)	C(12)-C(13)-P(2)	110.14(17)
C(3)-C(2)-C(1)	121.1(2)	C(11)-C(10)-C(9)	114.8(2)
C(3)-C(2)-C(7)	117.5(2)	C(11)-C(12)-C(13)	115.6(2)
C(7)-C(2)-C(1)	120.9(2)	F(1)-C(28)-C(27)	113.3(4)
C(26)-C(18)-P(1)	108.76(16)	F(1)-C(28)-F(2)	102.6(5)
C(26)-C(18)-C(25)	108.5(2)	F(1)-C(28)-F(3)	114.0(6)
C(26)-C(18)-C(19)	108.3(2)	F(2)-C(28)-C(27)	105.4(3)
C(25)-C(18)-P(1)	111.80(17)	F(3)-C(28)-C(27)	115.6(3)
C(25)-C(18)-C(19)	109.9(2)	F(3)-C(28)-F(2)	104.1(6)
C(19)-C(18)-P(1)	109.49(17)	F(5)-C(28)-C(27)	115.5(4)
C(20)-C(21)-C(22)	115.1(2)	F(5)-C(28)-F(4)	101.4(6)
C(3)-C(4)-C(5)	119.5(2)	F(4)-C(28)-C(27)	108.3(3)
C(2)-C(7)-C(8)	121.7(2)	F(6)-C(28)-C(27)	115.7(4)
C(6)-C(7)-C(2)	119.8(2)	F(6)-C(28)-F(5)	109.6(6)
C(6)-C(7)-C(8)	118.5(2)	F(6)-C(28)-F(4)	104.7(6)
C(20)-C(19)-C(18)	115.6(2)	F(8)-C(30)-C(29)	112.1(7)
C(7)-C(8)-P(2)	117.47(16)	F(8)-C(30)F(9)	107.3(8)
C(6)-C(5)-C(4)	119.2(2)	F(8)-C(30)-F(7)	107.1(9)
C(5)-C(6)-C(7)	121.3(2)	F(9)-C(30)-C(29)	113.1(5)
C(16)-C(9)-P(2)	113.20(18)	F(7)-C(30)-C(29)	111.0(5)
C(16)-C(9)-C(10)	108.5(2)	F(7)-C(30)-F(9)	105.9(8)
C(17)-C(9)-P(2)	108.11(17)	F(12)-C(30)-C(29)	111.6(9)
C(17)-C(9)-C(16)	108.3(2)	F(11)-C(30)-C(29)	114.3(12)
C(17)-C(9)-C(10)	109.1(2)	F(11)-C(30)-F(12)	104.5(16)
C(10)-C(9)-P(2)	109.54(17)	F(11)-C(30)-F(10)	108.8(16)
C(19)-C(20)-C(21)	112.7(2)	F(10)-C(30)-C(29)	111.7(8)
C(14)-C(13)-P(2)	108.01(17)	F(10)-C(30)-F(12)	105.3(13)
C(14)-C(13)- C(15)	108.3(2)	C(12)-C(11)-C(10)	112.7(2)
C(14)-C(13)-C(12)	108.7(2)		

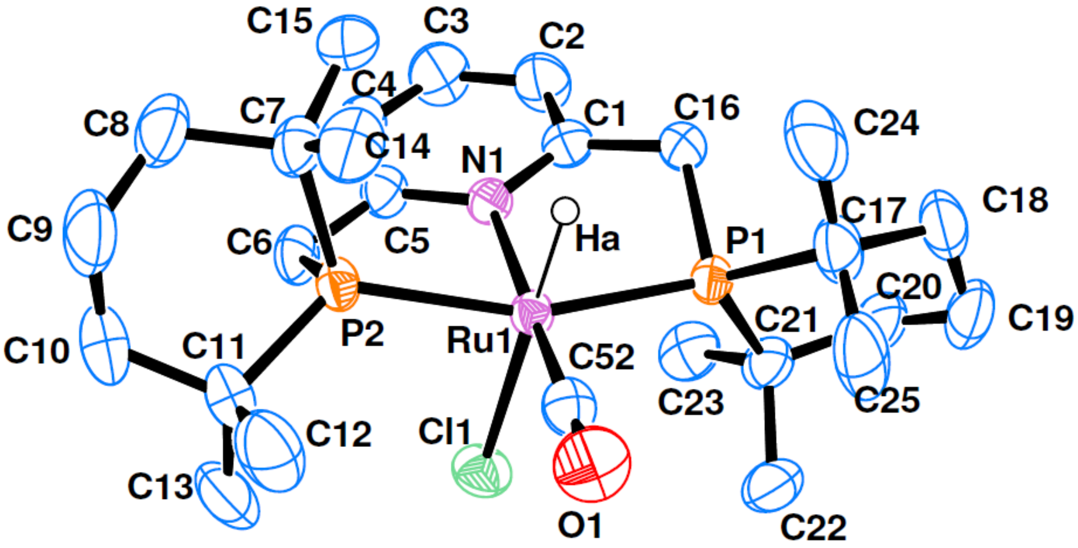

Supplementary Figure 9. Molecular structure of [$^{\mathbf{TMPhos}}$**(PNP)**Ru(CO)(Cl)H], Molecule-1, complex **24**, (thermal ellipsoids are drawn at 50% probability).

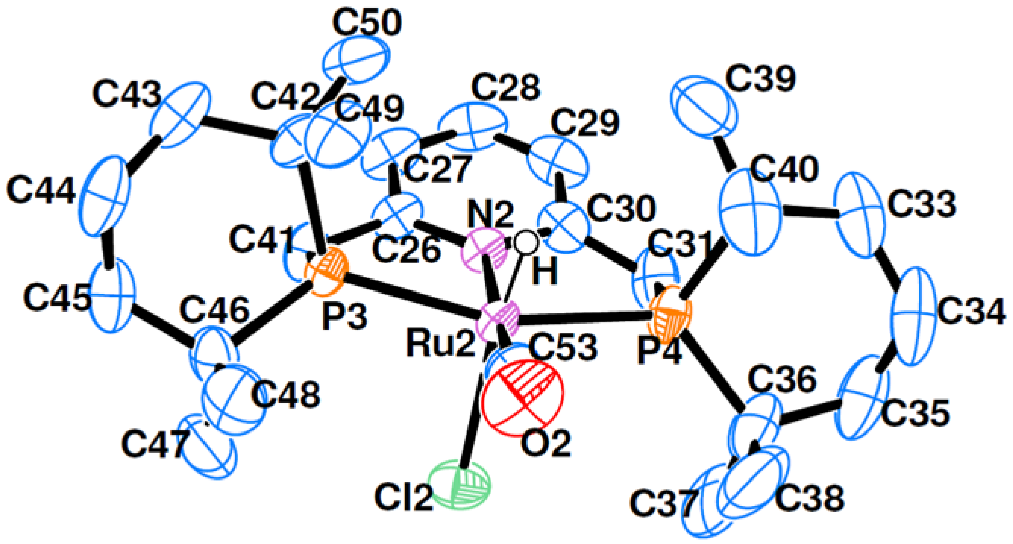

Supplementary Figure 10. Molecular structure of [^{TMPhos}**(PNP)**Ru(CO)(Cl)H], Molecule-2, complex **24**, (thermal ellipsoids are drawn at 50% probability).

Supplementary Table 13. Crystal data and structure refinement for [^{TMPhos}**(PNP)**Ru(CO)(Cl)H], complex **24**.

CCDC code	2182399	Absorption coefficient	6.996 mm^{-1}
Empirical formula	$C_{53}H_{90}Cl_4N_2O_2P_4Ru_2$	F(000)	2616.0
Formula weight	1255.08	Crystal size	0.591 x 0.475 x 0.103 mm
Temperature	293(2) K	Theta range for data collection	5.206 to 144.134°
Wavelength	1.54184 Å (CuKα)	Limiting indices	−17<=h<=16, -14<=k<=14, −41<=l<=41
Crystal system, space group	monoclinic, $P2_1/n$	Reflections collected / unique	80388 / 11763 [R(int) = 0.0641]
Unit cell dimensions	a = 14.68140(10) Å α = 90°	Data / restraints / parameters	11763 / 0 / 638
	b = 12.07460(10) Å β = 91.8770(10)°	Goodness-of-fit on F^2	1.045
	c = 33.9685(2) Å γ = 90°	Final R indices [I>2σ(I)]	R_1 = 0.0438, wR_2 = 0.1216
Volume	6018.43(7) Å^3	R indices (all data)	R_1 = 0.0452, wR_2 = 0.1230
Z, Calculated density	4, 1.385 g/cm^3	Largest diff. peak and hole	1.28 and −1.19 e.A^{-3}

Supplementary Table 14. Bond lengths [Å] and angles [°] for [^{TMPhos}**(PNP)**Ru(CO)(Cl)H], complex **24**.

Ru(1)-P(2)	2.3289(8)	P(1)-C(16)	1.850(3)
Ru(1)-Cl(1)	2.5463(8)	P(1)-C(17)	1.881(4)
Ru(1)-P(1)	2.3285(8)	P(4)-C(32)	1.887(4)
Ru(1)-N(1)	2.154(3)	P(4)-C(31)	1.837(4)
Ru(1)-C(52)	1.843(4)	P(4)-C(36)	1.878(5)
Ru(2)-P(3)	2.3412(8)	C(42)-C(50)	1.524(6)
Ru(2)-Cl(2)	2.5471(9)	C(1)-C(16)	1.511(5)
Ru(2)-P(4)	2.3379(9)	C(1)-C(2)	1.381(5)
Ru(2)-N(2)	2.176(3)	C(5)-C(6)	1.499(5)
Ru(2)-C(53)	1.836(4)	C(5)-C(4)	1.387(5)
P(3)-C(42)	1.884(4)	C(11)-C(12)	1.541(6)
P(3)-C(46)	1.890(4)	C(11)-C(10)	1.543(6)
P(3)-C(41)	1.838(3)	C(11)-C(13)	1.522(6)
P(2)-C(7)	1.875(4)	C(27)-C(28)	1.374(7)
P(2)-C(11)	1.878(4)	C(30)-C(29)	1.397(6)
P(2)-C(6)	1.845(4)	C(30)-C(31)	1.496(6)
P(1)-C(21)	1.873(4)	C(46)-C(45)	1.542(6)

C(46)-C(47)	1.541(7)
C(46)-C(48)	1.534(6)
C(4)-C(3)	1.382(6)
C(29)-C(28)	1.367(7)
C(2)-C(3)	1.377(6)
C(43)-C(44)	1.521(7)
C(17)-C(18)	1.554(6)
C(17)-C(25)	1.538(6)
C(17)-C(24)	1.536(7)
C(20)-C(19)	1.529(7)
N(1)-C(1)	1.349(4)
N(1)-C(5)	1.356(4)
N(2)-C(26)	1.353(5)
N(2)-C(30)	1.346(4)
O(1)-C(52)	1.146(5)
O(2)-C(53)	1.140(5)
C(26)-C(27)	1.392(5)
C(26)-C(41)	1.498(5)
C(21)-C(22)	1.545(5)
C(21)-C(23)	1.539(6)
C(21)-C(20)	1.531(5)
C(7)-C(15)	1.530(6)
C(7)-C(14)	1.527(5)
C(7)-C(8)	1.537(5)
C(42)-C(43)	1.549(5)
C(42)-C(49)	1.538(5)
C(32)-C(33)	1.543(6)
C(32)-C(39)	1.516(8)
C(32)-C(40)	1.541(6)
C(8)-C(9)	1.518(7)
C(10)-C(9)	1.524(7)
C(44)-C(45)	1.502(7)
C(18)-C(19)	1.502(8)
C(33)-C(34)	1.516(9)
C(36)-C(35)	1.531(7)
C(36)-C(37)	1.527(8)
C(36)-C(38)	1.549(9)
C(34)-C(35)	1.503(9)
C(51A)-Cl(3)	1.696(10)
C(51A)-Cl(4)	1.707(11)
Cl(3)-C(51B)	1.734(14)
Cl(4)-C(51B)	2.604(12)
P(2)-Ru(1)-Cl(1)	90.67(30)
P(1)-Ru(1)-P(2)	159.92(3)
P(1)-Ru(1)-Cl(1)	97.79(3)
N(1)-Ru(1)-P(2)	80.49(7)
N(1)-Ru(1)-Cl(1)	87.78(7)
N(1)-Ru(1)-P(1)	81.68(7)
C(52)-Ru(1)-P(2)	97.74(11)
C(52)-Ru(1)-Cl(1)	96.37(11)
C(52)-Ru(1)-P(1)	99.36(11)
C(52)-Ru(1)-N(1)	175.52(13)
P(3)-Ru(2)-Cl(2)	94.21(3)
P(4)-Ru(2)-P(3)	159.68(4)
P(4)-Ru(2)-Cl(2)	96.97(3)
N(2)-Ru(2)-P(3)	81.62(8)
N(2)-Ru(2)-Cl(2)	87.40(8)
N(2)-Ru(2)-P(4)	82.00(8)
C(53)-Ru(2)-P(3)	97.41(12)
C(53)-Ru(2)-Cl(2)	99.37(13)
C(53)-Ru(2)-P(4)	97.44(13)
C(53)-Ru(2)-N(2)	173.22(14)

C(42)-P(3)-Ru(2)	115.93(13)
C(50)-C(42)-P(3)	109.5(3)
C(50)-C(42)-C(43)	108.4(3)
C(50)-C(42)-C(49)	107.6(4)
N(1)-C(1)-C(16)	117.1(3)
N(1)-C(1)-C(2)	121.3(3)
C(2)-C(1)-C(16)	121.6(3)
O(1)-C(52)-Ru(1)	176.8(3)
N(1)-C(5)-C(6)	117.4(3)
N(1)-C(5)-C(4)	120.7(3)
C(4)-C(5)-C(6)	121.9(3)
C(12)-C(11)-P(2)	109.1(3)
C(12)-C(11)-C(10)	110.8(4)
C(10)-C(11)-P(2)	110.0(3)
C(13)-C(11)-P(2)	110.7(3)
C(13)-C(11)-C(12)	106.9(4)
C(13)-C(11)-C(10)	108.2(4)
C(28)-C(27)-C(26)	119.2(4)
O(2)-C(53)-Ru(2)	177.0(4)
N(2)-C(30)-C(29)	120.8(4)
N(2)-C(30)-C(31)	116.9(3)
C(29)-C(30)-C(31)	122.1(4)
C(42)-P(3)-C(46)	104.77(18)
C(46)-P(3)-Ru(2)	125.36(14)
C(41)-P(3)-Ru(2)	99.50(11)
C(41)-P(3)-C(42)	104.18(18)
C(41)-P(3)-C(46)	104.32(19)
C(7)-P(2)-Ru(1)	116.32(11)
C(7)-P(2)-C(11)	104.46(18)
C(11)-P(2)-Ru(1)	126.03(14)
C(6)-P(2)-Ru(1)	97.81(11)
C(6)-P(2)-C(7)	104.40(17)
C(6)-P(2)-C(11)	104.89(18)
C(21)-P(1)-Ru(1)	122.32(12)
C(21)-P(1)-C(17)	105.23(19)
C(16)-P(1)-Ru(1)	98.63(11)
C(16)-P(1)-C(21)	102.47(17)
C(16)-P(1)-C(17)	104.43(18)
C(17)-P(1)-Ru(1)	120.30(14)
C(32)-P(4)-Ru(2)	116.69(13)
C(31)-P(4)-Ru(2)	100.41(13)
C(31)-P(4)-C(32)	103.6(2)
C(31)-P(4)-C(36)	104.0(2)
C(36)-P(4)-Ru(2)	124.13(19)
C(36)-P(4)-C(32)	105.1(2)
C(1)-N(1)-Ru(1)	120.7(2)
C(1)-N(1)-C(5)	119.5(3)
C(5)-N(1)-Ru(1)	119.8(2)
C(26)-N(2)-Ru(2)	119.8(2)
C(30)-N(2)-Ru(2)	120.1(2)
C(30)-N(2)-C(26)	119.1(3)
N(2)-C(26)-C(27)	121.4(4)
N(2)-C(26)-C(41)	117.2(3)
C(27)-C(26)-C(41)	121.3(3)
C(22)-C(21)-P(1)	108.7(3)
C(23)-C(21)-P(1)	107.7(2)
C(23)-C(21)-C(22)	108.6(4)
C(20)-C(21)-P(1)	111.7(3)
C(20)-C(21)-C(22)	111.0(3)
C(20)-C(21)-C(23)	109.1(3)
C(15)-C(7)-P(2)	109.8(3)
C(15)-C(7)-C(8)	107.3(3)
C(14)-C(7)-P(2)	110.3(3)

C(14)-C(7)-C(15)	107.6(3)	C(40)-C(32)-C(33)	110.1(4)
C(45)-C(46)-P(3)	110.4(3)	C(30)-C(31)-P(4)	112.4(2)
C(47)-C(46)-P(3)	110.0(3)	C(9)-C(8)-C(7)	115.6(4)
C(47)-C(46)-C(45)	108.2(4)	C(2)-C(3)-C(4)	118.9(4)
C(48)-C(46)-P(3)	107.7(3)	C(9)-C(10)-C(11)	115.6(4)
C(48)-C(46)-C(45)	111.4(4)	C(45)-C(44)-C(43)	110.7(4)
C(48)-C(46)-C(47)	109.1(4)	C(44)-C(45)-C(46)	115.7(4)
C(5)-C(6)-P(2)	110.8(2)	C(19)-C(18)-C(17)	115.6(4)
C(1)-C(16)-P(1)	111.2(2)	C(18)-C(19)-C(20)	111.7(3)
C(3)-C(4)-C(5)	119.8(3)	C(34)-C(33)-C(32)	116.0(5)
C(28)-C(29)-C(30)	119.8(4)	C(35)-C(36)-P(4)	111.7(4)
C(3)-C(2)-C(1)	119.7(4)	C(35)-C(36)-C(38)	112.1(5)
C(44)-C(43)-C(42)	116.0(4)	C(37)-C(36)-P(4)	110.3(4)
C(26)-C(41)-P(3)	111.1(2)	C(37)-C(36)-C(35)	108.6(5)
C(18)-C(17)-P(1)	110.4(3)	C(37)-C(36)-C(38)	107.2(6)
C(25)-C(17)-P(1)	108.6(3)	C(38)-C(36)-P(4)	106.8(3)
C(25)-C(17)-C(18)	110.4(4)	C(14)-C(7)-C(8)	111.1(3)
C(24)-C(17)-P(1)	110.4(4)	C(8)-C(7)-P(2)	110.83)
C(24)-C(17)-C(18)	108.5(4)	C(43)-C(42)-P(3)	111.1(3)
C(24)-C(17)-C(25)	108.5(4)	C(49)-C(42)-P(3)	109.9(3)
C(19)-C(20)-C(21)	115.6(4)	C(49)-C(42)-C(43)	110.3(3)
C(29)-C(28)-P(27)	119.4(4)	C(8)-C(9)-C(10)	112.0(4)
C(33)-C(32)-P(4)	110.9(3)	C(35)-C(34)-C(33)	112.0(5)
C(39)-C(32)-P(4)	109.4(3)	C(34)-C(35)-C(36)	115.7(4)
C(39)-C(32)-C(33)	108.7(4)	Cl(3)-C(51A)-Cl(4)	113.0(5)
C(39)-C(32)-C(40)	108.6(4)	Cl(3)-C(51B)-Cl(44)	78.9(4)
C(40)-C(32)-P(4)	109.2(3)		

4. Buried Volume Calculations

Molecular structures obtained from X-ray crystallography (Complexes **23** and **24**) or from the Cambridge Crystallographic Data Centre (CCDC references: #162523 and #1849515) were subjected to ligand buried volume calculations using SambVca 2.1.[12] The Pd or Ru atom was set as the coordination centre, the P donor atoms (and N in case of complex **23**) were selected for z-axis definition (z-negative) and the carbon atoms attached to coordinating P or N were selected for xz-plane definition. Except the atoms corresponding to coordinating ligands, all atoms (metal, solvent, etc.) were deleted and thus not considered in the buried volume calculation. Atomic radii was set at Bondi radii and sphere radius was set to 3.5 Å. Mesh spacing for numerical integration set to 0.10 Å and hydrogen atoms were omitted from the calculation.

Complex 23, [(BTMPX)Pd(O_2CCF_3)$_2$]

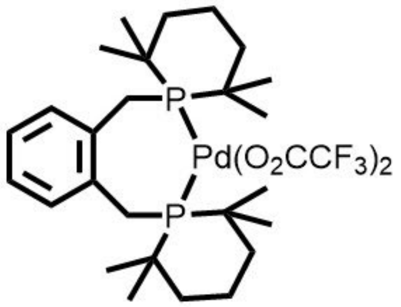

%V Free	%V Buried	% V Tot/V Ex
50.0	50.0	99.9

Quadrant	V f	V b	V t	%V f	%V b
SW	17.1	27.8	44.9	38.1	61.9
NW	28.1	16.8	44.9	62.5	37.5
NE	17.0	27.8	44.9	38.0	62.0
SE	27.6	17.2	44.9	61.6	38.4

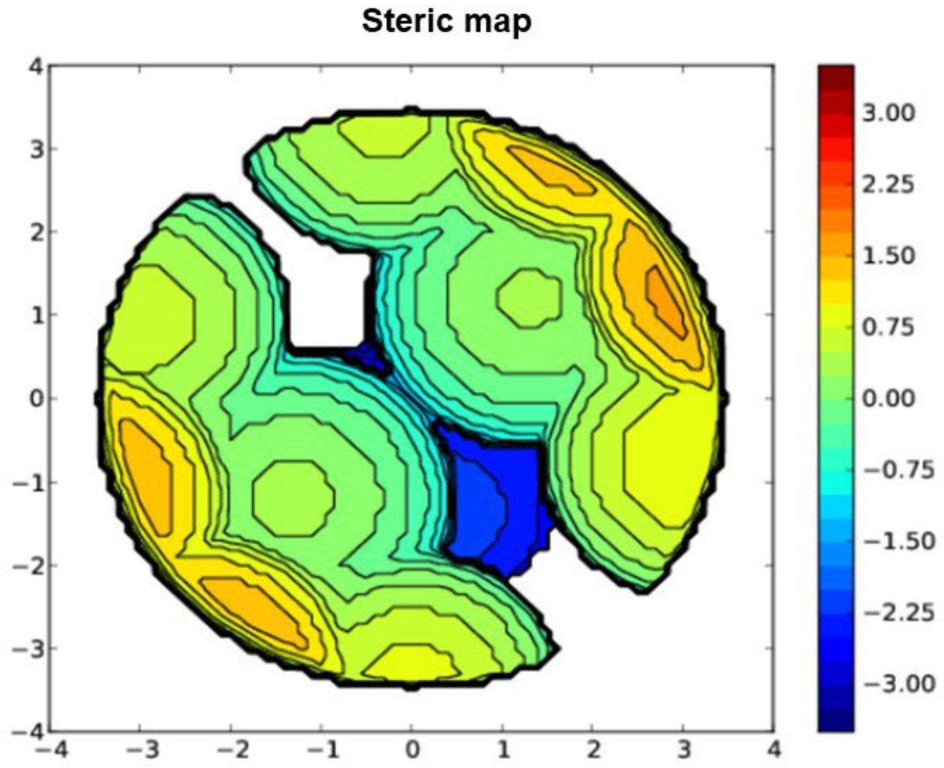

Supplementary Figure 11. Ligand buried volume steric map for complex 23, [(BTMPX)Pd(O_2CCF_3)$_2$]

Complex 24 – Molecule 1, [^{TMPhos}(PNP)Ru(CO)(Cl)H]

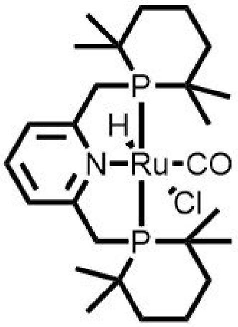

%V Free	%V Buried	% V Tot/V Ex
48.2	51.8	99.9

Quadrant	V f	V b	V t	%V f	%V b
SW	23.0	21.8	44.9	51.4	48.6
NW	23.3	21.5	44.9	52.0	48.0
NE	20.1	24.8	44.9	44.7	55.3
SE	20.0	24.9	44.9	44.5	55.5

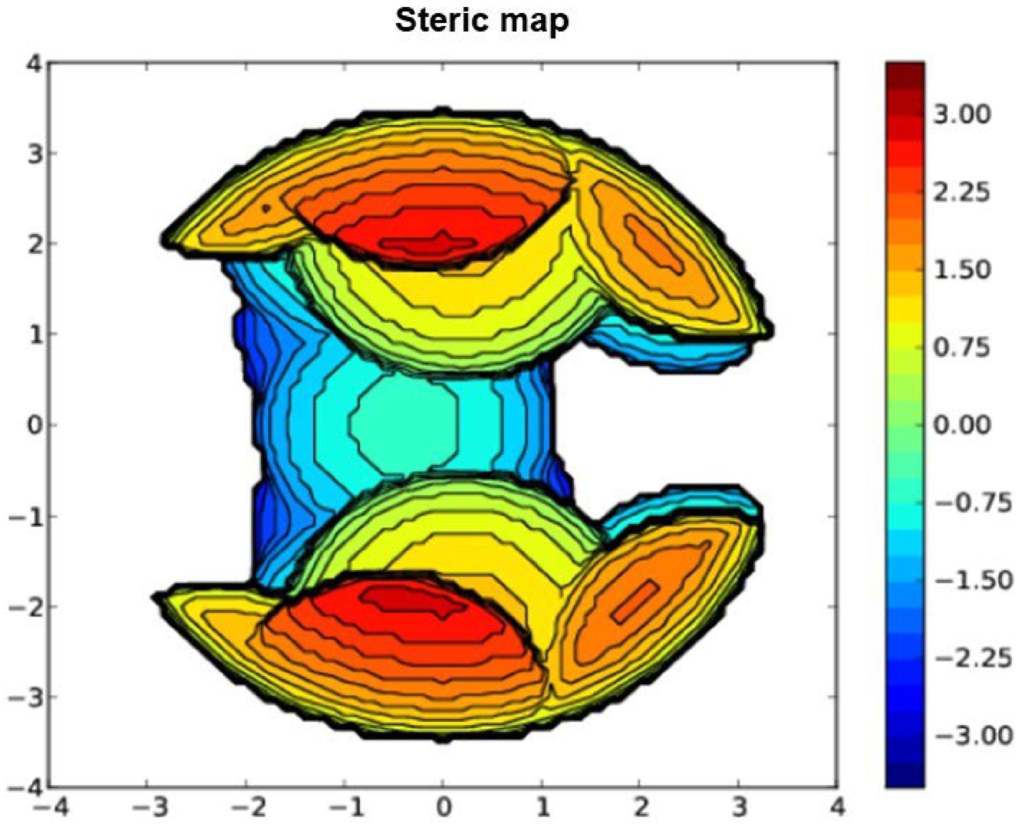

Supplementary Figure 12. Ligand buried volume steric map for complex 24, molecule 1, [^{TMPhos}(PNP)Ru(CO)(Cl)H].

Complex 24 – Molecule 2, [TMPhos(PNP)Ru(CO)(Cl)H]

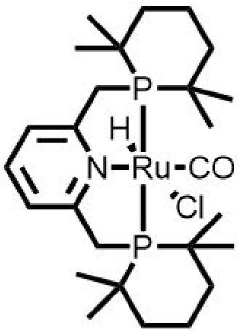

%V Free	%V Buried	% V Tot/V Ex
48.1	51.9	99.9

Quadrant	V f	V b	V t	%V f	%V b
SW	25.4	19.5	44.9	56.6	43.4
NW	19.7	25.2	44.9	43.9	56.1
NE	23.7	21.2	44.9	52.8	47.2
SE	17.5	27.4	44.9	39.0	61.0

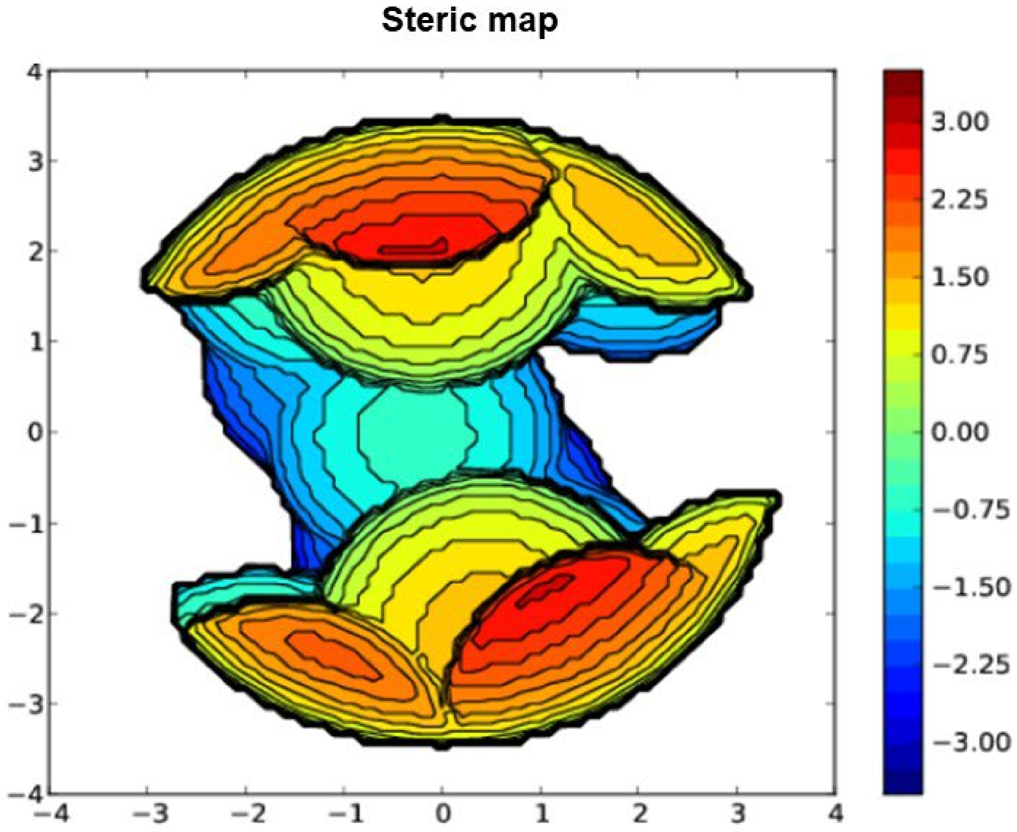

Supplementary Figure 13. Ligand buried volume steric map for complex 24, molecule 2, [TMPhos(PNP)Ru(CO)(Cl)H].

tBu_Complex 23, [(DTBPX)Pd(O_3SCH_3)$_2$]

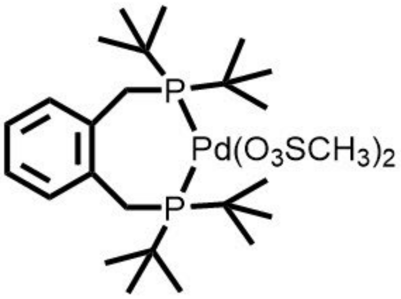

%V Free	%V Buried	% V Tot/V Ex
46.9	53.1	99.9

Quadrant	V f	V b	V t	%V f	%V b
SW	23.6	21.2	44.9	52.7	47.3
NW	18.1	26.7	44.9	40.4	59.6
NE	24.2	20.6	44.9	54.0	46.0
SE	18.1	26.7	44.9	40.5	59.5

Steric map

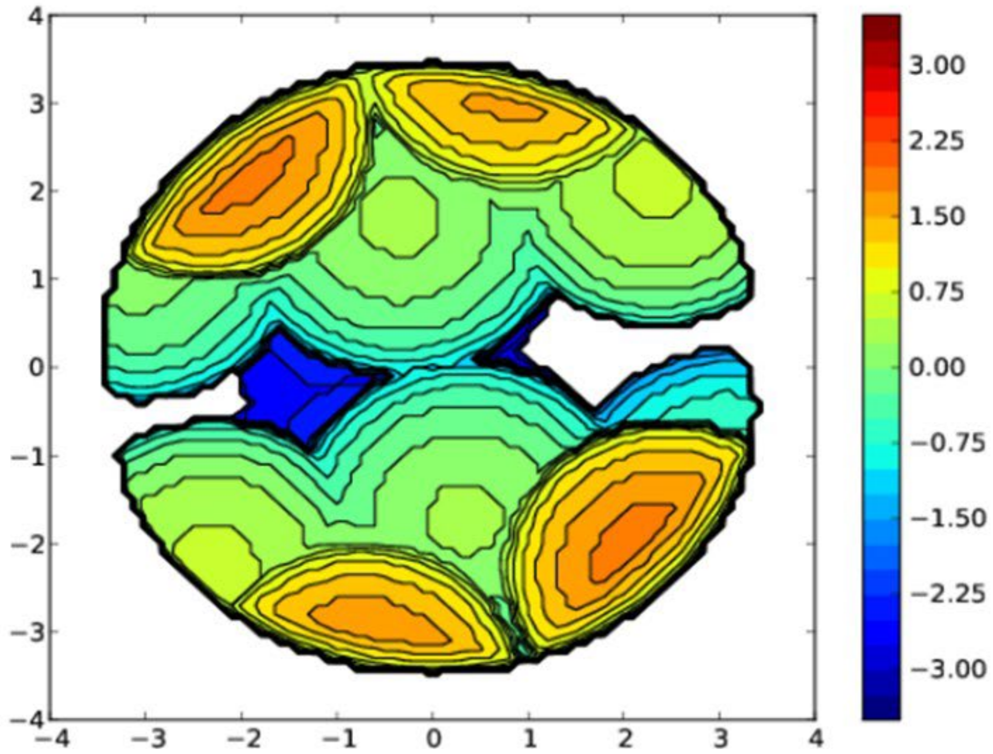

Supplementary Figure 14. Ligand buried volume steric map for [(DTBPX)Pd(O_3SCH_3)$_2$], CCDC #162523.

tBu_Complex 24, [^{tBu}(PNP)Ru(CO)(Cl)H]

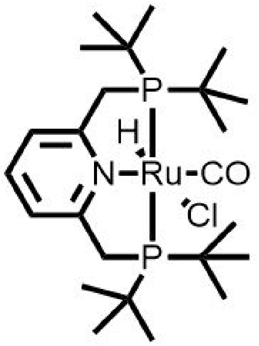

%V Free	%V Buried	% V Tot/V Ex
46.7	53.3	99.9

Quadrant	V f	V b	V t	%V f	%V b
SW	24.2	20.7	44.9	53.9	46.1
NW	18.7	26.2	44.9	41.6	58.4
NE	23.6	21.2	44.9	52.7	47.3
SE	17.3	27.6	44.9	38.5	61.5

Steric map

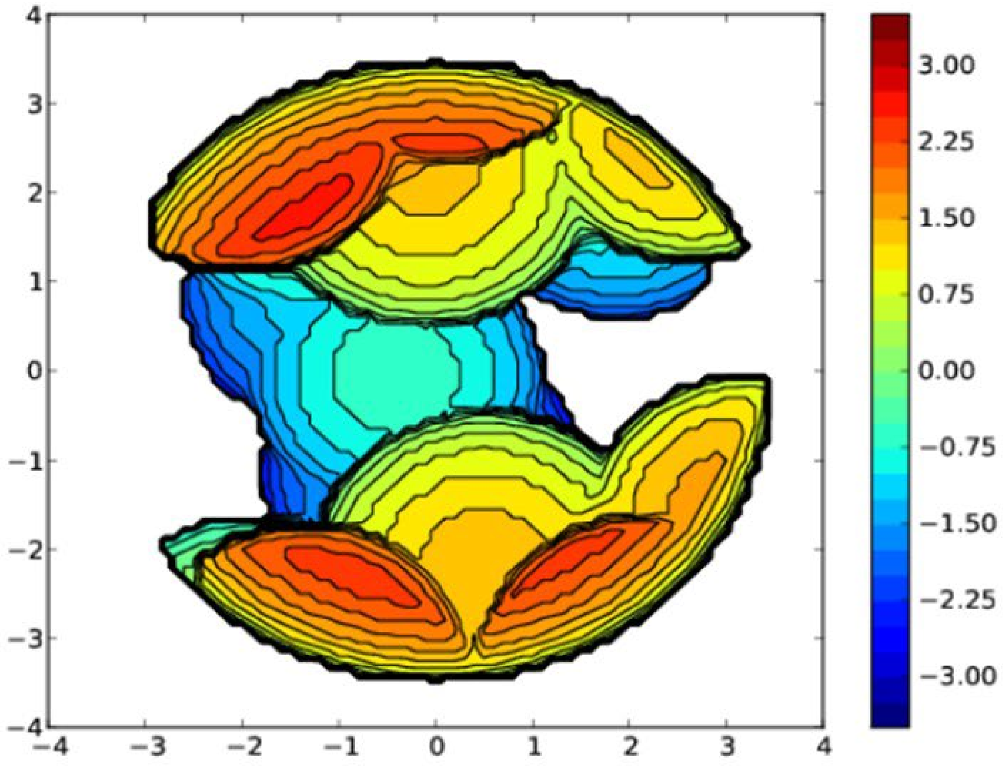

Supplementary Figure 15. Ligand buried volume steric map for [^{tBu}(PNP)Ru(CO)(Cl)H], CCDC #1849515.

5. Calculation of Tolman Cone Angles

The cone angle was calculated for the terminal **TMPhos** from the X-ray structure of complex **15** and the analogous HP^{t}Bu$_2$ complex (CCDC number: #887216) according to the below methodology:

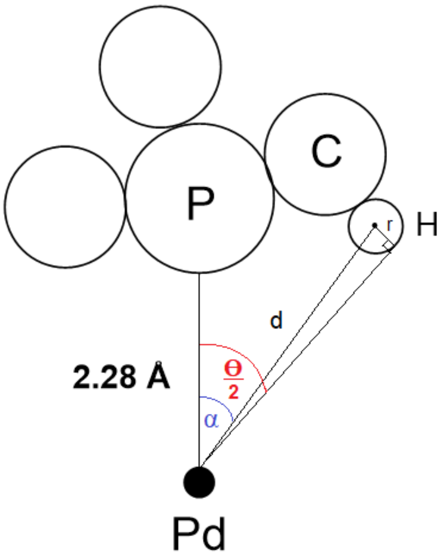

Supplementary Figure 16. Definition of parameters for the calculation of Tolman Cone Angles (°).

Due to the asymmetric nature of TMPhos, substituent half-angles, $\frac{\theta_i}{2}$, were first measured for each substituent connected to the P donor atom. The average of the 3 measured half-angles was then doubled to get the overall Tolman Cone Angles (°), Θ. Represented in an equation:

$$\theta = \frac{2}{3}\sum_i \frac{\theta_i}{2}$$

$$\frac{\theta_i}{2} = \alpha + \sin^{-1}\frac{r}{d} \times \frac{180}{\pi}$$

Where,

Θ = Tolman Cone Angle (°)

$\frac{\theta_i}{2}$ = Substituent half-angle (°)

α = Measured P-Pd-H angle (°)

r = Van der Waals radii of hydrogen (1.00 Å)[13]

d = Measured Pd-H distance (Å)

Supplementary Table 15. Cone Angle Calculation for Complex **15**.

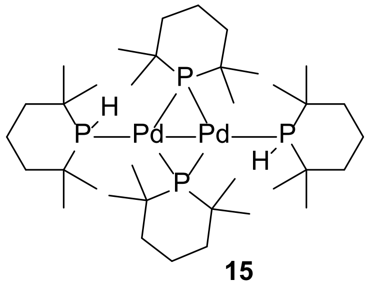

15

α (P-Pd-H) angle	r-H-radii	d (Pd-H)	Asin	θ/2	Average	Cone angle (°)

θ1	58.5	1	3.32	17.6	76.0		
θ2	56.3	1	3.47	16.8	73.1		
θ3	21.6	1	3.08	19.0	40.6	63.2	**126.5**

Supplementary Table 16. Cone Angle Calculation for CCDC # 887216.[14]

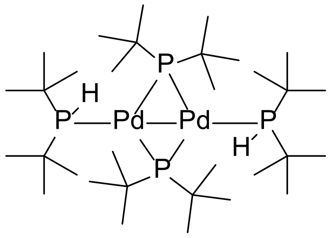

α (P-Pd-H) angle		r-H-radii	d (Pd-H)	Asin	θ/2	Average	Cone angle (°)
θ1	61.3	1	3.07	19.0	80.3		
θ2	61.5	1	3.04	19.2	80.7		
θ3	21.4	1	3.01	19.4	40.8	67.3	**134.6**

Supplementary References

1. Cristau, H.-J., Coulombeau, A., Genevois-Borella, A., Sanchez, F. & Pirat, J.-L. Preparation of phosphinodipeptide analogs as building blocks for pseudopeptides synthesis. J. Organomet. Chem. 643-644, 381-391 (2002).
2. Uryu, M. *et al*. Synthesis of Polybenzoacenes: Annulative Dimerization of Phenylene Triflate by Twofold C−H Activation. Angew. Chem. Int. Ed. 59, 6551-6554 (2020).
3. Rucklidge, A. J., Morris, G. E., Slawin, A. M. Z. & Cole-Hamilton, D. J. The Methoxycarbonylation of Vinyl Acetate Catalyzed by Palladium Complexes of [1,2-Phenylenebis(methylene)]bis[di(tert-butyl)phosphine]. Helv. Chim. Acta 89, 1783-1800 (2006).
4. Cabezon, B. *et al*. Self-Complementary [2]Catenanes and Their Related [3]Catenanes. Chem. Eur. J. 6, 2262-2273 (2000).
5. APEX4 Data Collection Software (Version 2021.4-0), Bruker AXS Inc., Madison, Wisconsin, USA, 2021.
6. SAINT Data Reduction Software (Version 8.40b), Bruker AXS Inc., Madison, Wisconsin, USA, 2019.
7. G.M. Sheldrick, SADABS. Program for Empirical Absorption Correction, University of Gottingen, Germany, 1996.
8. Rigaku Oxford Diffraction, C. S. S., version 1.171.38.46; Rigaku Corporation, Oxford, UK, 2018. CrysAlisPro Software System, version 1.171.38.46; 2018.
9. Sheldrick, G. M. A short history of SHELX, *Acta Crystallogr. A.* 2008, **64**, 112−122.
10. Sheldrick, G. M. Crystal structure refinement with SHELXL, Acta Crystallogr. *C.* 2015, **71**, 3−8.
11. Dolomanov, O. V. *et al.* H. OLEX2 : a complete structure solution, refinement and analysis program *J. Appl. Crystallogr.* 2009, **42**, 339−341.
12. Falivene L. *et al. Nat. Chem*., **2019**, 11, 872–879.
13. Müller, T. E. & Mingos, D. M. P. Determination of the Tolman cone angle from crystallographic parameters and a statistical analysis using the crystallographic data base. *Transition Met. Chem.* 20, 533-539 (1995).
14. Breunig, J., Lerner, H.-W. and Bolte, M. A triclinic polymorph of bis([mu]-di-tert-butylphosphanido)bis[(di-tert-butylphosphane)palladium(I)], *Acta Cryst. Sec. E*, **68**, m836, (2012).
